# Attention to Social and Non-Social Stimuli in a Continuous Performance Test in Autistic and Typically Developed Participants: An ERP Study

**DOI:** 10.1007/s10803-025-06825-9

**Published:** 2025-04-16

**Authors:** Samaneh S. Dastgheib, Jürgen M. Kaufmann, Andrea E. Kowallik, Stefan R. Schweinberger

**Affiliations:** 1 Department for General Psychology and Cognitive Neuroscience, Institute of Psychology, Friedrich Schiller University of Jena, Am Steiger 3/1, 07743 Jena, Germany; 2 Social Potentials in Autism Research Unit, Friedrich Schiller University of Jena, Leutragraben 1, 07743 Jena, Germany; 3 Department of Psychiatry, Jena University Hospital, 07743 Jena, Germany; 4 Center for Intervention and Research on Adaptive and Maladaptive Brain Circuits Underlying, Mental Health (C-I-R-C), Jena-Magdeburg-Halle, Germany; 5 German Center for Mental Health (DZPG), Site Jena-Magdeburg-Halle, Germany

**Keywords:** Autism spectrum disorder, Continuous performance test, Event-related potentials

## Abstract

Autism spectrum disorder (ASD) is characterized by a range of symptoms, including restrictive behaviors and deficient social skills. We investigated EEG correlates of social attention, face, and non-face perception by applying a continuous performance test (CPT) with two different sets of stimuli (letters and faces). The CPT required participants to respond to a specific target stimulus (e.g., “X”) only when it followed a specific preceding stimulus (e.g., “O”). Event-related potential (ERP) components, including P100, N170, P200, N250, P300, and continuous negative variation (CNV), were analyzed in 19 young adults with ASD and 19 typically developed (TD) individuals that were matched for intelligence, age, and gender. TD participants had higher accuracies only for the target condition and regardless of stimulus type. No ERP differences between the two groups were found for the CPT with letter stimuli. By contrast, autistic individuals exhibited lower amplitudes of P300 and CNV during face CPT. Results suggest diminished allocation of attentional resources and response preparation towards socially relevant face stimuli.

**Supplementary Information:**

The online version contains supplementary material available at 10.1007/s10803-025-06825-9.

## Introduction

Humans tend to pay attention to social stimuli, such as faces, eyes, bodies, and other nonverbal communicative signals (e.g., Langton et al., 2008). This attentional bias toward the social world manifests early in life and is key to social cognitive development (Chita-Tegmark, 2016a; Gliga & Csibra, 2007; Pascalis et al., 2011; Sifre et al., 2018). Face and emotion recognition, mental state attribution, joint attention, empathy, and social decision-making are all different facets of social cognition (Frith, 2008). Accordingly, social attention (1) involves extensive brain networks involving areas integrating face and gaze perception, attention, emotion, and mental state attribution (Klein et al., 2009), and (2) can be altered in various conditions, including autism spectrum disorder (ASD), for which social attention alterations even have been suggested as a potential biomarker (Chita-Tegmark, 2016b).

ASD includes a spectrum of conditions characterized by social communication impairments and restrictive, repetitive behaviors. Those core characteristics can be accompanied by sensory changes (Simmons et al., 2009) and also by various comorbidities, the most frequent of which include attention deficit hyperactivity disorder (ADHD), depression, anxiety (Lord et al., 2020; Sharma et al., 2018). At the same time, variability in behavioral symptoms has led researchers to variably emphasize different aspects of ASD, including impairments in face perception (Nomi & Uddin, 2015; Weigelt et al., 2012), atypical social attention (Chita-Tegmark, 2016b) deficits in executive functions (Chan et al., 2009), lack of mirroring activity (Yates & Hobson, 2020) or atypical empathy (Tordjman et al., 2019).

Attention, as a domain of executive functions, has been long investigated in ASD. Studies looked at attention impairment along with response preparation, inhibitory processes, conflict monitoring, and flexibility (Craig et al., 2016). Evidence for altered response preparation has been found using event-related potentials (ERPs) (Hoyland et al., 2017; Tye et al., 2014). ERPs are immensely advantageous in exploring the neural correlates of executive functions and enlighten us on cognitive operations involving preparedness for action execution, inhibiting inappropriate responses, and conflict monitoring. The Go-NoGo paradigm, particularly the continuous performance test (CPT), represents a useful ERP tool for studying these functions. A trial of the cued CPT paradigm consists of two stimuli: the first stimulus (S1) serves as a cue for the secondary imperative stimulus (S2) that might or might not require a response, in the sense that participants are to respond to, say, the letter X only, and only if X has been immediately preceded by the letter O (Hoyland et al., 2017; Tye et al., 2014). A cued CPT elicits a variety of ERPs that are substantially informative about the neural substrate of sustained attention and impulsivity. Among those is the Contingent Negative Variation (CNV), characterized by gradual negative voltage shifts between S1 and S2 associated with response preparation (Brunia et al., 2011; Riccio et al., 1999). The CNV reflects both attention and motor preparation for the upcoming action with respect to S2; it is also considered to be related to response time, and its amplitude may represent the neuronal substrates engaged in the preparatory processes (Brunia et al., 2011; Hoyland et al., 2017; Riccio et al., 1999). Using the CPT paradigm, though with different stimulus types, both Tye and Hoyland found enhanced (i.e., more negative) CNV amplitudes in ASD individuals (Hoyland et al., 2017; Tye et al., 2014). Thillay et al. also found an enhanced CNV in people with autism, but only for non-predictable target stimuli, suggesting an altered top-down response preparation in ASD compared to typically developed (TD) individuals (Thillay et al., 2016).

The P300 ERP component is a positive deflection at centro-pariatal electrode sites, peaking roughly 300 ms post-stimulus onset, and has been linked to various processes including stimulus classification, memory updating, context closure, and response selection. In NoGo scenarios – when the participant has been shown the cue and thus has prepared a response but then is supposed to withhold it – P300 reflects the inhibitory process following the presentation of S2. As its magnitude mirrors the degree of attentional resources used, P300 can increase when the participant puts more effort into the task. Conversely, P300 amplitude can diminish when the significance of the stimulus is vague or its classification is difficult (Hoyland et al., 2017; Polich, 2011). Thus, the precise relationship between attention and P300 amplitude remains controversial (Polich, 2007), and this may be in part because different aspects of resource allocation (i.e., task difficulty and task emphasis) can have opposite effects on P300 amplitude (Kok, 2001). Interestingly, although a meta-analysis concluded attenuated P300 in ASD compared to TD individuals (Cui et al., 2017), in the context of a CPT, neither Tye et al., with letter stimuli, nor Hoyland et al., with facial emotions, found such a difference (Hoyland et al., 2017; Tye et al., 2014).

The present work investigated ERP components using two structurally identical CPT paradigms but with two stimulus types: letters and human faces. Thus, we tapped into different skills and processes, including sustained and social attention, executive functions such as response preparation, and face discrimination/recognition. Our approach applying a social (face) and a non-social (letter) CPT allows for investigating nuanced differences between both types of stimulus processing and potentially promotes a better understanding of the cognitive profile in autism. Given the social deficits in autism, we hypothesized that differences between TD and autistic individuals should be either specific to, or particularly prominent in, the face CPT experiment. In addition to longer response times and lower accuracy in the autistic group, we also expected lower (i.e., less positive) P300 and lower (i.e., less negative) CNV amplitudes in ASD, as CNV enhancement has been shown to be linked to performance (Brunia et al., 2011). In short, we expected to see the attentional target effect (i.e., OX vs. O.NotX) to reflect larger ERP amplitudes across ERP components. In addition, considering the research in the field of face perception and autism (Khorrami et al., 2013), we hypothesized that smaller ERP amplitudes in ASD individuals for the face (but not letter) CPT would provide further information regarding a putative change of social attention in autism.

## Method

### Participants

Nineteen individuals previously diagnosed with high-functioning autism spectrum disorder (ASD) by a professional psychiatrist or psychologist and an equal number of typically developed (TD) individuals, matched in terms of intelligence (IQ), age, handedness, and gender, were included (six females and one gender-diverse individual in each group), aged between 16 and 39 years (*M* = 23.18, *SD* = 6.22), actively participated in the study and provided data. Before data collection, we conducted a power analysis using GPower 3.1.9.7 (Faul et al., 2009; Kang, 2021), aimed to achieve a power of 0.80, enabling the detection of a medium effect, *η*_*p*_^*2*^ = 0.1 for a main effect of group (*F*-test, ANOVA with repeated measures, between factors), at an alpha level of 0.05. GPower indicated that 13 participants per group would be necessary, assuming equal group sizes (achieved power = 0.806; achieved power with 19 participants per group = 0.912). The study was preregistered on OSF (https://doi.org/10.17605/OSF.IO/NQ9BE). The study was part of a more extensive project investigating multiple facets of social cognition in autism; we decided to collect data from 19 participants per group throughout, as implicated by the power analysis for a different study for which we had planned to recruit the same participants.

Each participant engaged in a two-session process, consisting of a diagnostic session followed by an experimental session, for which they received monetary compensation (€ 2.50 per 15 min). All participants were native German speakers. Typically developed participants reported no neurological or psychiatric diagnoses except one individual who was diagnosed with chronic depression and was matched to an autistic participant who had the same comorbidity diagnosis.

A psychotherapist with certified ADOS- 2 training, co-author AEK, conducted the Autism Diagnostic Observation Schedule, Second Edition (ADOS- 2) for the ASD group, validating the existing diagnosis of ASD. The study adhered to the principles outlined in the Declaration of Helsinki in its current version and received approval from the Ethics Committee of Jena University Hospital (Reg. No. 2018–1156-BO).

### Matching Process

The study aimed for frequency-matching (Stürmer & Brenner, 2001), intending to align the distribution of age, gender, handedness, and intelligence (assessed via the Wechsler Adult Intelligence Scale–Fourth Edition, WAIS-IV) in the TD group with that of the case group. In practice, the approach resembled perfect individual matching with minor variations. Following the diagnostic session of each participant with ASD and confirmation of their voluntary participation, TDs were selected to match specific characteristics. Some individuals in both the ASD and TD (only one participant) groups had psychiatric comorbidities and were prescribed medication to a limited extent. Table 1 provides a comprehensive overview of demographic *information and questionnaire results. Additional details, including demographics, raw data, statistical analyses, and scripts, are accessible in the supplementary material on the Open Science Framework (OSF) (*https://osf.io/38sae/?view_only=778e9eed8ea14465a92c64559103e45e*).*

**Table 1:** Clinical and demographic characteristics

	ASD (n = 19)	TD (n = 19)	Statistic	*p*	Direction of Difference
Age (years), *M* (*SD*)	23.42 (7.05)	23.00 (5.91)	*W* = 178.00	.955	n.s
Right-handed, *n* (%)	18 (94.74)	18 (94.74)	*X*.^*2*^ = 0.12	.940	n.s
AQ_Total,* M* (*SD*)	32.65 (6.95)	15.26 (3.97)	*W* = 365.50	<.0001	ASD > TD
WAIS_IQ,* M* (*SD*)	102.85 (15.10)	105.95 (11.31)	*t* = − 0.72	.475	n.s
CatQ,* M* (*SD*)	105.70 (21.61)	73.26 (15.25)	*t* = 5.39	<.0001	ASD > TD
TAS26,* M* (*SD*)	51.30 (7.87)	42.95 (12.77)	*t* = 2.47	.018	ASD > TD
WHO_QoL,* M* (*SD*)	3.50 (0.69)	4.00 (0.94)	*W* = 130	.074	n.s
SPF,* M* (*SD*)	40.85 (7.90)	42.58 (6.15)	*t* = − 0.76	.452	n.s
MAIA,* M* (*SD*)	20.81 (4.49)	23.21 (4.87)	*t* = − 1.60	.119	n.s
ADOS- 2, *M* (*SD*)	9 (1)	–	*–*	–	–

*ASD* Autism spectrum disorder, *TD* Typically developed, *AQ* Autism spectrum quotient, *WAIS_IQ* Wechsler adult intelligence Scale (WAIS) intelligence quotient, *CatQ* Camouflaging autistic traits questionnaire, *TAS26* Toronto alexithymia scale 26, *WHO_QoL* WHO quality of life, *SPF* Saarbrücker personality questionnaire, *MAIA* Multidimensional assessment of interoceptive awareness, *ADOS- 2* Autism Diagnostic Observation Schedule second edition, *M* Mean, *SD* Standard deviation, *n.s* Non-significant difference, *W* Wilcoxon test statistic, *t* t statistic, *X*^*2*^ Pearson’s chi-squared statistic. See the OSF project for the subgroups’ scores. Please note that ADOS- 2 was tested only for the ASD group

### Experimental Settings and Stimuli

The diagnostic session included the administration of the Wechsler Adult Intelligence Scale – Fourth Edition (WAIS-IV) and a battery of exploratory questionnaires, namely the Autism Spectrum Quotient (AQ), World Health Organization Quality of Life Questionnaire Short Form (WHOQoL-BREF), Multidimensional Assessment of Interoceptive Awareness Questionnaire (MAIA), Camouflaging Autistic Traits Questionnaire (CAT-Q), Saarbrücker questionnaire (SPF), and Toronto Alexithymia Scale (TAS- 26).

**Fig. 1:**
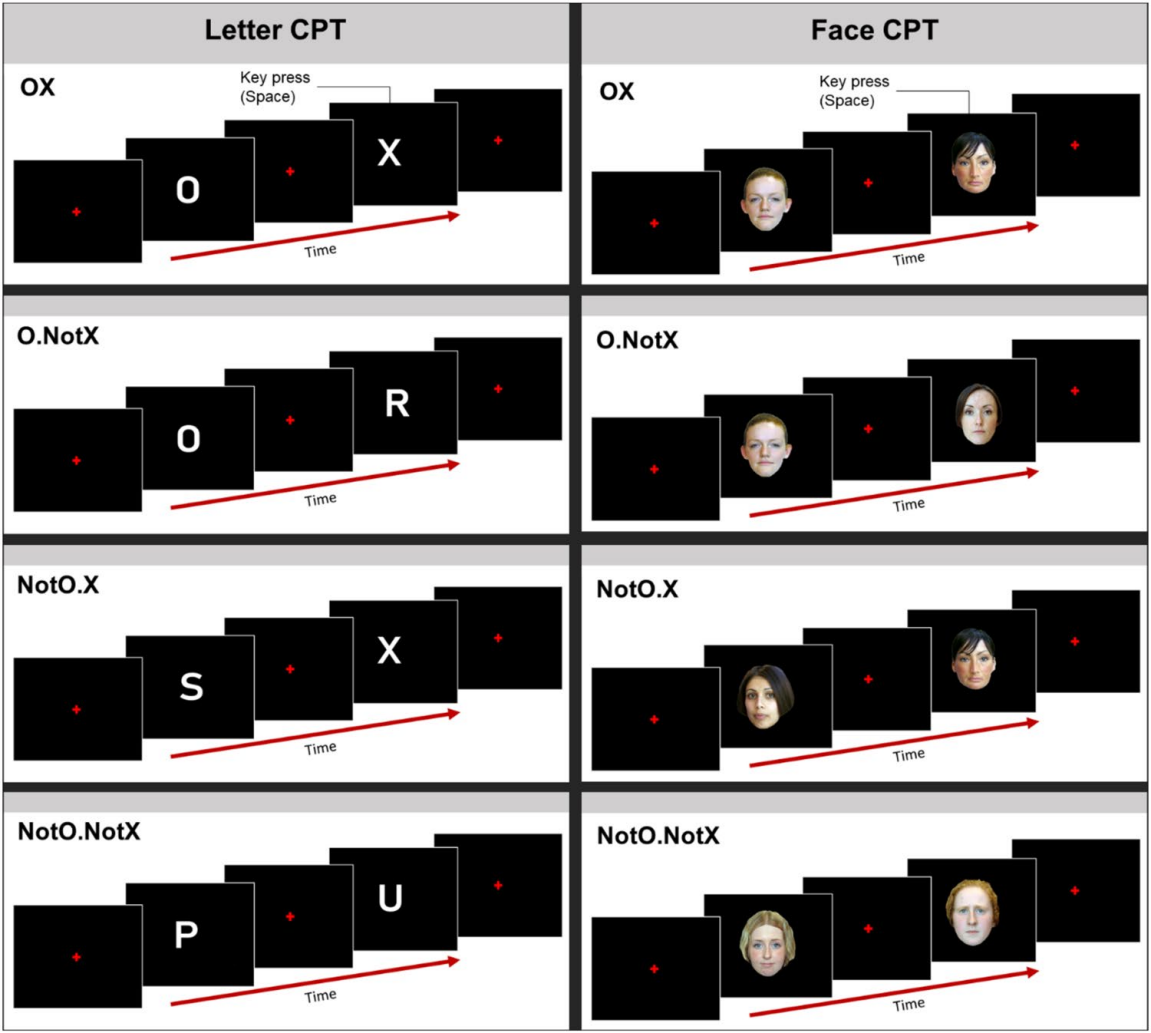
Schematic illustration of conditions and trial sequences in both experiments. Left: Letter CPT. Right: Face CPT. Note that experiment order was fixed, with the Letter CPT experiment preceding the Face CPT experiment

The experimental session of the present study adopted a cued CPT variant, the OX-CPT, which requires participants to respond to the target (S2) “X” but only when it follows a cue stimulus (S1), “O”. Two types of OX-CPT were administered sequentially: a social version (face CPT) featuring a set of 26 naturally distinctive faces and a non-social version (letter CPT) utilizing the 26 letters of the English alphabet. Participants were seated in front of a 19″ color computer monitor (1280 by 1024 pixels), on the center of which they saw a continuous succession of stimuli (letters in the letter CPT block and faces in the face CPT block) on a black background presented with EPrime™ (version 3.0). As measured by a Gossen™ MavoSpot 2 USP luminance meter with 1° measurement field (directed at the center of the stimuli, and using the same viewing distance of 110 cm as participants did), letters had an average luminance of 58.0 cd/m2 (SD = 13.1), and faces had an average luminance of 66.0 cd/m2 (SD = 8.0). Viewing distance was kept constant at 110 cm by a chin rest; stimuli sizes were 8 cm by 12 cm, resulting in viewing angles of 3.09° by 4.58°. Each CPT version included four conditions, for which there were 40 trials per condition per experiment in randomized order. Each trial consisted of the first stimulus (S1), presented for 1000 ms, followed by a red fixation cross, presented for 1000 ms, and the second stimulus (S2), shown for another 1000 ms (see Fig. 1 for examples of stimuli and conditions). The four conditions were:

a) The target condition “OX” involved the presentation of the letter O (or the corresponding face) as S1, followed by X (or its assigned face for social CPT) as S2. Here, participants were explicitly instructed to press the space bar as quickly as possible with both index fingers after the onset of S2, which is termed the OX throughout this manuscript. This was the only condition in which participants had to press a key.

b) “O.NotX” featured the presentation of the letter O (or the corresponding face) as S1, followed by a stimulus other than X as S2. Note that this represents a prototypical NoGo condition in which a preceding cue is suggested to prepare a response, which is then to be withheld.

c)"NotO.X"encompassed any letter or face as S1 followed by the letter X (or the corresponding face) as S2.

d)"NotO.NotX"involved any letter (or face), excluding O (or the corresponding face) as S1, followed by any letter (or face) other than X (or the corresponding face) as S2. Note that c) and d) represent the NoGo conditions in which the cue already indicated that no response would be required.

The experiment was carried out in a sound-attenuated chamber with low illumination. The experimenter was situated in the adjacent room and monitored the participant via a camera throughout the experiment. A practice block preceded each of the two CPT sessions, which began only once the participants confirmed they had understood the task. Face stimuli (all emotionally neutral and female) consisted of a subset of the Glasgow Unfamiliar Face Database (Burton et al., 2010) and the Facial Recognition Technology (FERET) database (Phillips et al., 1998), which were rated high in natural distinctiveness in a study by Schulz and colleagues (Schulz et al., 2012). The experimental session of this study lasted approximately 13 min (plus individual self-paced breaks every 80 trials).

### Data Processing and EEG Analysis

Electroencephalographic (EEG) data were recorded using a 64-channel BioSemi™ (BioSemi, Amsterdam, Netherlands) Active Two system using sintered Ag/AgCI electrodes affixed to an elastic electrode cap. The electrode arrangement ensured comprehensive coverage by the extended international 10–20 system, covering positions FP1, FT9, AF3, F1, F3, F5, F7, FT7, TP9, FC3, FC1, C1, C3, C5, T7, TP7, PO9, CP3, CP1, P1, P3, O9, P7, P9, PO7, PO3, O1, Iz, Oz, POz, Pz, CPz, FPz, FP2, FT10, AF4, AFz, Fz, F2, F4, F6, F8, FT8, TP10, FC4, FC2, FCz, Cz, C2, C4, C6, T8, TP8, PO10, CP4, CP2, P2, P4, O10, P8, P10, PO8, PO4, and O2.

Note that BioSemi systems work with a ‘‘zero-Ref’’ setup with ground and reference electrodes replaced by a so-called CMS/DRL circuit (for further information, refer to http://www.biosemi.com/faq/cms&drl.htm). Horizontal electrooculogram (EOG) electrodes were positioned at the outer canthi of both eyes, while vertical EOG was recorded from two electrodes placed above and below the right eye. Data were continuously amplified and recorded using a BioSemi™ amplifier and ActiView™ (version 6.0.5) software, incorporating online filtering (DC to 120 Hz, low-pass) and a sampling rate of 512 Hz.

Post-acquisition, ocular artifacts were automatically rectified using BESA™ 7.1 (Berg & Scherg, 1994; Picton et al., 2000). Any trials featuring residual artifacts, such as drifts, were manually excluded by visual inspection. For standard ERPs, epochs were established offline from − 200 to 1200 ms relative to S2 onset (either for letters or faces). Only for analysis of the contingent negative variation (CNV) additional epochs were cut from − 2200 to 0 ms relative to S2 onset. The baseline interval for standard ERPs stemmed from − 200 to 0 ms relative to S2 onset and from − 200 to 0 ms relative to S1 (cue stimulus) onset for the CNV interval. All ERPs were averaged separately for each experimental condition (digitally filtered low pass 30 Hz 24 db/oct and high pass 0.30 Hz 12 db/oct, both zero phase shift) and recalculated to average reference, excluding the vertical EOG. Only trials with correct responses (i.e., correct space press for condition “OX” and no space presses for all other conditions) were subjected to statistical analysis. Trials contaminated by non-ocular artifacts were excluded using the BESA™ artifact rejection tool (amplitude threshold 100 μV, gradient criterion 75 μV, and 0.01 µV criterion for low signal). A criterion of a minimum of 15 accepted trials per condition following artifact rejection was established as a prerequisite for the participant’s inclusion in the data analysis. Two individuals with autism and one typically developed individual were excluded from the ERP analysis because they did not meet this criterion. For the remaining participants with autism, the average numbers of correct and artifact-free trials per condition were 34.0, 37.1, 37.1, and 37.4 for OX, NotO.X, O.NotX, and NotO.X for the letter CPT, respectively. In the face CPT, the trial numbers were 34.9, 36.6, 38.2, and 37.9 (OX, NotO.X, O.NotX, and NotO.X). For typically developed participants the average numbers of correct and artifact-free trials per condition were 37.7, 37.1, 37.3, and 38.6 for OX, NotO.X, O.NotX, and NotO.X for the letter CPT, respectively. In the face CPT, the trial numbers were 37.4, 38.1, 38.4, and 37.7 (OX, NotO.X, O.NotX, and NotO.X).

ERPs were quantified using mean amplitudes for occipital P100 (100–140 ms), occipitotemporal N170 (140–190 ms), P200 (190–240 ms), N250 (260–340 ms), P300 (300–400 ms for letter CPT; 300–500 ms for face CPT), all relative to S2 onset, and for the CNV components (1700–2000 ms, relative to S1 onset). Time intervals were chosen based on distinct peaks identified in the grand mean averages across all conditions and based on visual inspection of the grand means and previous research, (e.g.,Kaufmann et al., 2009; Schweinberger et al., 2002; Tye et al., 2014). Note that we prioritized using identical time segments for early (< 300 ms) ERP components across stimulus types and groups for comparability, tolerating minor differences in peak latencies, which meant that time segments were not exactly symmetrical around every peak. Electrodes of interest were selected based on the maxima of a particular component in grand means and previous research. Accordingly, P100 was analyzed at O1/O2; N170 was quantified at P7/P8, P9/P10, PO7/PO8, and PO9/PO10; P200 was measured at P7/P8 and PO7/PO8; N250 was investigated at TP9/TP10, P7/P8, P9/P10, PO7/PO8, and PO9/PO10; P300 was explored at C3/Cz/C4 and P3/Pz/P4; and finally, CNV was quantified at Cz.

## Results

Where applicable, Epsilon corrections for heterogeneity of covariances were executed employing the Huynh–Feldt method (Huynh & Feldt, 1976) throughout. In instances involving three post-hoc comparisons, the *p*-values underwent adjustment using the Holm method (Holm, 1979). All statistical analyses adhered to an alpha level of α = 0.05, and computations were executed using R version 4.2.3 (R Core Team, 2023). The reported intervals surrounding effect sizes signify 95% confidence intervals. For a full statistical report, including the pairwise comparisons and interaction plots, see the supplementary material uploaded at OSF (https://osf.io/38sae/?view_only = 778e9eed8ea14465a92c64559103e45e).

### Behavioural Data

#### Accuracies

A 2 × 4 × 2 analysis of variance (ANOVA) with the between factor group (ASD, TD), and the within factors condition (OX, NotO.X, O.NotX, NotO.NotX), and stimulus type (letters, faces) on proportion correct was conducted, which resulted in a main effect of group, *F*(1,36) = 7.92, *p* = 0.008, *η*_*p*_^*2* =^ 0.18 [0.01, 0.40], showing overall higher accuracies for typically developed individuals, and a main effect of condition, *F*(1,36) = 11.67, *p* = 0.001, *η*_*p*_^*2*=^ 0.24 [0.11, 0.36], which was further qualified by an interaction of group with condition, *F*(3, 108) = 7.22, *p* = 0.009, *η*_*p*_^*2*^ = 0.17 [0.05, 0.28]. A follow-up analysis depicted a significant difference between the two groups only for the OX condition, *t*(36) = 2.78, *p* = 0.009, *d* = 0.93 [1.61, 0.23]. Both groups showed the lowest accuracies for condition OX (*M*_*ASD*_ = 0.86, *SE* = 0.03, *M*_*TD*_ = 0.98, *SE* = 0.03) and second lowest during NotO.X (Both *M*_*ASD*_ and *M*_*TD*_ = 0.99, *SE* = 0.02). No effect of stimulus type was discovered, *F* < 1, *p* = 0.720. Moreover, lower accuracy in the OX condition of the ASD group was seen both for letters (*M*_*ASD*_ = 0.83, *M*_*TD*_ = 0.99) and faces (*M*_*ASD*_ = 0.88, *M*_*TD*_ = 0.97).

#### Response Times (RTs)

Given that responses were only available for the OX condition, RTs were exclusively analyzed for this condition. A 2 × 2 ANOVA was conducted on correct responses, incorporating group as between-factor and stimulus type as within-factor. The results unveiled a significant main effect of stimulus type, *F*(1,36) = 138.11, *p* < 0.001, *η*_*p*_^*2* =^ 0.79 [0.66, 0.86], which showed markedly shorter RTs for letter stimuli in comparison to face stimuli across both groups (*M*_Faces=_ 547 ms, *SE* = 14, *M*_Letters=_ 455 ms, *SE* = 14). A trend with medium effect size was found for the group effect, *F*(1,36) = 3.41, *p* = 0.073, *η*_*p*_^*2*^ = 0.09 [0.00, 0.29] suggesting somewhat faster responses for TD participants, *M*_*ASD*_ = 526 ms, *SE* = 19, *M*_*TD*_ = 475, *SE* = 19).

Note that both accuracy data and RT data typically deviate from normal distribution, and even though ANOVAs are empirically found to be remarkably robust to such violations of the normality assumption (Schmider et al., 2010), the present accuracy data additionally are close to ceiling in some conditions. Accordingly, we additionally performed non-parametric tests (Friedman ANOVAs and Mann–Whitney U tests) for both accuracy and RT data, which confirmed the above pattern of findings (for full detail, please cf. Supplementary Material, Page 6–9).

### Electrophysiological Data

ERP components from 17 ASD and 18 TD individuals underwent analysis via mixed model analysis of variance (ANOVA). For the components P100, N170, P200, and N250, the analyses incorporated the between factor group and the within factors electrode site, hemisphere, and condition. The analyses of the P300 included the between factor group and the within factors anteriority (C3/Cz/C4 vs. P3/Pz/P4) and laterality (C3/P3 vs. Cz/Pz vs. C4/P4). As the main focus of statistical analyses was on the effects of the between-subject factor group in combination with effects of experimental conditions, the main effects of the electrode site, anteriority, and laterality will not be reported here for the sake of readability and rigor, unless necessary for component interpretation. Note that the complete statistical reports are accessible on OSF (https://osf.io/38sae/?view_only = 778e9eed8ea14465a92c64559103e45e).

### ERPs in the Letter CPT Experiment

#### Letter P100

**Fig. 2:**
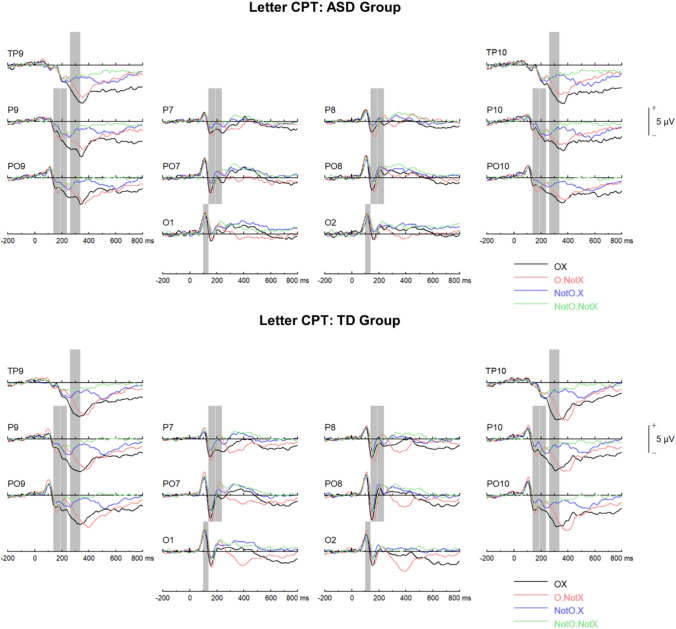
Grand-average ERPs for S2 stimuli in the letter CPT experiment at occipitotemporal electrodes. Analyzed time intervals and corresponding electrode sites for P100, N170, P200, and N250 amplitudes are shaded gray. Top: ASD group. Bottom: Typically developed group

A main effect of condition was observed, *F*(3, 99) = 11.47, *p* < 0.001, *η*_*p*_^*2* =^ 0.26 [0.11, 0.39], indicating attenuated amplitudes for conditions featuring X as S2, with the “OX” condition showing the smallest mean amplitudes. For the complete report on pairwise comparisons, see the supplementary material uploaded at OSF (Fig. 2).

#### Letter N170

A main effect of condition emerged, *F*(3, 99) = 22.90, *p* < 0.001, *η*_*p*_^*2*^ = 0.41 [0.26, 0.52], denoting heightened amplitudes for conditions with O as S1, creating this pattern: OX/O.NotX > NotO.X > NotO.NotX (all *ts* > 2.44, *ps* < 0.041, *|d|*> 0.42). No significant differences were found between OX vs. O.NotX (Fig. 2).

#### Letter P200

A main effect of condition was found, *F*(3, 99) = 11.95, *p* < 0.001, *η*_*p*_^*2* =^ 0.27 [0.12, 0.39], which was further qualified by an interaction with hemisphere, *F*(3, 99) = 6.29, *p* = 0.001, *η*_*p*_^*2* =^ 0.16 [0.04, 0.28]. The follow-up analysis for left hemispheric electrode sites revealed significantly lower amplitudes for condition OX compared to the other three conditions |*ts|*> 5.36, *ps* < 0.001, *|d|*> 0.93. For the right hemisphere, however, no significant differences were observed |ts|< 2.43, ps > 0.12, |d|< 0.42 (Fig. 2).

#### Letter N250

A significant effect of condition *F*(3, 99) = 39.18, *p* < 0.001, *η*_*p*_^*2*^ = 0.54 [0.41, 0.64], was discovered, which presented itself with more negative amplitudes during conditions with the letter O as S1. The main effect was further qualified by a two-way interaction with electrode site, *F*(3, 99) = 21.13, *p* = 0.002, *η*_*p*_^*2*^ = 0.39 [0.31, 0.45]. Further analyses following up on the interaction revealed that at more superior temporal electrode sites (P7/P8 and PO7/PO8), N250 was mainly enhanced for condition O.NotX. By contrast, condition OX had the most enhancement at more inferior temporal electrode sites (P9/P10, PO9/PO10, TP9/TP10) (See Fig. 2).

#### Letter P300

Main effects of condition, *F*(3, 99) = 59.02, *p* < 0.001, *η*_*p*_^*2*^ = 0.64 [0.53, 0.72], and laterality, *F*(2, 66) = 6.66, *p* = 0.002, *η*_*p*_^*2*^ = 0.17 [0.03, 0.32], were detected, along with their interaction, *F*(6, 198) = 14.80, p < 0.001, *η*_*p*_^*2* =^ 0.31 [0.20, 0.40]). There was also an interaction between condition and anteriority, *F*(3, 99) = 52.44, p < 0.001, *η*_*p*_^*2* =^ 0.62 [0.50, 0.70]. Overall, P300 was most prominent for the OX condition and was more pronounced at central compared to right or left lateral sites. Further analyses following up on these interactions revealed no differences between conditions NotO.NotX and NotO.X at right and left lateralities. At central sites, there was no difference between conditions OX and O.NotX, |*ts|*< 0.84, *ps* > 0.40, *|d|*< 0.15. At parietal areas, P3/Pz/P4, however, the highest positivity was seen for condition OX, accounting for the interaction between condition and anteriority. Simple contrasts between conditions at P3/Pz/P4 suggested a 4-fold pattern of graduation (OX > O.NotX > NotO.X > NotO.NotX; all *t*s(33) > 2.49, *ps* < 0.024). In contrast, the numerically highest amplitudes at C3/Cz/C4 were found for condition O.NotX; but the contrast between OX and O.Notx was not significant at C3/Cz/C4 (*M*_*OX* =_ 3.07, *SE* = 0.41 vs *M*_*O.NotX* =_ 3.90, *SE* = 0.42) (Fig. 4). Of note, we observed no significant effects of group (as a main effect or in interaction with other variables; all *p* values > 0.082) in the letter P300.

#### Letter CNV

In order to test whether there was a significant CNV for conditions with O as S1, compared to conditions with another letter as S1, we averaged across two conditions and ran an additional ANOVA with the within-factor condition type (S1 = O vs. S1 ≠ O) and the between-factor group. There was only a significant effect of condition type, *F*(1, 33) = 151.94, *p* < 0.001, *η*_*p*_^*2*^ = 0.82 [0.70, 0.88], with *M*_*First-O*_ = − 1.60, *SE* = 0.20 vs. *M*_*First-NotO*_ = 0.30, *SD* = 0.12 (Fig. 5). There was no main effect of group, and no interaction of condition by group, *F*s(1, 33) < 0.83, *p*s > 0.369, *η*_*p*_^*2*^ < 0.025. This demonstrates that there was a CNV component for letters which was not modulated by group.

### ERPs in the Face CPT Experiment

#### Face P100

**Fig. 3:**
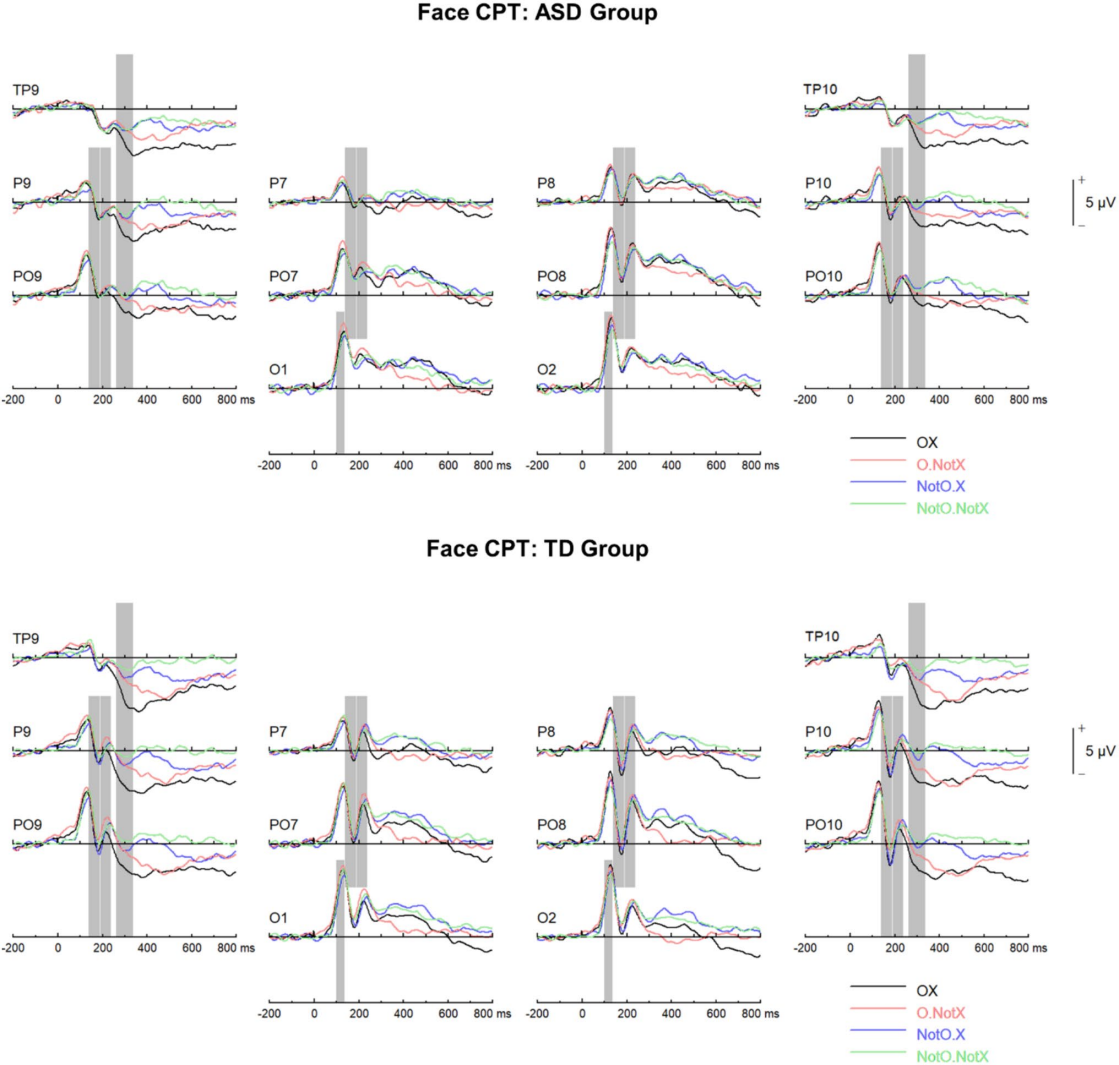
Grand-average ERPs for the Face CPT experiment at occipitotemporal electrodes. Analyzed time intervals and corresponding electrode sites for P100, N170, P200, and N250 amplitudes are grayed out. Top: ASD group. Bottom: Typically developed group

A main effect of condition emerged, *F*(1, 33) = 18.72, *p* < 0.001, *η*_*p*_^*2*^ = 0.36 [0.20, 0.48], denoting heightened amplitudes for conditions with O as S1, with condition O.NotX showing the highest amplitude numerically. This main effect was qualified by a two-way interaction between condition and hemisphere, *F*(3, 99) = 5.50, *p* = 0.002, *η*_*p*_^*2*^ = 0.14 [0.03, 0.26]. All pairwise comparisons for the right hemisphere but OX vs. O.NotX, and between NotO.NotX and NotO.X were significant, *ts* > 3.87, *ps* < 0.002, *ds* > 0.67. For the left hemisphere, all contrasts apart from the one between OX and NotO.NotX were significant (Fig. 3).

#### Face N170

A significant effect of condition, *F*(3, 99) = 6.93, *p* < 0.001, *η*_*p*_^*2*^ = 0.18 [0.05, 0.30], presenting higher negativity for the conditions OX numerically, which was further qualified by an interaction with electrode site, *F*(3, 99) = 3.33, *p* = 0.006, *η*_*p*_^*2*^ = 0.09 [0.02, 0.14]. Further analysis revealed a pattern of N170 enhancement, OX > NotO.X > O.NotX > NotO.NotX at P9/P10 and PO9/PO10 versus a pattern of OX > O.NotX > NotO.X > NotO.NotX at P7/P8 and PO7/PO8. Furthermore, an interaction of group with electrode site was significant, *F*(3, 99) = 4.13, *p* = 0.014, *η*_*p*_^*2*^ = 0.11 [0.01, 0.22]. However, the follow-up analyses for every electrode site separately showed no differences between the ASD and TD groups (*ts*(33) < 1.40, *ps* > 0.17) (Fig. 3). When tested separately per group, then comparing electrode sites showed a significant difference between P9/P10 and P7/P8 electrode sites in ASD group but not in TD, *t*(33) = 2.49, *p* = 0.032, *d* = 0.87 [0.15, 1.58]; the difference between PO9/PO10 and PO7/PO8 was also significant in ASD but not in TD, *t*(33) = 4.94, *p* < 0.001, *d* = 1.72 [0.91, 2.51]. In short, this reflects a difference in component topography, with a tendency towards a more inferior maximum of the N170 for participants with ASD.

#### Face P200

As for the earlier components, a significant effect of condition was discovered, *F*(3, 99) = 6.09, *p* = 0.002, *η*_*p*_^*2*^ = 0.16 [0.03, 0.28], which was further qualified by an interaction with electrode site, F(3, 99) = 9.59, *p* < 0.001, *η*_*p*_^*2*^ = 0.22 [0.08, 0.35], and a three-way interaction with electrode site and hemisphere *F*(3, 99) = 2.90, *p* = 0.039, *η*_*p*_^*2*^ = 0.08 [0.00, 0.18]. Separate ANOVAs for both hemispheres were performed to follow up on the three-way interaction. For the right hemisphere, the ANOVA yielded a main effect of electrode site, *F*(1, 34) = 79.63, *p* < 0.001, *η*_*p*_^*2*^ = 0.70 [0.52, 0.80], with P200 being primarily enhanced at PO7/PO8 in comparison to P7/P8. For the left hemisphere, the ANOVA revealed a main effect of condition *F*(3, 102) = 7.46, *p* < 0.001, *η*_*p*_^*2*^ = 0.18 [0.05, 0.30], a main effect of electrode site *F*(1, 34) = 78.25, *p* < 0.001, *η*_*p*_^*2*^ = 0.70 [0.51, 0.80], and an interaction between the two *F*(3, 102) = 9.86, *p* < 0.001, *η*_*p*_^*2*^ = 0.23 [0.09, 0.35]. Further t-tests only demonstrated a significant difference between OX and O.NotX at P7/P8 electrode sites, *t*(34) = 3.17, *p* = 0.019, *d* = 0.54 [0.18, 0.90]. Conversely, O.NotX had the highest amplitude at PO7/PO8 compared to all the other three conditions (*ts*(34) > 4.41, *ps* < 0.001, *|ds|*> 0.11). An interaction between the factors group and hemisphere was also discovered, *F*(1, 33) = 4.55, *p* = 0.040, *η*_*p*_^*2*^ = 0.12 [0.00, 0.34]. Further analysis depicted no significant difference between ASD and TD groups for either hemisphere (*ts*(33) < 1.68, *ps* > 0.1, *|ds|*< 0.59). Interestingly, separate analyses per group comparing the hemispheres revealed significant asymmetry between left and right hemispheres in the ASD Group *t*(33) = 2.82, *p* = 0.008, *d* = 0.98 [0.25, 1.70] with higher amplitudes over the right hemisphere. However, no hemispheric asymmetry was observed in the TD individuals (See Table 2 and Fig. 3).

**Table 2:** Average amplitude values (in microvolts) of the P200 component during the face CPT experiment

ASD				TD			
	RH		LH		RH		LH
P7	2.27 (0.74)	P8	0.31 (0.48)	P7	1.46 (0.72)	P8	1.74 (0.46)
PO7	4.59 (0.82)	PO8	2.75 (0.62)	PO7	3.84 (0.80)	PO7	3.77 (0.60)
*Mean (SEM)*	3.43 (0.76)		1.53 (0.52)		2.65 (0.74)		2.75 (0.51)

*ASD* Autism spectrum disorder, *TD* Typically developed, *RH* Right hemisphere, *LH* Left hemisphere, *SEM* Standard error of the mean. The larger P200 amplitude over the right hemisphere, compared to the left hemisphere, is seen for participants with autism, but not for typically developed participants

#### Face N250

There was a main effect of condition, *F*(3, 99) = 22.40, *p* < 0.001, *η*_*p*_^*2*^ = 0.41 [0.25, 0.52], as well as an effect of hemisphere, *F*(1, 33) = 4.86, *p* = 0.035, *η*_*p*_^*2*^ = 0.12 [0.00, 0.35], accompanied by a two-way interaction between both factors, *F*(3, 99) = 3.32, *p* = 0.033, *η*_*p*_^*2*^ = 0.09 [0.00, 0.20]. Further analysis for exploring this interaction discovered a four-fold pattern of gradation of N250 enhancement in conditions OX > O.NotX > NotO.X > NotO.NotX, and larger amplitudes for the left hemisphere. A two-way interaction was found between the condition and electrode site, *F*(12, 396) = 10.38, *p* < 0.001, *η*_*p*_^*2*^ = 41 [0.25, 0.52], for which t-tests were performed at each electrode site. These confirmed larger amplitudes for the OX condition (compared to O.NotX). at lateral and temporal sites (P9/P10, PO9/PO10, TP9/TP10), whereas, at P7/P8, PO7/PO8, the differences between OX and O.NotX are less prominent. A trend for a three-way interaction between group, hemisphere, and electrode site was discovered, *F*(4, 132) = 2.11, *p* = 0.084*, η*_*p*_^*2*^ = 0.06 [0.00, 0.13], which potentially reflects less negative amplitudes for ASD participants at right hemispheric electrodes P8 and PO8 (Fig. 3) was not further investigated because it failed to reach conventional levels of statistical significance.

#### Face P300

**Fig. 4:**
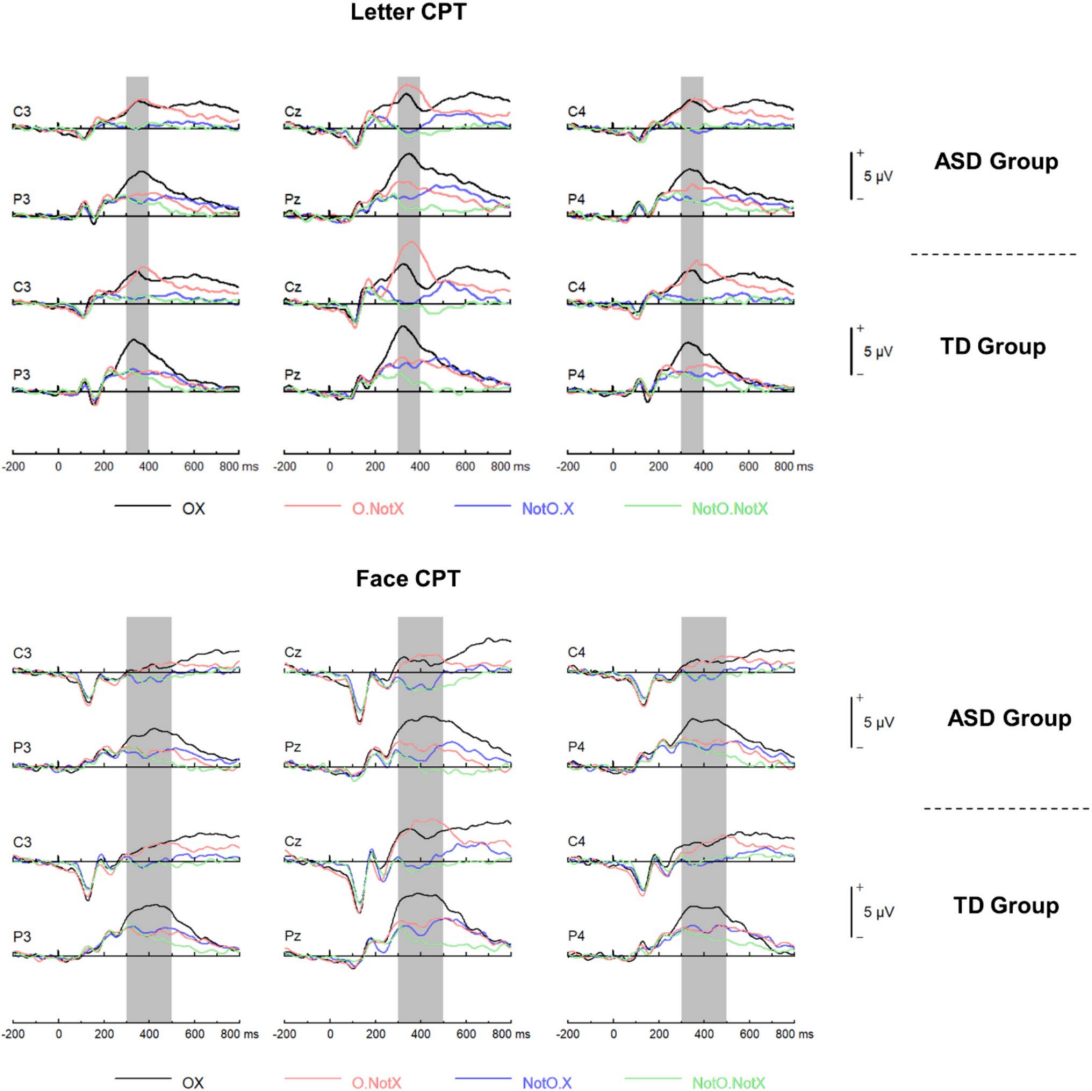
Grand-average ERPs at centroparietal electrodes. Analyzed time intervals for P300 amplitudes are highlighted in gray shading. Top panel: Letter CPT. Bottom Panel: Face CPT. Within each panel, Top row: ASD group. Bottom row: Typically developed group. Note that different time intervals were utilized for the Letter CPT compared to the Face CPT

The earliest prominent effects of group were observed in the P300 during the face CPT, *F*(1, 33) = 6.73, *p* = 0.014, *η*_*p*_^*2*^ = 0.17 [0.01, 0.39], with higher amplitudes for the typically developed group. A main effect of laterality, *F*(2, 66) = 3.43, *p* = 0.014, *η*_*p*_^*2*^ = 0.09 [0.00, 0.23], showed higher amplitudes over central sites. These main effects were further qualified by an interaction, *F*(2, 66) = 4.01, *p* = 0.023, *η*_*p*_^*2* =^ 0.11 [0.00, 0.25], and a three-way interaction between group, laterality and anteriority, *F*(2, 66) = 3.31, *p* = 0.043, *η*_*p*_^*2*^ = 0.09 [0.00, 0.23]. This interaction was followed up with separate ANOVAs for both anteriorities. The analysis for C3/Cz/C4 yielded an interaction between group and laterality, *F*(2, 66) = 3.73, *p* = 0.029, *η*_*p*_^*2*^ = 0.10 [0.00, 0.24], along with a main effect of laterality, *F*(2, 66) = 3.38, *p* = 0.040, *η*_*p*_^*2*^ = 0.10 [0.00, 0.24]. Follow-up analyses showed significantly enhanced P300 for TD only for central electrode sites *t*(33) = 2.32, *p* = 0.027 *d* = 0.17 [− 0.52, 0.85]. At the more parietal electrode sites P3/Pz/P4, a main effect of group, *F*(2, 66) = 4.76, *p* = 0.036, *η*_*p*_^*2*^ = 0.13 [0.00, 0.35], and an interaction of group with laterality, *F*(2, 66) = 4.07, *p* = 0.022, *η*_*p*_^*2*^ = 0.11 [0.00, 0.25], were found, showing an effect of group (TD > ASD), for the left hemisphere, *t*(33) = 2.74, *p* = 0.010, *d* = 0.95 [0.23, 1.67], and for central sites, *t*(33) = 2.55, *p* = 0.015 *d* = 0.89 [0.17, 1.60], but not for right hemispheric sites (Fig. 4). Overall, and in line with our hypothesis, we observed larger P300 responses for TD than ASD participants in the face (but not letter) CPT.

As for the other ERP components, a significant main effect of condition emerged, *F*(3, 99) = 46.61, *p* < 0.001, *η*_*p*_^*2*^ = 0.59 [0.46, 0.67], showing a four-fold pattern of gradation of P300 amplitude (OX > O.NotX > NotO.X > NotO.NotX), which was further qualified by three interactions: a two-way interaction with anteriority, *F*(3, 99) = 31.83, *p* < 0.001, *η*_*p*_^*2*^ = 0.59 [0.46, 0.67], a two-way interaction with laterality, *F*(6, 198) = 22.39, *p* < 0.001, *η*_*p*_^*2*^ = 0.41 [0.29, 0.49], and finally a three-way interaction with anteriority and laterality, *F*(6, 198) = 7.97, *p* < 0.001, *η*_*p*_^*2*^ = 0.20 [0.09, 0.28]. Separate ANOVAs were performed for the two different anteriorities to unfold this three-way interaction. At C3/Cz/C4, the ANOVA yielded a main effect of condition, *F*(3, 102) = 32.73, *p* < 0.001, *η*_*p*_^*2*^ = 0.20 [0.35, 0.59], and an interaction of condition with laterality, *F*(6, 204) = 22.12, *p* < 0.001, *η*_*p*_^*2*^ = 0.39 [0.28, 0.47]. The follow-up analysis investigating the interaction revealed no significant difference between the conditions OX vs. O.Notx, as well as between the conditions NotO.NotX vs. NotO.X on any of the right, central, or left sides. In other words, the amplitudes for the conditions OX and O.NotX were similarly high and the highest over central sites. By contrast, at the C3/Cz/C4 anteriority, P300 amplitudes for conditions NotO.X and NotO.NotX were similarly low and lowest at the central sites. At P3/Pz/P4, the follow-up ANOVA resulted in a main effect of condition, *F*(3, 102) = 58.69, *p* < 0.001, *η*_*p*_^*2*^ = 0.63 [0.52, 0.71], and an interaction between condition and laterality, *F*(6, 204) = 15.78, *p* < 0.001, *η*_*p*_^*2*^ = 0.32 [0.20, 0.40]. Analyses following the latter interaction showed that the OX condition evoked significantly higher amplitudes than the three other conditions, specifically over central sites (See Table 3 and Fig. 4).

**Table 3:** Average amplitude values (in microvolts) of the P300 component during the face CPT experiment

ASD				TD			
	RH	Mid	LH		RH	Mid	LH
Central	0.33 (0.32)	− 0.03 (0.47)	− 0.07 (0.28)	Central	0.92 (0.31)	1.48 (0.45)	0.60 (0.28)
Parietal	2.78 (0.36)	2.04 (0.40)	1.87 (0.28)	Parietal	3.06 (0.35)	3.47 (0.39)	2.96 (0.28)
*Mean (SEM)*	1.56 (0.28)	1.00 (0.38)	0.90 (0.22)		1.99 (0.27)	2.47 (0.36)	1.78 (0.21)

*ASD*, Autism spectrum disorder, *TD* Typically developed, *RH* Right hemisphere, *Mid* Midline, *LH* Left hemisphere, *SEM* Standard error of the mean. At central sites, note the larger P300 amplitude for TD than ASD participants which was significant at the midline electrode only; for parietal sites, larger amplitudes for TD participants were seen at midline and left-hemispheric electrodes

#### Face CNV

**Fig. 5:**
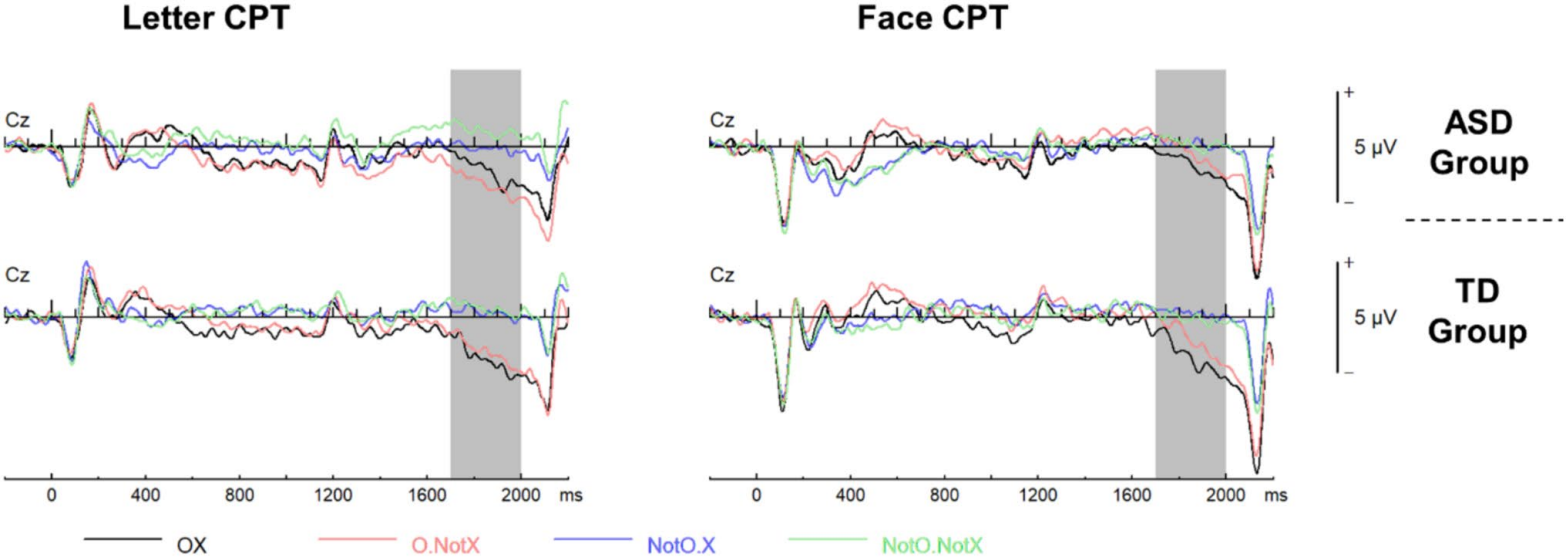
Grand-average ERPs at electrode Cz; analyzed time intervals for CNV amplitudes are shaded in gray. Note that the ERPs to the cued condition (in which S1 is the cue “O”; red and black lines) exhibit a prominent CNV preceding S2 onset at 2000 ms, relative to the uncued condition (in which S1 is not the cue “O”). These same cued conditions also exhibit a discernible P300 to S1. Note also larger CNV amplitudes for TD than for ASD participants in the face CPT only (also cf. main text for details)

Similar to the letter CPT, an ANOVA was conducted with the within-factor condition type (S1 = face O, vs, S1 ≠ face O) and the between-factor group. The results unveiled a main effect of group, *F*(1, 33) = 6.95, *p* = 0.013, *η*_*p*_^*2*^ = 0.17 [0.01, 0.40], denoting enhanced (i.e., more negative) CNV in TD participants, *M*_*ASD*_ = − 0.22, *SE* = 0.12, *M*_*TD*_ = − 0.68, *SE* = 0.12. A main effect of condition type emerged as well, *F*(1, 33) = 32.69, *p* < 0.001, *η*_*p*_^*2*^ = 0.50 [0.25, 0.67], revealing an enhanced CNV for the conditions with O as their first stimulus (OX and, O.NotX) versus conditions that start with stimulus other than O (NotO.X and NotO.NotX), with M_First-O_ = − 1.00, SE = 0.14 vs. M_First-NotO_ = 0.10, SE = 0.12 (Fig. 5).

**Fig. 6:**
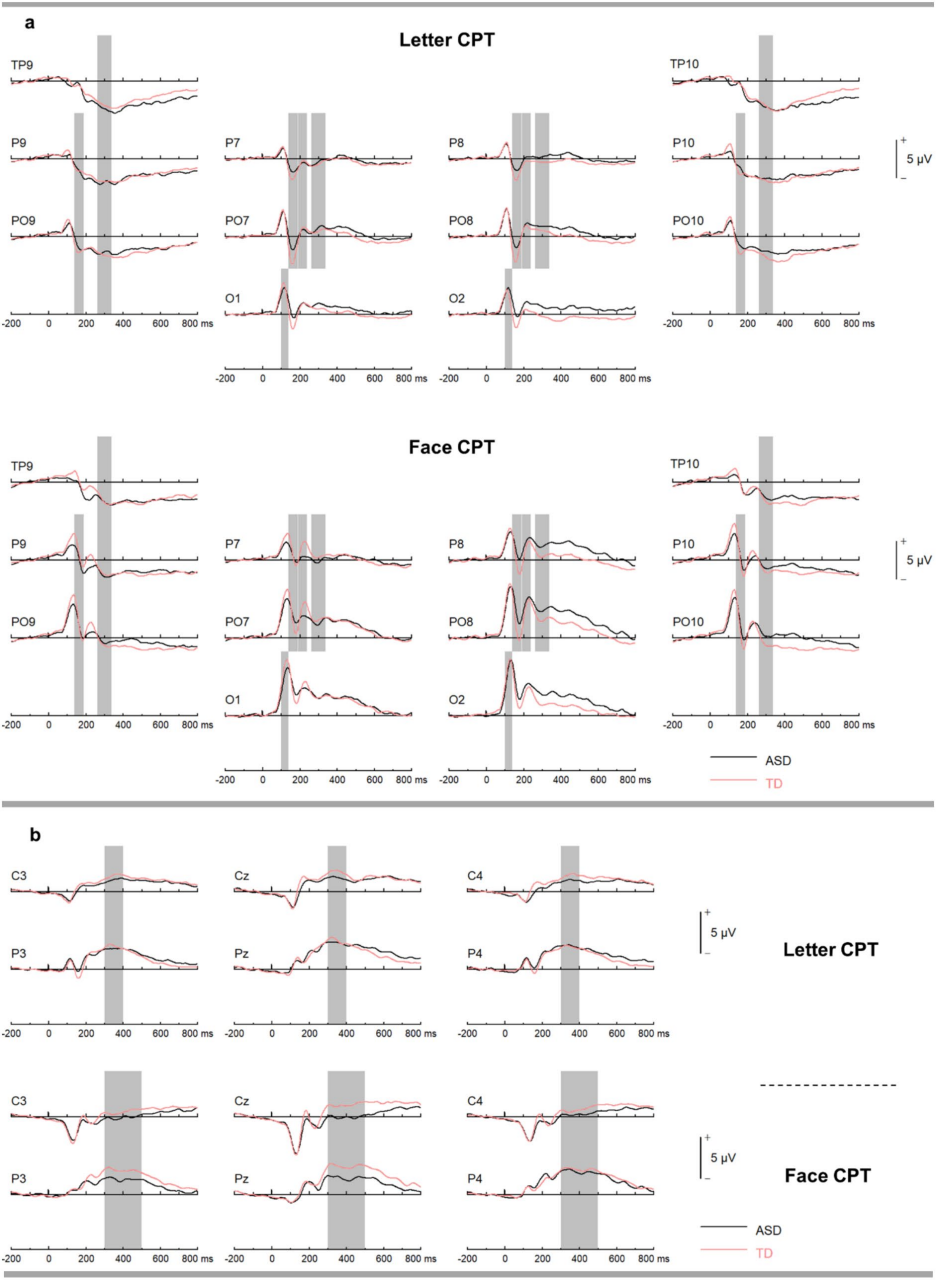

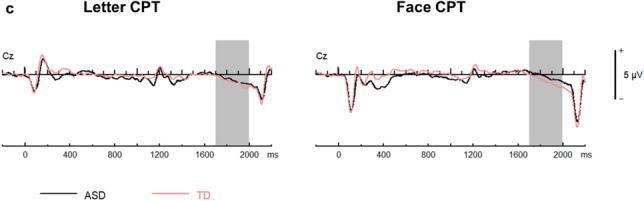
Grand mean ERPs averaged across the four experimental conditions within the letter (left) and the face CPT (right) for both groups, Panel (**a**) depicts P100, N170, P200, and N250 in letter and face CPTs. Panel (**b**) demonstrates the P300 components during both CPT experiments, and panel (**c**) portrays the CNV components. Note that for each panel, analyzed time intervals for ERP components are indicated by gray shading

Finally, although the interaction between group and condition only approached significance, *F*(1, 33) = 3.69, *p* = 0.063, *η*_*p*_^*2*^ = 0.10, Fig. 6 indicates that the group difference is particularly clear in the cued condition in which S1 = face O, and in which there is a substantial CNV present. Overall, and in line with our hypothesis, this study provided initial evidence for a larger CNV in TD vs ASD participants in the context of a face (but not letter) CPT task.

## Discussion

Using two types of equivalent CPT paradigms, we investigated attention toward social (faces) and non-social stimuli (letters) in young adults diagnosed with high functional autism compared to a group of typically developed individuals. Our behavioral results highlight that regardless of the group, responding to face stimuli took longer than responding to letters. Furthermore, regardless of stimulus type, TDs performed with higher accuracy to the OX conditions. For the other conditions, no group differences emerged, thus suggesting no evidence for an impairment in response inhibition in the autistic group in the behavioral data. For ERPs, evidence for group differences was found specifically for the face CPT, showing up in the P300 and CNV components.

The CNV amplitude reflects neuronal excitability in preparation for an upcoming internal or external stimulus, meaning that it indicates neural resources for both anticipatory attention and motoric response preparation (Brunia et al., 2011; Dhar et al., 2010; Hoyland et al., 2017; Tye et al., 2014). The primary neural generators of the CNV are thought to be located in the frontal cortex, namely the dorsolateral prefrontal cortex, along with the motor preparatory areas, which play a central role in exerting top-down response preparation and TD. The CNV has been investigated mainly within the interpretative framework of executive functions, representing higher-order cognitive processes that support goal-oriented behaviors, such as inhibitory control, problem-solving, and cognitive flexibility (Brunia et al., 2011; Prillinger et al., 2022). CNV has been used in neuropsychiatric disorders as a marker of executive functions and has been identified to be attenuated in several of them, e.g., in schizophrenia and bipolar disorder (Li et al., 2015), functional movement disorders (Teodoro et al., 2020), and even seizures (Drake et al., 1997; Vein et al., 1998). In ADHD individuals, enhanced CNV has been established to be an outcome measure of neurofeedback intervention (Mayer et al., 2016). Regarding ASD individuals, however, there are mixed findings. Hoyland observed enhanced CNV for both ASD and ASD with ADHD groups compared to neurotypicals, and Tye found enhanced CNV only in the ASD group compared to ADHD and TD groups. They both related their observation to lower flexibility in ASD individuals (Hoyland et al., 2017; Tye et al., 2014). By contrast, Hoofs et al. reported attenuated CNV in the ASD group compared to the TD group (Hoofs et al., 2018). Later on, Prillinger et al. used CNV as a marker of responding to the neurofeedback intervention in ASD individuals; attenuated CNV before neurofeedback was a dependent factor for increased CNV after NFT (Prillinger et al., 2022). Our results showed no difference between ASD and TD groups during letter CPT. However, interestingly, and in contrast to Tye et al. (2014) and Hoyland et al. (2017), we saw decreased CNV in ASD individuals during face CPT, suggestive of less response preparation and attention toward facial stimuli compared to TDs.

The P300 reflects information processing chains tied to attention-driven and memory processes. In the context of the CPT task, the P300 can index the amount of attentional resources allocated to a task (Cui et al., 2017; Polich, 2011). Several factors are known to influence the P300, including task difficulty, which decreases this component’s amplitude (Cui et al., 2017), or the target-to-target interval as the primary determinant of P300 amplitude. P300 amplitude recedes when two targets appear consecutively (Polich, 2011). Cui et al. (2017) found lower P3b amplitude, a subcomponent of P300, in ASD individuals compared to TDs but no group differences in P3a amplitude, another subcomponent of P300 with a more frontal maximum. The authors suggested that people with ASD might add self-inflicted difficulty to the task by often concentrating on tiny details instead of the entire context. In a recent meta-analysis, Farashi et al. investigated ERP components in ASD and TD individuals and found numerically (but insignificantly) smaller P300 amplitudes at posterior sites in ASD compared to TD group. The authors, however, related their insignificant finding to the variety of tasks and stimulus types they had included in their study (Farashi et al., 2023). Our results demonstrated lower P300 amplitudes evoked in the face CPT for ASD compared to TD individuals, a finding which is relevant in view of the fact that no such group difference was found for the letter CPT task. This pattern indicates reduced attentional resources toward the socially relevant face stimuli in ASD individuals. As such, the finding of a specific alteration of social attention to faces in ASD addresses one of the key questions posed by an influential review on face identity recognition in autism and has important implications for any strategy to improve face processing (cf. Weigelt et al., 2012).

As mentioned earlier, task difficulty can affect P300 amplitudes. However, considering the overall high accuracies for both CPT types, it seems that neither of the tasks was very challenging for our participants, such that task difficulty per se may be seen as having had little relevance in the present study. Alternatively, smaller amplitudes for the face CPT may suggest restricted information processing with fewer resources devoted to context updating of faces compared to letters. This would align with studies indicating lower involvement of neural resources in dorsal and ventral networks during face CPT in ASD participants (Cui et al., 2017; Farashi et al., 2023; Polich, 2011). Hoyland et al. and Thillay et al. did not discover differences in P300 amplitudes between ASD and TD groups, even during an emotional CPT using human facial emotions (Hoyland et al., 2017; Thillay et al., 2016). One reason might be that they used the same stimulus as both cue and target (e.g., an angry face), which decreases the target-to-target interval and causes smaller P300 amplitudes. However, our ERP results do not explain the poorer behavioral ASD group performance during the OX condition in both CPT types. One illustrative remark might be that most of ERP components linked to the performance including frontal N2 (Hoyland et al., 2017; Riccio et al., 1999; Tye et al., 2014), P300 latency (Cui et al., 2017; Farashi et al., 2023; Riccio et al., 1999), or error related negativity (Hüpen et al., 2016) were not the focus of the current study.

Although only P300 and CNV during face CPT resulted in the main effects of the group, we found group interactions with other factors during the face CPT. This was the case for the earlier components, N170 and P200. Our N170 results did not depict a significant difference in N170 amplitude between autistic and TD groups overall, which seems perfectly in line with a recent meta-analysis including seventeen other relevant studies (Farashi et al., 2023). Our findings only tentatively point to smaller topographical group differences, in that during the face CPT, N170 was prominent over inferior P9/P10 electrode sites in ASD participants, while in TD individuals, there was no significant difference between P9/P10 and P7/P8. Based on findings on the role of N170 in face perception research and on its putative neural sources (Itier & Batty, 2009; Rossion & Jacques, 2011; Schweinberger et al., 2002), while our findings might suggest small group differences in neural sources for face processing in ASD, but we advise that such small differences in N170 topography should be interpreted cautiously at this point.

Similar to the N170 outcomes, the analysis of P200 amplitudes showed a significant interaction involving the factor group during the face CPT. This demonstrated an asymmetry between the right and left hemispheres in the ASD group. The P200 component was primarily larger over the right side (*M*_*Right*_ = 3.43 µV) than the left side (*M*_*Left* =_ 1.53 µV). By contrast, P200 was largely symmetric, and there was no significant difference between the right and left hemispheres in the TD group (*M*_*Right*_ = 2.56 µV, *M*_*Left*_ = 2.75 µV). The posterior P200 has been neglected in the ERP literature to some extent. However, studies on face perception indicate that the P200 amplitude is associated with visual categorization (Farashi et al., 2023) and the perception of face typicality (Kloth et al., 2017; Schweinberger & Neumann, 2016; Wiese et al., 2024). In their meta-analysis, Farashi et al. uncovered that the posterior P200 latency was increased in ASD individuals compared to TDs. Conversely, they found delayed latencies of P200 for both hemispheres and did not report P200 amplitude differences differences between hemispheres in ASD individuals. Overall, however, studies investigating face-elicited P200 in autism are sparse, and more research is required to decipher the role of posterior P200 in autism.

Overall, the target (OX) conditions elicited the largest amplitudes in most ERP components, and particularly so in the P300. In other components, including the P100, N170, and CNV, large amplitudes to (or before) S2 were generally seen when S2 was attentionally cued by S1 = O. Effects of attention on ERPs is well-established, and attention effects in the P100 and N1/N170 may reflect top-down modulation of the flow of sensory activity through extrastriate visual cortex areas (Luck et al., 2000). Estimated source waveforms for the P1 and N1 are larger for attended than unattended stimuli, supporting this interpretation despite challenges associated with ERP source localization (Luck & Kappenman, 2011). It therefore may be surprising that the present P100 exhibited the lowest amplitude in trials where X was the second stimulus. As P100 amplitude is also sensitive to low-level physical characteristics of the stimuli, such as luminance (Johannes et al., 1995) it may be relevant to note that we verified that both types of stimuli had similar luminance (cf. Methods and OSF repository for further details).

Our study is not without limitations. Despite the fact that our sample planning followed a formal power analysis, we had focused on a main effect of group. Accordingly, it remains possible that some more nuanced or small effects of experimental variables (that would exhibit interactive effects depending on group) might have been missed with the present study design. Second, it should be acknowledged that the present research also focuses on highly functioning people with autism and preserved cognition and language, whereas there clearly is a paucity of neuroimaging research with people with a more severe phenotype of autism (e.g., Jack & Pelphrey, 2017). Third, having two CPT tasks in one EEG recording session is time-consuming and potentially demanding, raising the possible concern that participants’ tiredness, in principle, could have affected the present results – especially if one assumes differential effects of study demand for TD and ASD participants. However, we believe it is unlikely that these factors substantially affected the present results, especially when considering overall high behavioral accuracies across groups, with no differential differences depending on stimulus type and only marginally slower response times for ASD vs. TD participants during task performance. A fourth limitation could be seen in the fact that we focused our data analysis on ERP amplitudes but did not explicitly analyze ERP onset or peak latencies (in the interest of paper length, and because we did not have specific hypotheses regarding latencies), nor did we perform time–frequency analyses of the present data. Fifth, we generally acknowledge that the conclusions from this paper regarding social attention are constrained by the choice of the CPT paradigm, and we believe this paper can inform future investigations into different paradigms and tasks that focus on different aspects of attention and their ERP correlates, including frontal N2, Cue-P3, or error-related negativity (e.g., Hoyland et al., 2017; Tye et al., 2014).

In essence, the current study explored attentional processes toward social and non-social stimuli (faces and letters) in otherwise equivalent CPT experiments. Our observations revealed differences between autistic and neurotypical adults only for the face CPT, notably in terms of smaller amplitudes of both the P300 and CNV ERP components. Our results point to deviant information processing regarding both social attention and response preparation toward facial stimuli in individuals with autism, offering further insights into the cognitive profile of autism.

## Supplementary Information

Below is the link to the electronic supplementary material.

Supplementary file1 (PDF 6366 KB)
