# Supplementary material for: Attention to Social and Non-Social Stimuli in a Continuous Performance Test in Autistic and Typically Developed Participants: An ERP Study
Source: J Autism Dev Disord. 2025 Apr 16;56(9):3438–55. doi: 10.1007/s10803-025-06825-9 (PMC13427866; doi:10.1007/s10803-025-06825-9)

Attention to social and non-social stimuli in a continuous performance test in autistic and neurotypical participants

General approach and participants

- **Participants:** 20 individuals with high-functional ASD (diagnosed following current standards and using the ADOS-2) and 20 typically developing adolescents, matched in intelligence (IQ), age, and gender, planned to be included.
- For the behavioral analysis, 19 were in each group; for the ERP analysis, 17 ASDs and 18 NTs were finally included.

*** 2 ASD and 1 NT individuals were excluded due to lack of sufficient trials (threshold=15)**

- **The diagnostic session involved a battery of questionnaires, including:** the Autism Spectrum Quotient (AQ), World Health Organization Quality of Life Questionnaire Short Form (WHOQoL-BREF), Multidimensional Assessment of Interoceptive Awareness Questionnaire (MAIA), Camouflaging Autistic Traits Questionnaire (CAT-Q), Saarbrücker questionnaire (SPF), and Toronto Alexithymia Scale (TAS-26).

EEG Experiments:

1) **Letter Continuous Performance Test: Non-social stimuli**
2) **Face Continuous Performance Test: social stimuli**

Participants: Clinical and demographic characteristics including subscale results.

Variable	ASD M (S.D.)	TD M (S.D.)	Test type	statistic	p_value	Result
Age	23.421 (7.05)	23.000 (5.907)	wilcoxon test	178.000	0.9533	N.S
AQ_Attention Switching	7.632 (2.114)	4.235 (2.223)	t test	4.697	<0.0001	ASD > Control
AQ_ Attention to Detail,	6.579 (1.953)	4.412 (2.123)	t test	3.191	0.0030	ASD > Control
AQ_ Communication	6.368 (1.978)	2.176 (1.38)	t test	7.289	<0.0001	ASD > Control
AQ_Imagination	4.632 (1.571)	2.765 (1.48)	wilcoxon test	256.500	0.0023	ASD > Control
AQ_Social Skills	7.158 (2.672)	1.647 (1.73)	wilcoxon test	306.000	<0.0001	ASD > Control
AQ_Total	32.368 (7.018)	15.235 (4.024)	wilcoxon test	310.000	<0.0001	ASD > Control
Big Five_Openness	22.579 (3.834)	19.824 (5.114)	t test	1.841	0.0743	N.S
Big Five_Conscientiousness	21.105 (3.9)	18.294 (5.108)	t test	1.867	0.0705	N.S
Big Five_Extraversion	15.947 (3.993)	19.353 (4.568)	t test	-2.387	0.0227	ASD > Control

Note. ASD, autism spectrum disorder; TD, typically developing controls; AQ, autism spectrum quotient; WAIS_IQ, Wechsler Adult Intelligence Scale (WAIS) intelligence quotient; CatQ, Camouflaging Autistic Traits Questionnaire; TAS26, Toronto Alexithymia Scale 26; WHO_QoL, WHO Quality of Life; SPF, Saarbrücker personality questionnaire; MAIA, Multidimensional Assessment of Interoceptive Awareness; M, mean; S.D., standard deviation; N.S., non-significant difference.

Participants: Clinical and demographic characteristics including subscale results.

Variable	ASD M (S.D.)	TD M (S.D.)	Test type	statistic	p_value	Result
**Big Five_Agreeableness**	19.368 (3.975)	22.294 (4.538)	t test	-2.062	0.0469	ASD < Control
**Big Five_Neuroticism**	20.421 (4.488)	17.294 (5.241)	t test	1.928	0.0622	N.S
**WAIS_Speech Understanding**	104.632 (16.118)	109.912 (8.997)	t test	-1.230	0.2287	N.S
**WAIS_Logic Thinking**	107.842 (16.067)	104.000 (14.602)	t test	0.748	0.4599	N.S
**WAIS_Working Memory**	97.789 (15.824)	102.824 (12.866)	t test	-1.039	0.3060	N.S
**WAIS_Processing Speed**	93.947 (12.647)	104.412 (19.148)	wilcoxon test	99.000	0.0473	ASD < Control
**WAIS_IQ**	101.368 (13.937)	106.588 (11.662)	t test	-1.211	0.2344	N.S
**CatQ_Compensation**	35.947 (12.699)	20.706 (6.42)	wilcoxon test	276.000	**0.0003**	**ASD > Control**
**CatQ_Masking**	32.579 (10.194)	30.000 (7.794)	t test	0.845	0.4041	N.S
**CatQ_Assimilation**	36.421 (5.699)	22.529 (7.417)	t test	6.339	**<0.0001**	**ASD > Control**

Note. ASD, autism spectrum disorder; TD, typically developing controls; AQ, autism spectrum quotient; WAIS_IQ, Wechsler Adult Intelligence Scale (WAIS) intelligence quotient; CatQ, Camouflaging Autistic Traits Questionnaire; TAS26, Toronto Alexithymia Scale 26; WHO_QoL, WHO Quality of Life; SPF, Saarbrücker personality questionnaire; MAIA, Multidimensional Assessment of Interoceptive Awareness; M, mean; S.D., standard deviation; N.S., non-significant difference.

Participants: Clinical and demographic characteristics including subscale results.

Variable	ASD M (S.D.)	TD M (S.D.)	Test type	statistic	p_value	Result
CatQ_Total	104.947 (21.93)	73.235 (14.272)	t test	5.074	**<0.0001**	**ASD > Control**
TAS26_Identifcation Feelings	20.316 (3.713)	15.176 (7.35)	t test	2.601	**0.0159**	**ASD > Control**
TAS25_Description Feelings	17.526 (3.672)	13.353 (5.465)	t test	2.715	**0.0103**	**ASD > Control**
TAS26_External Oriented Thinking	13.421 (2.714)	14.118 (2.934)	t test	-0.740	0.4644	N.S
TAS26_Total	51.263 (8.082)	42.647 (13.416)	t test	2.301	**0.0298**	**ASD > Control**
WHO_QoL_Overall	3.474 (0.697)	3.941 (0.966)	wilcoxon test	115.500	0.1241	N.S
WHO_Qol_Physical Health	25.000 (3.873)	28.235 (3.817)	t test	-2.519	**0.0166**	**ASD < Control**
WHO_Qol_Psychological Health	19.368 (3.419)	21.176 (4.305)	t test	-1.403	0.1698	N.S
WHO_QoL_Social Relationships	9.474 (2.611)	10.588 (2.717)	t test	-1.254	0.2183	N.S
WHO_QoL_Environment	29.421 (3.421)	32.647 (3.968)	t test	-2.620	**0.0130**	**ASD < Control**

Note. ASD, autism spectrum disorder; TD, typically developing controls; AQ, autism spectrum quotient; WAIS_IQ, Wechsler Adult Intelligence Scale (WAIS) intelligence quotient; CatQ, Camouflaging Autistic Traits Questionnaire; TAS26, Toronto Alexithymia Scale 26; WHO_QoL, WHO Quality of Life; SPF, Saarbrücker personality questionnaire; MAIA, Multidimensional Assessment of Interoceptive Awareness; M, mean; S.D., standard deviation; N.S., non-significant difference.

Participants: Clinical and demographic characteristics including subscale results.

Variable	ASD M (S.D.)	TD M (S.D.)	Test type	statistic	p_value	Result
SPF_FS_Fantasy	13.316 (4.028)	13.059 (3.172)	t test	0.211	0.8343	N.S
SPF_EC_Empathic Concern	13.737 (2.786)	14.765 (1.48)	t test	-1.402	0.1718	N.S
SPF_PT_Perspective Taking	13.947 (3.719)	14.176 (3.026)	t test	-0.201	0.8417	N.S
SPF_PD_Personal Distress	15.000 (2.603)	11.176 (3.468)	t test	3.766	**0.0006**	**ASD > Control**
SPF_Total	41.000 (8.09)	39.353 (10.523)	wilcoxon test	153.500	0.8119	N.S
MAIA_ Noticing	3.158 (0.791)	3.294 (0.494)	t test	-0.611	0.5455	N.S
MAIA_ Non-Distraction	1.474 (0.81)	1.648 (0.768)	t test	-0.659	0.5141	N.S
MAIA_ Not Worrying	1.876 (0.772)	2.471 (0.86)	t test	-2.186	**0.0358**	**ASD < Control**
MAIA_ Attention Regulation	2.564 (0.842)	3.168 (0.945)	t test	-2.030	0.0503	N.S
MAIA_ Emotional Awareness	3.526 (1.033)	3.459 (0.674)	wilcoxon test	185.000	0.4631	N.S

Note. ASD, autism spectrum disorder; TD, typically developing controls; AQ, autism spectrum quotient; WAIS_IQ, Wechsler Adult Intelligence Scale (WAIS) intelligence quotient; CatQ, Camouflaging Autistic Traits Questionnaire; TAS26, Toronto Alexithymia Scale 26; WHO_QoL, WHO Quality of Life; SPF, Saarbrücker personality questionnaire; MAIA, Multidimensional Assessment of Interoceptive Awareness; M, mean; S.D., standard deviation; N.S., non-significant difference.

Participants: Clinical and demographic characteristics including subscale results.

Variable	ASD M (S.D.)	TD M (S.D.)	Test type	statistic	p_value	Result
**MAIA_ Self-Regulation**	2.737 (1.368)	2.603 (1.153)	t test	0.316	0.7543	N.S
**MAIA_Listening to the Body**	2.577 (0.942)	2.235 (1.252)	t test	0.933	0.3576	N.S
**MAIA_ Trust**	2.963 (0.958)	3.725 (0.966)	t test	-2.375	**0.0233**	**ASD < Control**
**MAIA_Total**	20.875 (4.606)	22.603 (4.659)	t test	-1.118	0.2715	N.S

Note. ASD, autism spectrum disorder; TD, typically developing controls; AQ, autism spectrum quotient; WAIS_IQ, Wechsler Adult Intelligence Scale (WAIS) intelligence quotient; CatQ, Camouflaging Autistic Traits Questionnaire; TAS26, Toronto Alexithymia Scale 26; WHO_QoL, WHO Quality of Life; SPF, Saarbrücker personality questionnaire; MAIA, Multidimensional Assessment of Interoceptive Awareness; M, mean; S.D., standard deviation; N.S., non-significant difference.

Participants' comorbidities and psychiatric medications

Group	Participant's number	Comorbidity	Medication
ASD	1	Maybe ADHD	-
ASD	2	Depression	Sertraline (200mg)
ASD	3	Panic disorder, Depression	Fluoxetine (20 mg)
ASD	4	-	Testosterone undecanoate (1000 mg)
ASD	5	Maybe ADHD, diverse PSD	Aripiprazole (10 mg), Fluoxetine (20mg), Candesartan cilexetil (8 mg) for HTN
ASD	6	-	-
ASD	7	-	-
ASD	8	ADHD	-
ASD	9	-	Amisulpride (400 mg), Aripiprazole (15mg), Candesartan cilexetil (8 mg) for HTN
ASD	10	Articulation disorder Musculoskeletal difficulties not otherwise specified	-

Note. Note. ASD, autism spectrum disorder; TD, typically developing controls; ADHD, attention deficit hyperactivity disorder; PSD, Psychosocial disorders; HTN, hypertension; PTSD, post-traumatic stress disorder. Sertraline, Fluoxetine, and Escitalopram are members of selective serotonin reuptake inhibitors (SSRIs), which are mostly prescribed for depression and anxiety disorders. Amisulpride and Aripiprazole belong to the big family of atypical antipsychotics, which effectively block D2 dopamine receptors with little or no impact on D1 receptors.

Participants' comorbidities and psychiatric medications

Group	Participant's number	Comorbidity	Medication
ASD	11	-	-
ASD	12	Depression	-
ASD	13	-	-
ASD	14	Selective mutismus	
ASD	15	Speech disorder, Game addiction	-
ASD	16	Dyscalculia, Depression<br>Panic disorder, Migraine headaches	Escitalopram (15mg)
ASD	17	Depression	Fluoxetine (20mg)
ASD	18	PTSD, Depression	-
ASD	19	-	-
ASD	20	Recurrent depression	-

Note. Note. ASD, autism spectrum disorder; TD, typically developing controls; ADHD, attention deficit hyperactivity disorder; PSD, Psychosocial disorders; HTN, hypertension; PTSD, post-traumatic stress disorder. Sertraline, Fluoxetine, and Escitalopram are members of selective serotonin reuptake inhibitors (SSRIs), which are mostly prescribed for depression and anxiety disorders. Amisulpride and Aripiprazole belong to the big family of atypical antipsychotics, which effectively block D2 dopamine receptors with little or no impact on D1 receptors.

Participants' comorbidities and psychiatric medications

Group	Participant's number	Comorbidity	Medication
**ASD**	21	Burnout, Depression	-
**TD**	101	-	-
**TD**	102	-	-
**TD**	103	-	-
**TD**	104	-	-
**TD**	105	-	-
**TD**	106	-	-
**TD**	107	-	-
**TD**	109	-	-
**TD**	110	Aggression problems in childhood	-

Note. Note. ASD, autism spectrum disorder; TD, typically developing controls; ADHD, attention deficit hyperactivity disorder; PSD, Psychosocial disorders; HTN, hypertension; PTSD, post-traumatic stress disorder. Sertraline, Fluoxetine, and Escitalopram are members of selective serotonin reuptake inhibitors (SSRIs), which are mostly prescribed for depression and anxiety disorders. Amisulpride and Aripiprazole belong to the big family of atypical antipsychotics, which effectively block D2 dopamine receptors with little or no impact on D1 receptors.

Participants' comorbidities and psychiatric medications

Group	Participant's number	Comorbidity	Medication
**ASD**	21	Burnout, Depression	-
**TD**	101	-	-
**TD**	102	-	-
**TD**	103	-	-
**TD**	104	-	-
**TD**	105	-	-
**TD**	106	-	-
**TD**	107	-	-
**TD**	109	-	-
**TD**	110	Aggression problems in childhood	-

Note. Note. ASD, autism spectrum disorder; TD, typically developing controls; ADHD, attention deficit hyperactivity disorder; PSD, Psychosocial disorders; HTN, hypertension; PTSD, post-traumatic stress disorder. Sertraline, Fluoxetine, and Escitalopram are members of selective serotonin reuptake inhibitors (SSRIs), which are mostly prescribed for depression and anxiety disorders. Amisulpride and Aripiprazole belong to the big family of atypical antipsychotics, which effectively block D2 dopamine receptors with little or no impact on D1 receptors.

Participants' comorbidities and psychiatric medications

Group	Participant's number	Comorbidity	Medication
**TD**	111	-	-
**TD**	112	-	-
**TD**	113	-	-
**TD**	114	-	-
**TD**	115	-	-
**TD**	116	Depression	Sertraline (100mg)
**TD**	117	-	-
**TD**	119	-	-
**TD**	120	-	-
**TD**	121	Remitted depression (2015-2017)	-

Note. Note. ASD, autism spectrum disorder; TD, typically developing controls; ADHD, attention deficit hyperactivity disorder; PSD, Psychosocial disorders; HTN, hypertension; PTSD, post-traumatic stress disorder. Sertraline, Fluoxetine, and Escitalopram are members of selective serotonin reuptake inhibitors (SSRIs), which are mostly prescribed for depression and anxiety disorders. Amisulpride and Aripiprazole belong to the big family of atypical antipsychotics, which effectively block D2 dopamine receptors with little or no impact on D1 receptors.

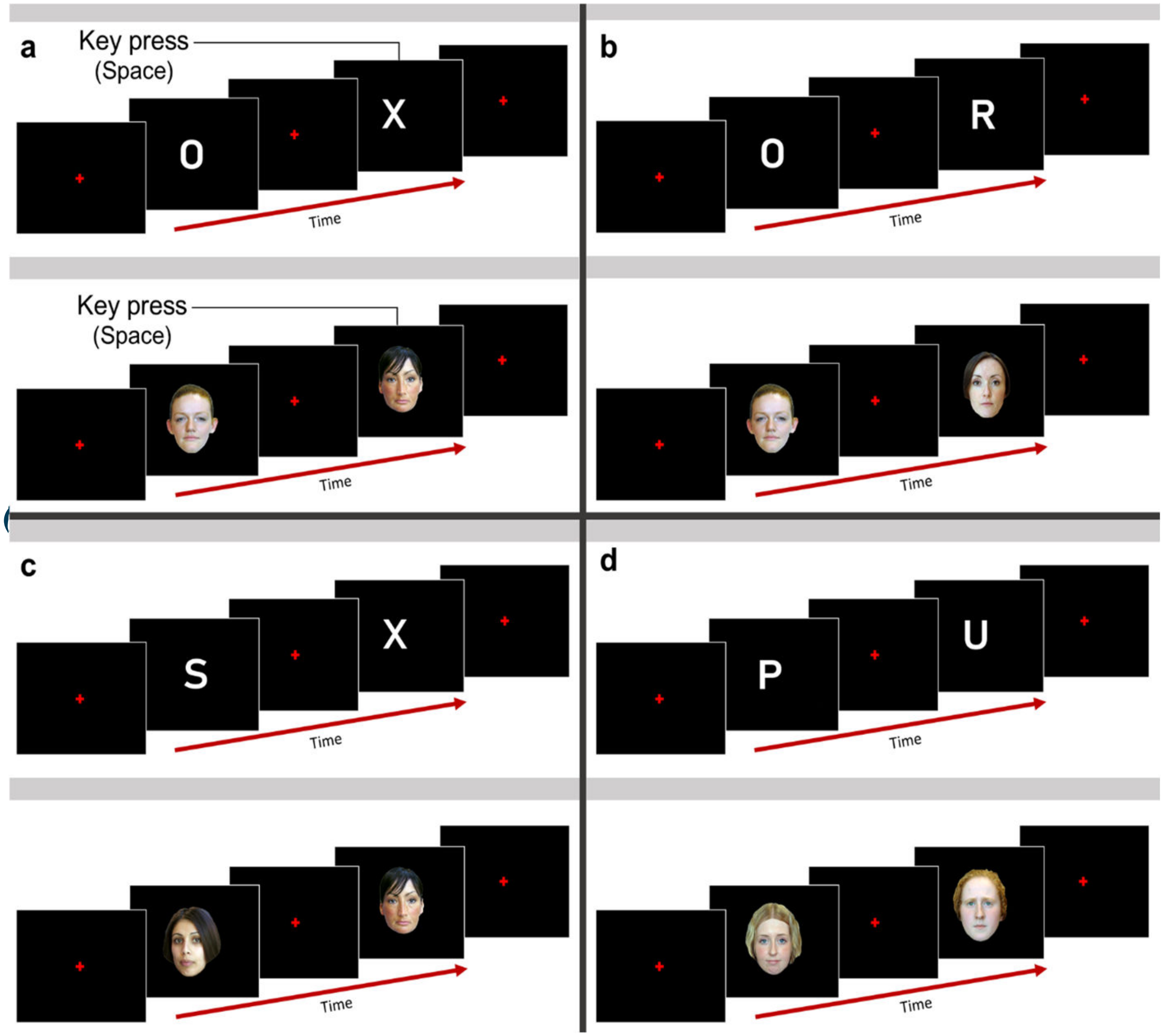

Experimental design

- Stimulus presentation: 1000ms
- Red cross presentation: 1000 ms
- Four possible conditions for each level of letters and faces:

a. OX (The target; required response)
b. O, Not X (No response required)
c. Not O, X (No response required)
d. Not O, Not X (No response required)

(For the complete set of Stimuli and the E-Prime designed experiment check: https://osf.io/38sae/?view_only=778e9eed8ea14465a92c64559103e45e
Under the title: Face_and_Letter_CPT_Experiment.zip)

Behavioral Analysis

- **Participants:** 19ASD, 19NT, age 16-39 years; $M = 23.18$, $SD = 6.22$
- We have response time only for the OX condition. Thus, we ran ANOVA for response time only for the condition OX. ANOVA had two factors: Stimuli type (Levels: Faces and Letters) and Group (Levels: Autism and Control)
- Accuracies. A 2×4×2 analysis of variance (ANOVA) with the factors group (ASD, neurotypical), condition (OX, NotO.X, O.NotX, NotO.NotX), and stimuli type (letters, faces) on proportion correct was conducted.
- **ANOVA for response time:**
- There was a main significant effect of the Stimuli type. Response times were significantly shorter for the letter stimuli compared to face stimuli in both groups (M_{Faces}= 547 ms, $M_{Letters}$= 455 ms, $F(1,36) = 138.11$, $p < .0001$, $\eta^2_p = 0.79$).
- A trend was observed for the factor Group (M_{Autism}= 526 ms, $M_{Control}$= 475 ms, $F(1,36) = 3.41$, $p = 0.073$, $\eta^2_p = 0.09$). There was no significant interaction between Stimuli factor and Group factor.
- **ANOVA for Accuracy:**
- A main effect of group emerged: $F(1,36)$= 7.92, p= .008, $\eta p2$= 0.18 [0.01, 0.40], showing overall higher accuracies for typically developing individuals, and a main effect of condition, $F(1,36)$= 11.67, p= .001, $\eta p2$= 0.24 [0.11, 0.36], which was approved by an interaction of group with condition, $F(3, 108)$= 7.22, p= .009, $\eta p2$= 0.17 [0.05, 0.28]. The follow-up analysis depicted a significant difference between the two groups only during OX condition, $t(36)$= 2.78, p= .009, d= .93 [1.61, 0.23]. Both groups showed the lowest accuracies, first during the condition OX (MASD= 0.96, SE= 008, MTD= 0.99, SE= 0.08) and second during NotO.X (M_{Both}= 0.99, SE= 0.02). No effect of stimuli type was discovered (F<1, p= .720).

Behavioral Analysis

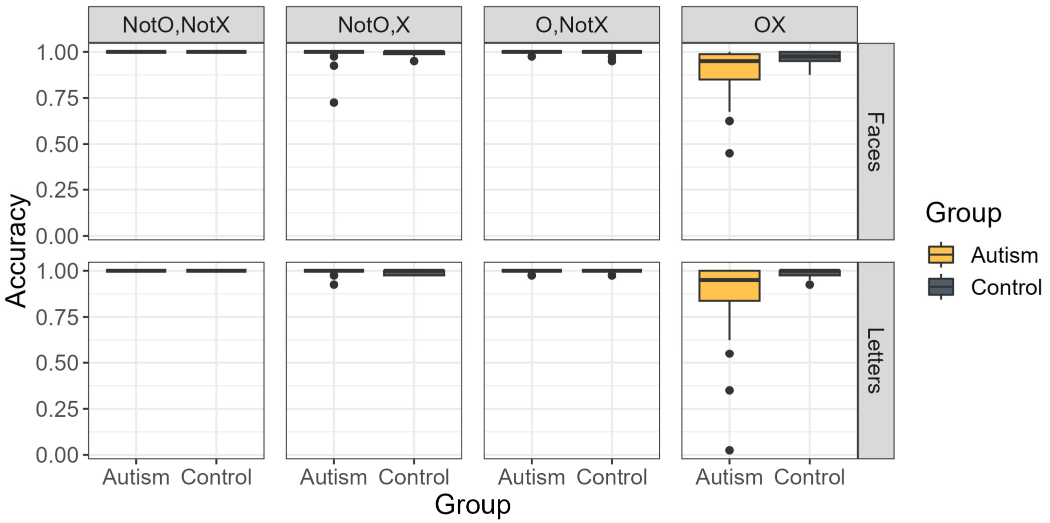

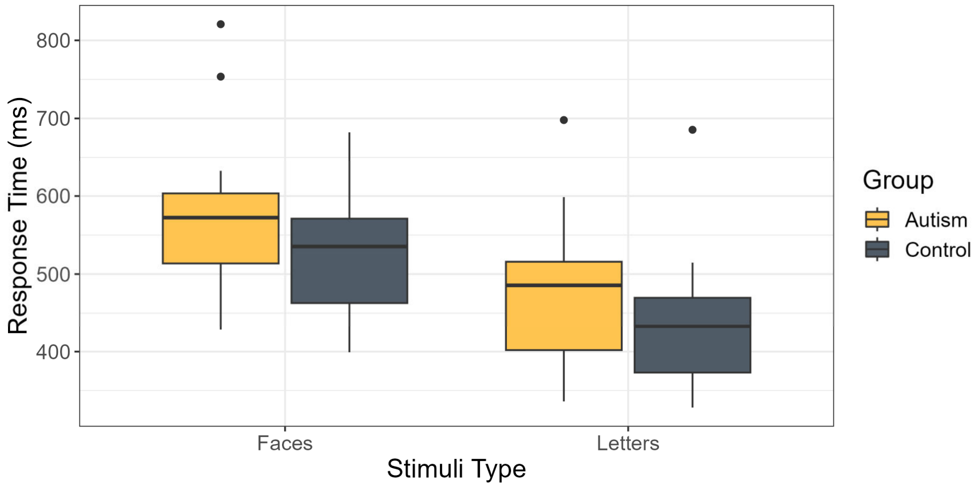

Behavioral Analysis (nonparametric statistics)

- **Response time:**
- **Compare groups**

Ranks										
Condition				N	Mean Rank	Sum of Ranks	Mann-Whitney U	Z	Sig.	Result
OX	Faces	Mean(RT)	Autism	19	22.79	433.00	118.000	-1.825	0.068	Autism = Control
			Control	19	16.21	308.00				
	Letters	Mean(RT)	Autism	19	22.68	431.00	120.000	-1.766	0.077	Autism = Control
			Control	19	16.32	310.00				
Mann-Whitney U										

- **Compare stimulus types**

Ranks									
Group				N	Mean Rank	Sum of Ranks	Z	Sig.	Result
Autism	OX	Mean(RT, Letters) - Mean(RT, Faces)	Negative Ranks	19[a]	10.00	190.00	-3.823[d]	0.000	Mean(RT, Letters) < Mean(RT, Faces)
			Positive Ranks	0[b]	0.00	0.00			
			Ties	0[c]					
Control	OX	Mean(RT, Letters) - Mean(RT, Faces)	Negative Ranks	18[a]	10.50	189.00	-3.783[d]	0.000	Mean(RT, Letters) < Mean(RT, Faces)
			Positive Ranks	1[b]	1.00	1.00			
			Ties	0[c]					
	Wilcoxon Signed Ranks Test								
b. Mean(RT, Letters) > Mean(RT, Faces)	The sum of negative ranks equals the sum of positive ranks.								
c. Mean(RT, Letters) = Mean(RT, Faces)	Based on negative ranks.								
d	Based on positive ranks.								

Behavioral Analysis (nonparametric statistics)

- **Accuracy:**
- **Compare groups**

Ranks										
Stimuli				N	Mean Rank	Sum of Ranks	Mann-Whitney U	Z	Sig.	Result
Faces	NotO,NotX	Acc	Autism	760	760.50	577980.00	288800.000	0.000	1.000	Autism = Control
			Control	760	760.50	577980.00				
	NotO,X	Acc	Autism	760	756.00	574560.00	285380.000	-1.977	0.052	Autism < Control (trend)
			Control	760	765.00	581400.00				
	O,NotX	Acc	Autism	760	762.00	579120.00	287660.000	-1.343	0.179	Autism = Control
			Control	760	759.00	576840.00				
	OX	Acc	Autism	760	724.00	550240.00	261060.000	-7.078	0.000	Autism < Control
			Control	760	797.00	605720.00				
Letters	NotO,NotX	Acc	Autism	760	760.50	577980.00	288800.000	0.000	1.000	Autism = Control
			Control	760	760.50	577980.00				
	NotO,X	Acc	Autism	760	762.50	579500.00	287280.000	-1.074	0.283	Autism = Control
			Control	760	758.50	576460.00				
	O,NotX	Acc	Autism	760	761.00	578360.00	288420.000	-0.448	0.654	Autism = Control
			Control	760	760.00	577600.00				
	OX	Acc	Autism	760	703.00	534280.00	245100.000	-10.297	0.000	Autism < Control
			Control	760	818.00	621680.00				
Mann-Whitney U										

Behavioral Analysis (nonparametric statistics)

- **Accuracy:**
- **Compare conditions**

Ranks							
Group			Mean Rank	Chi-Square	df	Asymp. Sig.	Result
Autism	Faces	Mean(Acc, NotO,NotX)	2.97	30.221	3	0.000	Mean(Acc, OX) > Mean(Acc, O,NotX)
		Mean(Acc, NotO,X)	2.63				
		Mean(Acc, O,NotX)	2.87				
		Mean(Acc, OX)	1.53				
	Letters	Mean(Acc, NotO,NotX)	2.92	19.258	3	0.000	Mean(Acc, OX) > Mean(Acc, NotO,NotX)
		Mean(Acc, NotO,X)	2.61				
		Mean(Acc, O,NotX)	2.74				
		Mean(Acc, OX)	1.74				
Control	Faces	Mean(Acc, NotO,NotX)	3.00	18.815	3	0.000	Mean(Acc, OX)>Mean(Acc, NotO,NotX)
		Mean(Acc, NotO,X)	2.50				
		Mean(Acc, O,NotX)	2.71				
		Mean(Acc, OX)	1.79				
	Letters	Mean(Acc, NotO,NotX)	3.00	12.095	3	0.007	
		Mean(Acc, NotO,X)	2.08				
		Mean(Acc, O,NotX)	2.68				
		Mean(Acc, OX)	2.24				
Friedman Test							

Behavioral Analysis (nonparametric statistics)

- **Accuracy:**
- **Compare stimuli types**

Ranks

Group				N	Mean Rank	Sum of Ranks	Z	Sig.	Result
Autism	NotO,NotX	Mean(Acc, Letters) - Mean(Acc, Faces)	Negative Ranks	0[a]	0.00	0.00	.000[b]	1.000	Mean(Acc, Letters) = Mean(Acc, Faces)
			Positive Ranks	0[b]	0.00	0.00			
			Ties	19[c]					
	NotO,X	Mean(Acc, Letters) - Mean(Acc, Faces)	Negative Ranks	2[a]	2.00	4.00	-.962[c]	0.336	Mean(Acc, Letters) = Mean(Acc, Faces)
			Positive Ranks	3[b]	3.67	11.00			
			Ties	14[c]					
	O,NotX	Mean(Acc, Letters) - Mean(Acc, Faces)	Negative Ranks	2[a]	2.00	4.00	-.577[d]	0.564	Mean(Acc, Letters) = Mean(Acc, Faces)
			Positive Ranks	1[b]	2.00	2.00			
			Ties	16[c]					
	OX	Mean(Acc, Letters) - Mean(Acc, Faces)	Negative Ranks	9[a]	9.33	84.00	-.833[d]	0.405	Mean(Acc, Letters) = Mean(Acc, Faces)
			Positive Ranks	7[b]	7.43	52.00			
			Ties	3[c]					
Control	NotO,NotX	Mean(Acc, Letters) - Mean(Acc, Faces)	Negative Ranks	0[a]	0.00	0.00	.000[b]	1.000	Mean(Acc, Letters) = Mean(Acc, Faces)
			Positive Ranks	0[b]	0.00	0.00			
			Ties	19[c]					
	NotO,X	Mean(Acc, Letters) - Mean(Acc, Faces)	Negative Ranks	7[a]	5.00	35.00	-.832[d]	0.405	Mean(Acc, Letters) = Mean(Acc, Faces)
			Positive Ranks	3[b]	6.67	20.00			
			Ties	9[c]					
	O,NotX	Mean(Acc, Letters) - Mean(Acc, Faces)	Negative Ranks	2[a]	2.00	4.00	-.378[c]	0.705	Mean(Acc, Letters) = Mean(Acc, Faces)
			Positive Ranks	2[b]	3.00	6.00			
			Ties	15[c]					
	OX	Mean(Acc, Letters) - Mean(Acc, Faces)	Negative Ranks	2[a]	3.00	6.00	-1.725[c]	0.084	Mean(Acc, Letters) = Mean(Acc, Faces)
			Positive Ranks	6[b]	5.00	30.00			
			Ties	11[c]					
a. Mean(Acc, Letters) < Mean(Acc, Faces)		Wilcoxon Signed Ranks Test							
b. Mean(Acc, Letters) > Mean(Acc, Faces)									
c. Mean(Acc, Letters) = Mean(Acc, Faces)									

Study design

- **Participants:** 17ASD, 18NT (2 ASD an 1NT individuals had less than 15 accepted trials and got excluded in ERP analysis)
- Participants had to have at least 15 accepted trials per condition after artifact rejection to be included in the ERP analysis (trials with missing the proper behavioral responses were excluded)
- **There are four possible conditions for each level of letters and faces (each 40 trials):(*The numbers show the condition number in KN, see CPT_mean_trials.xlsx in OSF database)**

1. Letters OX : The target, *M*=35.91

2. Letters Not O, X: *M*=37.09

3. Letters O, Not X: *M*=37.17

4. Letters Not O, Not X: *M=38*

5. Faces OX: The target, *M*=36.2

6. Faces Not O, X: *M*=37.4

7. Faces O, Not X: *M*=38.31

8. Faces Not O, Not X: *M*=37.77

- **ERPs:** Epochs were established offline from −200 to 1000 ms relative to S2 (For CNV, from -2200 to 200 relative to S2). Thresholds for subsequent automatic artifact rejection were 100 µV for amplitude, 75 µV for gradient, and 0.01 µV for low signal. Baseline correction of ERP amplitudes was performed for the interval from −200 to 0 ms relative to S2 (-2200 to -2000 relative to S2). ERPs were averaged separately for each experimental condition, digitally low-pass filtered (data were filtered low pass 30 Hz 24db/oct and high pass 0.30 Hz 12db/oct, both zero phase shift), and recalculated to average reference, excluding the vertical EOG. Components of interest are P100 relative toS2 at O1/O2; N1/N170, P200, and N250 ERPs to S2 at P7, P8, P9, P10, TP9, TP10, PO7, PO8, PO9, PO10 and P300 at C3, Cz, C4, and P3, Pz, P4.

ERP Analysis (Letter-CPT)

- **P100**: (100-140ms)
- 4*2*2 within and between design. Factors: Condition (OX,NotOX,ONotX,NotONotX), Electrode (O1,O2), Group (ASD, TD)
- *Throughout the document, if it was necessary, p-values were HF-adjusted. p> 0.1000 are considered not significant and, therefore sometimes not reported. 0.05<p<0.1 are considered trend. All scripts are available at OSF

  - **Condition F(3, 99) = 11.472, p < .001, np2 = .258**

Level1	Level2	Difference	95% CI	SE	t(33)	p	d	95% CI
NotO.NotX	NotO.X	0.68	[0.24, 1.13]	0.16	4.33	< .001	0.75	[0.36, 1.13]
NotO.NotX	O.NotX	-0.12	[-0.62, 0.38]	0.18	-0.67	> .999	-0.12	[-0.46, 0.23]
NotO.NotX	OX	0.73	[0.14, 1.33]	0.21	3.49	0.004	0.61	[0.23, 0.97]
NotO.X	O.NotX	-0.80	[-1.27, -0.34]	0.17	-4.82	< .001	-0.84	[-1.23, -0.44]
NotO.X	OX	0.05	[-0.49, 0.59]	0.19	0.26	> .999	0.05	[-0.30, 0.39]
O.NotX	OX	0.85	[0.27, 1.44]	0.21	4.09	0.001	0.71	[0.33, 1.09]

Estimated Marginal Means

Condition	Mean	SE	95% CI
NotO.NotX	3.16	0.33	[2.49, 3.82]
NotO.X	2.47	0.38	[1.71, 3.24]
O.NotX	3.28	0.35	[2.57, 3.98]
OX	2.42	0.36	[1.69, 3.16]

Marginal means estimated at Condition

- Marginal contrasts estimated at Condition
- p-value adjustment method: Holm (1979)
- d is Cohen's d effect size and the CI next to it is the confidence interval for the effect sizes.

ERP Analysis (Letter-CPT)

- **N170**: (140-190 ms)
- 4*4*2*2 within and between design. Factors: Condition (OX,NotOX,ONotX,NotONotX), Site (P7/P8, P9/P10, PO7/PO8, PO9/PO10), Hemisphere (Right , Left), and Group (ASD, Neurotypical)
    - **Condition F(3, 99) = 22.899, p < .001, np2 = .410**
- **Following up on the main effect of condition**:

Level1	Level2	Difference	95% CI	SE	t(33)	p	d	95% CI
NotO.NotX	NotO.X	-0.39	[-0.84, 0.06]	0.16	-2.44	0.041	0.42	[-0.78, -0.07]
NotO.NotX	O.NotX	1.16	[0.59, 1.72]	0.20	5.73	< .001	1.00	[0.58, 1.41]
NotO.NotX	OX	0.72	[0.03, 1.42]	0.25	2.93	0.018	0.51	[0.14, 0.87]
NotO.X	O.NotX	1.55	[0.92, 2.18]	0.22	6.90	< .001	1.20	[0.75, 1.64]
NotO.X	OX	1.11	[0.69, 1.54]	0.15	7.39	< .001	1.29	[0.82, 1.74]
O.NotX	OX	-0.43	[-1.09, 0.22]	0.23	-1.86	0.072	-0.32	[-0.67, 0.03]

Estimated Marginal Means

Condition	Mean	SE	95% CI
NotO.NotX	-1.26	0.33	[-1.92, -0.60]
NotO.X	-0.87	0.28	[-1.44, -0.30]
O.NotX	-2.42	0.35	[-3.14, -1.70]
OX	-1.98	0.32	[-2.63, -1.34]

Marginal means estimated at Condition

- Marginal contrasts estimated at Condition
- p-value adjustment method: Holm (1979)
- **Seems that we have enhanced N170 whenever O is the first stimulus, mostly enhanced in O,NotX condition.**

ERP Analysis (Letter-CPT)

- **P200**: (190-240 ms)
- 4*2*2*2 within and between design. Factors: Condition (OX,NotOX,ONotX,NotONotX), Site (P7/P8, PO7/PO8), Hemisphere (Right, Left) and Group (ASD, Neurotypical)
  - **Condition $F(3, 99) = 11.947$, $p < .001$, $np2 = .266$**
  - **Site $F(1, 33) = 71.424$, $p < .001$, $np2 = .684$**
  - **Condition:Hemisphere $F(3, 99) = 6.286$, $p = .001$, $np2 = .160$**
  - **Following up on the main effect of condition**:

Level1	Level2	Difference	95% CI	SE	t(33)	p	d	95% CI
NotO.NotX	NotO.X	0.16	[-0.35, 0.67]	0.18	0.90	> .999	0.16	[-0.19, 0.50]
NotO.NotX	O.NotX	0.15	[-0.30, 0.61]	0.16	0.94	> .999	0.16	[-0.18, 0.51]
NotO.NotX	OX	1.18	[0.50, 1.86]	0.24	4.85	< .001	0.84	[0.44, 1.24]
NotO.X	O.NotX	-0.01	[-0.69, 0.67]	0.24	-0.04	> .999	-7.54e-03	[-0.35, 0.33]
NotO.X	OX	1.02	[0.44, 1.59]	0.20	4.98	< .001	0.87	[0.46, 1.26]
O.NotX	OX	1.03	[0.25, 1.81]	0.28	3.71	0.003	0.65	[0.27, 1.02]

- Marginal contrasts estimated at Condition
- p-value adjustment method: Holm (1979)

Condition	Mean	SE	95% CI
NotO.NotX	-0.39	0.31	[-1.03, 0.25]
NotO.X	-0.66	0.28	[-1.22, -0.10]
O.NotX	-0.53	0.35	[-1.25, 0.19]
OX	-1.72	0.34	[-2.43, -1.02]

Marginal means estimated at Condition

***Seems that the target (OX) has the lowest P200, and is significant against others**

ERP Analysis (Letter-CPT)

- **Continued: P200**: (190-240 ms)
- **Site F(1, 33) = 71.424, p < .001, np2 = .684**
- **It has only two levels and thus we look only at the means:**

Site	Mean	SE	95% CI
P7.P8	-0.38	0.27	[-0.92, 0.17]
PO7.PO8	1.03	0.34	[0.33, 1.73]

Marginal means estimated at Site

- ***Seems that P200 has its highest amplitude on PO7/PO8 sites**

ERP Analysis (Letter-CPT)

- **Continued: P200**: (190-240 ms)
- **Following up on the interaction of**
- **Condition:Hemisphere F(3, 99) = 5.470, p = .002, np2 = .142**
- **On the left hemisphere:**

Level1	Level2	Difference	95% CI	SE	t(33)	p
NotO.NotX	OX	Left	1.72	[0.92, 2.53]	0.29	5.99	< .001
NotO.X	OX	Left	1.36	[0.73, 1.99]	0.22	6.06	< .001
O.NotX	OX	Left	1.71	[0.81, 2.60]	0.32	5.36	< .001

- **On the right hemisphere:**

Nothing is significant, t<2.43, p> 0.12

***On the left side, OX is significantly lower than the other three conditions. On the right side, OX is significantly lower only comparing to the conditions "NotO,X" and "NotO, NotX"**

Condition	Hemisphere	Mean	SE	95% CI
NotO.NotX	Left	0.61	0.26	[0.08, 1.13]
NotO.X	Left	0.24	0.32	[-0.41, 0.90]
O.NotX	Left	0.59	0.32	[-0.06, 1.25]
OX	Left	-1.12	0.39	[-1.90, -0.33]
NotO.NotX	Right	0.79	0.45	[-0.12, 1.70]
NotO.X	Right	0.83	0.41	[-0.01, 1.66]
O.NotX	Right	0.50	0.49	[-0.50, 1.50]
OX	Right	0.16	0.44	[-0.73, 1.04]

Marginal means estimated at Condition, Hemisphere

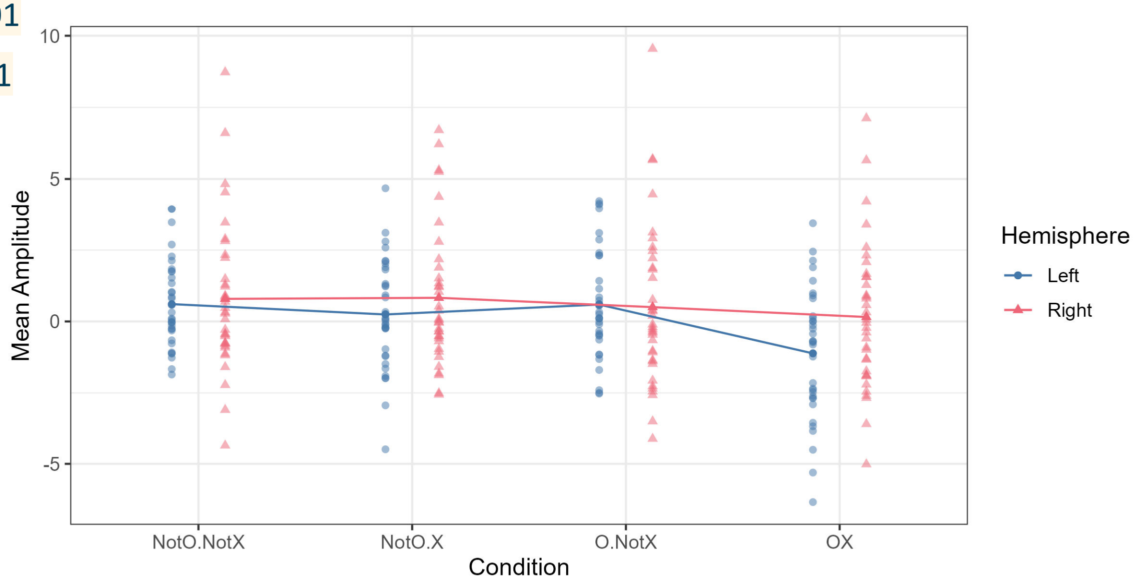

ERP Analysis (Letter-CPT)

- **N250**: (260-340 ms)
- 4*5*2*2 within and between design. Factors: Condition (OX,NotOX,ONotX,NotONotX), Site (P7/P8, P9/P10, PO7/PO8, PO9/PO10, TP9/TP10), Hemisphere, and Group (ASD, Neurotypical)
- **Condition F(3, 99) = 39.179, p < .001, np2 = .543**
- **Site F(4, 132) = 110.373, p < .001, np2 = .770**
- **Condition:Site F(12, 396) = 21.132, p < .001, np2 = .390**
- **Following up on the main effect of condition**:

Level1	Level2	Difference	95% CI	SE	t(33)	p	d	95% CI
NotO.NotX	NotO.X	0.40	[0.01, 0.79]	0.14	2.91	0.013	0.51	[0.14, 0.87]
NotO.NotX	O.NotX	2.15	[1.23, 3.08]	0.33	6.53	< .001	1.14	[0.69, 1.57]
NotO.NotX	OX	2.78	[1.93, 3.63]	0.30	9.19	< .001	1.60	[1.08, 2.11]
NotO.X	O.NotX	1.76	[0.69, 2.82]	0.38	4.61	< .001	0.80	[0.41, 1.19]
NotO.X	OX	2.38	[1.49, 3.27]	0.32	7.52	< .001	1.31	[0.84, 1.77]
O.NotX	OX	0.63	[-0.21, 1.47]	0.30	2.10	0.043	0.37	[0.01, 0.72]

- Marginal contrasts estimated at Condition
- p-value adjustment method: Holm (1979)

Condition	Mean	SE	95% CI
NotO.NotX	-0.24	0.21	[-0.67, 0.18]
NotO.X	-0.64	0.22	[-1.08, -0.20]
O.NotX	-2.40	0.36	[-3.13, -1.67]
OX	-3.03	0.33	[-3.71, -2.35]

Marginal means estimated at Condition

***Conditions OX and O,Not X had the most enhanced N250 and were significant comparing to all other conditions.**

ERP Analysis (Letter-CPT)

- **Continued N250**: (260-340 ms)
- **Following up on the main effect of Site $F(4, 132) = 110.373$, $p < .001$, np2 = .770**

Level1	Level2	Difference	95% CI	SE	t(33)	p	d	95% CI
P7.P8	P9.P10	2.61	[1.86, 3.36]	0.25	10.45	< .001	1.82	[1.26, 2.37]
P7.P8	PO7.PO8	-1.28	[-1.85, -0.72]	0.19	-6.88	< .001	-1.20	[-1.64, -0.75]
P9.P10	PO7.PO8	-3.89	[-4.89, -2.90]	0.33	-11.77	< .001	-2.05	[-2.64, -1.44]
PO9.PO10	P7.P8	-2.00	[-2.57, -1.42]	0.19	-10.48	< .001	-1.82	[-2.37, -1.26]
PO9.PO10	P9.P10	0.61	[0.11, 1.12]	0.17	3.64	0.002	0.63	[0.26, 1.00]
PO9.PO10	PO7.PO8	-3.28	[-4.03, -2.53]	0.25	-13.11	< .001	-2.28	[-2.92, -1.63]
TP9.TP10	P7.P8	-3.08	[-3.88, -2.29]	0.26	-11.70	< .001	-2.04	[-2.63, -1.44]
TP9.TP10	P9.P10	-0.48	[-0.98, 0.02]	0.17	-2.87	0.007	-0.50	[-0.86, -0.13]
TP9.TP10	PO7.PO8	-4.37	[-5.53, -3.21]	0.39	-11.34	< .001	-1.97	[-2.55, -1.38]
TP9.TP10	PO9.PO10	-1.09	[-1.72, -0.46]	0.21	-5.20	< .001	-0.90	[-1.30, -0.50]

- Marginal contrasts estimated at Site
- p-value adjustment method: Holm (1979) ***As expected, the site with the most enhanced N250 is TP9/TP10**

Site	Mean	SE	95% CI
TP9.TP10	-3.38	0.28	[-3.95, -2.81]
PO9.PO10	-2.29	0.26	[-2.82, -1.76]
P7.P8	-0.30	0.25	[-0.80, 0.20]
P9.P10	-2.90	0.29	[-3.49, -2.32]
PO7.PO8	0.99	0.28	[0.42, 1.55]

Marginal means estimated at Site

ERP Analysis (Letter-CPT)

- **Continued N250**: (260-340 ms)
- **Following up on the interaction of Condition:Site F(12, 396) = 21.132, p < .001, np2 = .390**

- **On the P7/P8 site**

Level1	Level2	Site	Difference	95% CI	SE	t(33)	p
NotO.NotX	NotO.X	P7.P8	0.36	[-0.03, 0.75]	0.14	2.61	0.027
NotO.NotX	O.NotX	P7.P8	2.04	[1.30, 2.78]	0.26	7.77	< .001
NotO.NotX	OX	P7.P8	1.53	[0.83, 2.23]	0.25	6.17	< .001
NotO.X	O.NotX	P7.P8	1.68	[0.71, 2.64]	0.34	4.89	< .001
NotO.X	OX	P7.P8	1.17	[0.38, 1.97]	0.28	4.14	< .001
O.NotX	OX	P7.P8	-0.51	[-1.30, 0.29]	0.28	-1.79	0.082

- Marginal contrasts estimated at Condition
- p-value adjustment method: Holm (1979)

Condition	Site	Mean	SE	95% CI
NotO.NotX	P7.P8	0.69	0.26	[0.17, 1.21]
NotO.X	P7.P8	0.33	0.25	[-0.18, 0.83]
O.NotX	P7.P8	-1.35	0.35	[-2.07, -0.64]
OX	P7.P8	-0.84	0.31	[-1.48, -0.21]

Marginal means estimated at Site

***For more medial sites (P7/P8, and PO7/PO8) N250 is mostly enhanced for O.NotX condition, and for more lateral sites, maybe sites closer to fusiform word area, (P9/P10,PO9/PO10, TP9/TP10), OX is the condition that wins the most enhanced N200.**

ERP Analysis (Letter-CPT)

- **Continued N250**: (260-340 ms)
- **Following up on the interaction of Condition:Site F(12, 396) = 21.132, p < .001, np2 = .390**
- **On the P9/P10 site**

Level1	Level2	Site	Difference	95% CI	SE	t(33)	p
NotO.NotX	NotO.X	P9.P10	0.40	[-0.20, 0.99]	0.21	1.88	0.069
NotO.NotX	O.NotX	P9.P10	2.28	[1.08, 3.48]	0.43	5.35	< .001
NotO.NotX	OX	P9.P10	3.82	[2.61, 5.02]	0.43	8.88	< .001
NotO.X	O.NotX	P9.P10	1.89	[0.57, 3.20]	0.47	4.02	< .001
NotO.X	OX	P9.P10	3.42	[2.23, 4.61]	0.42	8.08	< .001
O.NotX	OX	P9.P10	1.53	[0.56, 2.51]	0.35	4.40	< .001

- Marginal contrasts estimated at Condition
- p-value adjustment method: Holm (1979)

Condition	Site	Mean	SE	95% CI
NotO.NotX	P9.P10	-1.28	0.25	[-1.79, -0.77]
NotO.X	P9.P10	-1.68	0.28	[-2.24, -1.11]
O.NotX	P9.P10	-3.56	0.45	[-4.48, -2.65]
OX	P9.P10	-5.10	0.47	[-6.05, -4.14]

Marginal means estimated at Site

ERP Analysis (Letter-CPT)

- **Continued N250**: (260-340 ms)
- **Following up on the interaction of Condition:Site F(12, 396) = 21.132, p < .001, np2 = .390**
- **On the PO7/PO8 site**
- Level1 | Level2 | Site | Difference | 95% CI | SE | t(33) | p
- ---
- NotO.NotX | NotO.X | PO7.PO8 | 0.46 | [0.08, 0.85] | 0.14 | 3.38 | 0.006
- NotO.NotX | O.NotX | PO7.PO8 | 1.98 | [1.26, 2.70] | 0.26 | 7.75 | < .001
- NotO.NotX | OX | PO7.PO8 | 1.11 | [0.41, 1.80] | 0.25 | 4.47 | < .001
- NotO.X | O.NotX | PO7.PO8 | 1.52 | [0.62, 2.42] | 0.32 | 4.76 | < .001
- NotO.X | OX | PO7.PO8 | 0.64 | [-0.07, 1.36] | 0.26 | 2.52 | 0.033
- O.NotX | OX | PO7.PO8 | -0.88 | [-1.85, 0.10] | 0.35 | -2.52 | 0.033
- Marginal contrasts estimated at Condition
- p-value adjustment method: Holm (1979)

Condition	Site	Mean	SE	95% CI
NotO.NotX	PO7.PO8	1.88	0.27	[1.32, 2.43]
NotO.X	PO7.PO8	1.41	0.31	[0.79, 2.04]
O.NotX	PO7.PO8	-0.11	0.34	[-0.80, 0.58]
OX	PO7.PO8	0.77	0.36	[0.03, 1.50]

Marginal means estimated at Site

ERP Analysis (Letter-CPT)

- **Continued N250**: (260-340 ms)
- **Following up on the interaction of Condition:Site F(12, 396) = 21.132, p < .001, np2 = .390**
- **On the PO9/PO10 site**

Level1	Level2	Site	Difference	95% CI	SE	t(33)	p
NotO.NotX	NotO.X	PO9.PO10	0.48	[-0.06, 1.03]	0.19	2.51	0.017
NotO.NotX	O.NotX	PO9.PO10	2.23	[1.16, 3.29]	0.38	5.88	< .001
NotO.NotX	OX	PO9.PO10	3.49	[2.43, 4.54]	0.38	9.29	< .001
NotO.X	O.NotX	PO9.PO10	1.74	[0.50, 2.98]	0.44	3.94	0.001
NotO.X	OX	PO9.PO10	3.00	[1.88, 4.12]	0.40	7.54	< .001
O.NotX	OX	PO9.PO10	1.26	[0.17, 2.35]	0.39	3.24	0.005

- Marginal contrasts estimated at Condition
- p-value adjustment method: Holm (1979)

Condition	Site	Mean	SE	95% CI
NotO.NotX	PO9.PO10	-0.74	0.24	[-1.22, -0.27]
NotO.X	PO9.PO10	-1.23	0.26	[-1.75, -0.71]
O.NotX	PO9.PO10	-2.97	0.42	[-3.83, -2.11]
OX	PO9.PO10	-4.23	0.42	[-5.09, -3.37]

Marginal means estimated at Site

ERP Analysis (Letter-CPT)

- **Continued N250**: (260-340 ms)
- **Following up on the interaction of Condition:Site F(12, 396) = 21.132, p < .001, np2 = .390**
- **On the TP9/TP10 site**

Level1	Level2	Site	Difference	95% CI	SE	t(33)	p
NotO.NotX	O.NotX	TP9.TP10	2.24	[0.95, 3.53]	0.46	4.87	< .001
NotO.NotX	OX	TP9.TP10	3.97	[2.58, 5.37]	0.50	7.99	< .001
NotO.X	O.NotX	TP9.TP10	1.95	[0.48, 3.41]	0.52	3.73	0.001
NotO.X	OX	TP9.TP10	3.68	[2.36, 5.00]	0.47	7.81	< .001
O.NotX	OX	TP9.TP10	1.73	[0.68, 2.78]	0.37	4.63	< .001

- Marginal contrasts estimated at Condition
- p-value adjustment method: Holm (1979)

Condition	Site	Mean	SE	95% CI
NotO.NotX	TP9.TP10	-1.75	0.22	[-2.19, -1.32]
NotO.X	TP9.TP10	-2.05	0.29	[-2.64, -1.46]
O.NotX	TP9.TP10	-3.99	0.46	[-4.94, -3.05]
OX	TP9.TP10	-5.73	0.50	[-6.75, -4.70]

Marginal means estimated at Site

ERP Analysis (Letter-CPT)

- **Continued N250**: (260-340 ms)
- **Following up on the interaction of Condition:Site F(12, 396) = 21.132, p < .001, np2 = .390**
- **The interaction plot:**

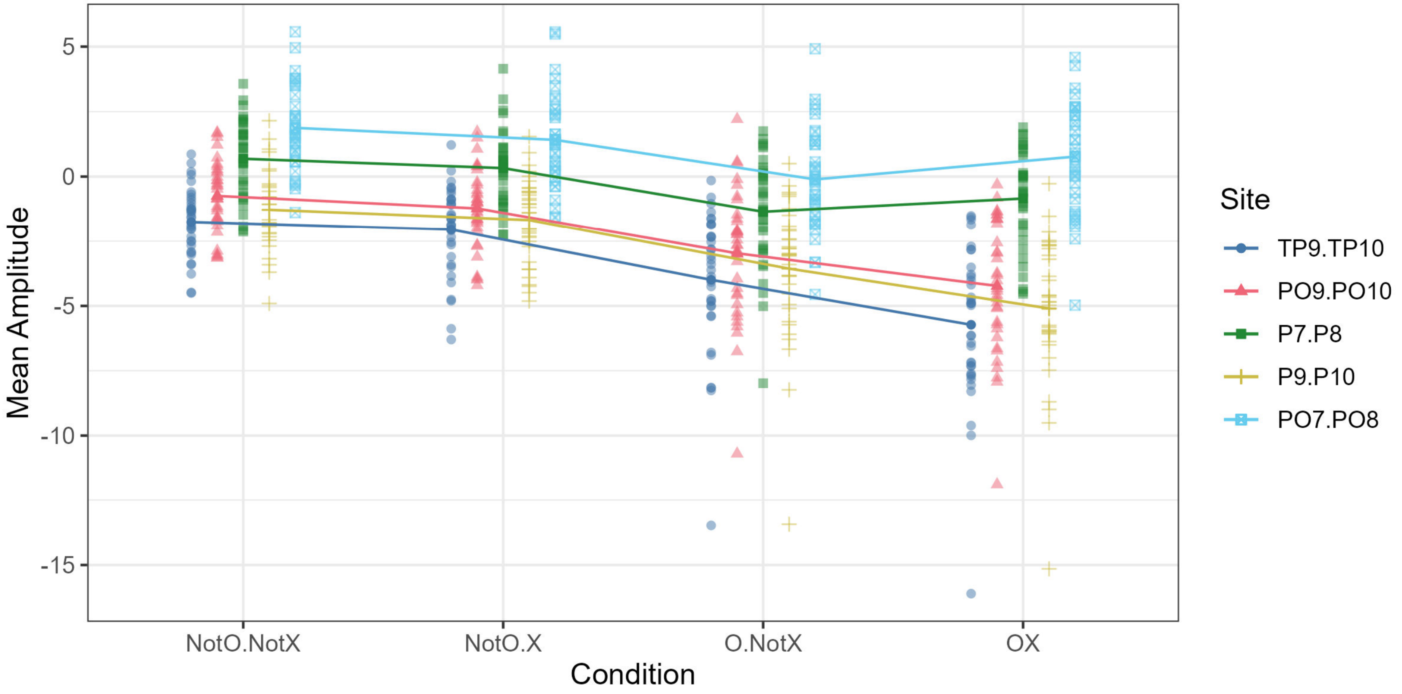

ERP Analysis (Letter-CPT)

- **P300** (300-400 ms)

4*2*3*2 within and between design. Factors: Condition (OX,NotOX,ONotX,NotONotX), Anteriority (Central (C3/Cz/C4), Parietal (P3/Pz/P4), Laterality (Right, Left, Central), Group (ASD, Neurotypical)

- **Main effect of Condition $F(3, 99) = 59.024$, $p < .001$, np2 = .641**
- **Main effect of Laterality $F(2, 66) = 6.661$, $p = .002$, np2 = .168**
- **Main effect of Anteriority $F(1, 33) = 25.221$, $p < .001$, np2 = .433**
- **Interaction Condition:Laterality $F(6, 198) = 14.796$, $p < .001$, np2 = .310**
- **Interaction Condition: Anteriority $F(3, 99) = 52.440$, $p < .001$, np2 = .614**
- **Interaction Laterality:Site $F(2, 66) = 2.431$, $p = .096$, np2 = .069**
- **Interaction Group:Condition:Laterality:Site $F(6, 198) = 1.963$, $p = .082$, np2 = .056**

ERP Analysis (Letter-CPT)

- **Continued P300** (300-400 ms)

4*2*3*2 within and between design. Factors: Condition (OX,NotOX,ONotX,NotONotX), Site (Central (C3/Cz/C4), Parietal (P3/Pz/P4), Laterality (Right, Left, Central), Group (ASD, Neurotypical)

Following up on the main effect of Condition: Main effect of Condition F(3, 99) = 59.024, p < .001, np2 = .641

Level1	Level2	Difference	95% CI	SE	t(33)	p	d	95% CI
NotO.NotX	NotO.X	-0.31	[-0.83, 0.21]	0.18	-1.67	0.104	0.29	[-0.64, 0.06]
NotO.NotX	O.NotX	-2.55	[-3.38, -1.72]	0.30	-8.58	< .001	-1.49	[-1.98, -0.99]
NotO.NotX	OX	-3.40	[-4.41, -2.40]	0.36	-9.51	< .001	-1.66	[-2.17, -1.13]
NotO.X	O.NotX	-2.24	[-3.10, -1.38]	0.31	-7.32	< .001	-1.27	[-1.73, -0.81]
NotO.X	OX	-3.09	[-4.01, -2.18]	0.33	-9.47	< .001	-1.65	[-2.17, -1.12]
O.NotX	OX	-0.85	[-1.81, 0.11]	0.34	-2.50	0.035	-0.43	[-0.79, -0.07]

Condition	Mean	SE	95% CI
NotO.NotX	0.80	0.15	[0.50, 1.11]
NotO.X	1.11	0.17	[0.77, 1.46]
O.NotX	3.35	0.33	[2.69, 4.02]
OX	4.21	0.38	[3.43, 4.98]

Marginal means estimated at Condition

- Marginal contrasts estimated at Condition
- p-value adjustment method: Holm (1979) ***We had the most enhanced P300 on conditions with O as the fist stimulus**

ERP Analysis (Letter-CPT)

- **Continued P300** (300-400 ms)

4*2*3*2 within and between design. Factors: Condition (OX,NotOX,ONotX,NotONotX), Site (Central (C3/Cz/C4), Parietal (P3/Pz/P4), Laterality (Right, Left, Central), Group (ASD, Neurotypical)

Following up on the main effect of Laterality: Main effect of Laterality F(2, 66) = 6.661, p = .002, np2 = .168

- Marginal Contrasts Analysis

Level1	Level2	Difference	95% CI	SE	t(33)	p	d	95% CI
Central	Right	0.47	[0.05, 0.88]	0.16	2.86	0.015	0.50	[0.13, 0.86]
Left	Central	-0.53	[-0.96, -0.11]	0.17	-3.15	0.010	-0.55	[-0.91, -0.18]
Left	Right	-0.06	[-0.42, 0.30]	0.14	-0.45	0.657	-0.08	[-0.42, 0.26]

Laterality	Mean	SE	95% CI
Left	2.17	0.19	[1.79, 2.55]
Central	2.70	0.28	[2.14, 3.27]
Right	2.24	0.19	[1.85, 2.62]

Marginal means estimated at Laterality

- Marginal contrasts estimated at Laterality
- p-value adjustment method: Holm (1979)

***Enhanced P300 on the central areas as expected**

ERP Analysis (Letter-CPT)

- **Continued P300** (300-400 ms)

4*2*3*2 within and between design. Factors: Condition (OX,NotOX,ONotX,NotONotX), Site (Central (C3/Cz/C4), Parietal (P3/Pz/P4), Laterality (Right, Left, Central), Group (ASD, Neurotypical)

Main effect of Site F(1, 33) = 25.221, p < .001, np2 = .433

Following up on the main effect of Site: it has two levels (central and parietal) and we only need to look at the means:

Estimated Marginal Means

Site	Mean	SE	95% CI
C3.Cz.C4	1.81	0.22	[1.36, 2.26]
P3.Pz.P4	2.93	0.24	[2.44, 3.42]

Marginal means estimated at Site

ERP Analysis (Letter-CPT)

- **Continued P300** (300-400 ms)

Following up on the interaction of Condition:Laterality F(6, 198) = 14.796, p < .001, np2 = .310

When the laterality is right:

Level1	Level2	Laterality	Difference	95% CI	SE	t(33)	p
NotO.NotX	NotO.X	Right	-0.06	[-0.53, 0.41]	0.17	-0.35	0.726
NotO.NotX	O.NotX	Right	-2.13	[-3.01, -1.26]	0.31	-6.83	< .001
NotO.NotX	OX	Right	-2.99	[-4.09, -1.90]	0.39	-7.71	< .001
NotO.X	O.NotX	Right	-2.07	[-3.02, -1.13]	0.34	-6.17	< .001
NotO.X	OX	Right	-2.94	[-4.03, -1.84]	0.39	-7.52	< .001
O.NotX	OX	Right	-0.86	[-1.63, -0.10]	0.27	-3.17	0.007

Marginal contrasts estimated at Condition

p-value adjustment method: Holm (1979)

Condition	Laterality	Mean	SE	95% CI
NotO.NotX	Right	0.94	0.19	[0.56, 1.32]
NotO.X	Right	1.00	0.18	[0.64, 1.36]
O.NotX	Right	3.07	0.31	[2.43, 3.71]
OX	Right	3.93	0.36	[3.20, 4.67]

Marginal means estimated at Condition, Laterality

ERP Analysis (Letter-CPT)

- **Continued P300** (300-400ms)

Following up on the interaction of Condition:Laterality F(6, 198) = 14.796, p < .001, np2 = .310

When the laterality is Central:

Level1	Level2	Laterality	Difference	95% CI	SE	t(33)	p
NotO.NotX	NotO.X	Central	-0.82	[-1.57, -0.07]	0.27	-3.06	0.009
NotO.NotX	O.NotX	Central	-3.99	[-5.20, -2.78]	0.43	-9.27	< .001
NotO.NotX	OX	Central	-4.44	[-5.81, -3.07]	0.49	-9.09	< .001
NotO.X	O.NotX	Central	-3.17	[-4.39, -1.96]	0.43	-7.31	< .001
NotO.X	OX	Central	-3.62	[-4.84, -2.40]	0.43	-8.34	< .001
O.NotX	OX	Central	-0.45	[-1.95, 1.05]	0.53	-0.84	0.404

Marginal contrasts estimated at Condition

p-value adjustment method: Holm (1979)

Condition	Laterality	Mean	SE	95% CI
NotO.NotX	Central	0.39	0.23	[-0.08, 0.86]
NotO.X	Central	1.21	0.25	[0.70, 1.72]
O.NotX	Central	4.38	0.47	[3.42, 5.34]
OX	Central	4.83	0.51	[3.80, 5.86]

Marginal means estimated at Condition, Laterality

ERP Analysis (Letter-CPT)

- **Continued P300**

Following up on the interaction of Condition:Laterality F(6, 198) = 14.796, p < .001, np2 = .310

When the laterality is Left:

Level1	Level2	Laterality	Difference	95% CI	SE	t(33)	p
NotO.NotX	NotO.X	Left	-0.05	[-0.60, 0.51]	0.20	-0.25	0.807
NotO.NotX	O.NotX	Left	-1.53	[-2.26, -0.80]	0.26	-5.86	< .001
NotO.NotX	OX	Left	-2.77	[-3.67, -1.88]	0.32	-8.71	< .001
NotO.X	O.NotX	Left	-1.48	[-2.24, -0.72]	0.27	-5.48	< .001
NotO.X	OX	Left	-2.72	[-3.46, -1.99]	0.26	-10.39	< .001
O.NotX	OX	Left	-1.25	[-2.09, -0.40]	0.30	-4.15	< .001

Marginal contrasts estimated at Condition

p-value adjustment method: Holm (1979)

Condition	Laterality	Mean	SE	95% CI
NotO.NotX	Left	1.08	0.14	[0.81, 1.36]
NotO.X	Left	1.13	0.20	[0.73, 1.53]
O.NotX	Left	2.61	0.27	[2.05, 3.17]
OX	Left	3.86	0.34	[3.16, 4.55]

Marginal means estimated at Condition, Laterality

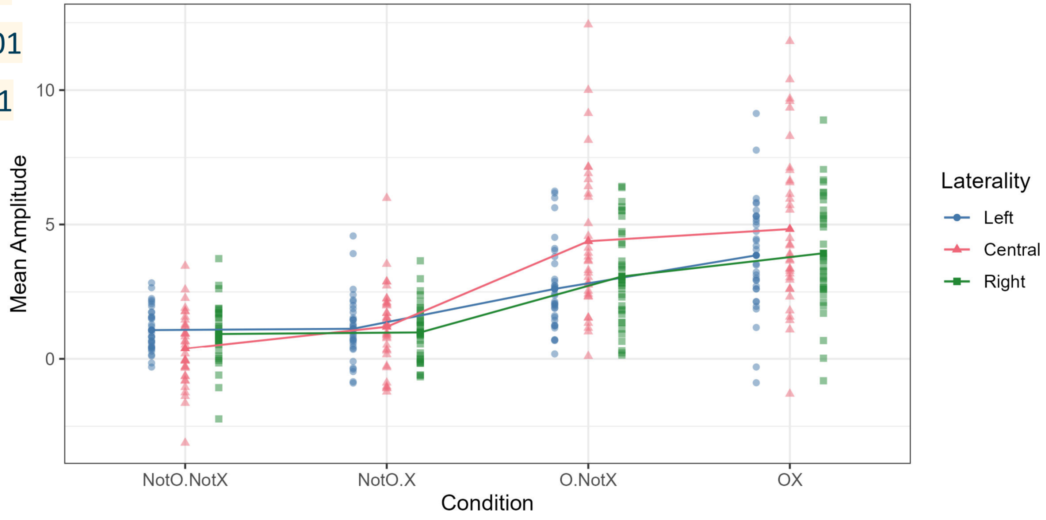

ERP Analysis (Letter-CPT)

- **Continued P300** (300-400 ms)

Following up on the interaction of Condition:Site F(3, 99) = 52.440, p < .001, np2 = .614

On the central site:

Level1	Level2	Site	Difference	95% CI	SE	t(33)	p
NotO.NotX	NotO.X	C3.Cz.C4	-0.06	[-0.58, 0.47]	0.19	-0.31	0.758
NotO.NotX	O.NotX	C3.Cz.C4	-3.81	[-4.84, -2.78]	0.37	-10.38	< .001
NotO.NotX	OX	C3.Cz.C4	-2.97	[-4.13, -1.82]	0.41	-7.21	< .001
NotO.X	O.NotX	C3.Cz.C4	-3.75	[-4.87, -2.62]	0.40	-9.34	< .001
NotO.X	OX	C3.Cz.C4	-2.91	[-4.00, -1.83]	0.39	-7.51	< .001
O.NotX	OX	C3.Cz.C4	0.83	[-0.42, 2.08]	0.45	1.87	0.140

Marginal contrasts estimated at Condition

p-value adjustment method: Holm (1979)

Estimated Marginal Means

Condition	Site	Mean	SE	95% CI
NotO.NotX	C3.Cz.C4	0.10	0.16	[-0.23, 0.43]
NotO.X	C3.Cz.C4	0.16	0.19	[-0.22, 0.53]
O.NotX	C3.Cz.C4	3.90	0.42	[3.05, 4.76]
OX	C3.Cz.C4	3.07	0.41	[2.23, 3.91]

Marginal means estimated at Condition, Site

ERP Analysis (Letter-CPT)

- **Continued P300**

Following up on the Interaction

Condition:Site F(3, 99) = 52.440, p < .001, np2 = .614

On the parietal site:

Level1	Level2	Site	Difference	95% CI	SE	t(33)	p
NotO.NotX	NotO.X	P3.Pz.P4	-0.56	[-1.19, 0.07]	0.22	-2.49	0.024
NotO.NotX	O.NotX	P3.Pz.P4	-1.29	[-2.07, -0.51]	0.28	-4.66	< .001
NotO.NotX	OX	P3.Pz.P4	-3.83	[-4.86, -2.80]	0.37	-10.43	< .001
NotO.X	O.NotX	P3.Pz.P4	-0.73	[-1.51, 0.04]	0.28	-2.65	0.024
NotO.X	OX	P3.Pz.P4	-3.27	[-4.20, -2.34]	0.33	-9.88	< .001
O.NotX	OX	P3.Pz.P4	-2.54	[-3.44, -1.64]	0.32	-7.92	< .001

Marginal contrasts estimated at Condition

p-value adjustment method: Holm (1979)

Estimated Marginal Means

Condition	Site	Mean	SE	95% CI
NotO.NotX	P3.Pz.P4	1.51	0.19	[1.11, 1.91]
NotO.X	P3.Pz.P4	2.07	0.25	[1.56, 2.58]
O.NotX	P3.Pz.P4	2.80	0.31	[2.17, 3.43]
OX	P3.Pz.P4	5.34	0.41	[4.50, 6.19]

Marginal means estimated at Condition, Site

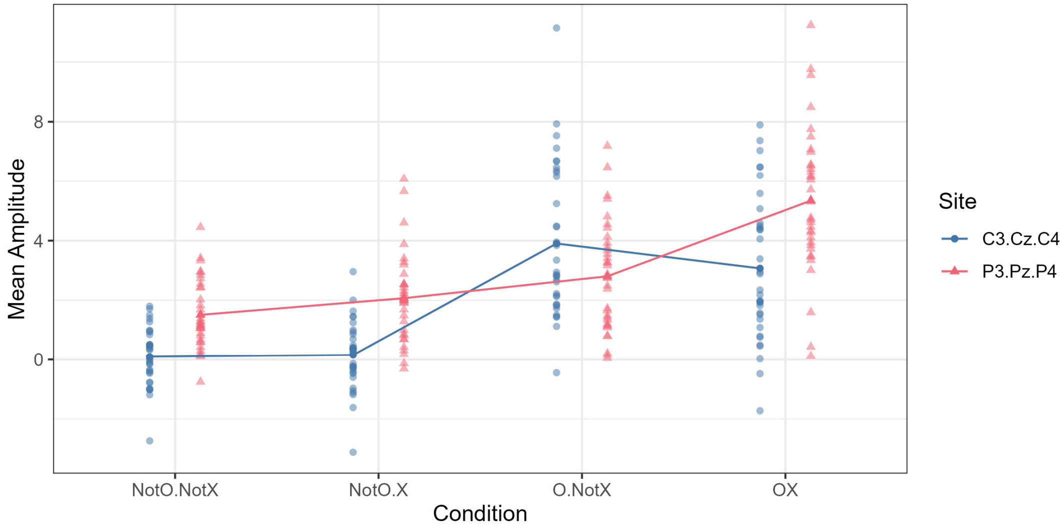

ERP Analysis (Letter-CPT)

CNV: (1700-2000) only Cz

- In the plot of our ERP grand averages, we saw that only two conditions caused a CNV component, and thus we keep only these two conditions: OX, O,NotX, and **the results show NO EFFECT!**
- 2*2 within and between design. Factors: Condition Type (S1=O, S1≠O), Group (ASD, Neurotypical). The studied electrode is Cz. *some papers have used Cz, CPz and Pz as electrode sites.

  - **Group F(1, 33) = 0.438, p = .513, np2 = .013**
  - **condtype F(1, 33) = 151.936, p < .001, np2 = .822 (has two levels, First-O (*Mean*=-1.602) versus First-NotO (*Mean*=0.304))**
  - **Group:condtype F(1, 33) = 0.824, p = .370, np2 = .024**

Estimated Marginal Means

Group	Mean	SE	95% CI
ASD	-0.55	0.21	[-0.98, -0.12]
TD	-0.74	0.20	[-1.16, -0.33]

Marginal means estimated at Group

Estimated Marginal Means

condtype	Mean	SE	95% CI
FirstO	-1.60	0.20	[-2.01, -1.19]
NFirstO	0.30	0.12	[0.06, 0.55]

Marginal means estimated at Group

ERP Analysis (Face-CPT)

- **P100:**(100-140 ms)
- 4*2*2 within and between design. Factors: Condition (OX, NotOX, ONotX, NotONotX), Electrode (O1, O2), Group (ASD, Neurotypical)
  - **Main effect of Condition** **F(3, 99) = 18.724, p < .001, np2 = .362**
  - **Interaction of Condition: Electrode** **F(3, 99) = 5.500, p = .002, np2 = .143**
- **Following up on the main effect of condition**

Level1	Level2	Difference	95% CI	SE	t(33)	p	d	95% CI
NotO.NotX	NotO.X	0.19	[-0.25, 0.64]	0.16	1.23	0.226	0.21	[-0.13, 0.56]
NotO.NotX	O.NotX	-1.11	[-1.69, -0.54]	0.20	-5.45	< .001	-0.95	[-1.36, -0.53]
NotO.NotX	OX	-0.61	[-1.18, -0.04]	0.20	-3.00	0.015	-0.52	[-0.88, -0.15]
NotO.X	O.NotX	-1.31	[-1.89, -0.73]	0.21	-6.34	< .001	-1.10	[-1.53, -0.67]
NotO.X	OX	-0.80	[-1.29, -0.32]	0.17	-4.64	< .001	-0.81	[-1.20, -0.41]
O.NotX	OX	0.50	[-0.11, 1.11]	0.22	2.32	0.053	0.40	[0.05, 0.76]

- Marginal contrasts estimated at Condition
- p-value adjustment method: Holm (1979)

Condition	Mean	SE	95% CI
NotO.NotX	5.64	0.51	[4.61, 6.67]
NotO.X	5.45	0.51	[4.42, 6.47]
O.NotX	6.75	0.47	[5.81, 7.70]
OX	6.25	0.53	[5.17, 7.33]

Marginal means estimated at Condition

***In contrast to letter P100, Face P100 is enhanced when the first stimulus is O**

ERP Analysis (Face-CPT)

- **Continued P100:**(100-140 ms)
  - **Following up on the interaction of Condition: Electrode $F(3, 99) = 5.500$, $p = .002$, $np2 = .143$**

If the electrode O1 is constant:

Level1	Level2	electrode	Difference	95% CI	SE	t(33)	p
NotO.NotX	NotO.X	O1	0.45	[-0.03, 0.93]	0.17	2.63	0.026
NotO.NotX	O.NotX	O1	-1.03	[-1.58, -0.47]	0.20	-5.16	< .001
NotO.NotX	OX	O1	-0.31	[-0.93, 0.31]	0.22	-1.41	0.167
NotO.X	O.NotX	O1	-1.48	[-2.08, -0.88]	0.21	-6.92	< .001
NotO.X	OX	O1	-0.76	[-1.24, -0.28]	0.17	-4.45	< .001
O.NotX	OX	O1	0.72	[0.06, 1.37]	0.23	3.05	0.014

- Marginal contrasts estimated at Condition
- p-value adjustment method: Holm (1979)

Condition	electrode	Mean	SE	95% CI
NotO.NotX	O1	5.60	0.50	[4.58, 6.62]
NotO.X	O1	5.15	0.51	[4.10, 6.19]
O.NotX	O1	6.62	0.47	[5.67, 7.58]
OX	O1	5.91	0.53	[4.83, 6.99]

Marginal means estimated at electrode O1

ERP Analysis (Face-CPT)

- **Continued P100:**(100-140 ms)
  - **Following up on the interaction of Condition: Electrode $F(3, 99) = 5.500$, $p = .002$, $np2 = .143$**

  If the data is aggregated on the electrode O2:

Level1	Level2	electrode	Difference	95% CI	SE	t(33)	p
NotO.NotX	NotO.X	O2	-0.06	[-0.54, 0.42]	0.17	-0.37	0.712
NotO.NotX	O.NotX	O2	-1.20	[-1.88, -0.52]	0.24	-4.97	< .001
NotO.NotX	OX	O2	-0.91	[-1.57, -0.25]	0.23	-3.87	0.002
NotO.X	O.NotX	O2	-1.14	[-1.79, -0.48]	0.23	-4.89	< .001
NotO.X	OX	O2	-0.84	[-1.44, -0.24]	0.21	-3.95	0.002
O.NotX	OX	O2	0.29	[-0.35, 0.93]	0.23	1.28	0.416

  - Marginal contrasts estimated at Condition
  - p-value adjustment method: Holm (1979)

Condition	electrode	Mean	SE	95% CI
NotO.NotX	O2	5.68	0.55	[4.57, 6.80]
NotO.X	O2	5.75	0.54	[4.66, 6.84]
O.NotX	O2	6.88	0.51	[5.84, 7.92]
OX	O2	6.59	0.58	[5.41, 7.77]

Marginal means estimated at electrode O2

- Please find the interaction plot on the next slide:

ERP Analysis (Face-CPT)

- **Continued P100:**(100-140 ms)

  - Following up on the interaction of Condition: Electrode $F(3, 99) = 5.500$, $p = .002$, $np2 = .143$

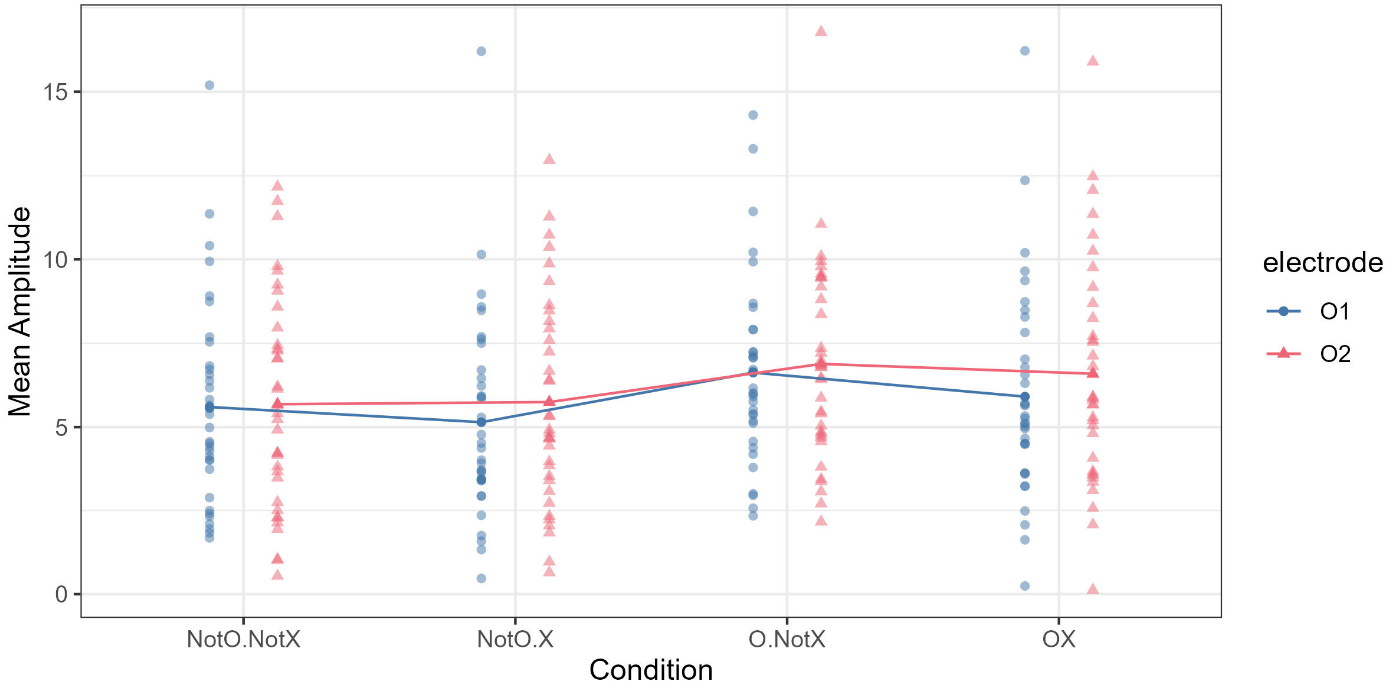

ERP Analysis (Face-CPT)

- **N170:** (140-190 ms)
- 4*4*2*2 within and between design. Factors: Condition (OX, NotOX, ONotX, NotONotX), Electrode site (P7/P8, P9/P10, PO7/PO8, PO9/PO10), Hemisphere (Right, Left), Group (ASD, Neurotypical)
  - **Main effect of Condition F(3, 99) = 6.933, p < .001, np2 = .174**
  - **Main effect of Site F(3, 99) = 49.002, p < .001, np2 = .598**
  - **The interaction of Group:Site F(3, 99) = 4.132, p = .014, np2 = .111**
  - **The interaction of Condition:Site F(9, 297) = 3.326, p = .006, np2 = .092**
- Following up on the main effect of condition:

Level1	Level2	Difference	95% CI	SE	t(33)	p	d	95% CI
NotO.NotX	NotO.X	0.32	[-0.10, 0.75]	0.15	2.14	0.120	0.37	[0.02, 0.72]
NotO.NotX	O.NotX	0.28	[-0.20, 0.77]	0.17	1.63	0.225	0.28	[-0.07, 0.63]
NotO.NotX	OX	0.69	[0.20, 1.17]	0.17	3.97	0.002	0.69	[0.31, 1.07]
NotO.X	O.NotX	-0.04	[-0.37, 0.28]	0.12	-0.35	0.727	-0.06	[-0.40, 0.28]
NotO.X	OX	0.36	[-0.05, 0.78]	0.15	2.44	0.081	0.42	[0.07, 0.78]
O.NotX	OX	0.40	[0.02, 0.79]	0.14	2.94	0.030	0.51	[0.15, 0.87]

- Marginal contrasts estimated at Condition
- p-value adjustment method: Holm (1979) *** Seems mostly enhanced in target, OX, condition. In contrast to N170 in letter CPT, in faces N170 is less enhanced during O,NotX condition.**

Condition	Mean	SE	95% CI
NotO.NotX	1.69	0.41	[0.85, 2.53]
NotO.X	1.36	0.36	[0.64, 2.09]
O.NotX	1.40	0.38	[0.63, 2.17]
OX	1.00	0.37	[0.26, 1.74]

Marginal means estimated at Condition

ERP Analysis (Face-CPT)

- **Continued N170:** (140-190 ms)
- Following up on the main effect of Site:
- **Main effect of Site F(3, 99) = 49.002, p < .001, np2 = .598**

Level1	Level2	Difference	95% CI	SE	t(33)	p	d	95% CI
P7.P8	P9.P10	0.26	[-0.27, 0.80]	0.19	1.37	0.179	0.24	[-0.11, 0.58]
P7.P8	PO7.PO8	-2.03	[-2.55, -1.51]	0.18	-10.99	< .001	-1.91	[-2.48, -1.34]
P9.P10	PO7.PO8	-2.29	[-3.03, -1.56]	0.26	-8.75	< .001	-1.52	[-2.02, -1.02]
PO9.PO10	P7.P8	0.98	[0.33, 1.63]	0.23	4.23	< .001	0.74	[0.35, 1.12]
PO9.PO10	P9.P10	1.24	[0.77, 1.72]	0.17	7.31	< .001	1.27	[0.81, 1.73]
PO9.PO10	PO7.PO8	-1.05	[-1.64, -0.46]	0.21	-4.98	< .001	-0.87	[-1.26, -0.46]

- Marginal contrasts estimated at Condition
- p-value adjustment method: Holm (1979)
- ***As expected we observe the most enhanced N170 on P9/P10 sites**

Site	Mean	SE	95% CI
PO9.PO10	1.66	0.43	[0.78, 2.54]
P7.P8	0.68	0.32	[0.03, 1.33]
P9.P10	0.41	0.38	[-0.37, 1.19]
PO7.PO8	2.71	0.41	[1.86, 3.55]

Marginal means estimated at Site

ERP Analysis (Face-CPT)

- **Continued N170:** (140-190 ms)
- Following up on the Interaction of Group: Site: **Group:Site F(3, 99) = 4.132, p = .014, np2 = .111**
- If the site is considered constant, then for every site, the difference between ASD and TD are:

Level1	Level2	Site	Difference	95% CI	SE	t(33)	p
ASD	TD	P7.P8	0.54	[-0.76, 1.85]	0.64	0.85	0.401
ASD	TD	P9.P10	-0.30	[-1.86, 1.26]	0.77	-0.39	0.701
ASD	TD	PO7.PO8	1.16	[-0.52, 2.84]	0.83	1.40	0.170
ASD	TD	PO9.PO10	0.27	[-1.48, 2.03]	0.86	0.32	0.752

- Marginal contrasts estimated at Group
- p-value adjustment method: Holm (1979)

Group	Site	Mean	SE	95% CI
ASD	P7.P8	0.95	0.46	[0.02, 1.88]
TD	P7.P8	0.40	0.45	[-0.50, 1.31]
ASD	P9.P10	0.27	0.55	[-0.85, 1.39]
TD	P9.P10	0.56	0.53	[-0.53, 1.65]
ASD	PO7.PO8	3.29	0.59	[2.08, 4.49]
TD	PO7.PO8	2.13	0.58	[0.95, 3.30]
ASD	PO9.PO10	1.79	0.62	[0.53, 3.05]
TD	PO9.PO10	1.52	0.60	[0.29, 2.74]

Marginal means estimated at Group, Site

- ***Our second analysis following up on each site reveals no significant difference between ASD and TD on any specific sites. However, the interaction plot (next slide) shows that for ASD individuals, N170 was mostly enhanced at P9/P10, but for TD, it was mostly enhanced at P7/P8, though not significant.**

Level1	Level2	Group	Difference	95% CI	SE	t(33)	p
P7.P8	P9.P10	ASD	0.68	[-0.09, 1.45]	0.27	2.49	0.032
P7.P8	P9.P10	TD	-0.16	[-0.91, 0.59]	0.27	-0.59	0.556

ERP Analysis (Face-CPT)

- **Continued N170:** (140-190 ms)
- Following up on the Interaction of Group: Site: **Group:Site F(3, 99) = 4.132, p = .014, np2 = .111**
- ***Our second analysis following up on each site reveals no significant difference between ASD and TD on any specific sites. However, the interaction plot shows that for ASD individuals, N170 was mostly enhanced at P9/P10, but for TD, it was mostly enhanced at P7/P8, though not significant. Also, the comparison between PO9/PO10 & Po7/PO8 was significant in ASD but not in TD!**

Level1	Level2	Group	Difference	95% CI	SE	t(33)	p
P7.P8	P9.P10	ASD	0.68	[-0.09, 1.45]	0.27	2.49	0.032
P7.P8	P9.P10	TD	-0.16	[-0.91, 0.59]	0.27	-0.59	0.556
PO7.PO8	PO9.PO10	ASD	1.49	[0.64, 2.34]	0.30	4.94	< .001
PO7.PO8	PO9.PO10	TD	0.61	[-0.22, 1.43]	0.29	2.07	0.094

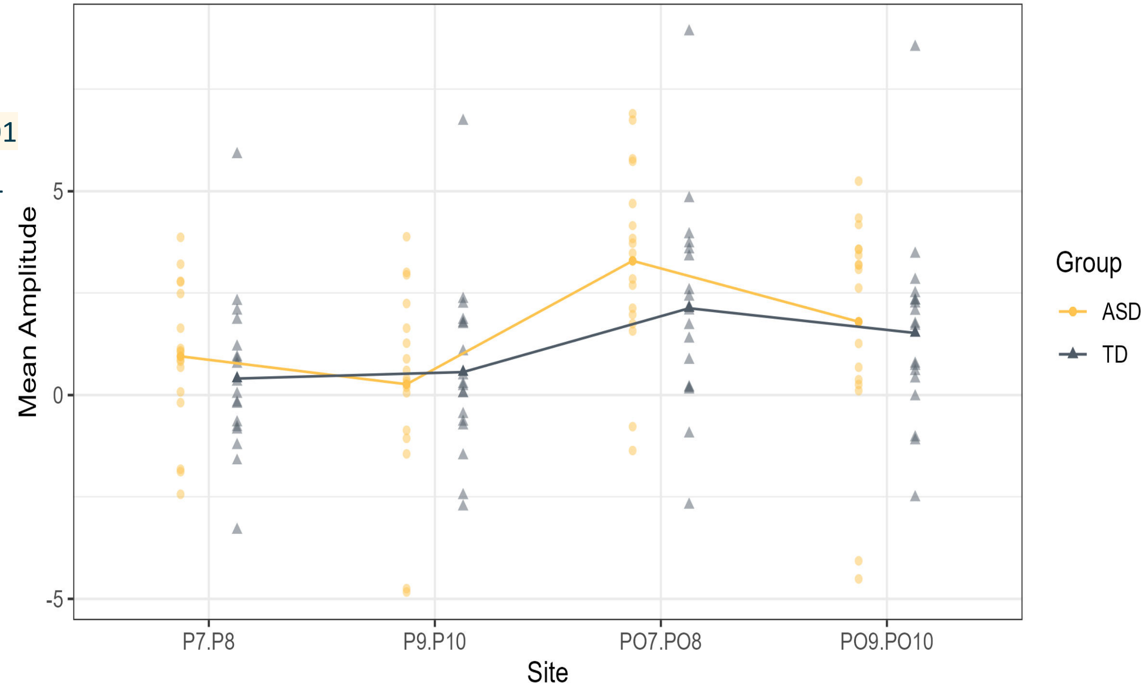

ERP Analysis (Face-CPT)

- **Continued N170:** (140-190 ms)
- Following up on the Interaction of **Condition:Site $F(9, 297) = 3.326$, $p = .006$, np2 = .092**
- If the site is considered constant, then for P7/P8:

Level1	Level2	Site	Difference	95% CI	SE	t(33)	p
NotO.NotX	NotO.X	P7.P8	0.11	[-0.28, 0.49]	0.14	0.78	0.439
NotO.NotX	O.NotX	P7.P8	0.39	[-0.01, 0.78]	0.14	2.71	0.042
NotO.NotX	OX	P7.P8	0.69	[0.26, 1.12]	0.15	4.47	< .001
NotO.X	O.NotX	P7.P8	0.28	[-0.05, 0.61]	0.12	2.37	0.050
NotO.X	OX	P7.P8	0.58	[0.20, 0.96]	0.13	4.30	< .001
O.NotX	OX	P7.P8	0.30	[-0.03, 0.64]	0.12	2.52	0.050

Condition	Site	Mean	SE	95% CI
NotO.NotX	P7.P8	0.97	0.36	[0.23, 1.71]
NotO.X	P7.P8	0.86	0.31	[0.24, 1.49]
O.NotX	P7.P8	0.59	0.33	[-0.08, 1.25]
OX	P7.P8	0.28	0.32	[-0.37, 0.94]

- Marginal contrasts estimated at Condition
- p-value adjustment method: Holm (1979)

ERP Analysis (Face-CPT)

- **Continued N170:** (140-190 ms)
- Following up on the Interaction of Condition Site: **Condition:Site F(9, 297) = 3.326, p = .006, np2 = .092**
- If the site is considered constant, then for P9/P10:
- Level1 | Level2 | Site | Difference | 95% CI | SE | t(33) | p
- --
- NotO.NotX | NotO.X | P9.P10 |0.52 | [-0.08, 1.11] | 0.21 | 2.44 | 0.101
- NotO.NotX | O.NotX | P9.P10 |0.32 | [-0.33, 0.96] | 0.23 | 1.37 | 0.540
- NotO.NotX | OX | P9.P10 |0.76 | [0.13, 1.39] | 0.22 | 3.41 | 0.010
- NotO.X | O.NotX | P9.P10 |-0.20 | [-0.63, 0.23] | 0.15 | -1.32 | 0.540
- NotO.X | OX | P9.P10 | 0.25 | [-0.29, 0.79] | 0.19 | 1.29 | 0.540
- O.NotX | OX | P9.P10 | 0.45 | [-0.07, 0.97] | 0.18 | 2.43 | 0.101

- Marginal contrasts estimated at Condition
- p-value adjustment method: Holm (1979)

Condition	Site	Mean	SE	95% CI
NotO.NotX	P9.P10	0.81	0.43	[-0.07, 1.69]
NotO.X	P9.P10	0.30	0.37	[-0.45, 1.05]
O.NotX	P9.P10	0.50	0.42	[-0.36, 1.35]
OX	P9.P10	0.05	0.39	[-0.74, 0.84]

ERP Analysis (Face-CPT)

- **Continued N170:** (140-190 ms)
- Following up on the Interaction of Condition Site: **Condition:Site F(9, 297) = 3.326, p = .006, np2 = .092**
- If the site is considered constant, then for PO7/PO8, the mean values are very close, and thus no significant effect of condition at this site is observed.

Condition	Site	Mean	SE	95% CI
NotO.NotX	PO7.PO8	2.88	0.46	[1.94, 3.81]
NotO.X	PO7.PO8	2.79	0.42	[1.94, 3.63]
O.NotX	PO7.PO8	2.70	0.41	[1.87, 3.52]
OX	PO7.PO8	2.46	0.42	[1.60, 3.32]

ERP Analysis (Face-CPT)

- **Continued N170:** (140-190 ms)
- Following up on the Interaction of Condition Site:
- If the site is considered constant, then for PO9/PO10: **Condition:Site F(9, 297) = 3.326, p = .006, np2 = .092**
- Level1 | Level2 | Site | Difference | 95% CI | SE | t(33) | p
- --
- NotO.NotX | NotO.X | PO9.PO10 | 0.58 | [0.05, 1.11] | 0.19 | 3.05 | 0.018
- NotO.NotX | O.NotX | PO9.PO10 | 0.25 | [-0.40, 0.90] | 0.23 | 1.08 | 0.288
- NotO.NotX | OX | PO9.PO10 | 0.88 | [0.26, 1.50] | 0.22 | 3.98 | 0.002
- NotO.X | O.NotX | PO9.PO10 | -0.33 | [-0.75, 0.10] | 0.15 | -2.17 | 0.112
- NotO.X | OX | PO9.PO10 | 0.30 | [-0.21, 0.81] | 0.18 | 1.65 | 0.217
- O.NotX | OX | PO9.PO10 | 0.63 | [0.09, 1.17] | 0.19 | 3.27 | 0.012

Condition	Site	Mean	SE	95% CI
NotO.NotX	PO9.PO10	2.08	0.48	[1.10, 3.06]
NotO.X	PO9.PO10	1.50	0.44	[0.61, 2.40]
O.NotX	PO9.PO10	1.83	0.45	[0.91, 2.76]
OX	PO9.PO10	1.21	0.42	[0.36, 2.05]

- Marginal contrasts estimated at Condition
- p-value adjustment method: Holm (1979)
- Interaction plot(next slide)

ERP Analysis (Face-CPT)

- **Continued N170:** (140-190 ms)
- Following up on the Interaction of Condition :Site:
- **Condition:Site F(9, 297) = 3.326, p = .006, np2 = .092**
- **Interaction plot**

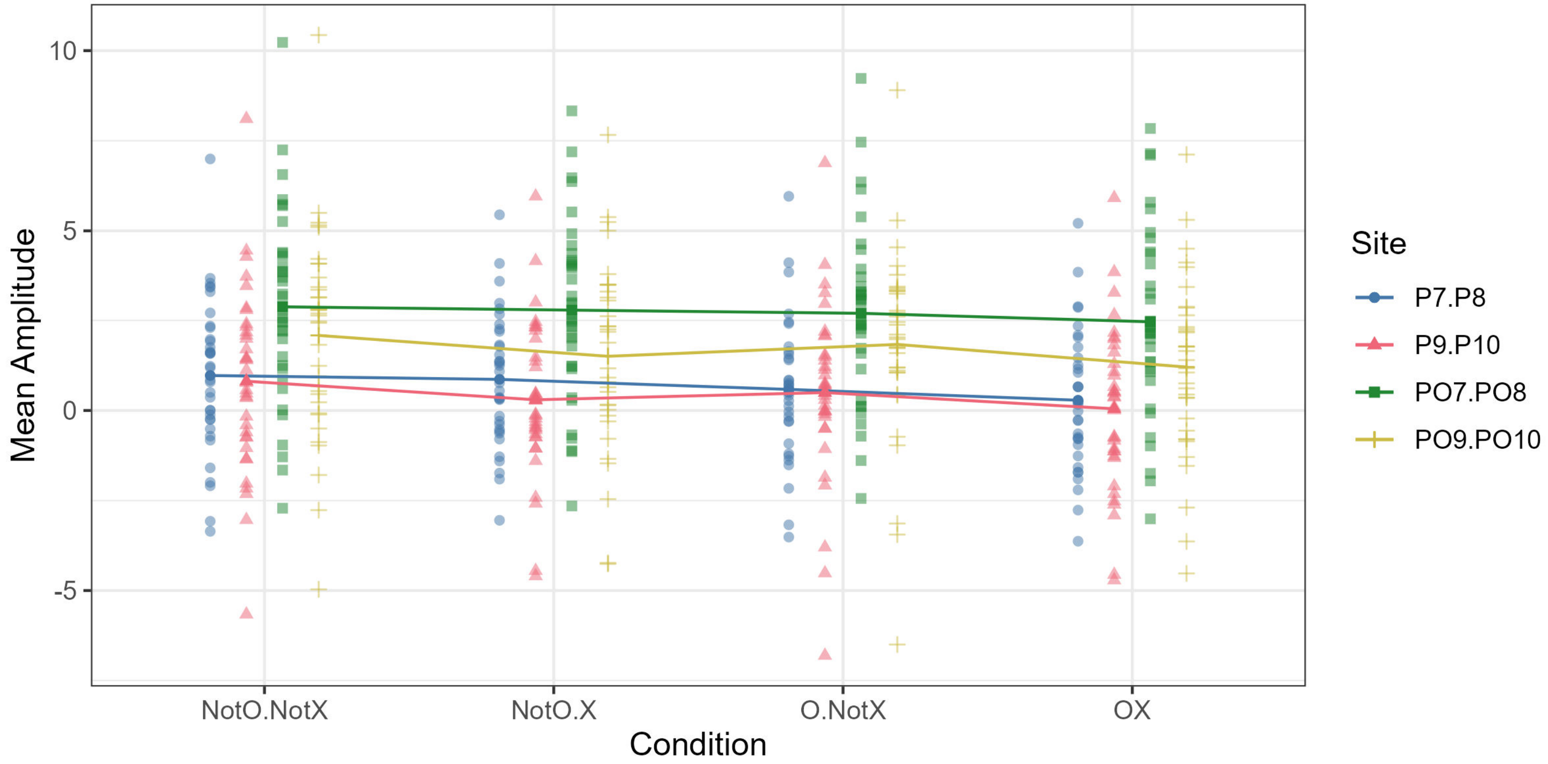

ERP Analysis (Face-CPT)

- **P200:** (190-240)

4*2*2*2 within and between design. Factors: Condition (OX, NotOX, ONotX, NotONotX), Electrode (P7/P8, PO7/PO8), Hemisphere (Right, Left), Group (ASD, Neurotypical)

- **Group F(1, 33) = 0.081, p = .778, np2 < .012**
- **Condition F(3, 99) = 6.094, p = .002, np2 = .156**
- **Hemisphere F(1, 33) = 3.671, p = .064, np2 = .100**
- **Site F(1, 33) = 118.504, p < .001, np2 = .782**
- **Group:Hemisphere F(1, 33) = 4.550, p = .040, np2 = .121**
- **Condition:Site F(3, 99) = 9.593, p < .001, np2 = .225**
- **Condition:Hemisphere:Site F(3, 99) = 2.900, p = .039, np2 = .081**

The trend effect of the Group:

Group	Mean	SE	95% CI
ASD	2.48	0.56	[1.35, 3.61]
TD	2.70	0.54	[1.60, 3.80]

Marginal means estimated at Group

ERP Analysis (Face-CPT)

- **Continued P200:** (190-240)
- Following up on the main effect of Condition
  - **Condition F(3, 99) = 6.094, p = .002, np2 = .156**

Level1	Level2	Difference	95% CI	SE	t(33)	p	d	95% CI
NotO.NotX	NotO.X	0.10	[-0.38, 0.58]	0.17	0.60	> .999	0.10	[-0.24, 0.45]
NotO.NotX	O.NotX	-0.57	[-0.99, -0.15]	0.15	-3.83	0.003	-0.67	[-1.04, -0.29]
NotO.NotX	OX	0.05	[-0.44, 0.53]	0.17	0.28	> .999	0.05	[-0.29, 0.39]
NotO.X	O.NotX	-0.67	[-1.29, -0.06]	0.22	-3.07	0.017	-0.53	[-0.90, -0.17]
NotO.X	OX	-0.05	[-0.64, 0.53]	0.21	-0.26	> .999	-0.05	[-0.39, 0.30]
O.NotX	OX	0.62	[0.22, 1.02]	0.14	4.35	< .001	0.76	[0.37, 1.14]

Condition	Mean	SE	95% CI
NotO.NotX	2.49	0.41	[1.64, 3.33]
NotO.X	2.38	0.41	[1.55, 3.21]
O.NotX	3.06	0.42	[2.20, 3.92]
OX	2.44	0.36	[1.70, 3.18]

Marginal means estimated at Condition

ERP Analysis (Face-CPT)

- **Continued P200:** (190-240)
- Following up on the main effect of Site:
  - **Site F(1, 33) = 118.504, p < .001, np2 = .782**
- Site for P200 has only two level; so we look at the means:

Site	Mean	SE	95% CI
P7.P8	1.44	0.35	[0.72, 2.17]
PO7.PO8	3.74	0.44	[2.83, 4.64]

Marginal means estimated at Site

- ***Same as the letter CPT, P200 has its highest amplitude at PO7/PO8 sites.**

ERP Analysis (Face-CPT)

- **Continued P200:** (190-240)
- Following up on the interaction of the Group: Hemisphere
  - **Group:Hemisphere F(1, 33) = 4.550, p = .040, np2 = .121**
- If the hemisphere is kept constant, then:
- Level1 | Level2 | Hemisphere | Difference |95% CI | SE | t(33) | p | d | 95% CI
- --
- ASD | TD | Left | -1.22 | [-2.70, 0.26] | 0.73 | -1.68 | 0.102 |0.59 | [-1.28, 0.12]
- ASD | TD | Right | 0.78 | [-1.37, 2.93] | 1.06 | 0.74 | 0.466 |0.26 | [-0.43, 0.94]
- Marginal contrasts estimated at Condition
- p-value adjustment method: Holm (1979)
- However, if we keep the group constant:
- Level1 | Level2 | Group | Difference | 95% CI | SE | t(33) | p | d | 95% CI
- ---
- Left | Right | ASD | -1.90 | [-3.27, -0.53] | 0.67 | -2.82 | 0.008 |0.98 | [-1.70, -0.25]
- Left | Right | TD | 0.10 | [-1.23, 1.43] | 0.65 | 0.16 | 0.877 |0.05 | [-0.63, 0.74]

Group	Hemisphere	Mean	SE	95% CI
ASD	Left	1.53	0.52	[0.47, 2.59]
TD	Left	2.75	0.51	[1.72, 3.78]
ASD	Right	3.43	0.76	[1.89, 4.97]
TD	Right	2.65	0.74	[1.15, 4.15]

Marginal means estimated at Group, Hemisphere

- **In ASD, there is an asymmetry between the right and left hemispheres in P2, which, in TD, does not exist! See the interaction plot on the next slide**

ERP Analysis (Face-CPT)

- **Continued P200:** (190-240)
- Following up on the interaction of the Group: Hemisphere
  - **Group:Hemisphere F(1, 33) = 4.550, p = .040, np2 = .121**
- **In ASD, there is an asymmetry between the right and left hemispheres in P2, which, in TD, does not exist! See the interaction plot on the next slide**

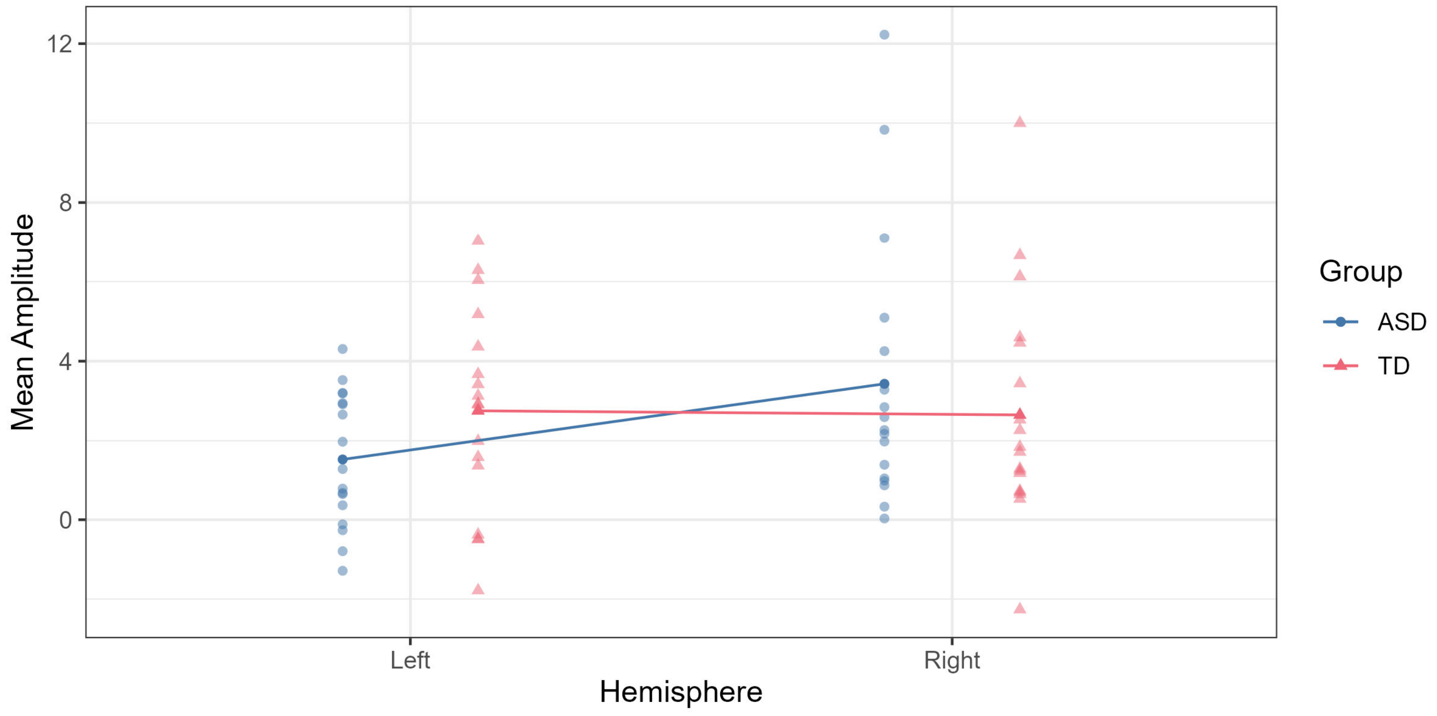

ERP Analysis (Face-CPT)

- **Continued P200:** (190-240)
- Following up on the interaction of Condition: Site
  - **Condition:Site F(3, 99) = 9.593, p < .001, np2 = .225**
- If the site is considered the constant factor, then at P7/P8:
- Level1 | Level2 | Site | Difference | 95% CI | SE | t(33) | p
- --
- NotO.NotX | NotO.X | P7.P8 | 3.02e-03 | [-0.47, 0.48] | 0.17 | 0.02 | > .999
- NotO.NotX | O.NotX | P7.P8 | -0.39 | [-0.82, 0.04] | 0.15 | -2.51 | 0.085
- NotO.NotX | OX | P7.P8 | 0.16 | [-0.33, 0.64] | 0.17 | 0.92 | > .999
- NotO.X | O.NotX | P7.P8 | -0.39 | [-1.01, 0.23] | 0.22 | -1.76 | 0.351
- NotO.X | OX | P7.P8 | 0.16 | [-0.41, 0.72] | 0.20 | 0.78 | > .999
- O.NotX | OX | P7.P8 | 0.54 | [0.14, 0.95] | 0.15 | 3.74 | 0.004

- Marginal contrasts estimated at Condition
- p-value adjustment method: Holm (1979)

Condition | Site | Mean | SE | 95% CI

NotO.NotX | P7.P8 | 1.39 | 0.39 | [0.59, 2.19]
NotO.X | P7.P8 | 1.39 | 0.36 | [0.65, 2.12]
O.NotX | P7.P8 | 1.77 | 0.40 | [0.96, 2.58]
OX | P7.P8 | 1.23 | 0.33 | [0.56, 1.90]

Marginal means estimated at Site

ERP Analysis (Face-CPT)

- **Continued P200:** (190-240)
- Following up on the interaction of Condition: Site
  - **Condition:Site F(3, 99) = 9.593, p < .001, np2 = .225**
- If the site is considered the constant factor, then at PO7/PO8:

Level1	Level2	Site	Difference	95% CI	SE	t(33)	p
NotO.NotX	NotO.X	PO7.PO8	0.20	[-0.35, 0.75]	0.20	1.03	0.764
NotO.NotX	O.NotX	PO7.PO8	-0.76	[-1.24, -0.28]	0.17	-4.45	< .001
NotO.NotX	OX	PO7.PO8	-0.06	[-0.63, 0.50]	0.20	-0.31	0.764
NotO.X	O.NotX	PO7.PO8	-0.96	[-1.61, -0.31]	0.23	-4.17	< .001
NotO.X	OX	PO7.PO8	-0.26	[-0.90, 0.37]	0.23	-1.16	0.764
O.NotX	OX	PO7.PO8	0.70	[0.28, 1.11]	0.15	4.66	< .001

- Marginal contrasts estimated at Condition
- p-value adjustment method: Holm (1979)

Condition	Site	Mean	SE	95% CI
NotO.NotX	PO7.PO8	3.58	0.46	[2.65, 4.52]
NotO.X	PO7.PO8	3.38	0.48	[2.41, 4.35]
O.NotX	PO7.PO8	4.34	0.48	[3.37, 5.31]
OX	PO7.PO8	3.65	0.43	[2.78, 4.52]

Marginal means estimated at Site

ERP Analysis (Face-CPT)

- **Continued P200:**
- Following up on the interaction of Condition: Site
  - **Condition:Site $F(3, 99) = 9.593$, $p < .001$, np2 = .225**
- **Interaction Plot**

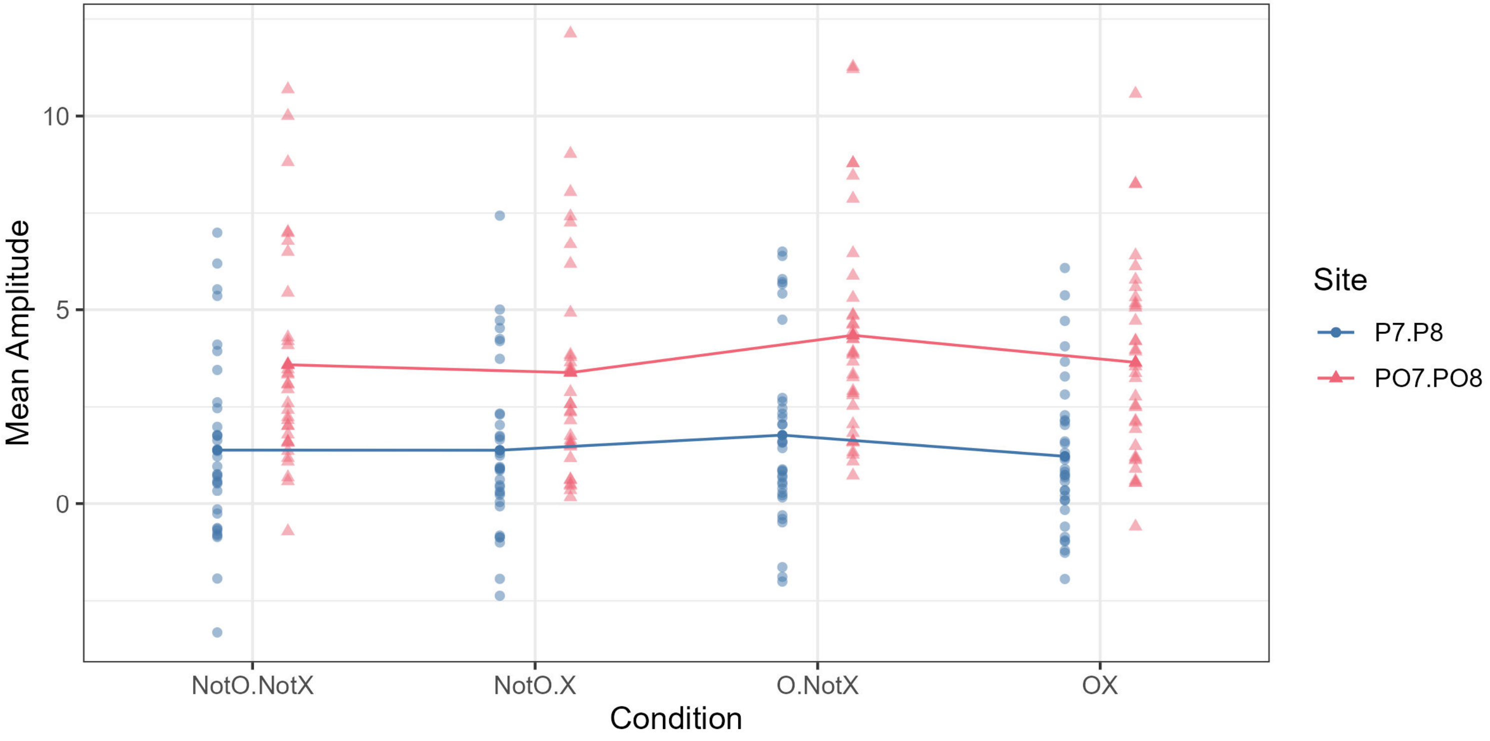

ERP Analysis (Face-CPT)

- **Continued P200:**
- Following up on the 3-way interaction of Condition:Hemisphere: Site
  - **Condition:Hemisphere:Site F(3, 99) = 2.900, p = .039, np2 = .081**
- If the Hemisphere is constant, then over the right hemisphere:
- **Condition F(3, 102) = 2.788, p = .052, np2 = .076 [0.00, 0.17]**
- **Site F(1, 34) = 79.631, p < .001, np2 = .701 [0.52, 0.80]**
- **Condition:Site F(3, 102) = 2.273, p = .085, np2 = .063 [0.00, 0.15]**
- **There is only the main effect of Site, for which we look only at the means (has 2 levels). Trend main effect of condition and trend intraction of Cond*Site that we do not follow up**

Estimated Marginal Means

Site	Mean	SE	95% CI
P7.P8	1.85	0.51	[0.81, 2.89]
PO7.PO8	4.21	0.57	[3.05, 5.36]

Marginal means estimated at Site

ERP Analysis (Face-CPT)

- **Continued P200:**
- Following up on the 3-way interaction of Condition:Hemisphere: Site
  - **Condition:Hemisphere:Site F(3, 99) = 2.900, p = .039, np2 = .081**
- If the Hemisphere is constant, then over the left hemisphere:
- **Condition F(3, 102) = 7.463, p < .001, np2 = .180 [0.05, 0.30]**
- **Site F(1, 34) = 78.253, p < .001, np2 = .697 [0.51, 0.80]**
- **Condition:Site F(3, 102) = 9.863, p < .001, np2 = .225 [0.09, 0.35]**

Following up the main effect of Site (has two levels and we require means only):

Estimated Marginal Means

Site	Mean	SE	95% CI
P7.P8	1.05	0.35	[0.34, 1.75]
PO7.PO8	3.27	0.43	[2.39, 4.15]

Marginal means estimated at Site

ERP Analysis (Face-CPT)

- **Continued P200:**
- Following up on the 3-way interaction of Condition:Hemisphere: Site
  - **Condition:Hemisphere:Site F(3, 99) = 2.900, p = .039, np2 = .081**
- If the Hemisphere is constant, then over the left hemisphere:
- **Condition F(3, 102) = 7.463, p < .001, np2 = .180 [0.05, 0.30]**

Following up on the main effect of Condition:

Level1	Level2	Difference	95% CI	SE	t(34)	p
NotO.NotX	NotO.X	0.03	[-0.41, 0.46]	0.16	0.16	> .999
NotO.NotX	O.NotX	-0.67	[-1.13, -0.22]	0.16	-4.17	0.001
NotO.NotX	OX	0.11	[-0.50, 0.72]	0.22	0.50	> .999
NotO.X	O.NotX	-0.70	[-1.23, -0.17]	0.19	-3.69	0.003
NotO.X	OX	0.08	[-0.48, 0.64]	0.20	0.42	> .999
O.NotX	OX	0.78	[0.24, 1.33]	0.19	4.03	0.001

Marginal contrasts estimated at Condition

p-value adjustment method: Holm (1979)

Condition	Mean	SE	95% CI
NotO.NotX	2.02	0.40	[1.21, 2.83]
NotO.X	2.00	0.39	[1.20, 2.80]
O.NotX	2.70	0.40	[1.89, 3.51]
OX	1.92	0.37	[1.17, 2.66]

Marginal means estimated at Condition

ERP Analysis (Face-CPT)

- **Continued P200:**
- Following up on the 3-way interaction of Condition:Hemisphere: Site
  - **Condition:Hemisphere:Site $F(3, 99) = 2.900$, $p = .039$, np2 = .081**
- If the Hemisphere is constant, then over the left hemisphere:
- **Condition:Site $F(3, 102) = 9.863$, $p < .001$, np2 = .225 [0.09, 0.35]**

Following up on the interaction of Condition: Site. At P7/P8 sites:

Level1	Level2	Site	Difference	95% CI	SE	t(34)	p
NotO.NotX	NotO.X	P7.P8	-0.06	[-0.54, 0.41]	0.17	-0.38	0.705
NotO.NotX	O.NotX	P7.P8	-0.36	[-0.86, 0.14]	0.18	-2.00	0.269
NotO.NotX	OX	P7.P8	0.27	[-0.36, 0.90]	0.22	1.21	0.466
NotO.X	O.NotX	P7.P8	-0.29	[-0.82, 0.24]	0.19	-1.55	0.390
NotO.X	OX	P7.P8	0.34	[-0.20, 0.88]	0.19	1.75	0.353
O.NotX	OX	P7.P8	0.63	[0.07, 1.19]	0.20	3.17	0.019

Marginal contrasts estimated at Condition

p-value adjustment method: Holm (1979)

Estimated Marginal Means

Condition	Site	Mean	SE	95% CI
NotO.NotX	P7.P8	1.01	0.38	[0.24, 1.78]
NotO.X	P7.P8	1.07	0.37	[0.32, 1.83]
O.NotX	P7.P8	1.37	0.37	[0.62, 2.11]
OX	P7.P8	0.74	0.36	[0.01, 1.46]

Marginal means estimated at Condition, Site

ERP Analysis (Face-CPT)

- **Continued P200:**
- Following up on the 3-way interaction of Condition:Hemisphere: Site
  - **Condition:Hemisphere:Site F(3, 99) = 2.900, p = .039, np2 = .081**
- If the Hemisphere is constant, then over the left hemisphere:
- **Condition:Site F(3, 102) = 9.863, p < .001, np2 = .225 [0.09, 0.35]**

Following up on the interaction of Condition: Site. At PO7/PO8 sites:

Level1	Level2	Site	Difference	95% CI	SE	t(34)	p
NotO.NotX	NotO.X	PO7.PO8	0.12	[-0.39, 0.62]	0.18	0.64	> .999
NotO.NotX	O.NotX	PO7.PO8	-0.99	[-1.50, -0.48]	0.18	-5.46	< .001
NotO.NotX	OX	PO7.PO8	-0.06	[-0.76, 0.65]	0.25	-0.22	> .999
NotO.X	O.NotX	PO7.PO8	-1.11	[-1.73, -0.49]	0.22	-5.02	< .001
NotO.X	OX	PO7.PO8	-0.17	[-0.84, 0.50]	0.24	-0.72	> .999
O.NotX	OX	PO7.PO8	0.94	[0.34, 1.53]	0.21	4.41	< .001

Marginal contrasts estimated at Condition

p-value adjustment method: Holm (1979)

Estimated Marginal Means

Condition	Site	Mean	SE	95% CI
NotO.NotX	PO7.PO8	3.04	0.46	[2.11, 3.97]
NotO.X	PO7.PO8	2.92	0.45	[2.01, 3.84]
O.NotX	PO7.PO8	4.03	0.47	[3.07, 4.99]
OX	PO7.PO8	3.09	0.43	[2.22, 3.97]

Marginal means estimated at Condition, Site

ERP Analysis (Face-CPT)

- **Continued P200:**
- Following up on the 3-way interaction of Condition:Hemisphere: Site
  - **Condition:Hemisphere:Site F(3, 99) = 2.900, p = .039, np2 = .081**
- Interaction plot:

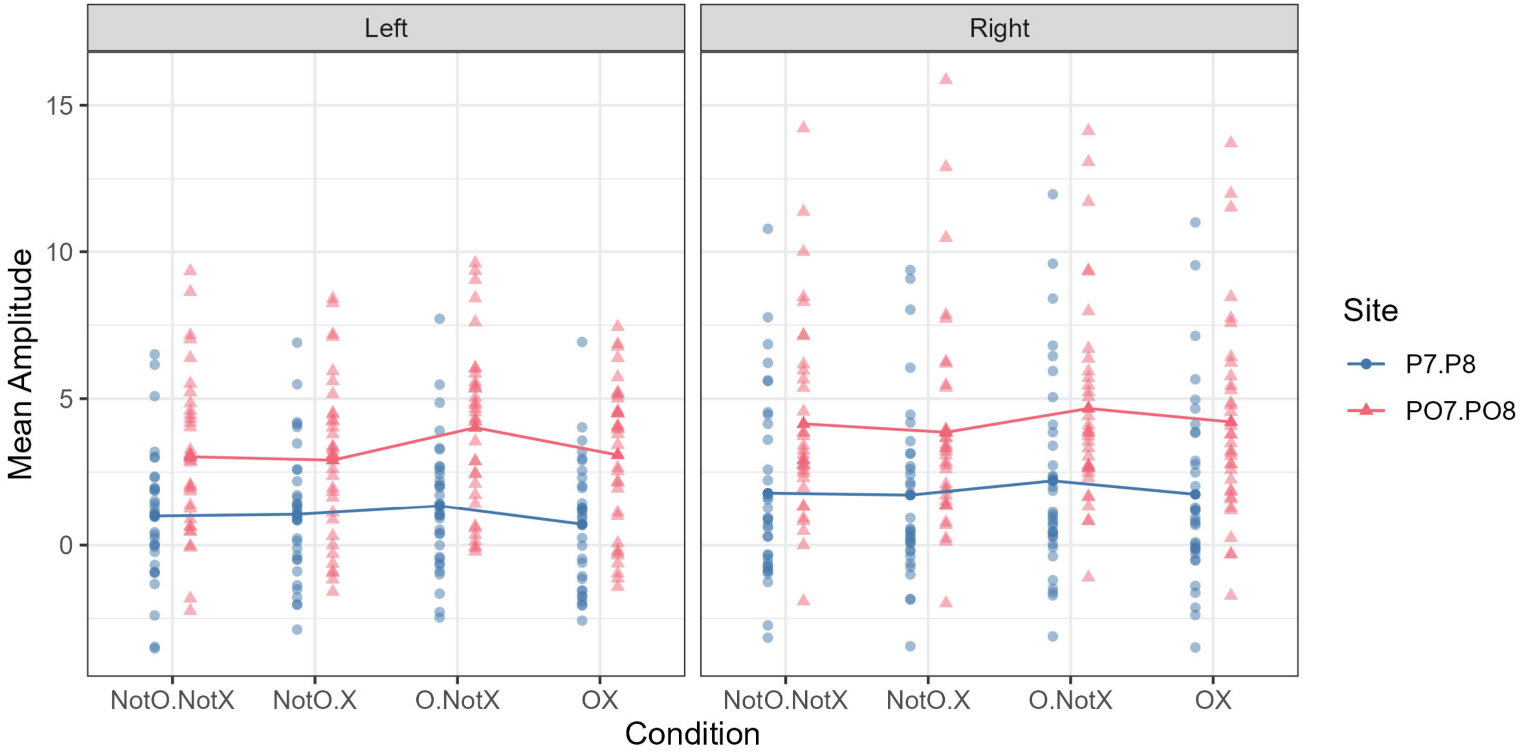

ERP Analysis (Face-CPT)

- **N250** (260-340)
- 4*5*2*2 within and between design. Factors: Condition (OX, NotOX, ONotX, NotONotX), Electrode (P7/P8, P9/P10, PO7/PO8, PO9/PO10, TP9, TP10), Hemisphere (Right, Left), Group (ASD, Neurotypical)

  - **Condition $F(3, 99) = 22.400$, $p < .001$, np2 = .404**
  - **Hemisphere $F(1, 33) = 4.857$, $p = .035$, np2 = .128**
  - **Site $F(4, 132) = 125.550$, $p < .001$, np2 = .792**
  - **Condition:Hemisphere $F(3, 99) = 3.321$, $p = .033$, np2 = .091**
  - **Condition:Site $F(12, 396) = 10.380$, $p < .001$, np2 = .239**
  - **Group:Condition:Hemisphere $F(3, 99) = 0.042$, $p = .976$, np2 < .011**
  - **Group:Hemisphere:Site $F(4, 132) = 2.106$, $p = .084$, np2 = .060**

ERP Analysis (Face-CPT)

- **Continued N250:** (260-340)
- Following up on the main effect of Condition
    - **Condition F(3, 99) = 22.400, p < .001, np2 = .404**

Level1	Level2	Difference	95% CI	SE	t(33)	p	d	95% CI
NotO.NotX	NotO.X	0.29	[-0.19, 0.77]	0.17	1.68	0.102	0.29	[-0.06, 0.64]
NotO.NotX	O.NotX	0.87	[0.12, 1.63]	0.27	3.25	0.008	0.57	[0.19, 0.93]
NotO.NotX	OX	1.92	[1.04, 2.80]	0.31	6.14	< .001	1.07	[0.64, 1.49]
NotO.X	O.NotX	0.58	[-0.15, 1.32]	0.26	2.23	0.065	0.39	[0.03, 0.74]
NotO.X	OX	1.63	[0.86, 2.40]	0.27	5.94	< .001	1.03	[0.61, 1.45]
O.NotX	OX	1.05	[0.47, 1.62]	0.20	5.12	< .001	0.89	[0.48, 1.29]

- Marginal contrasts estimated at Condition
- p-value adjustment method: Holm (1979)
- ***Same as the letter CPT, conditions OX and O,NotX had the most enhanced N200**

Condition	Mean	SE	95% CI
NotO.NotX	0.64	0.29	[0.06, 1.22]
NotO.X	0.35	0.24	[-0.14, 0.84]
O.NotX	-0.23	0.37	[-0.98, 0.51]
OX	-1.28	0.38	[-2.04, -0.51]

Marginal means estimated at Condition

ERP Analysis (Face-CPT)

- **Continued N250:** (260-340)
- Following up on the main effect of Hemisphere
  - **Hemisphere F(1, 33) = 4.857, p = .035, np2 = .128 (Hemisphere has only two levels and requires no follow up analysis)**

Hemisphere	Mean	SE	95% CI
Left	-0.49	0.31	[-1.12, 0.15]
Right	0.23	0.34	[-0.46, 0.92]

Marginal means estimated at Hemisphere

ERP Analysis (Face-CPT)

- **Continued N250:** (260-340)
- Following up on the main effect of Site
  - **Site F(4, 132) = 125.550, p < .001, np2 = .792**

Level1	Level2	Difference	95% CI	SE	t(33)	p	d	95% CI
P7.P8	P9.P10	2.20	[1.46, 2.93]	0.24	9.03	< .001	1.57	[1.06, 2.07]
P7.P8	PO7.PO8	-1.95	[-2.57, -1.34]	0.20	-9.55	< .001	-1.66	[-2.18, -1.13]
P7.P8	PO9.PO10	1.13	[0.50, 1.76]	0.21	5.39	< .001	0.94	[0.52, 1.34]
P9.P10	PO7.PO8	-4.15	[-5.14, -3.17]	0.33	-12.67	< .001	-2.21	[-2.83, -1.57]
P9.P10	PO9.PO10	-1.07	[-1.67, -0.47]	0.20	-5.35	< .001	-0.93	[-1.34, -0.52]
PO7.PO8	PO9.PO10	3.08	[2.39, 3.78]	0.23	13.31	< .001	2.32	[1.66, 2.96]
TP9.TP10	P7.P8	-3.05	[-3.72, -2.38]	0.22	-13.71	< .001	-2.39	[-3.05, -1.71]
TP9.TP10	P9.P10	-0.86	[-1.32, -0.39]	0.16	-5.48	< .001	-0.95	[-1.36, -0.54]
TP9.TP10	PO7.PO8	-5.01	[-6.08, -3.93]	0.36	-14.04	< .001	-2.44	[-3.12, -1.76]
TP9.TP10	PO9.PO10	-1.92	[-2.68, -1.17]	0.25	-7.64	< .001	-1.33	[-1.79, -0.86]]

- p-value adjustment method: Holm (1979)
- ***As expected, N250 is mostly enhanced at TP9/TP10**

Site	Mean	SE	95% CI
TP9.TP10	-2.30	0.27	[-2.85, -1.75]
P7.P8	0.75	0.27	[0.20, 1.31]
P9.P10	-1.44	0.34	[-2.13, -0.76]
PO7.PO8	2.71	0.34	[2.01, 3.40]
PO9.PO10	-0.37	0.38	[-1.15, 0.40]

Marginal means estimated at Site

ERP Analysis (Face-CPT)

- **Continued N250:** (260-340)
- Following up on the interaction Condition: Hemisphere
  - **Condition:Hemisphere F(3, 99) = 3.321, p = .033, np2 = .091**
- If the Hemisphere factor is kept constant, then on the right side:

Level1	Level2	Hemisphere	Difference	95% CI	SE	t(33)	p
NotO.NotX	NotO.X	Right	0.12	[-0.48, 0.72]	0.21	0.57	0.575
NotO.NotX	O.NotX	Right	0.97	[0.11, 1.82]	0.30	3.18	0.012
NotO.NotX	OX	Right	1.71	[0.74, 2.68]	0.35	4.92	< .001
NotO.X	O.NotX	Right	0.85	[-0.02, 1.71]	0.31	2.74	0.020
NotO.X	OX	Right	1.59	[0.71, 2.46]	0.31	5.10	< .001
O.NotX	OX	Right	0.74	[0.09, 1.39]	0.23	3.21	0.012

- Marginal contrasts estimated at Condition
- p-value adjustment method: Holm (1979)

Condition	Hemisphere	Mean	SE	95% CI
NotO.NotX	Right	0.93	0.36	[0.20, 1.65]
NotO.X	Right	0.81	0.30	[0.19, 1.42]
O.NotX	Right	-0.04	0.43	[-0.91, 0.83]
OX	Right	-0.78	0.43	[-1.66, 0.09]

Marginal means estimated at Condition, Hemisphere

ERP Analysis (Face-CPT)

- **Continued N250:** (260-340)
- Following up on the interaction Condition: Hemisphere
  - **Condition:Hemisphere F(3, 99) = 3.321, p = .033, np2 = .091**
- If the Hemisphere factor is kept constant, then on the left side:

Level1	Level2	Hemisphere	Difference	95% CI	SE	t(33)	p
NotO.NotX	NotO.X	Left	0.46	[-0.04, 0.95]	0.18	2.60	0.028
NotO.NotX	O.NotX	Left	0.78	[0.07, 1.49]	0.25	3.06	0.013
NotO.NotX	OX	Left	2.13	[1.18, 3.08]	0.34	6.27	< .001
NotO.X	O.NotX	Left	0.32	[-0.37, 1.01]	0.24	1.31	0.200
NotO.X	OX	Left	1.67	[0.87, 2.47]	0.29	5.84	< .001
O.NotX	OX	Left	1.35	[0.67, 2.04]	0.24	5.54	< .001

- Marginal contrasts estimated at Condition
- p-value adjustment method: Holm (1979)
- **Please find the interaction plot on the next slide**

Condition	Hemisphere	Mean	SE	95% CI
NotO.NotX	Left	0.35	0.30	[-0.26, 0.97]
NotO.X	Left	-0.10	0.29	[-0.68, 0.48]
O.NotX	Left	-0.42	0.38	[-1.20, 0.35]
OX	Left	-1.77	0.41	[-2.61, -0.93]

Marginal means estimated at Condition, Hemisphere

ERP Analysis (Face-CPT)

- **Continued N250:** (260-340)
- Following up on the the interaction Condition: Hemisphere
  - **Condition:Hemisphere F(3, 99) = 3.321, p = .033, np2 = .091**

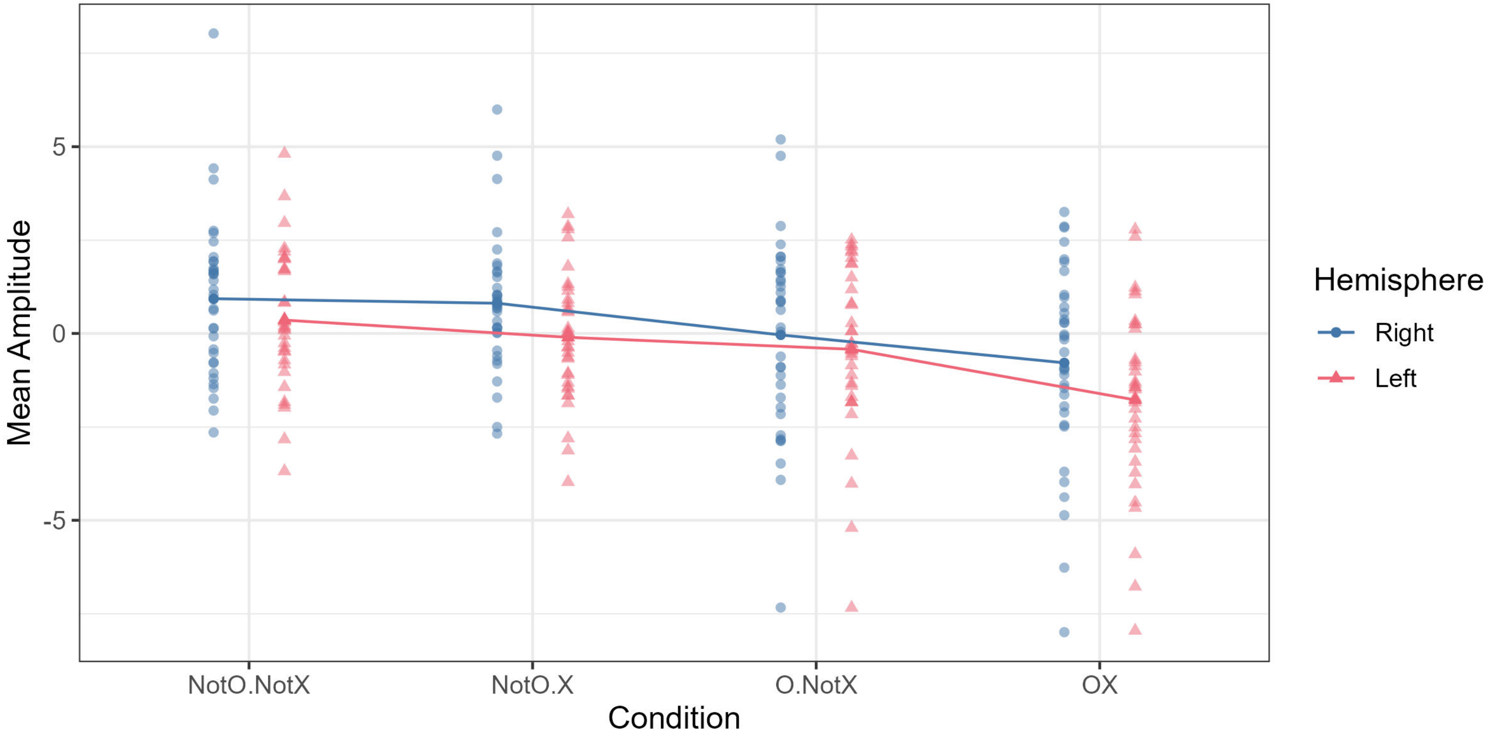

ERP Analysis (Face-CPT)

- **Continued N250:** (260-340)
- Following up on the interaction Condition: Site
  - **Condition:Site F(12, 396) = 10.380, p < .001, np2 = .239**
- If the Site factor is kept constant, then on the TP9/TP10:

Level1	Level2	Site	Difference	95% CI	SE	t(33)	p
NotO.NotX	NotO.X	TP9.TP10	0.37	[-0.25, 0.99]	0.22	1.68	0.204
NotO.NotX	O.NotX	TP9.TP10	0.65	[-0.31, 1.61]	0.34	1.91	0.196
NotO.NotX	OX	TP9.TP10	2.34	[1.11, 3.56]	0.44	5.36	< .001
NotO.X	O.NotX	TP9.TP10	0.28	[-0.55, 1.11]	0.30	0.95	0.347
NotO.X	OX	TP9.TP10	1.97	[0.89, 3.04]	0.38	5.12	< .001
O.NotX	OX	TP9.TP10	1.68	[0.90, 2.46]	0.28	6.06	< .001

- Marginal contrasts estimated at Condition
- p-value adjustment method: Holm (1979)

Condition	Site	Mean	SE	95% CI
NotO.NotX	TP9.TP10	-1.46	0.29	[-2.04, -0.87]
NotO.X	TP9.TP10	-1.83	0.22	[-2.28, -1.38]
O.NotX	TP9.TP10	-2.11	0.36	[-2.84, -1.38]
OX	TP9.TP10	-3.79	0.45	[-4.71, -2.88]

Marginal means estimated at Condition, Hemisphere

ERP Analysis (Face-CPT)

- **Continued N250:** (260-340)
- Following up on the interaction Condition: Site
  - **Condition:Site F(12, 396) = 10.380, p < .001, np2 = .239**
- If the Site factor is kept constant, then on the P7/P8:
- Level1 | Level2 | Site | Difference | 95% CI | SE | t(33) | p
- --
- NotO.NotX | NotO.X | P7.P8 | 0.19 | [-0.25, 0.63] | 0.16 | 1.22 | 0.233
- NotO.NotX | O.NotX | P7.P8 | 0.84 | [0.21, 1.47] | 0.23 | 3.72 | 0.003
- NotO.NotX | OX | P7.P8 | 1.41 | [0.72, 2.09] | 0.24 | 5.75 | < .001
- NotO.X | O.NotX | P7.P8 | 0.65 | [-0.04, 1.34] | 0.25 | 2.65 | 0.024
- NotO.X | OX | P7.P8 | 1.22 | [0.61, 1.83] | 0.22 | 5.59 | < .001
- O.NotX | OX | P7.P8 | 0.57 | [0.06, 1.07] | 0.18 | 3.14 | 0.011

Condition	Site	Mean	SE	95% CI
NotO.NotX	P7.P8	1.36	0.27	[0.82, 1.91]
NotO.X	P7.P8	1.17	0.25	[0.67, 1.67]
O.NotX	P7.P8	0.52	0.35	[-0.19, 1.24]
OX	P7.P8	-0.04	0.33	[-0.72, 0.63]

Marginal means estimated at Condition, Hemisphere

- Marginal contrasts estimated at Condition
- p-value adjustment method: Holm (1979)

ERP Analysis (Face-CPT)

- **Continued N250:** (260-340)
- Following up on the interaction Condition: Site
  - **Condition:Site $F(12, 396) = 10.380$, $p < .001$, np2 = .239**
- If the Site factor is kept constant, then on the P9/P10:

Level1	Level2	Site	Difference	95% CI	SE	t(33)	p
NotO.NotX	NotO.X	P9.P10	0.42	[-0.21, 1.05]	0.22	1.86	0.086
NotO.NotX	O.NotX	P9.P10	1.04	[0.11, 1.97]	0.33	3.12	0.011
NotO.NotX	OX	P9.P10	2.63	[1.47, 3.79]	0.41	6.36	< .001
NotO.X	O.NotX	P9.P10	0.62	[-0.21, 1.45]	0.30	2.10	0.086
NotO.X	OX	P9.P10	2.21	[1.22, 3.20]	0.35	6.25	< .001
O.NotX	OX	P9.P10	1.59	[0.83, 2.34]	0.27	5.91	< .001

- Marginal contrasts estimated at Condition
- p-value adjustment method: Holm (1979)

Condition	Site	Mean	SE	95% CI
NotO.NotX	P9.P10	-0.42	0.35	[-1.13, 0.29]
NotO.X	P9.P10	-0.84	0.27	[-1.38, -0.29]
O.NotX	P9.P10	-1.46	0.43	[-2.33, -0.60]
OX	P9.P10	-3.05	0.48	[-4.03, -2.07]

Marginal means estimated at Condition, Hemisphere

ERP Analysis (Face-CPT)

- **Continued N250:** (260-340)
- Following up on the interaction Condition: Site
  - **Condition:Site F(12, 396) = 10.380, p < .001, np2 = .239**
- If the Site factor is kept constant, then at the PO7/PO8:

Level1	Level2	Site	Difference	95% CI	SE	t(33)	p
NotO.NotX	NotO.X	PO7.PO8	0.11	[-0.37, 0.59]	0.17	0.64	> .999
NotO.NotX	O.NotX	PO7.PO8	0.89	[0.31, 1.46]	0.21	4.32	< .001
NotO.NotX	OX	PO7.PO8	0.91	[0.31, 1.52]	0.22	4.23	< .001
NotO.X	O.NotX	PO7.PO8	0.78	[0.06, 1.49]	0.25	3.06	0.013
NotO.X	OX	PO7.PO8	0.80	[0.23, 1.37]	0.20	3.96	0.002
O.NotX	OX	PO7.PO8	0.03	[-0.61, 0.66]	0.22	0.11	> .999

Condition	Site	Mean	SE	95% CI
NotO.NotX	PO7.PO8	3.19	0.34	[2.50, 3.87]
NotO.X	PO7.PO8	3.08	0.36	[2.34, 3.81]
O.NotX	PO7.PO8	2.30	0.39	[1.51, 3.09]
OX	PO7.PO8	2.27	0.37	[1.51, 3.04]

Marginal means estimated at Condition, Hemisphere

- Marginal contrasts estimated at Condition
- p-value adjustment method: Holm (1979)

ERP Analysis (Face-CPT)

- **Continued N250:** (260-340)
- Following up on the interaction Condition: Site
  - **Condition:Site $F(12, 396) = 10.380, p < .001, np2 = .239$**
- If the Site factor is kept constant, then at the PO9/PO10:

Level1	Level2	Site	Difference	95% CI	SE	t(33)	p
NotO.NotX	NotO.X	PO9.PO10	0.36	[-0.31, 1.03]	0.24	1.51	0.204
NotO.NotX	O.NotX	PO9.PO10	0.94	[-0.01, 1.90]	0.34	2.78	0.027
NotO.NotX	OX	PO9.PO10	2.32	[1.19, 3.44]	0.40	5.79	< .001
NotO.X	O.NotX	PO9.PO10	0.58	[-0.39, 1.56]	0.35	1.68	0.204
NotO.X	OX	PO9.PO10	1.95	[0.93, 2.98]	0.37	5.34	< .001
O.NotX	OX	PO9.PO10	1.37	[0.62, 2.12]	0.27	5.14	< .001

- Marginal contrasts estimated at Condition
- p-value adjustment method: Holm (1979)

Condition	Site	Mean	SE	95% CI
NotO.NotX	PO9.PO10	0.53	0.38	[-0.25, 1.31]
NotO.X	PO9.PO10	0.17	0.36	[-0.56, 0.90]
O.NotX	PO9.PO10	-0.41	0.47	[-1.36, 0.54]
OX	PO9.PO10	-1.78	0.50	[-2.80, -0.77]

Marginal means estimated at Condition, Hemisphere

ERP Analysis (Face-CPT)

- **Continued N250:** (260-340)
- Following up on the interaction Condition: Site
  - **Condition:Site F(12, 396) = 10.380, p < .001, np2 = .239**
- **Interaction plot**

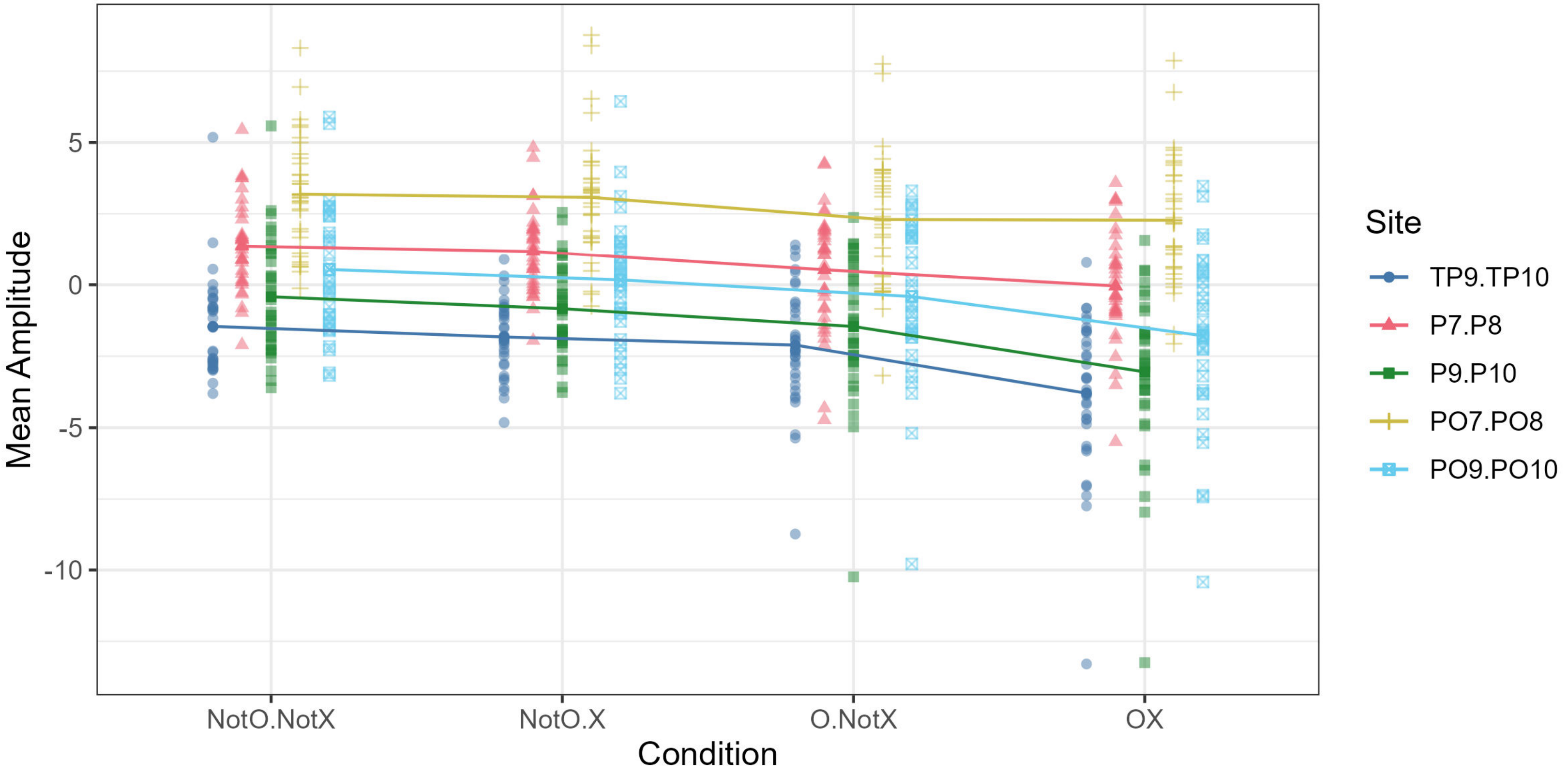

ERP Analysis (Face-CPT)

- **P300:** (300-500 ms)
- 4*2*3*2 within and between design. Factors: Condition (OX, NotOX, ONotX, NotONotX), Site or anteriority ((Central (C3,Cz,C4), Parietal (P3,Pz,P4)), Laterality (Right, Left, Central), Group (ASD, Neurotypical)
  - **Group $F(1, 33) = 6.728$, $p = .014$, np2 = .169**
  - **Condition $F(3, 99) = 46.613$, $p < .001$, np2 = .585**
  - **Laterality $F(2, 66) = 3.430$, $p = .038$, np2 = .094**
  - **Site $F(1, 33) = 67.086$, $p < .001$, np2 = .670**
  - **Group:Laterality $F(2, 66) = 4.013$, $p = .023$, np2 = .108**
  - **Condition:Laterality $F(6, 198) = 22.387$, $p < .001$, np2 = .404**
  - **Condition:Site $F(3, 99) = 31.833$, $p < .001$, np2 = .491**
  - **Group:Laterality:Site $F(2, 66) = 3.308$, $p = .043$, np2 = .091**
  - **Condition:Laterality:Site $F(6, 198) = 7.971$, $p < .001$, np2 = .195**
- Following up on the main effect of group which has only two levels and thus, we only require the means:

Group	Mean	SE	95% CI
ASD	1.15	0.26	[0.63, 1.68]
TD	2.08	0.25	[1.57, 2.59]

Marginal means estimated at Group

ERP Analysis (Face-CPT)

- **Continued P300** (300-500 ms)
- Following up on the main effect of Condition
  - **Condition F(3, 99) = 46.613, p < .001, np2 = .585**

Level1	Level2	Difference	95% CI	SE	t(33)	p	d	95% CI
NotO.NotX	NotO.X	-0.40	[-0.79, -0.01]	0.14	-2.86	0.007	0.50	[-0.86, -0.13]
NotO.NotX	O.NotX	-1.54	[-2.14, -0.94]	0.21	-7.17	< .001	-1.25	[-1.70, -0.79]
NotO.NotX	OX	-2.57	[-3.46, -1.68]	0.32	-8.08	< .001	-1.41	[-1.88, -0.92]
NotO.X	O.NotX	-1.14	[-1.77, -0.51]	0.23	-5.05	< .001	-0.88	[-1.28, -0.47]
NotO.X	OX	-2.17	[-2.94, -1.40]	0.27	-7.92	< .001	-1.38	[-1.85, -0.90]
O.NotX	OX	-1.03	[-1.69, -0.37]	0.23	-4.40	< .001	-0.77	[-1.15, -0.37]

- Marginal contrasts estimated at Condition
- p-value adjustment method: Holm (1979)
- ***Same as the Letter CPT, P300 is mostly enhanced during the target condition and then when O is the first stimulus**

Condition	Mean	SE	95% CI
NotO.NotX	0.49	0.15	[0.19, 0.80]
NotO.X	0.89	0.18	[0.52, 1.26]
O.NotX	2.03	0.23	[1.56, 2.50]
OX	3.06	0.33	[2.40, 3.72]

Marginal means estimated at Condition

ERP Analysis (Face-CPT)

- **Continued P300** (300-500 ms)
- Following up on the main effect of Laterality
  - **Laterality $F(2, 66) = 3.430$, $p = .038$, $np2 = .094$**

Level1	Level2	Difference	95% CI	SE	t(33)	p	d	95% CI
Central	Left	0.40	[-0.06, 0.85]	0.18	2.19	0.071	0.38	[0.03, 0.73]
Right	Central	0.03	[-0.47, 0.53]	0.20	0.17	0.866	0.03	[-0.31, 0.37]
Right	Left	0.43	[0.01, 0.86]	0.17	2.55	0.046	0.44	[0.08, 0.80]

- Marginal contrasts estimated at Laterality
- p-value adjustment method: Holm (1979)

Laterality	Mean	SE	95% CI
Right	1.77	0.20	[1.37, 2.17]
Central	1.74	0.26	[1.21, 2.27]
Left	1.34	0.15	[1.04, 1.65]

Marginal means estimated at Laterality

***Unlike P300 in letter CPT experiment, P300 in face CPT is mostly enhanced on the right side rather than the central side**.

ERP Analysis (Face-CPT)

- **Continued P300** (300-500 ms)
- Following up on the main effect of Site (anteriority)
  - **Site $F(1, 33) = 67.086$, $p < .001$, np2 = .670**

Site	Mean	SE	95% CI
C3.Cz.C4	0.54	0.23	[0.07, 1.01]
P3.Pz.P4	2.70	0.21	[2.26, 3.13]

Marginal means estimated at Site

*** Similar to P300 in letter CPT experiment, and as expected P300 in face CPT is mostly enhanced at parietal areas.**

ERP Analysis (Face-CPT)

- **Continued P300** (300-500 ms)
- Following up on the interaction of Group: Laterality
  - **Group:Laterality F(2, 66) = 4.013, p = .023, np2 = .108**
- If laterality is kept constant, then on the right, central, and left side, the difference between ASD and typically developed individuals are:

Level1	Level2	Laterality	Difference	95% CI	SE	t(33)	p	d	95% CI
ASD	TD	Central	-1.47	[-2.53, -0.40]	0.52	-2.81	0.008	0.98	[-1.69, -0.25]
ASD	TD	Left	-0.88	[-1.49, -0.27]	0.30	-2.93	0.006	-1.02	[-1.74, -0.29]
ASD	TD	Right	-0.43	[-1.23, 0.36]	0.39	-1.11	0.276	-0.39	[-1.07, 0.31]

- Marginal contrasts estimated at Group
- p-value adjustment method: Holm (1979)

Group	Laterality	Mean	SE	95% CI
ASD	Right	1.56	0.28	[0.98, 2.13]
TD	Right	1.99	0.27	[1.43, 2.55]
ASD	Central	1.00	0.38	[0.24, 1.77]
TD	Central	2.47	0.36	[1.73, 3.22]
ASD	Left	0.90	0.22	[0.46, 1.34]
TD	Left	1.78	0.21	[1.36, 2.21]

Marginal means estimated at Group, Laterality

ERP Analysis (Face-CPT)

- **Continued P300** (300-500 ms)
- Following up on the interaction of Group: Laterality
    - **Group:Laterality $F(2, 66) = 4.013$, $p = .023$, np2 = .108**
- **Interaction plot**

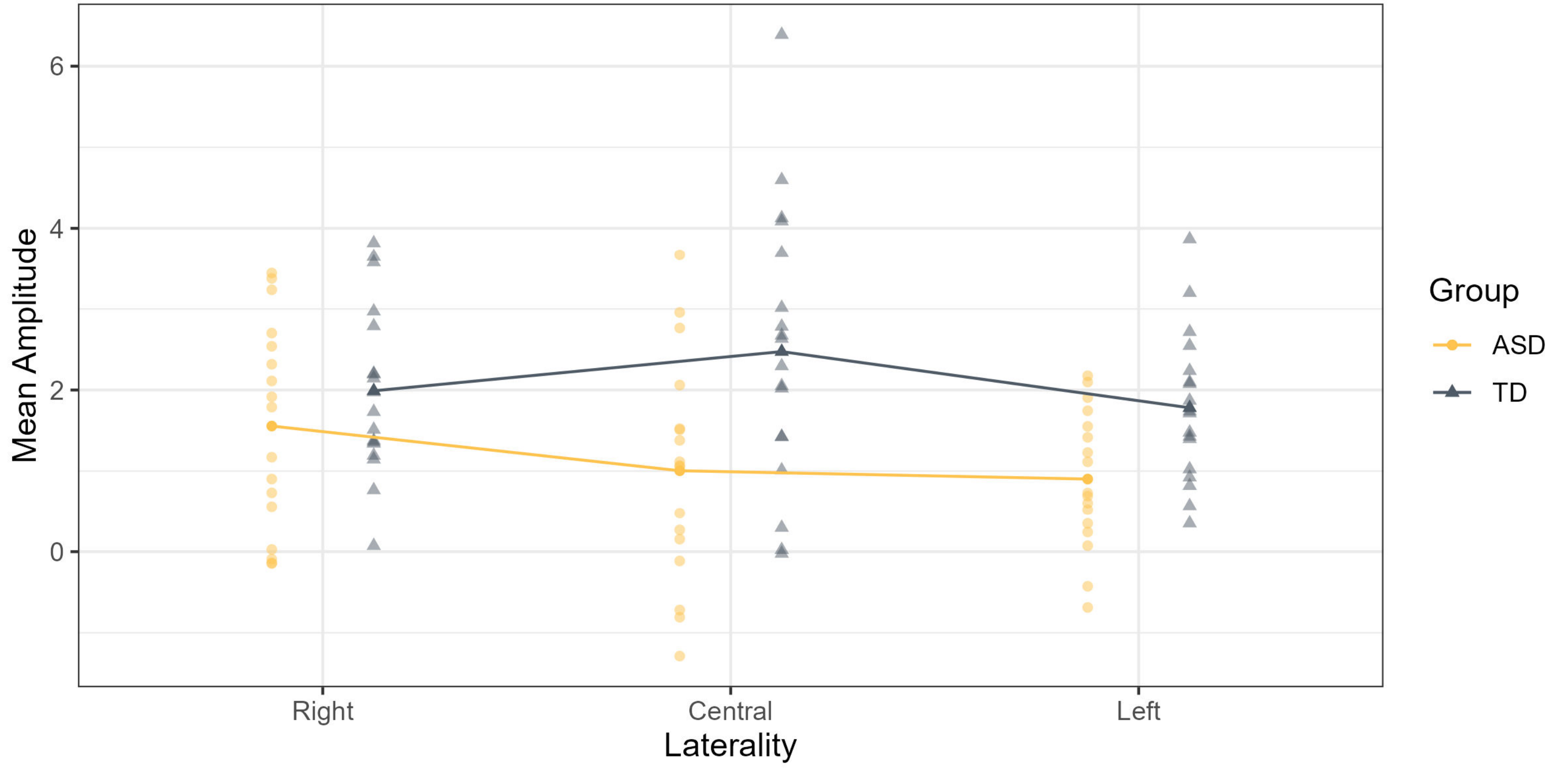

ERP Analysis (Face-CPT)

- **Continued P300** (300-500 ms)
- Following up on the interaction of Condition: Laterality
  - **Condition:Laterality $F(6, 198) = 22.387$, $p < .001$, np2 = .404**
- If laterality is kept constant, then on the right side:

Level1	Level2	Laterality	Difference	95% CI	SE	t(33)	p
NotO.NotX	NotO.X	Right	-0.22	[-0.59, 0.15]	0.13	-1.64	0.111
NotO.NotX	O.NotX	Right	-1.09	[-1.83, -0.35]	0.26	-4.14	< .001
NotO.NotX	OX	Right	-2.11	[-3.07, -1.16]	0.34	-6.20	< .001
NotO.X	O.NotX	Right	-0.87	[-1.59, -0.15]	0.26	-3.41	0.003
NotO.X	OX	Right	-1.90	[-2.81, -0.98]	0.32	-5.84	< .001
O.NotX	OX	Right	-1.02	[-1.62, -0.43]	0.21	-4.80	< .001

- Marginal contrasts estimated at Condition
- p-value adjustment method: Holm (1979)

Condition	Laterality	Mean	SE	95% CI
NotO.NotX	Right	0.92	0.20	[0.51, 1.32]
NotO.X	Right	1.13	0.20	[0.72, 1.55]
O.NotX	Right	2.01	0.26	[1.47, 2.54]
OX	Right	3.03	0.33	[2.36, 3.70]

Marginal means estimated at Condition, Laterality

ERP Analysis (Face-CPT)

- **Continued P300** (300-500 ms)
- Following up on the interaction of Condition: Laterality
  - **Condition:Laterality F(6, 198) = 22.387, p < .001, np2 = .404**
- If laterality is kept constant, then on the central side:

Level1	Level2	Laterality	Difference	95% CI	SE	t(33)	p
NotO.NotX	NotO.X	Central	-0.74	[-1.27, -0.20]	0.19	-3.83	0.001
NotO.NotX	O.NotX	Central	-2.67	[-3.45, -1.89]	0.28	-9.61	< .001
NotO.NotX	OX	Central	-3.68	[-4.85, -2.52]	0.41	-8.88	< .001
NotO.X	O.NotX	Central	-1.93	[-2.80, -1.07]	0.31	-6.30	< .001
NotO.X	OX	Central	-2.95	[-3.91, -1.98]	0.35	-8.53	< .001
O.NotX	OX	Central	-1.01	[-1.88, -0.15]	0.31	-3.28	0.002

- Marginal contrasts estimated at Condition
- p-value adjustment method: Holm (1979)

Condition	Laterality	Mean	SE	95% CI
NotO.NotX	Central	-0.03	0.20	[-0.45, 0.38]
NotO.X	Central	0.70	0.26	[0.17, 1.23]
O.NotX	Central	2.64	0.34	[1.95, 3.32]
OX	Central	3.65	0.45	[2.74, 4.56]

Marginal means estimated at Condition, Laterality

ERP Analysis (Face-CPT)

- **Continued P300** (300-500 ms)
- Following up on the interaction of Condition: Laterality
  - **Condition:Laterality $F(6, 198) = 22.387$, $p < .001$, np2 = .404**
- If laterality is kept constant, then on the left side:

Level1	Level2	Laterality	Difference	95% CI	SE	t(33)	p
NotO.NotX	NotO.X	Left	-0.25	[-0.67, 0.18]	0.15	-1.63	0.113
NotO.NotX	O.NotX	Left	-0.85	[-1.40, -0.30]	0.20	-4.35	< .001
NotO.NotX	OX	Left	-1.91	[-2.65, -1.17]	0.26	-7.23	< .001
NotO.X	O.NotX	Left	-0.61	[-1.21, -0.01]	0.21	-2.84	0.015
NotO.X	OX	Left	-1.66	[-2.27, -1.05]	0.22	-7.61	< .001
O.NotX	OX	Left	-1.05	[-1.79, -0.32]	0.26	-4.05	< .001

- Marginal contrasts estimated at Condition
- p-value adjustment method: Holm (1979)

Condition	Laterality	Mean	SE	95% CI
NotO.NotX	Left	0.59	0.14	[0.30, 0.88]
NotO.X	Left	0.84	0.19	[0.46, 1.21]
O.NotX	Left	1.44	0.18	[1.08, 1.80]
OX	Left	2.50	0.28	[1.94, 3.06]

Marginal means estimated at Condition, Laterality

ERP Analysis (Face-CPT)

- **Continued P300** (300-500 ms)
- Following up on the interaction of Condition: Laterality
  - **Condition:Laterality F(6, 198) = 22.387, p < .001, np2 = .404**
- Interaction plot

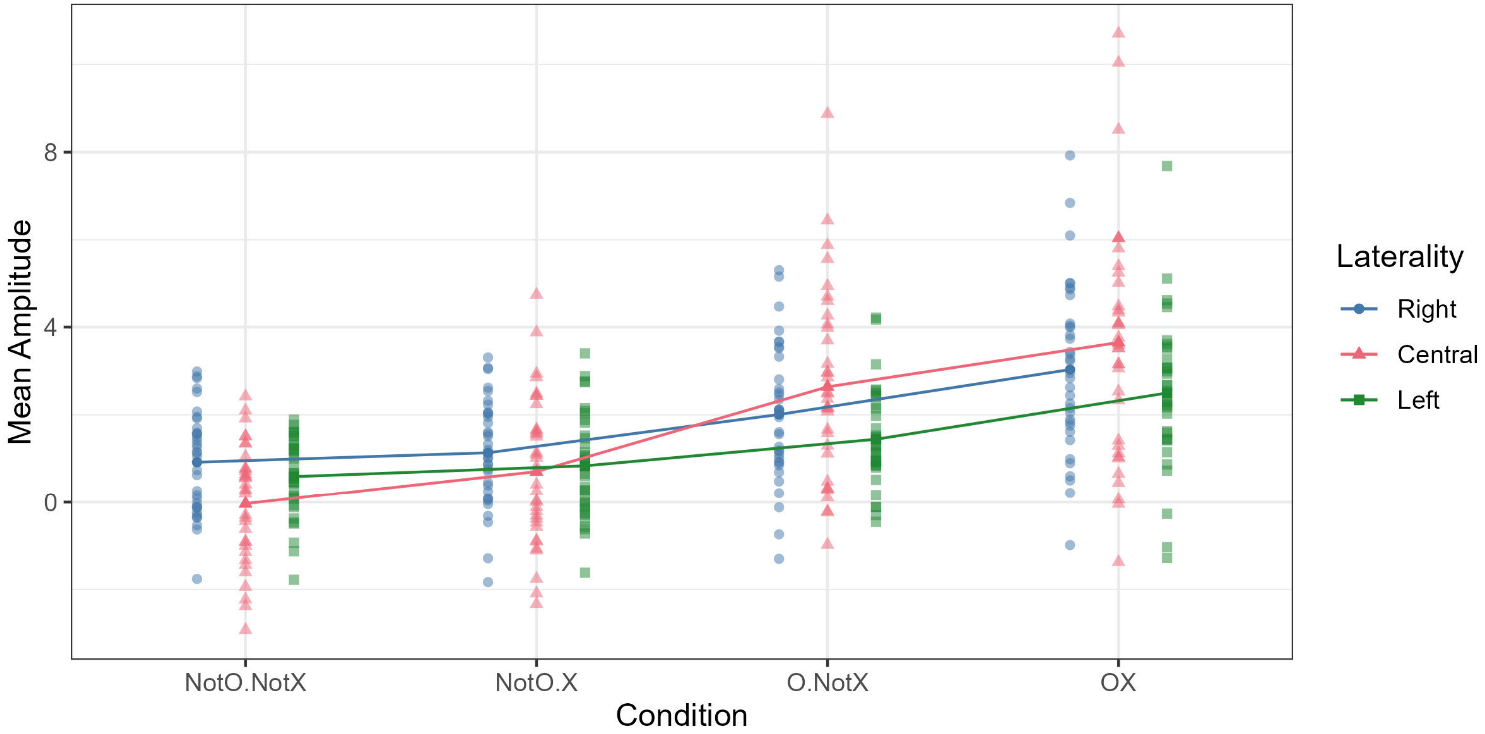

ERP Analysis (Face-CPT)

- **Continued P300** (300-500 ms)
- Following up on the interaction of Condition: Site (anteriority)
  - **Condition:Site $F(3, 99) = 31.833$, $p < .001$, np2 = .491**
- If Site is kept constant, then at the central areas:

Level1	Level2	Site	Difference	95% CI	SE	t(33)	p
NotO.NotX	NotO.X	C3.Cz.C4	-0.12	[-0.51, 0.26]	0.14	-0.90	0.750
NotO.NotX	O.NotX	C3.Cz.C4	-2.13	[-2.89, -1.37]	0.27	-7.86	< .001
NotO.NotX	OX	C3.Cz.C4	-1.96	[-3.02, -0.90]	0.38	-5.20	< .001
NotO.X	O.NotX	C3.Cz.C4	-2.01	[-2.76, -1.25]	0.27	-7.46	< .001
NotO.X	OX	C3.Cz.C4	-1.84	[-2.76, -0.92]	0.33	-5.64	< .001
O.NotX	OX	C3.Cz.C4	0.17	[-0.62, 0.95]	0.28	0.59	0.750

- Marginal contrasts estimated at Condition
- p-value adjustment method: Holm (1979)

Condition	Site	Mean	SE	95% CI
NotO.NotX	C3.Cz.C4	-0.51	0.22	[-0.97, -0.06]
NotO.X	C3.Cz.C4	-0.39	0.21	[-0.82, 0.04]
O.NotX	C3.Cz.C4	1.61	0.31	[0.98, 2.25]
OX	C3.Cz.C4	1.45	0.38	[0.68, 2.22]

Marginal means estimated at Condition, Site

ERP Analysis (Face-CPT)

- **Continued P300** (300-500 ms)
- Following up on the interaction of Condition:Site
  - **Condition:Site $F(3, 99) = 31.833$, $p < .001$, $np2 = .491$**
- If Site is kept constant, then at the parietal areas:

Level1	Level2	Site	Difference	95% CI	SE	t(33)	p
NotO.NotX	NotO.X	P3.Pz.P4	-0.67	[-1.18, -0.17]	0.18	-3.75	0.001
NotO.NotX	O.NotX	P3.Pz.P4	-0.94	[-1.54, -0.35]	0.21	-4.44	< .001
NotO.NotX	OX	P3.Pz.P4	-3.17	[-4.07, -2.28]	0.32	-9.96	< .001
NotO.X	O.NotX	P3.Pz.P4	-0.27	[-0.95, 0.41]	0.24	-1.12	0.271
NotO.X	OX	P3.Pz.P4	-2.50	[-3.29, -1.70]	0.28	-8.79	< .001
O.NotX	OX	P3.Pz.P4	-2.23	[-3.00, -1.45]	0.28	-8.05	< .001

- Marginal contrasts estimated at Condition
- p-value adjustment method: Holm (1979)

Condition	Site	Mean	SE	95% CI
NotO.NotX	P3.Pz.P4	1.50	0.16	[1.17, 1.83]
NotO.X	P3.Pz.P4	2.17	0.24	[1.68, 2.66]
O.NotX	P3.Pz.P4	2.44	0.27	[1.90, 2.98]
OX	P3.Pz.P4	4.67	0.36	[3.94, 5.39]

Marginal means estimated at Condition, Site

ERP Analysis (Face-CPT)

- **Continued P300** (300-500 ms)
- Following up on the interaction of Condition: Site
    - **Condition:Site $F(3, 99) = 31.833$, $p < .001$, np2 = .491**
- **Interaction plot**

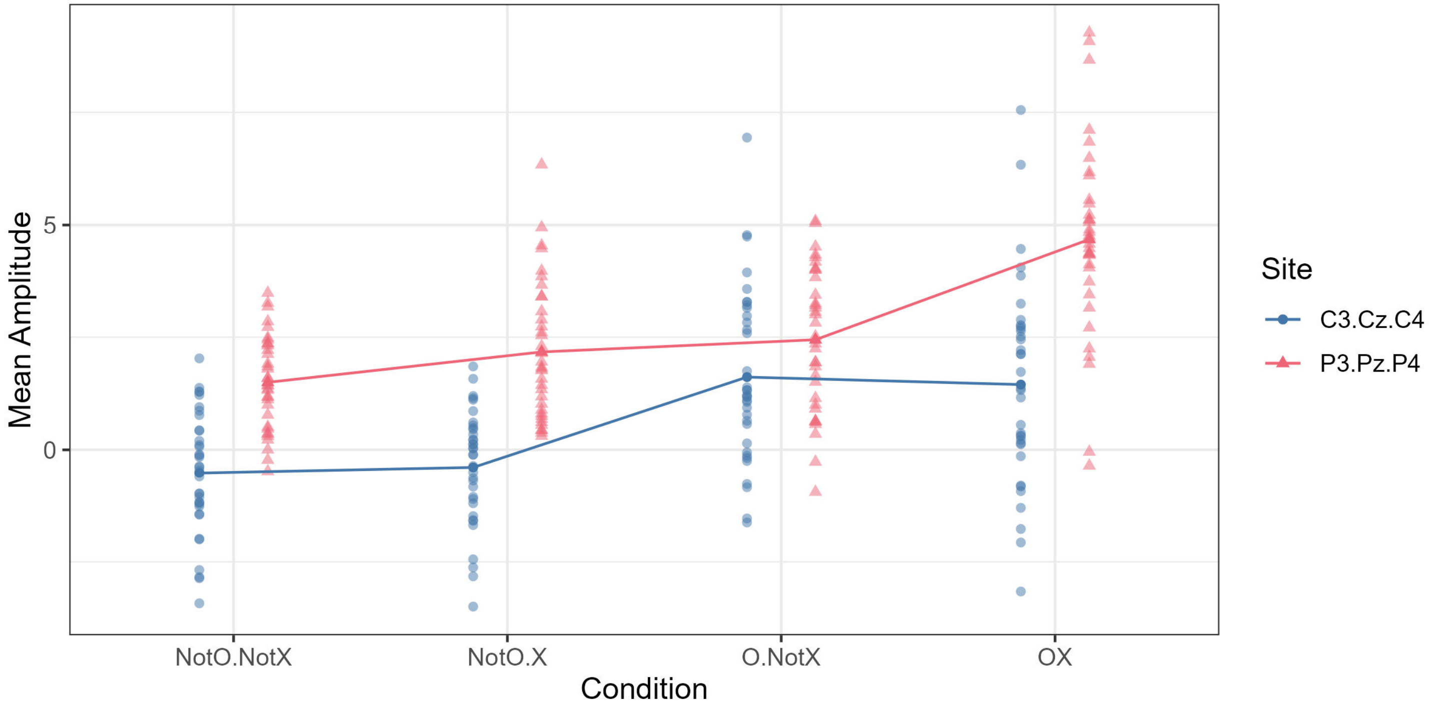

ERP Analysis (Face-CPT)

- **Continued P300** (300-500 ms)
- Following up on the 3-way interaction of Group: Laterality: Site (anteriority)
  - **Group:Laterality:Site F(2, 66) = 3.308, p = .043, np2 = .091**
- If the factor Site is kept constant, the follow-up analysis for the central site shows:
- Group F(1, 33) = 4.022, p = .053, np2 = .109
- Laterality F(2, 66) = 3.378, p = .040, np2 = .093
- Group:Laterality F(2, 66) = 3.733, p = .029, np2 = .102
- For the effect of laterality:
- Level1 | Level2 | Difference | 95% CI | SE | t(33) | p
- ---
- Central | Left | 0.46 | [-0.04, 0.96] | 0.20 | 2.34 | 0.066
- Right | Central | -0.10 | [-0.62, 0.42] | 0.21 | -0.48 | 0.631
- Right | Left | 0.36 | [-0.02, 0.74] | 0.15 | 2.41 | 0.066
- Marginal contrasts estimated at Laterality
- p-value adjustment method: Holm (1979)

Laterality	Mean	SE	95% CI
Right	0.63	0.22	[0.18, 1.07]
Central	0.73	0.33	[0.06, 1.39]
Left	0.27	0.20	[-0.14, 0.67]

Marginal means estimated at Laterality

ERP Analysis (Face-CPT)

- **Continued P300** (300-500 ms)
- Following up on the 3-way interaction of Group: Laterality: Site
  - **Group:Laterality:Site F(2, 66) = 3.308, p = .043, np2 = .091**
- If the factor Site is kept constant, the follow-up analysis for the central site shows:
- Group F(1, 33) = 4.022, p = .053, np2 = .109
- Laterality F(2, 66) = 3.378, p = .040, np2 = .093
- Group:Laterality F(2, 66) = 3.733, p = .029, np2 = .102
- For the interaction of Group:laterality:

Level1	Level2	Laterality	Difference	95% CI	SE	t(33)	p
ASD	TD	Central	-1.51	[-2.83, -0.18]	0.65	-2.32	0.027
ASD	TD	Left	-0.67	[-1.48, 0.14]	0.40	-1.69	0.100
ASD	TD	Right	-0.59	[-1.48, 0.30]	0.44	-1.34	0.189

Group	Laterality	Mean	SE	95% CI
ASD	Right	0.33	0.32	[-0.31, 0.97]
TD	Right	0.92	0.31	[0.30, 1.54]
ASD	Central	-0.03	0.47	[-0.98, 0.92]
TD	Central	1.48	0.45	[0.56, 2.40]
ASD	Left	-0.07	0.28	[-0.65, 0.51]
TD	Left	0.60	0.28	[0.04, 1.17]

Marginal means estimated at Group, Laterality

ERP Analysis (Face-CPT)

- **Continued P300** (300-500 ms)
- Following up on the 3-way interaction of Group: Laterality: Site
    - **Group:Laterality:Site F(2, 66) = 3.308, p = .043, np2 = .091**
- If the factor Site is kept constant, the follow-up analysis for the parietal site shows:
- Group F(1, 33) = 4.759, p = .036, np2 = .126
- Laterality F(2, 66) = 3.064, p = .053, np2 = .085
- Group:Laterality F(2, 66) = 4.069, p = .022, np2 = .110
- For the effect of the Group:

Group	Mean	SE	95% CI
ASD	2.23	0.31	[1.61, 2.85]
TD	3.16	0.30	[2.56, 3.77]

Marginal means estimated at Group

ERP Analysis (Face-CPT)

- **Continued P300** (300-500 ms)
- Following up on the 3-way interaction of Group: Laterality: Site
  - **Group:Laterality:Site F(2, 66) = 3.308, p = .043, np2 = .091**
- If the factor Site is kept constant, the follow-up analysis for the Parietal site shows:
- Group F(1, 33) = 4.022, p = .053, np2 = .109
- Laterality F(2, 66) = 3.378, p = .040, np2 = .093
- Group:Laterality F(2, 66) = 3.733, p = .029, np2 = .102
- For the interaction of Group:laterality:

Level1	Level2	Laterality	Difference	95% CI	SE	t(33)	p
ASD	TD	Central	-1.43	[-2.57, -0.29]	0.56	-2.55	0.015
ASD	TD	Left	-1.09	[-1.89, -0.28]	0.40	-2.74	0.010
ASD	TD	Right	-0.28	[-1.29, 0.73]	0.50	-0.56	0.578

Group	Laterality	Mean	SE	95% CI
ASD	Right	2.78	0.36	[2.05, 3.51]
TD	Right	3.06	0.35	[2.35, 3.77]
ASD	Central	2.04	0.40	[1.22, 2.85]
TD	Central	3.47	0.39	[2.67, 4.26]
ASD	Left	1.87	0.28	[1.29, 2.45]
TD	Left	2.96	0.28	[2.40, 3.52]

Marginal means estimated at Group, Laterality

ERP Analysis (Face-CPT)

- **Continued P300** (300-500 ms)
- Following up on the 3-way interaction of Group: Laterality: Site
    - **Group:Laterality:Site $F(2, 66) = 3.308$, $p = .043$, np2 = .091**
- **Interaction plot**

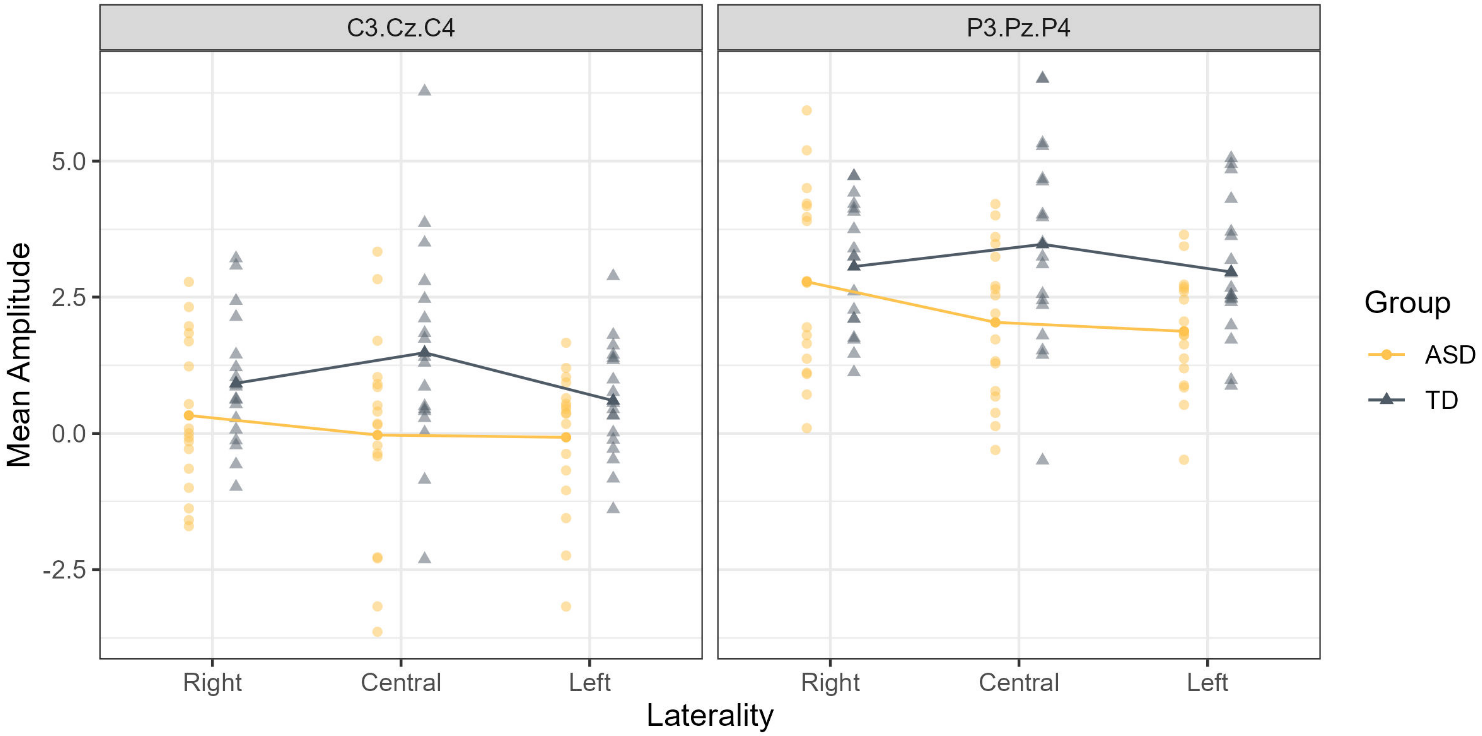

ERP Analysis (Face-CPT)

- **Continued P300** (300-500 ms)
- Following up on the 3-way interaction of Condition: Laterality: Site (anteriority)
  - **Condition:Laterality:Site F(6, 198) = 7.971, p < .001, np2 = .195**
- If the factor Site is kept constant, the follow-up analysis for the central site shows:
- Laterality F(2, 68) = 3.246, p = .052, np2 = .087
- Condition F(3, 102) = 32.731, p < .001, np2 = .490
- Laterality:Condition F(6, 204) = 22.129, p < .001, np2 = .394
- For the effect of Condition:
- Level1 | Level2 | Difference | 95% CI | SE | t(34) | p
- --
- NotO.NotX | NotO.X | -0.12 | [-0.51, 0.26] | 0.14 | -0.91 | 0.735
- NotO.NotX | O.NotX | -2.14 | [-2.90, -1.38] | 0.27 | -7.86 | < .001
- NotO.NotX | OX | -1.97 | [-3.02, -0.92] | 0.38 | -5.26 | < .001
- NotO.X | O.NotX | -2.01 | [-2.77, -1.26] | 0.27 | -7.46 | < .001
- NotO.X | OX | -1.85 | [-2.76, -0.94] | 0.32 | -5.69 | < .001
- O.NotX | OX | 0.17 | [-0.61, 0.94] | 0.28 | 0.60 | 0.735
- Marginal contrasts estimated at Condition/p-value adjustment method: Holm (1979)

Condition	Mean	SE	95% CI
NotO.NotX	-0.51	0.23	[-0.97, -0.04]
NotO.X	-0.38	0.22	[-0.82, 0.06]
O.NotX	1.63	0.32	[0.98, 2.29]
OX	1.47	0.39	[0.68, 2.25]

Marginal means estimated at Condition

ERP Analysis (Face-CPT)

- **Continued P300** (300-500 ms)
- Following up on the 3-way interaction of Condition: Laterality: Site
  - **Condition:Laterality:Site F(6, 198) = 7.971, p < .001, np2 = .195**
- If the factor Site (C3.Cz.C4, P3.Pz.P4)is kept constant, the follow-up analysis for the central site shows:
- Laterality F(2, 68) = 3.246, p = .052, np2 = .087
- Condition F(3, 102) = 32.731, p < .001, np2 = .490
- Laterality:Condition F(6, 204) = 22.129, p < .001, np2 = .394
- For the interaction effect of Condition: Laterality (Right, Central, Left), if laterality is constant, then on the right side:

Level1	Level2	Laterality	Difference	95% CI	SE	t(34)	p
NotO.NotX	NotO.X	Right	0.11	[-0.42, 0.63]	0.19	0.57	> .999
NotO.NotX	O.NotX	Right	-1.48	[-2.41, -0.56]	0.33	-4.50	< .001
NotO.NotX	OX	Right	-1.56	[-2.61, -0.51]	0.37	-4.16	< .001
NotO.X	O.NotX	Right	-1.59	[-2.41, -0.77]	0.29	-5.43	< .001
NotO.X	OX	Right	-1.67	[-2.62, -0.71]	0.34	-4.86	< .001
O.NotX	OX	Right	-0.08	[-0.78, 0.63]	0.25	-0.30	> .999

- Marginal contrasts estimated at Condition/ p-value adjustment method: Holm (1979)

Condition	Laterality	Mean	SE	95% CI
NotO.NotX	Right	-0.10	0.25	[-0.61, 0.41]
NotO.X	Right	-0.21	0.22	[-0.66, 0.25]
O.NotX	Right	1.38	0.32	[0.74, 2.02]
OX	Right	1.46	0.35	[0.75, 2.17]

Marginal means estimated at Condition, Laterality

ERP Analysis (Face-CPT)

- **Continued P300** (300-500 ms)
- Following up on the 3-way interaction of Condition: Laterality: Site
  - **Condition:Laterality:Site F(6, 198) = 7.971, p < .001, np2 = .195**
- If the factor Site (C3.Cz.C4, P3.Pz.P4) is kept constant, the follow-up analysis for the central site shows:
- Laterality F(2, 68) = 3.246, p = .052, np2 = .087
- Condition F(3, 102) = 32.731, p < .001, np2 = .490
- Laterality:Condition F(6, 204) = 22.129, p < .001, np2 = .394
- For the interaction effect of Condition: Laterality, if laterality (Right, Central, Left)is constant, then on the central side:

Level1	Level2	Laterality	Difference	95% CI	SE	t(34)	p
NotO.NotX	NotO.X	Central	-0.39	[-0.90, 0.13]	0.18	-2.11	0.084
NotO.NotX	O.NotX	Central	-3.58	[-4.59, -2.57]	0.36	-9.95	< .001
NotO.NotX	OX	Central	-2.99	[-4.41, -1.56]	0.51	-5.88	< .001
NotO.X	O.NotX	Central	-3.19	[-4.22, -2.16]	0.37	-8.70	< .001
NotO.X	OX	Central	-2.60	[-3.79, -1.41]	0.42	-6.12	< .001
O.NotX	OX	Central	0.59	[-0.47, 1.66]	0.38	1.56	0.128

- Marginal contrasts estimated at Condition/ p-value adjustment method: Holm (1979)

Condition	Laterality	Mean	SE	95% CI
NotO.NotX	Central	-0.99	0.30	[-1.59, -0.39]
NotO.X	Central	-0.60	0.30	[-1.21, 0.00]
O.NotX	Central	2.59	0.47	[1.63, 3.55]
OX	Central	2.00	0.55	[0.88, 3.11]

Marginal means estimated at Condition, Laterality

ERP Analysis (Face-CPT)

- **Continued P300** (300-500 ms)
- Following up on the 3-way interaction of Condition: Laterality: Site
  - **Condition:Laterality:Site F(6, 198) = 7.971, p < .001, np2 = .195**
- If the factor Site (C3.Cz.C4, P3.Pz.P4) is kept constant, the follow-up analysis for the central site shows:
- Laterality F(2, 68) = 3.246, p = .052, np2 = .087
- Condition F(3, 102) = 32.731, p < .001, np2 = .490
- Laterality:Condition F(6, 204) = 22.129, p < .001, np2 = .394
- For the interaction effect of Condition: Laterality, if laterality (Right, Central, Left)is constant, then on the left side:

Level1	Level2	Laterality	Difference	95% CI	SE	t(34)	p
NotO.NotX	NotO.X	Left	-0.09	[-0.52, 0.34]	0.15	-0.59	> .999
NotO.NotX	O.NotX	Left	-1.35	[-2.03, -0.68]	0.24	-5.60	< .001
NotO.NotX	OX	Left	-1.37	[-2.23, -0.51]	0.31	-4.45	< .001
NotO.X	O.NotX	Left	-1.26	[-1.95, -0.57]	0.25	-5.12	< .001
NotO.X	OX	Left	-1.28	[-2.06, -0.49]	0.28	-4.56	< .001
O.NotX	OX	Left	-0.02	[-0.84, 0.81]	0.29	-0.05	> .999

- Marginal contrasts estimated at Condition/ p-value adjustment method: Holm (1979)

Condition	Laterality	Mean	SE	95% CI
NotO.NotX	Left	-0.43	0.21	[-0.86, 0.00]
NotO.X	Left	-0.34	0.21	[-0.76, 0.08]
O.NotX	Left	0.93	0.27	[0.38, 1.47]
OX	Left	0.94	0.33	[0.27, 1.61]

Marginal means estimated at Condition, Laterality

ERP Analysis (Face-CPT)

- **Continued P300** (300-500 ms)
- Following up on the 3-way interaction of Condition: Laterality: Site
  - **Condition:Laterality:Site F(6, 198) = 7.971, p < .001, np2 = .195**
- If the factor Site (C3.Cz.C4, P3.Pz.P4) is kept constant, the follow-up analysis for the parietal site shows:
- Laterality F(2, 68) = 2.717, p = .073, np2 = .074
- Condition F(3, 102) = 58.693, p < .001, np2 = .633
- Laterality:Condition F(6, 204) = 15.781, p < .001, np2 = .317
- For the effect of Condition:

Level1	Level2	Difference	95% CI	SE	t(34)	p
NotO.NotX	NotO.X	-0.68	[-1.18, -0.18]	0.18	-3.79	0.001
NotO.NotX	O.NotX	-0.94	[-1.53, -0.35]	0.21	-4.49	< .001
NotO.NotX	OX	-3.17	[-4.05, -2.29]	0.31	-10.11	< .001
NotO.X	O.NotX	-0.26	[-0.94, 0.41]	0.24	-1.09	0.283
NotO.X	OX	-2.49	[-3.28, -1.71]	0.28	-8.89	< .001
O.NotX	OX	-2.23	[-3.00, -1.46]	0.27	-8.16	< .001

- Marginal contrasts estimated at Condition/p-value adjustment method: Holm (1979)

Condition	Mean	SE	95% CI
NotO.NotX	1.51	0.18	[1.15, 1.87]
NotO.X	2.19	0.26	[1.67, 2.71]
O.NotX	2.45	0.27	[1.91, 3.00]
OX	4.68	0.36	[3.95, 5.42]

Marginal means estimated at Condition

ERP Analysis (Face-CPT)

- **Continued P300** (300-500 ms)
- Following up on the 3-way interaction of Condition: Laterality: Site
  - **Condition:Laterality:Site F(6, 198) = 7.971, p < .001, np2 = .195**
- If the factor Site (C3.Cz.C4, P3.Pz.P4) is kept constant, the follow-up analysis for the parietal site shows:
- Laterality F(2, 68) = 2.717, p = .073, np2 = .074
- Condition F(3, 102) = 58.693, p < .001, np2 = .633
- Laterality:Condition F(6, 204) = 15.781, p < .001, np2 = .317
- For the interaction effect of Condition: Laterality, if the Laterality (Right, Central, Left) factor is constant, then on the right side:
- Level1 | Level2 | Laterality | Difference | 95% CI | SE | t(34) | p
- ---
- NotO.NotX | NotO.X | Right | -0.54 | [-1.05, -0.03] | 0.18 | -2.97 | 0.016
- NotO.NotX | O.NotX | Right | -0.69 | [-1.40, 0.02] | 0.25 | -2.74 | 0.020
- NotO.NotX | OX | Right | -2.67 | [-3.71, -1.63] | 0.37 | -7.22 | < .001
- NotO.X | O.NotX | Right | -0.15 | [-0.90, 0.59] | 0.27 | -0.58 | 0.567
- NotO.X | OX | Right | -2.13 | [-3.16, -1.10] | 0.37 | -5.80 | < .001
- O.NotX | OX | Right | -1.98 | [-2.73, -1.23] | 0.27 | -7.39 | < .001
- Marginal contrasts estimated at Condition/p-value adjustment method: Holm (1979)

Condition	Laterality	Mean	SE	95% CI
NotO.NotX	Right	1.95	0.23	[1.47, 2.42]
NotO.X	Right	2.49	0.27	[1.94, 3.03]
O.NotX	Right	2.64	0.32	[2.00, 3.29]
OX	Right	4.62	0.38	[3.85, 5.39]

Marginal means estimated at Condition, Laterality

ERP Analysis (Face-CPT)

- **Continued P300** (300-500 ms)
- Following up on the 3-way interaction of Condition: Laterality: Site
  - **Condition:Laterality:Site F(6, 198) = 7.971, p < .001, np2 = .195**
- If the factor Site (C3.Cz.C4, P3.Pz.P4) is kept constant, the follow-up analysis for the parietal site shows:
- Laterality F(2, 68) = 2.717, p = .073, np2 = .074
- Condition F(3, 102) = 58.693, p < .001, np2 = .633
- Laterality:Condition F(6, 204) = 15.781, p < .001, np2 = .317
- For the interaction effect of Condition: Laterality, if the Laterality factor is constant, then on the central side:

Level1	Level2	Laterality	Difference	95% CI	SE	t(34)	p
NotO.NotX	NotO.X	Central	-1.09	[-1.74, -0.44]	0.23	-4.70	< .001
NotO.NotX	O.NotX	Central	-1.78	[-2.48, -1.07]	0.25	-7.04	< .001
NotO.NotX	OX	Central	-4.38	[-5.45, -3.32]	0.38	-11.52	< .001
NotO.X	O.NotX	Central	-0.69	[-1.58, 0.20]	0.32	-2.17	0.037
NotO.X	OX	Central	-3.30	[-4.25, -2.34]	0.34	-9.71	< .001
O.NotX	OX	Central	-2.61	[-3.54, -1.67]	0.33	-7.83	< .001

- Marginal contrasts estimated at Condition/p-value adjustment method: Holm (1979)

Condition	Laterality	Mean	SE	95% CI
NotO.NotX	Central	0.96	0.24	[0.47, 1.45]
NotO.X	Central	2.05	0.34	[1.35, 2.74]
O.NotX	Central	2.74	0.35	[2.02, 3.45]
OX	Central	5.34	0.46	[4.41, 6.28]

Marginal means estimated at Condition, Laterality

ERP Analysis (Face-CPT)

- **Continued P300** (300-500 ms)
- Following up on the 3-way interaction of Condition: Laterality: Site
  - **Condition:Laterality:Site F(6, 198) = 7.971, p < .001, np2 = .195**
- If the factor Site (C3.Cz.C4, P3.Pz.P4) is kept constant, the follow-up analysis for the parietal site shows:
- Laterality F(2, 68) = 2.717, p = .073, np2 = .074
- Condition F(3, 102) = 58.693, p < .001, np2 = .633
- Laterality:Condition F(6, 204) = 15.781, p < .001, np2 = .317
- For the interaction effect of Condition: Laterality, if the Laterality factor is constant, then on the left side:

Level1	Level2	Laterality	Difference	95% CI	SE	t(34)	p
NotO.NotX	NotO.X	Left	-0.41	[-0.96, 0.14]	0.20	-2.07	0.138
NotO.NotX	O.NotX	Left	-0.36	[-0.97, 0.26]	0.22	-1.63	0.223
NotO.NotX	OX	Left	-2.46	[-3.26, -1.67]	0.28	-8.66	< .001
NotO.X	O.NotX	Left	0.05	[-0.68, 0.78]	0.26	0.20	0.842
NotO.X	OX	Left	-2.05	[-2.65, -1.46]	0.21	-9.62	< .001
O.NotX	OX	Left	-2.11	[-2.99, -1.22]	0.31	-6.69	< .001

- Marginal contrasts estimated at Condition/p-value adjustment method: Holm (1979)

Condition	Laterality	Mean	SE	95% CI
NotO.NotX	Left	1.62	0.17	[1.27, 1.98]
NotO.X	Left	2.03	0.28	[1.47, 2.60]
O.NotX	Left	1.98	0.23	[1.51, 2.46]
OX	Left	4.09	0.35	[3.38, 4.79]

Marginal means estimated at Condition, Laterality

ERP Analysis (Face-CPT)

- **Continued P300** (300-500 ms)
- Following up on the 3-way interaction of Condition: Laterality: Site
  - **Condition:Laterality:Site F(6, 198) = 7.971, p < .001, np2 = .195**
- **3-way Interaction plot (**I created two types of interaction plot, each representing the same data, with different visualization**)**
- **Plot 1:**

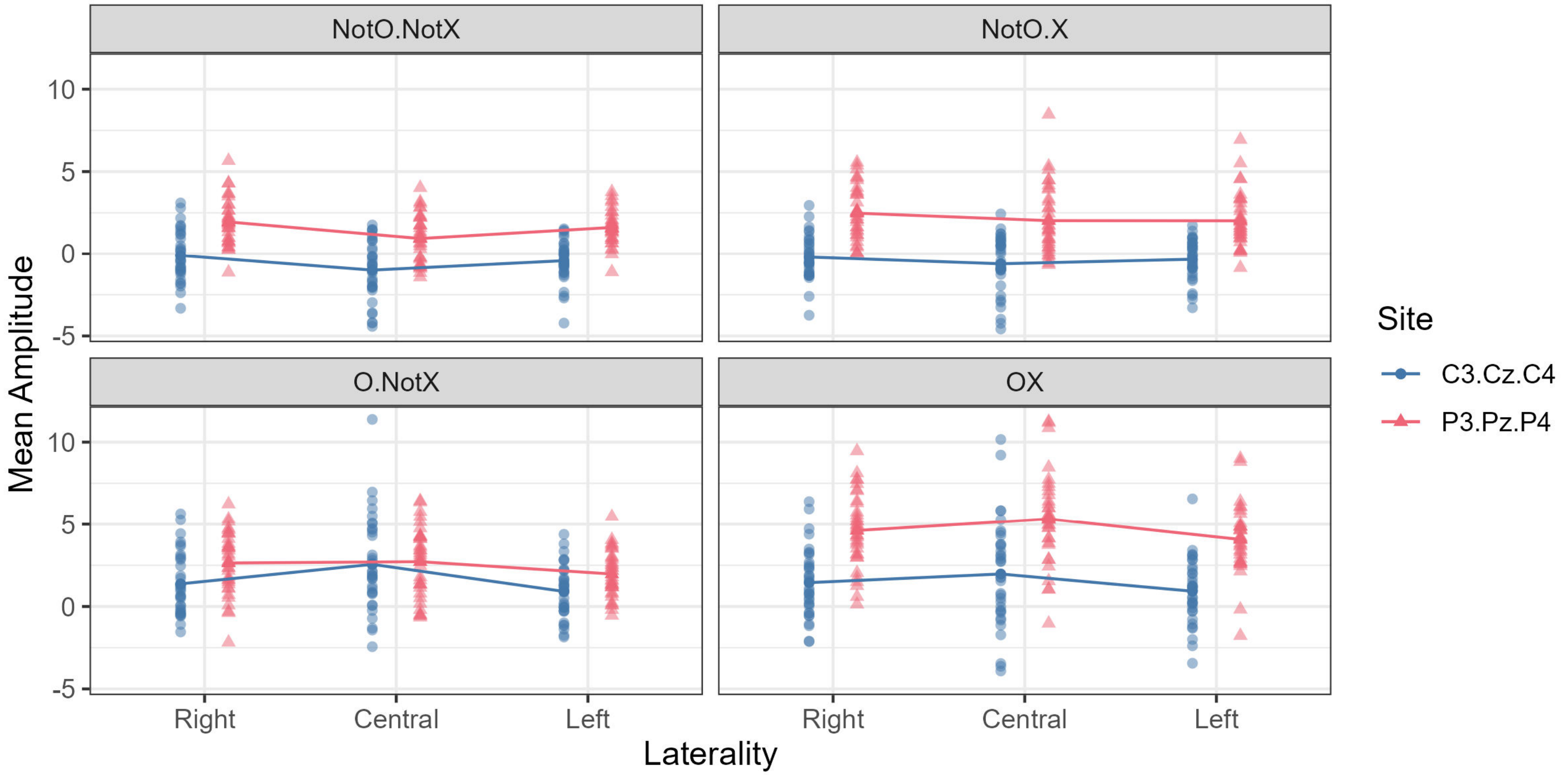

ERP Analysis (Face-CPT)

- **Continued P300** (300-500 ms)
- Following up on the 3-way interaction of Condition: Laterality: Site
    - **Condition:Laterality:Site $F(6, 198) = 7.971, p < .001$, np2 = .195**
- **3-way Interaction plot (**I created two types of interaction plot, each representing the same data, with different visualization**)**

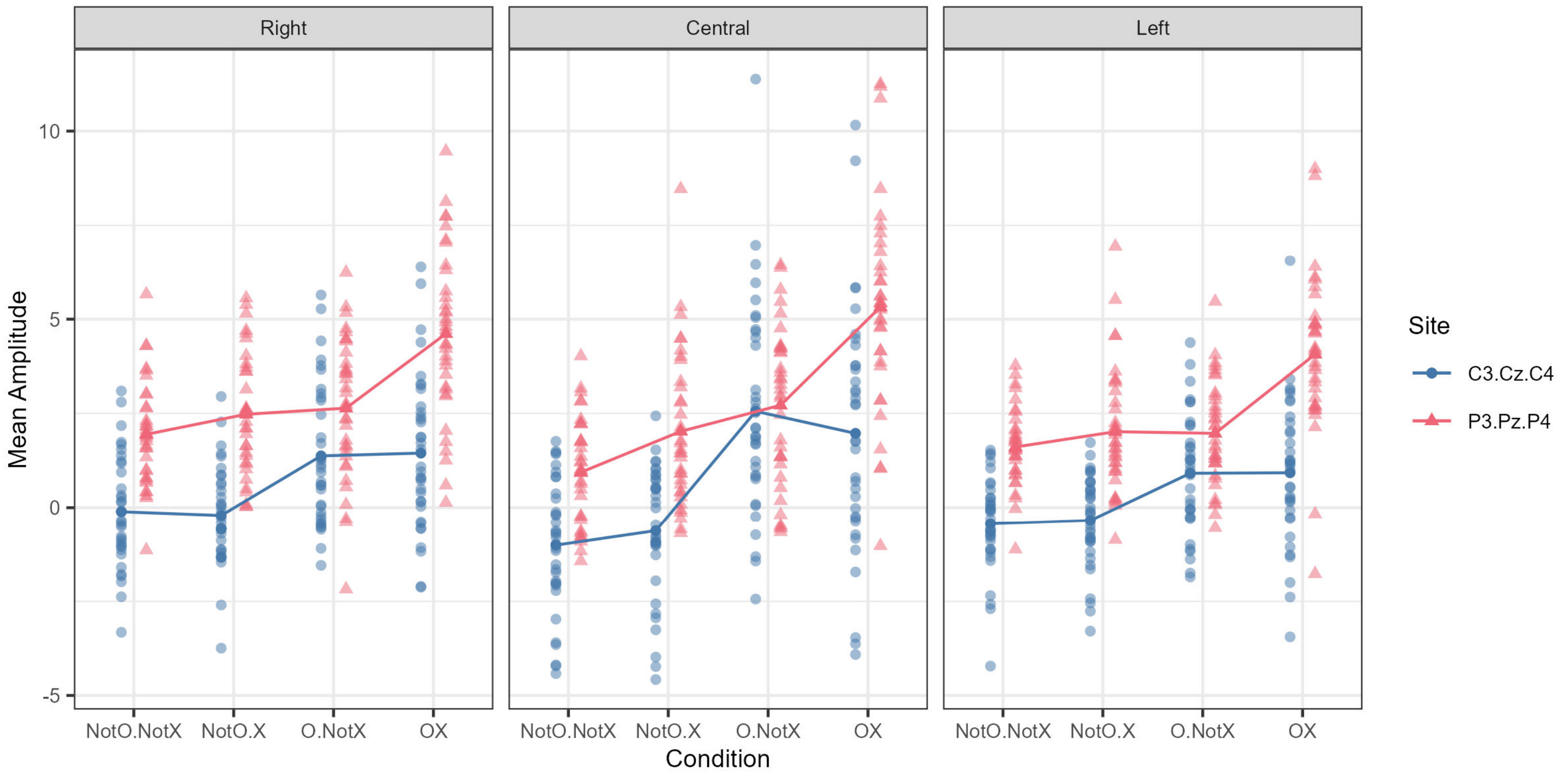

- **Plot 2:**

ERP Analysis (Face-CPT)

- **Continued CNV:** (1700-2000)
- If I put all conditions in the analysis to check their relationship further:
- 2*2 within and between design. Factors: Condition type (Stimulus 1= O, Stimulus 1≠ O), Group (ASD, Neurotypical). The studied electrode is Cz:
  - **Group F(1, 33) = 6.947, p = .013, np2 = .174**
  - **condtype F(1, 33) = 32.692, p < .001, np2 = .498**
  - **Group:condtype F(1, 33) = 3.689, p = .063, np2 = .101**
  - For the group factor, the means are:

Group	Mean	SE	95% CI
ASD	-0.22	0.12	[-0.47, 0.03]
TD	-0.68	0.12	[-0.92, -0.43]

Marginal means estimated at Group

For the condition type , the means are:

condtype	Mean	SE	95% CI
FirstO	-1.00	0.14	[-1.29, -0.71]
NFirstO	0.10	0.12	[-0.14, 0.34]

Marginal means estimated at Group

Letter CPT, P300

Condition	Mean	SE	95% CI
NotO.NotX	0.80	0.15	[0.50, 1.11]
NotO.X	1.11	0.17	[0.77, 1.46]
O.NotX	3.35	0.33	[2.69, 4.02]
OX	4.21	0.38	[3.43, 4.98]

Marginal means estimated at Condition

Face CPT, P300

Condition	Mean	SE	95% CI
NotO.NotX	0.49	0.15	[0.19, 0.80]
NotO.X	0.89	0.18	[0.52, 1.26]
O.NotX	2.03	0.23	[1.56, 2.50]
OX	3.06	0.33	[2.40, 3.72]

Marginal means estimated at Condition

Face CPT, CNV

Group	Mean	SE	95% CI
ASD	-0.22	0.12	[-0.47, 0.03]
TD	-0.68	0.12	[-0.92, -0.43]

Marginal means estimated at Group

Letter CPT, CNV

Group	Mean	SE	95% CI
ASD	-0.55	0.21	[-0.98, -0.12]
TD	-0.74	0.20	[-1.16, -0.33]

Marginal means estimated at Group

samanehsadat.dastgheib@uni-jena.de

Overall ERPs for letters OX

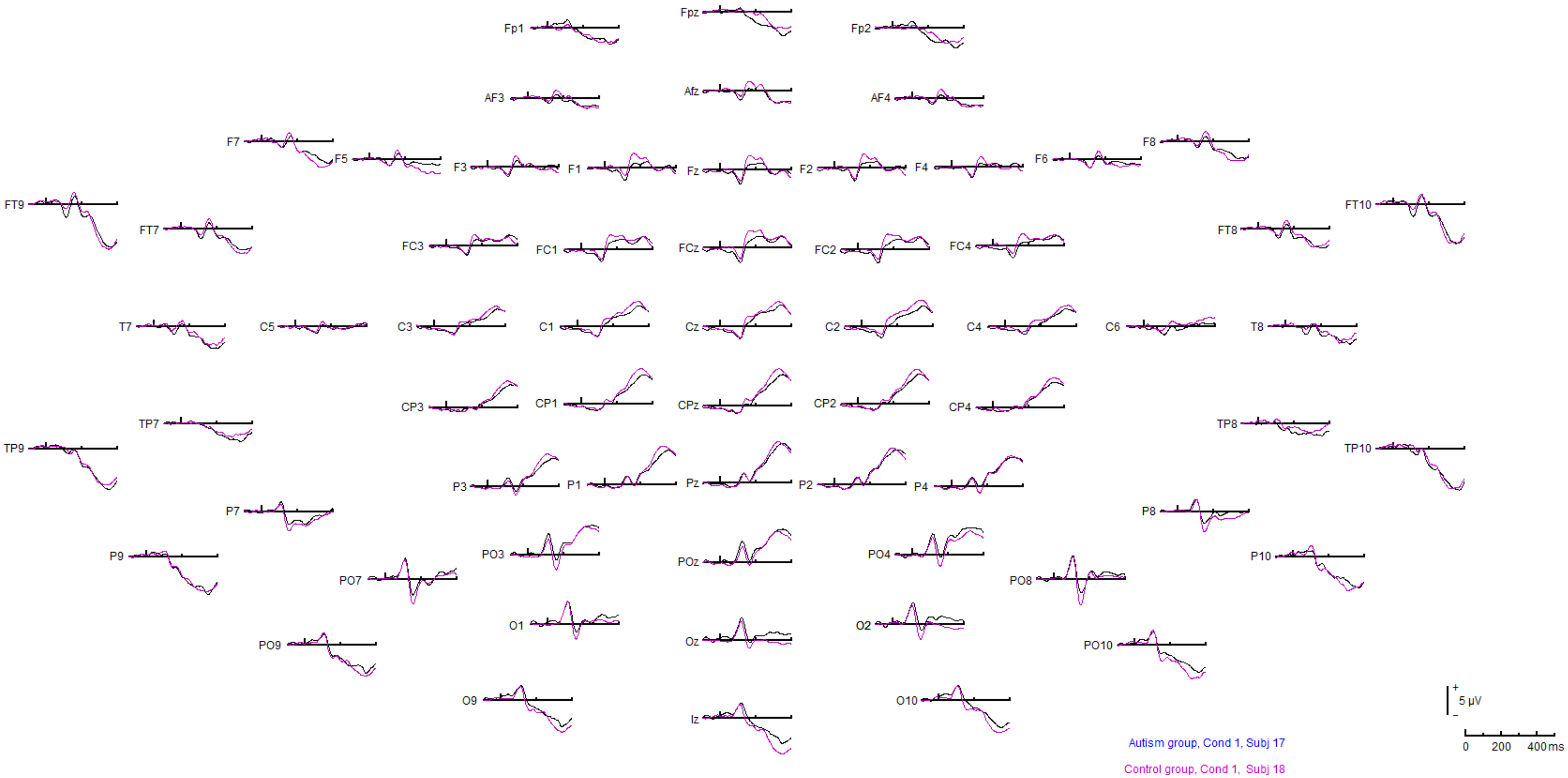

Overall ERPs for faces OX

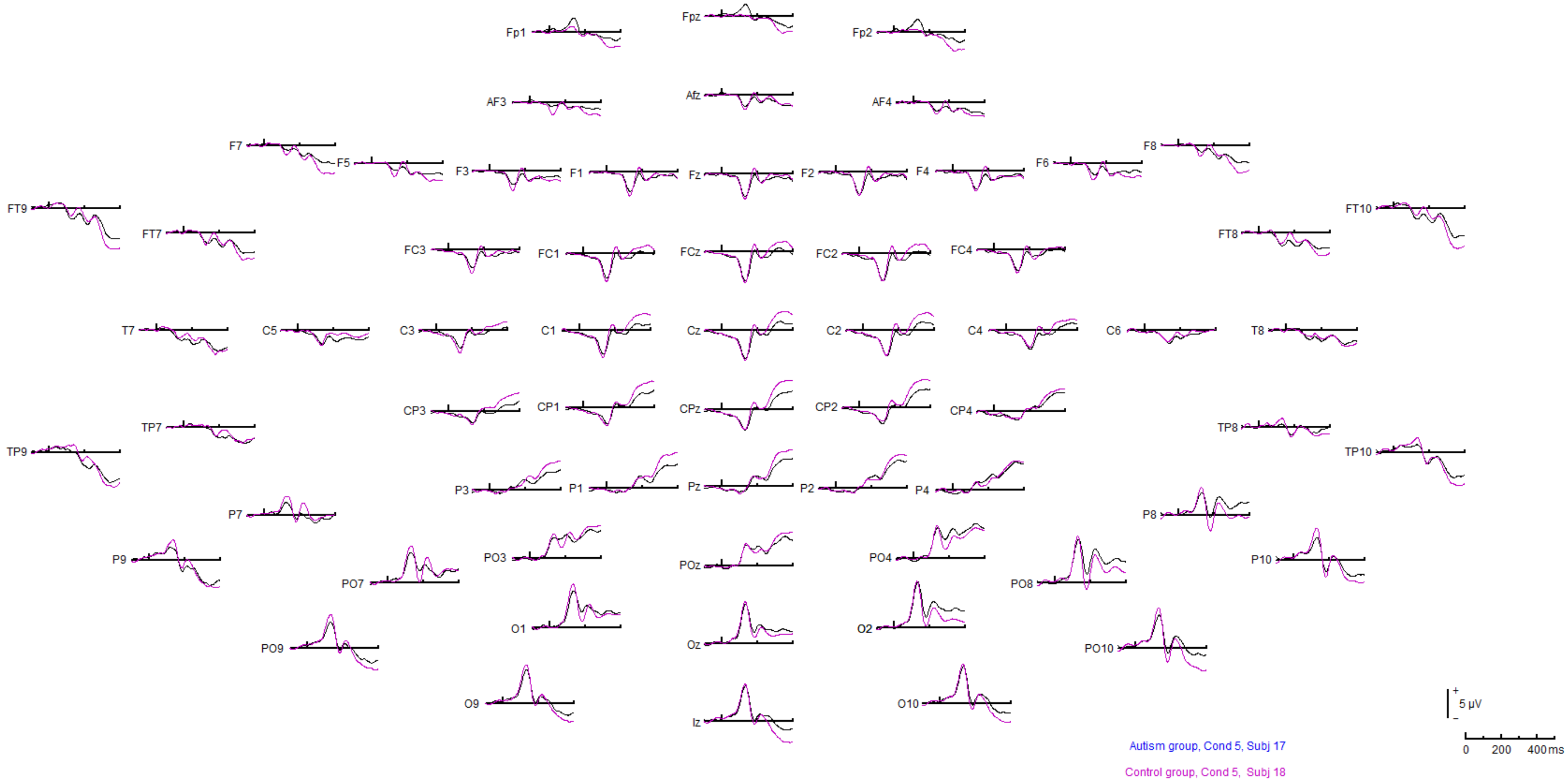

Overall ERPs for letters Not O but X

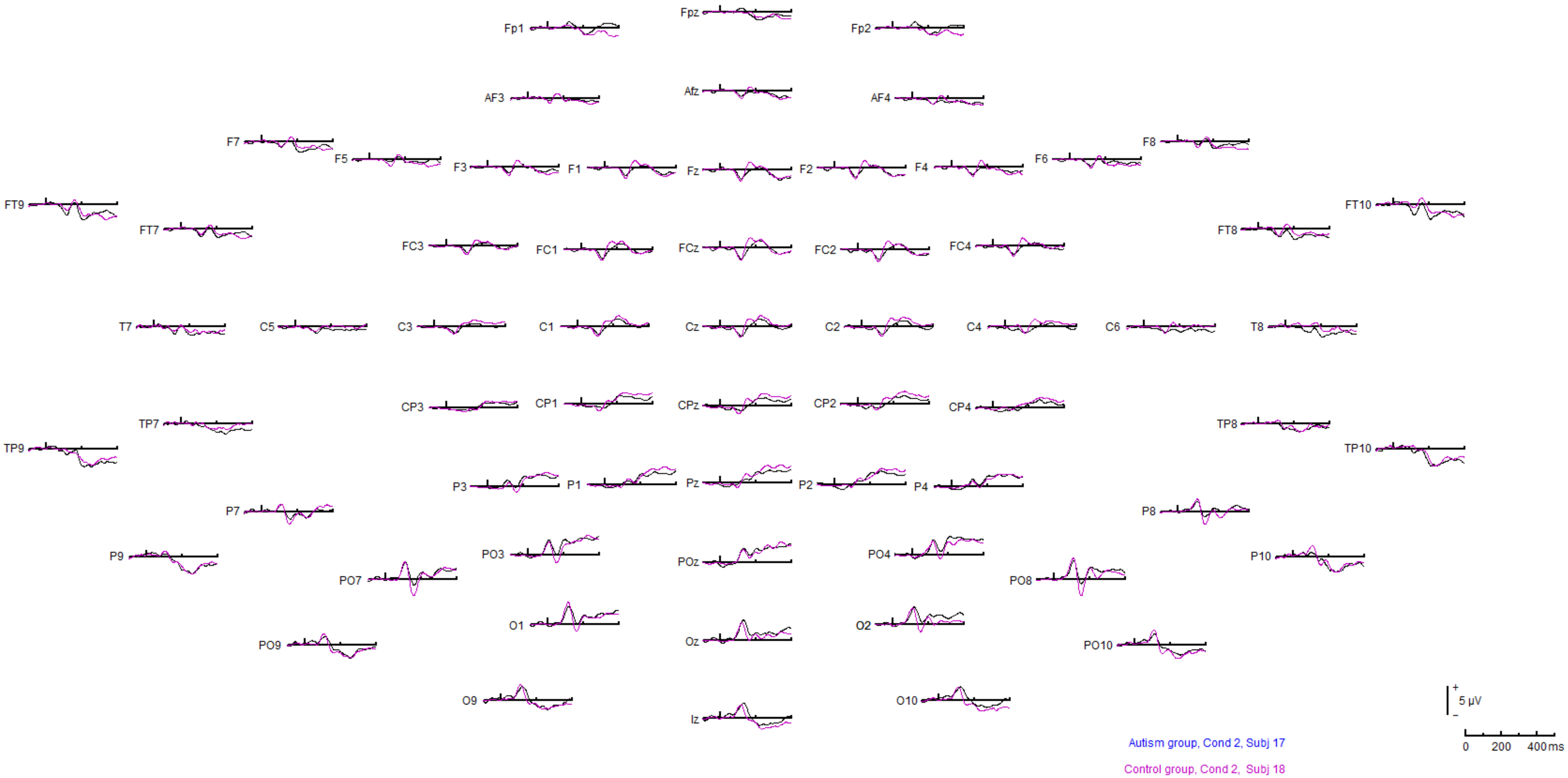

Overall ERPs for faces Not O but X

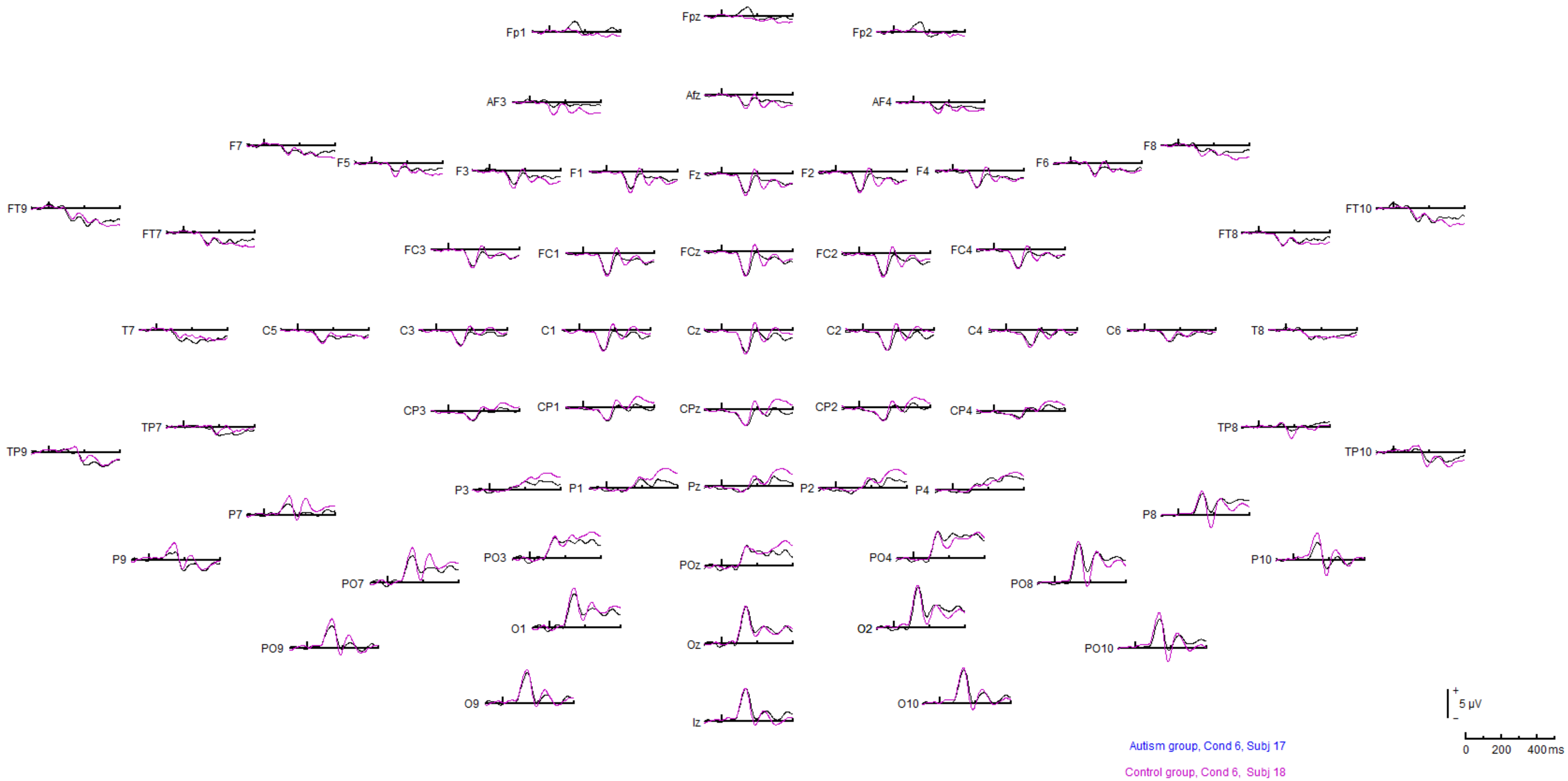

Overall ERPs for letters O but Not X

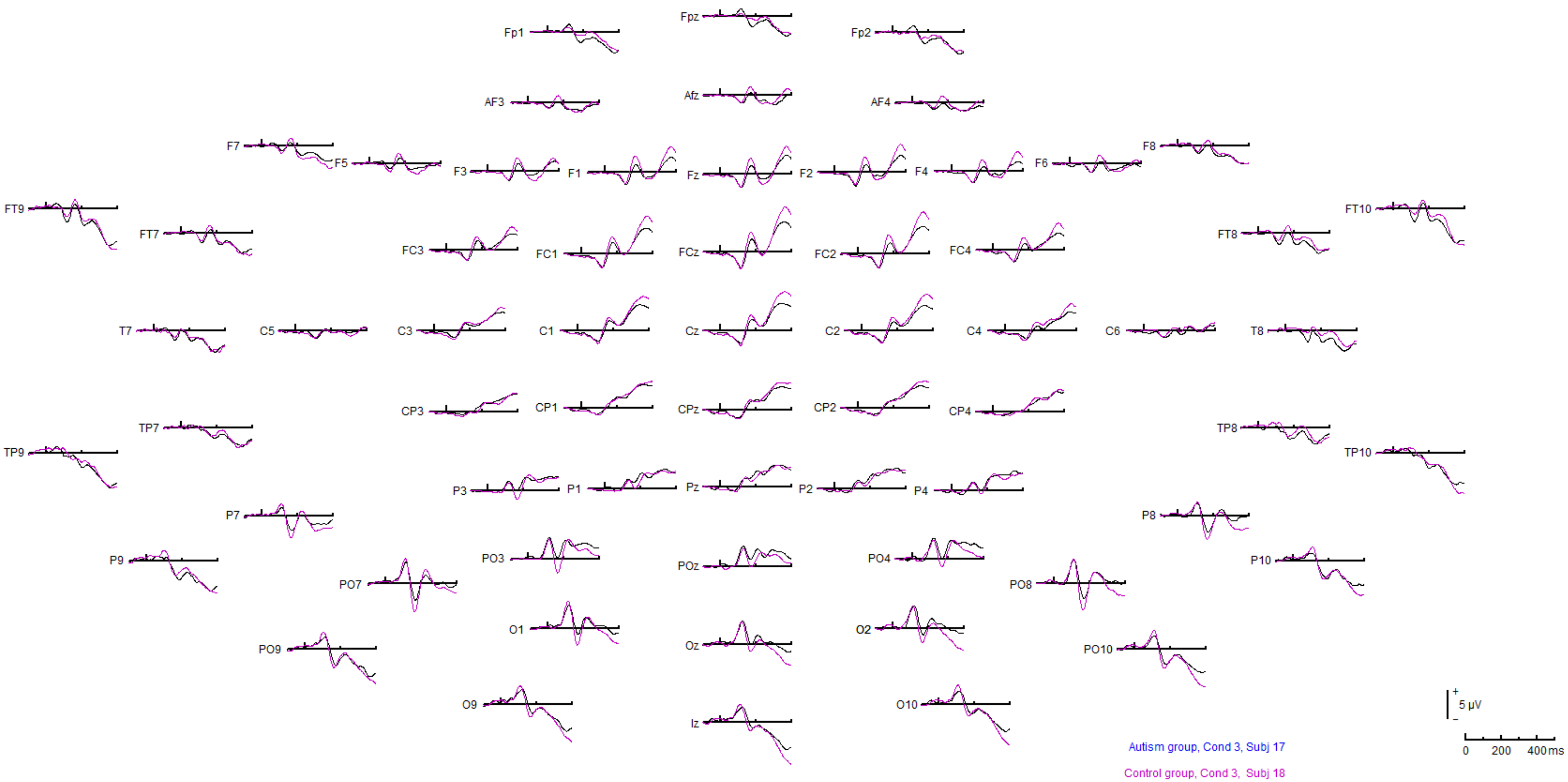

Overall ERPs for faces O but Not X

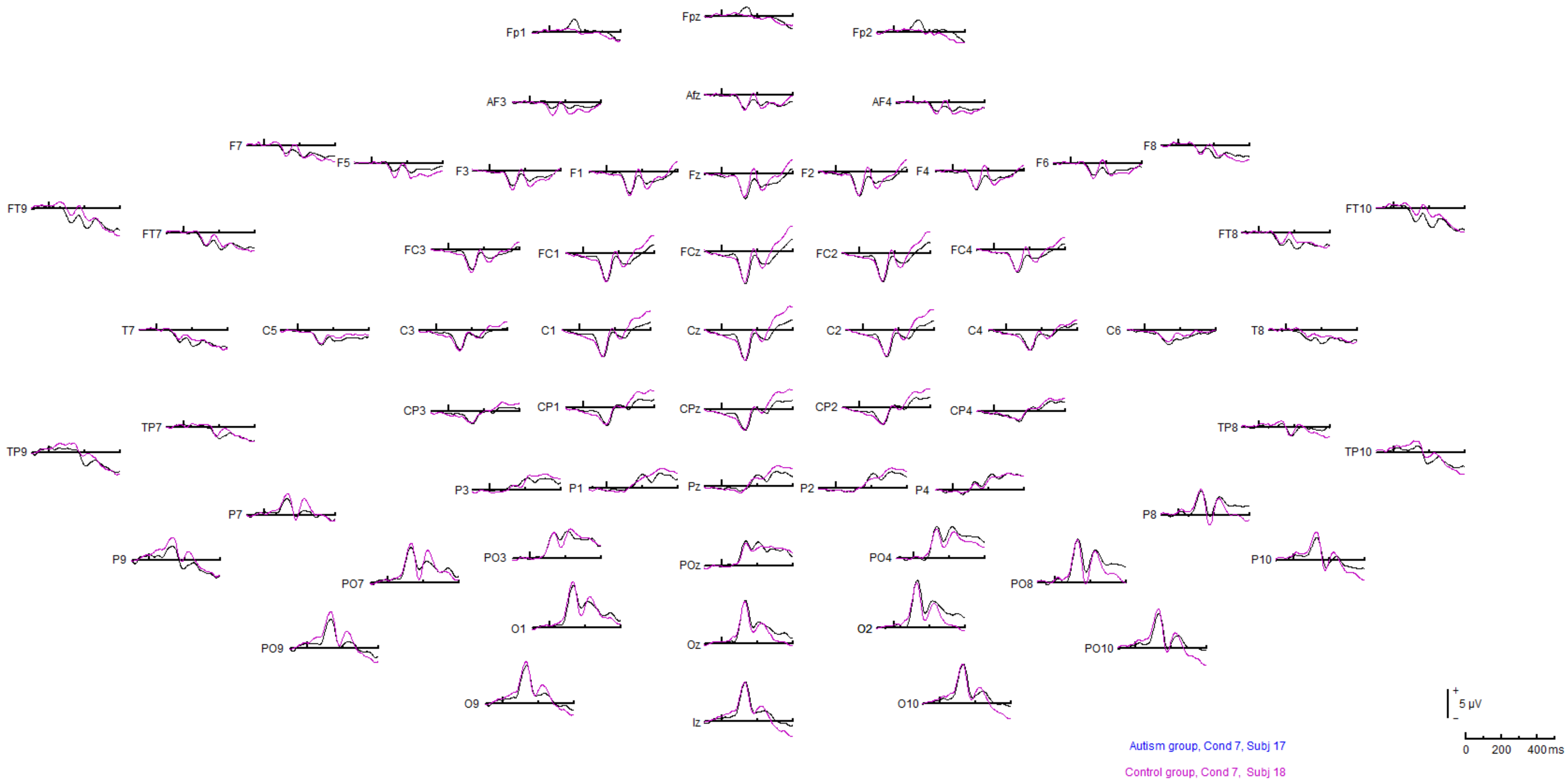

Overall ERPs for letters Not O Not X

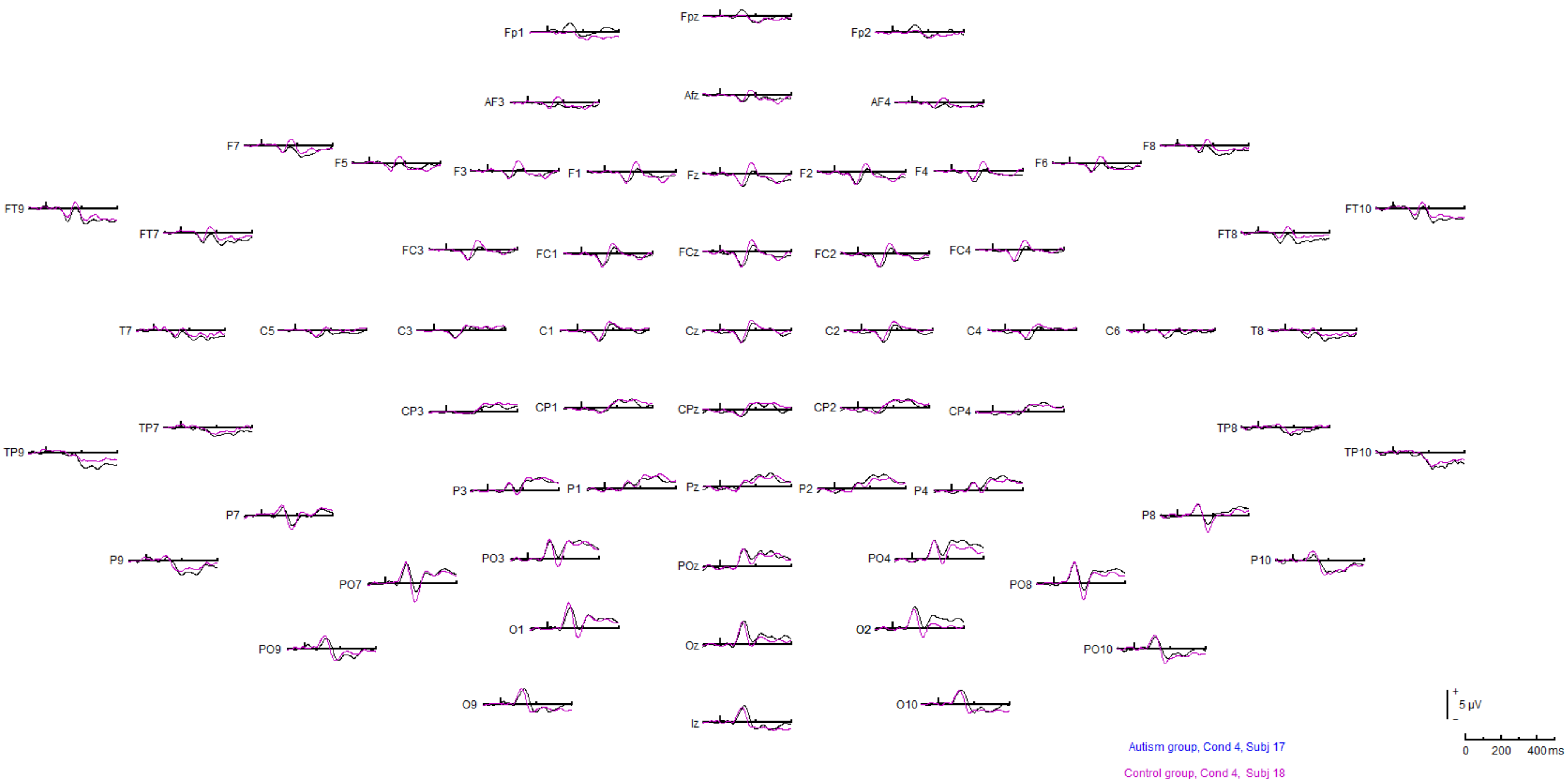

Overall ERPs for faces Not O Not X

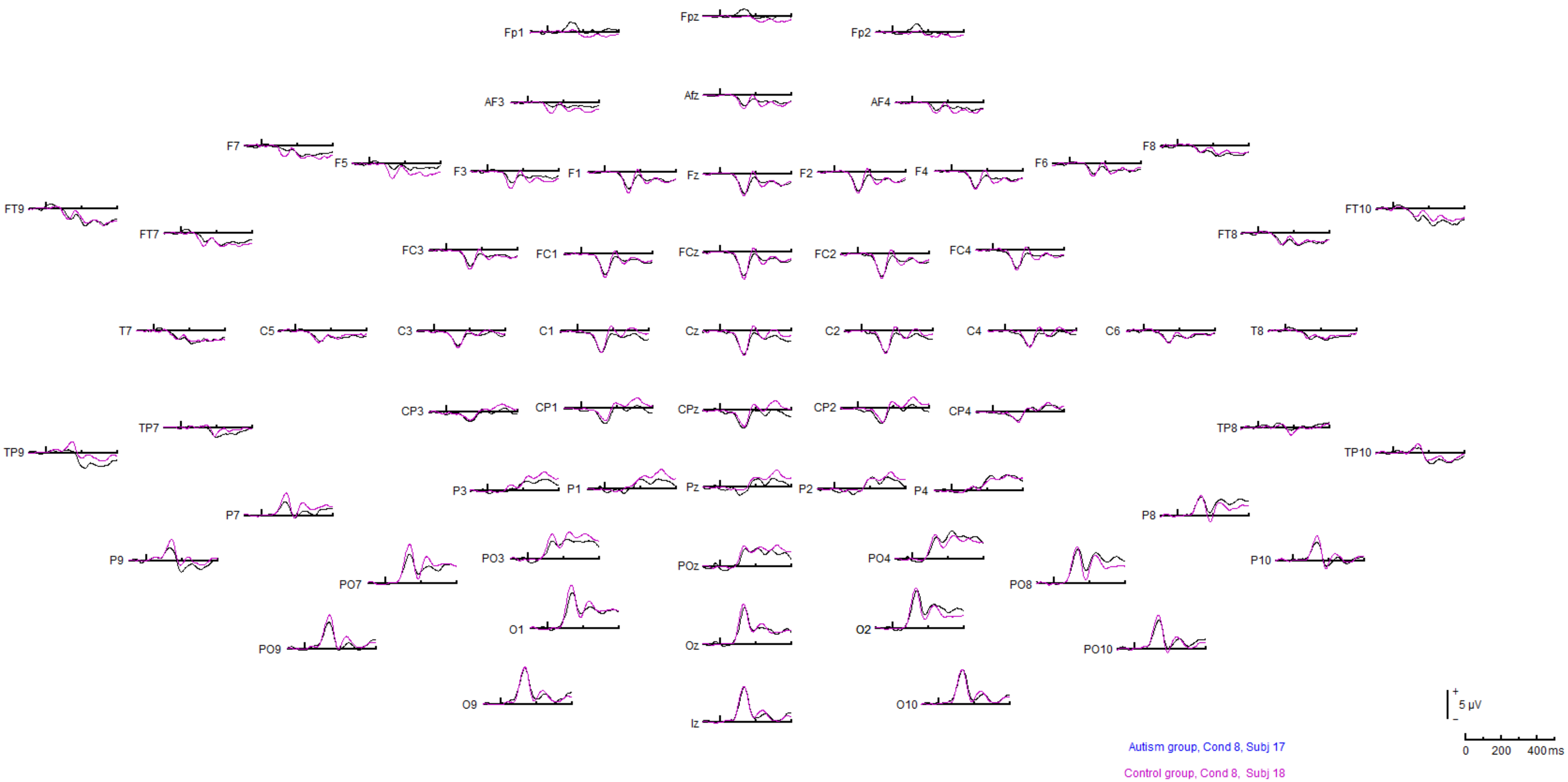

ERPs for the 4 letter conditions at Cz in autism vs control

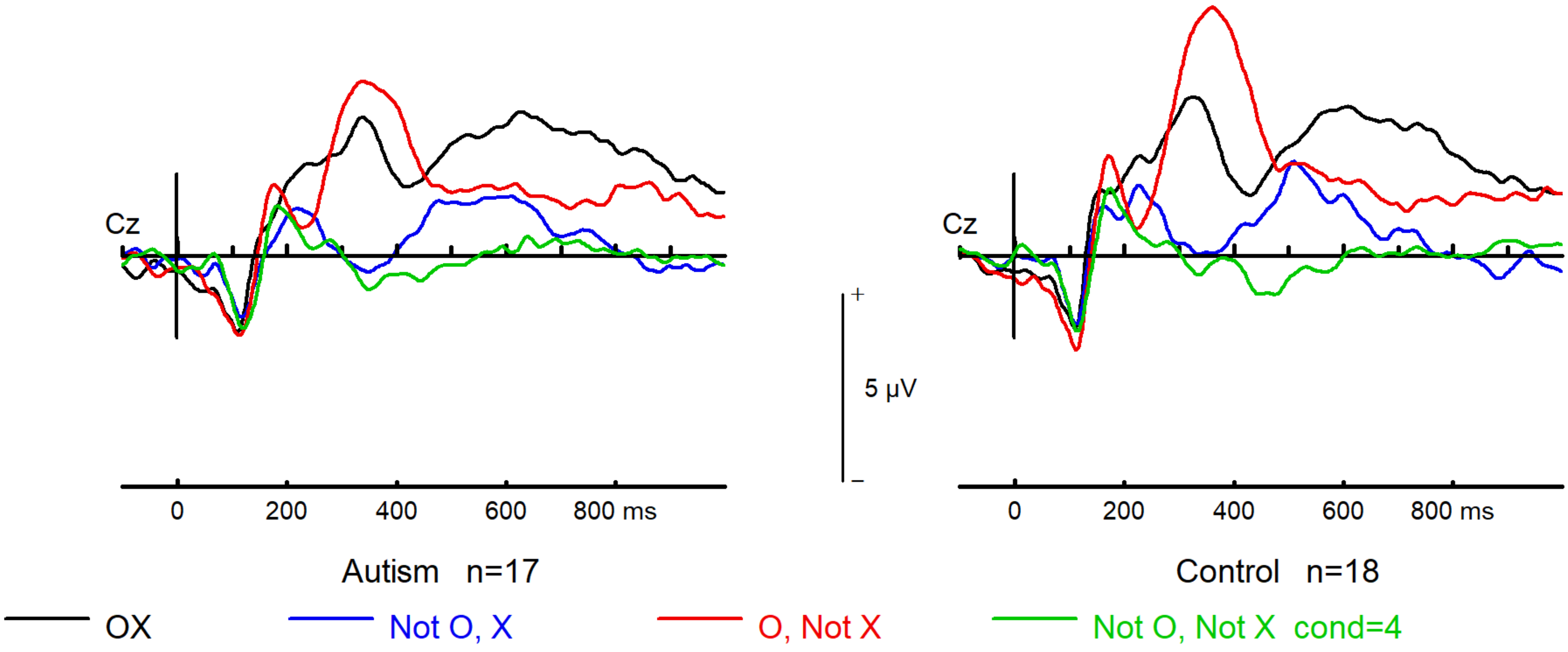

ERPs for the 4 face conditions at Cz in autism vs control

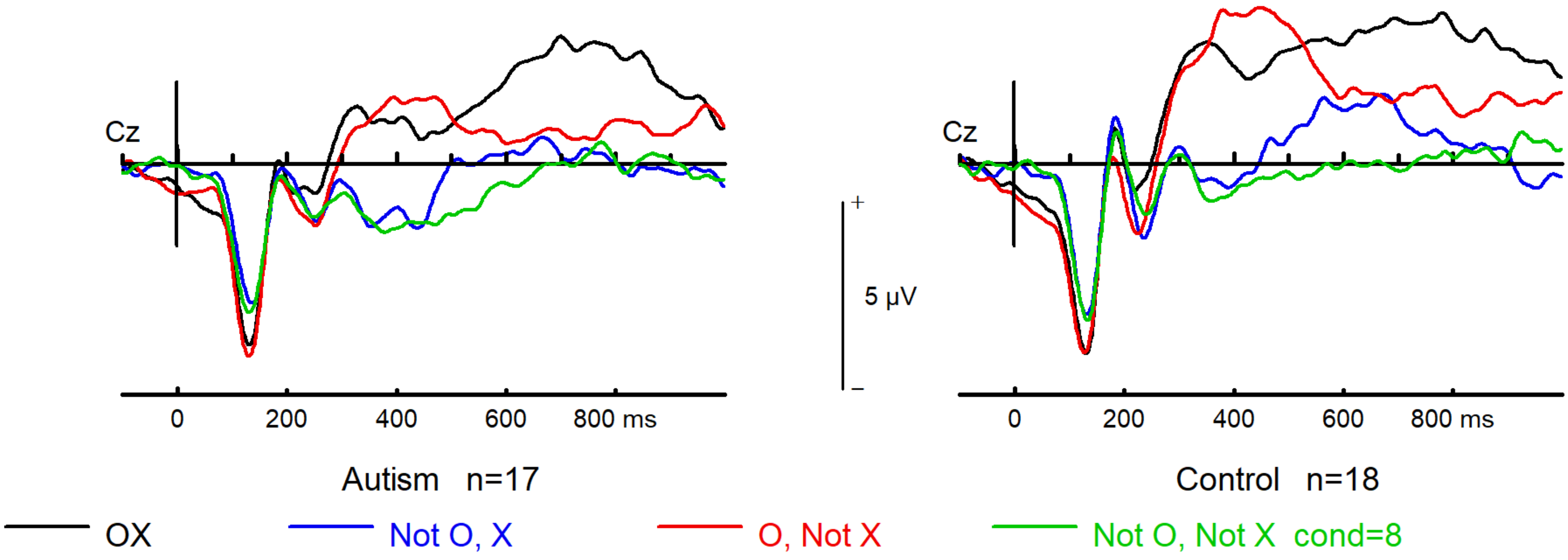

ERPs for the 4 letter conditions at Pz in autism vs control

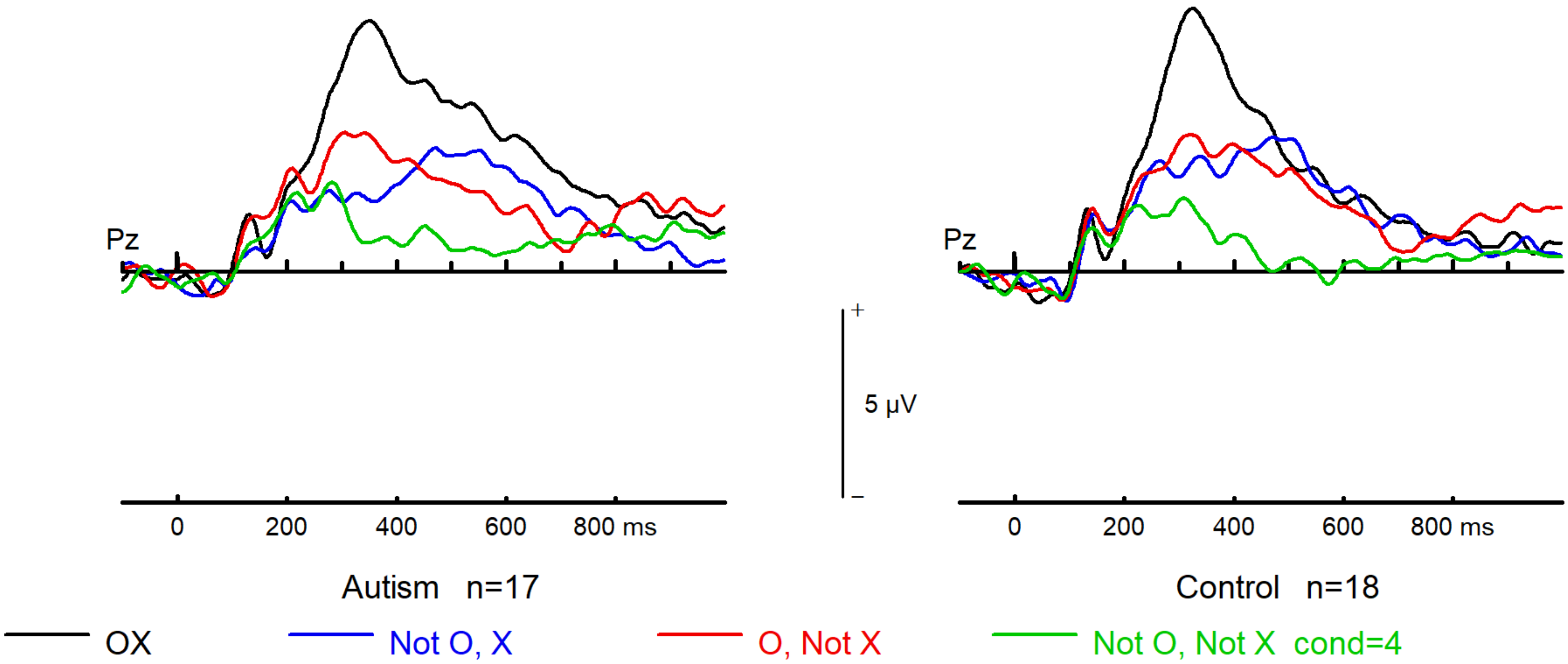

ERPs for the 4 face conditions at Pz in autism vs control

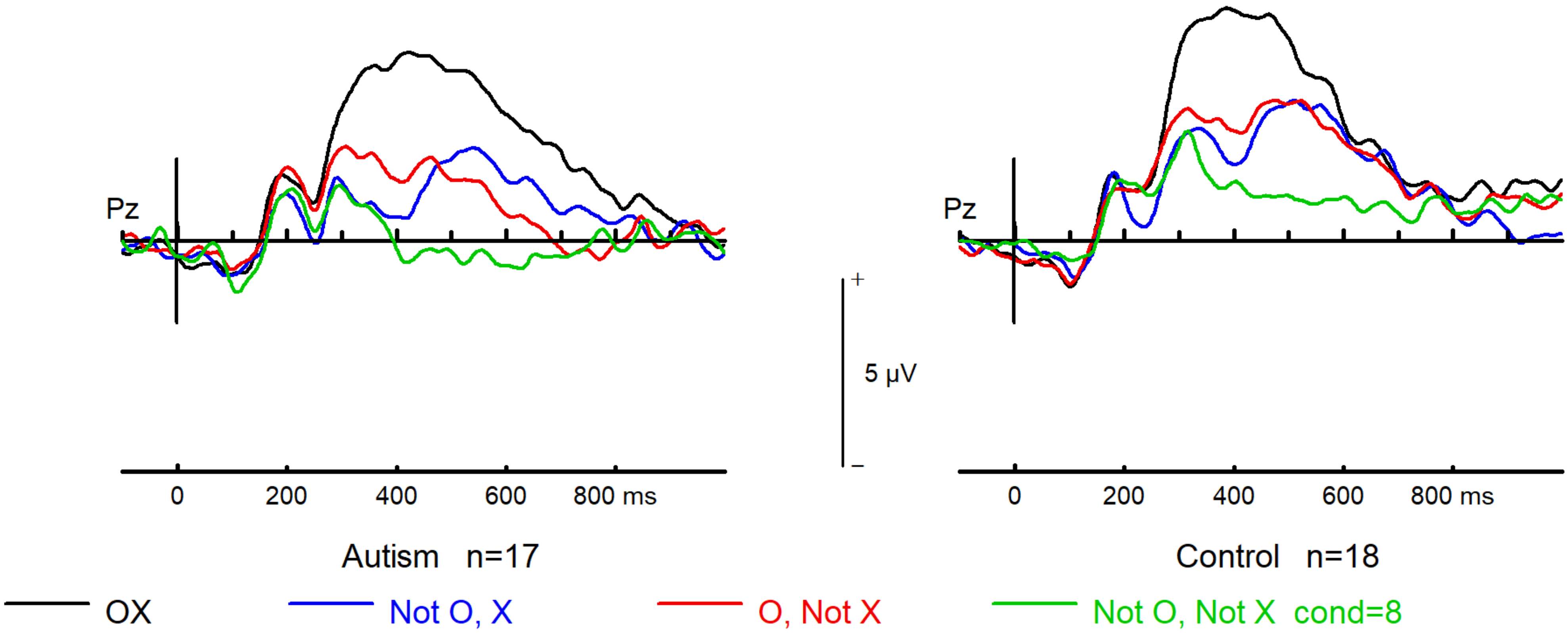

Condition= Letters OX

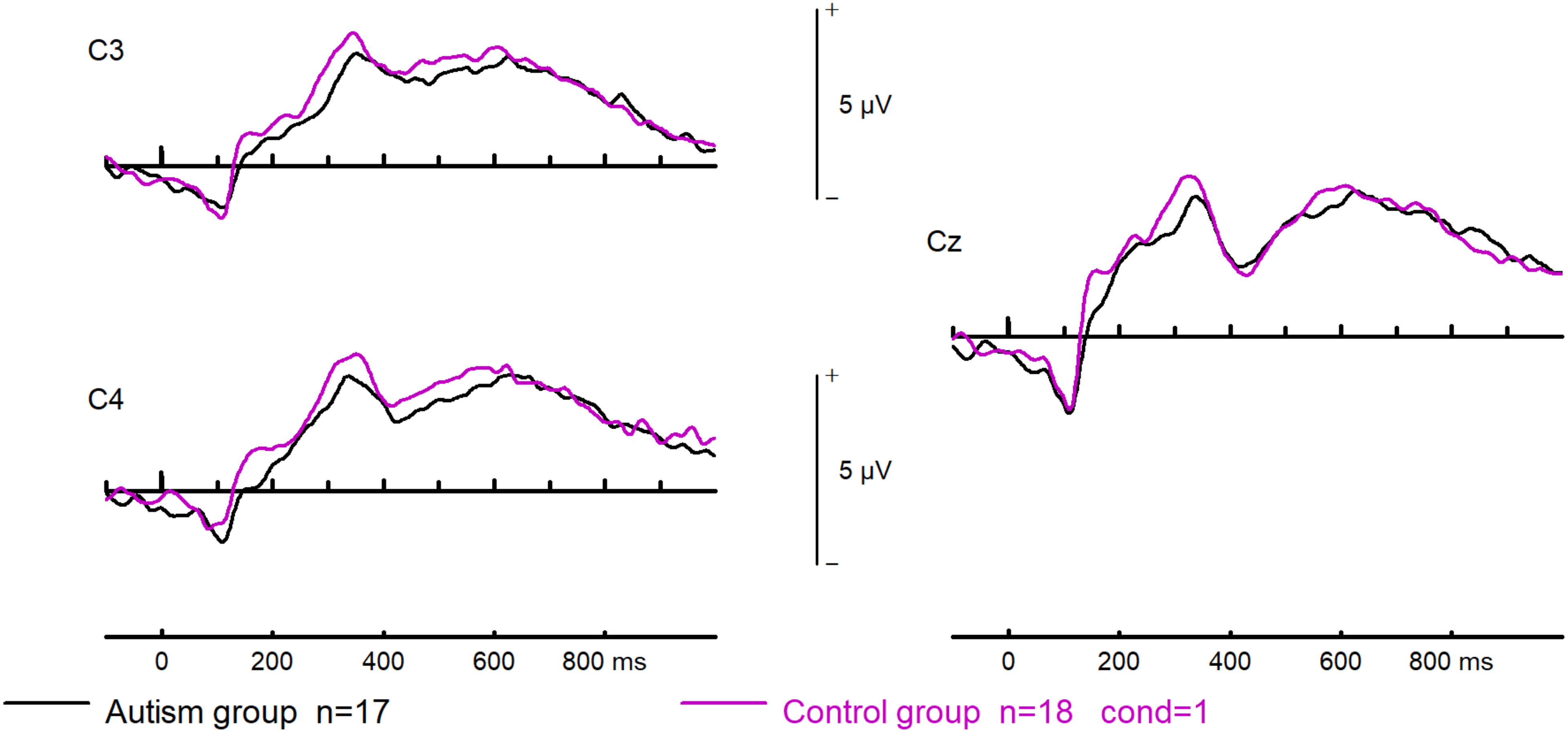

Condition= Letters Not O, X

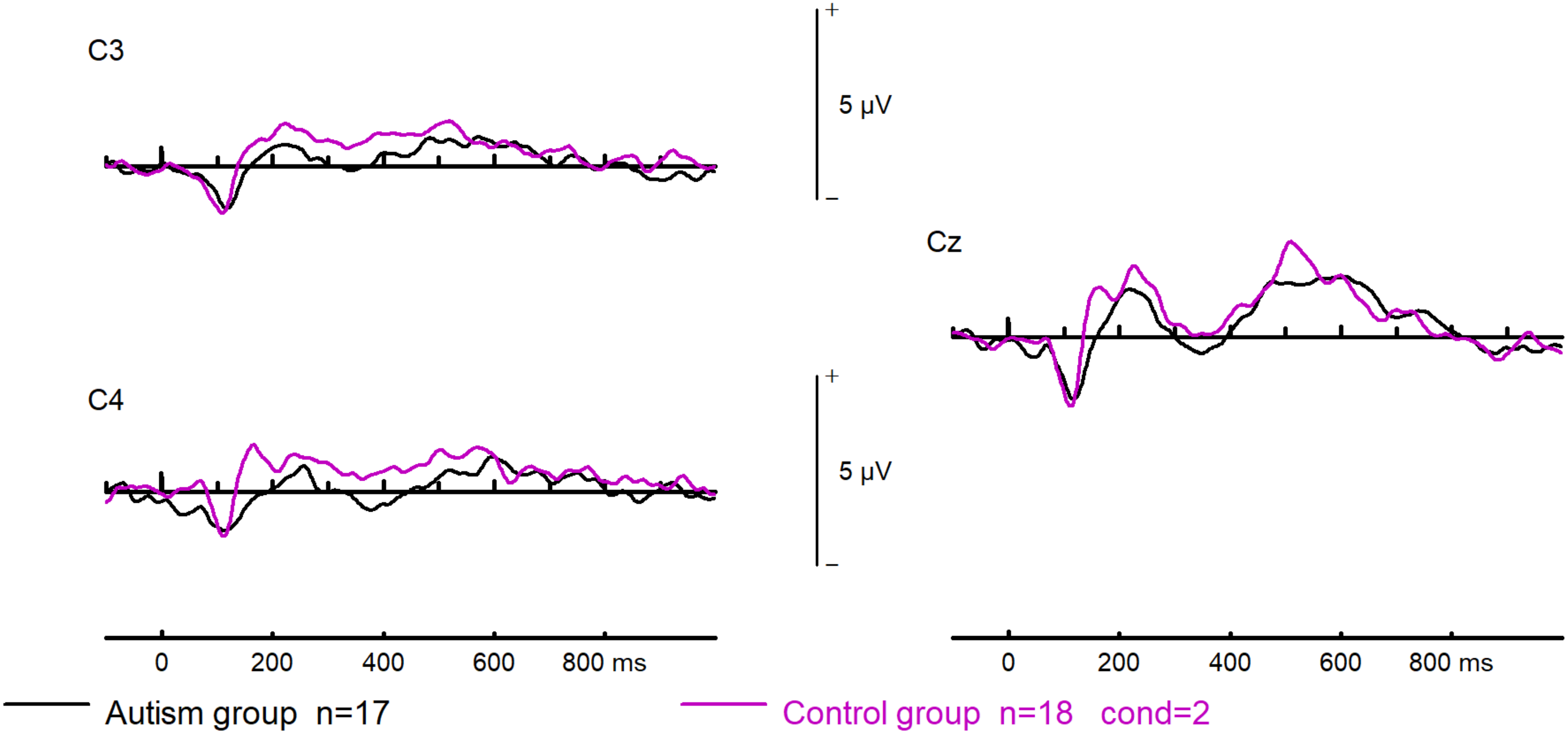

Condition= Letters O, NotX

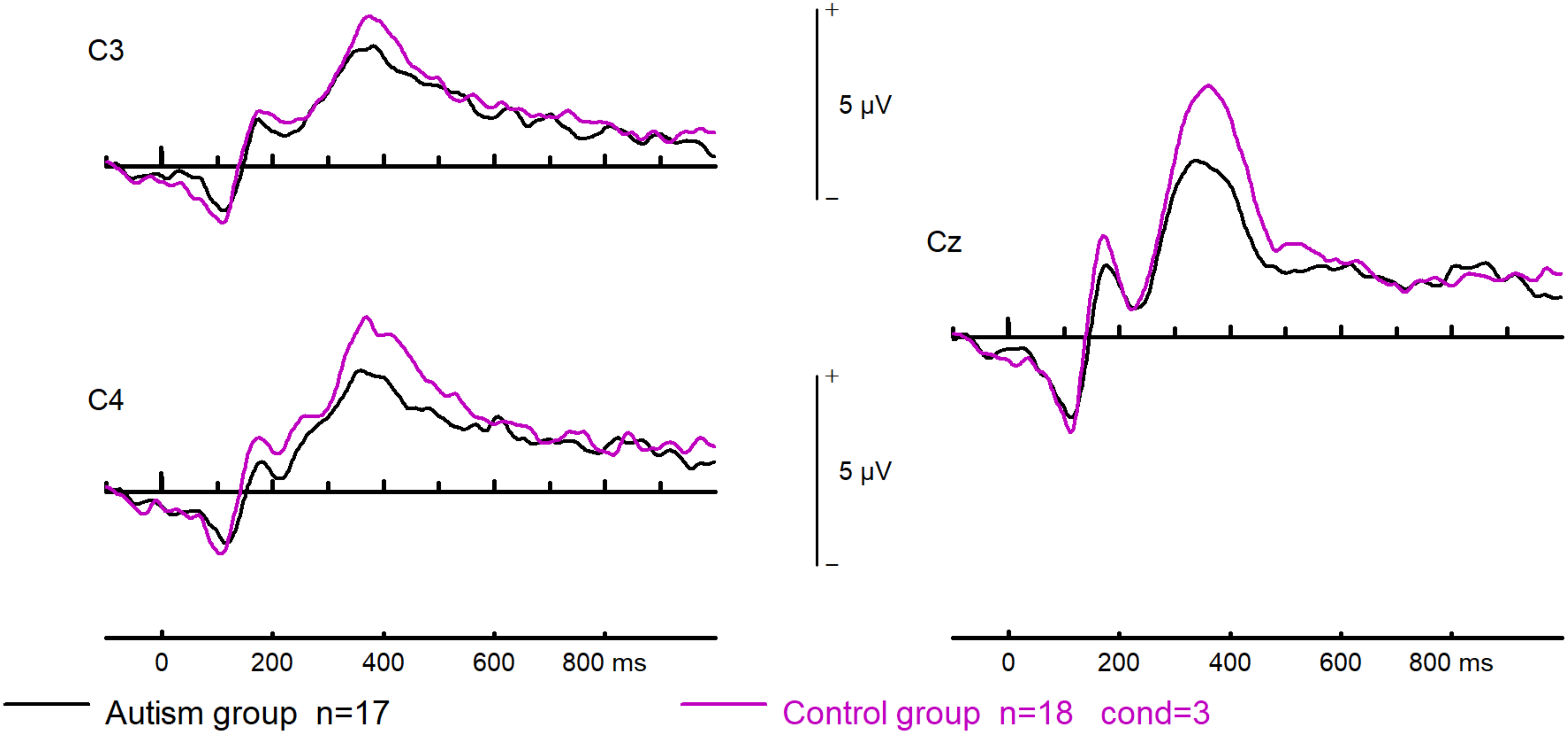

Condition= Letters NotO, NotX

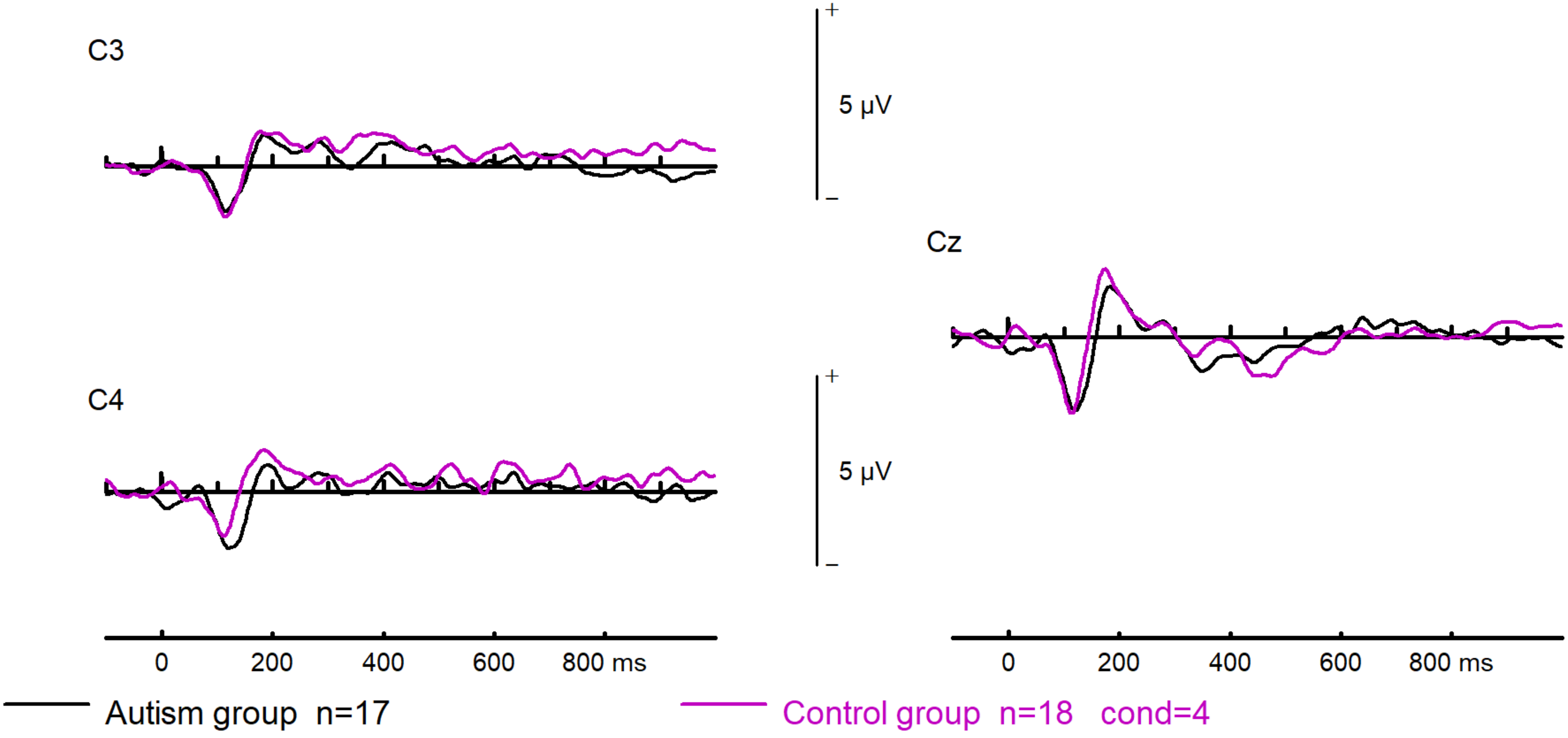

Condition= Faces OX

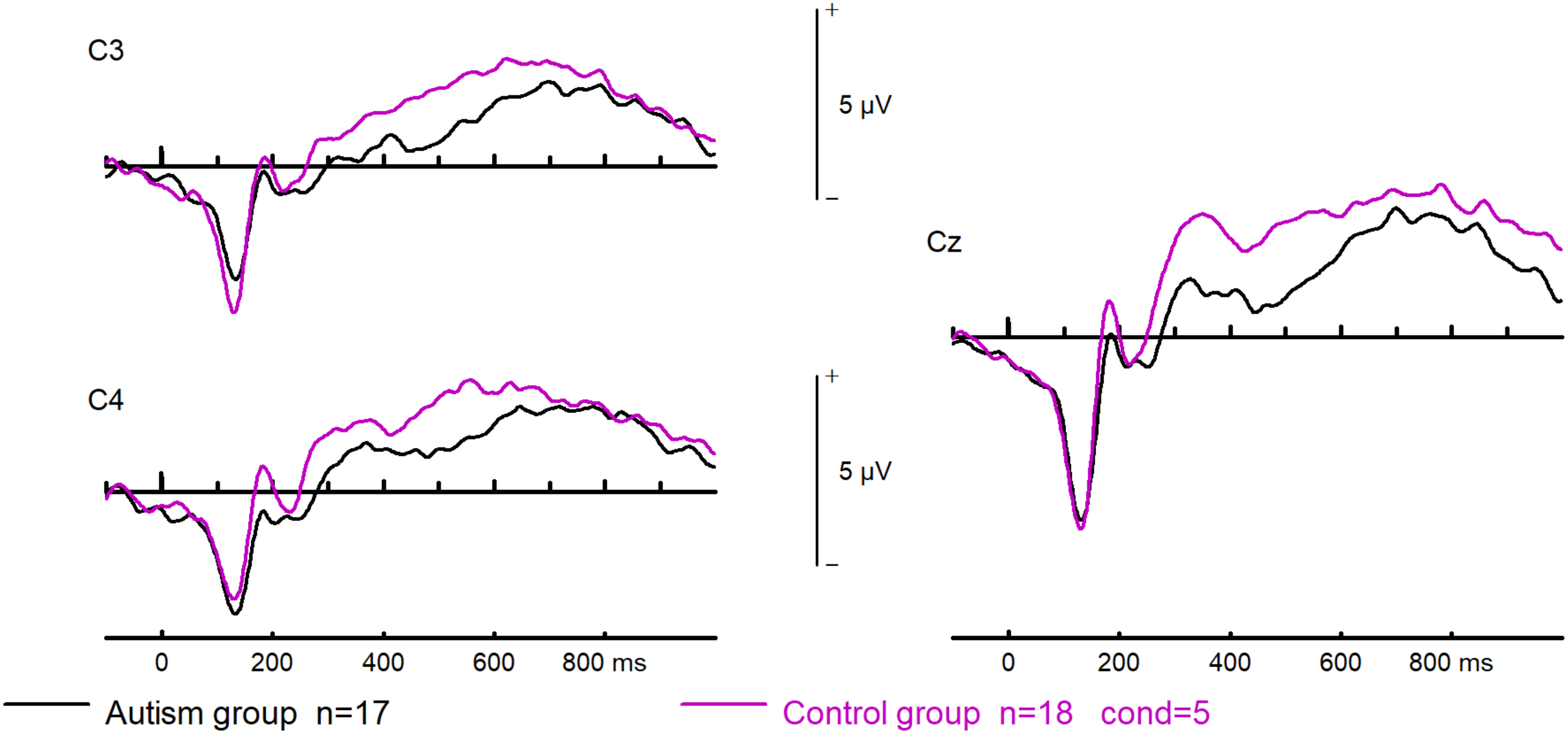

Condition= Faces NotO,X

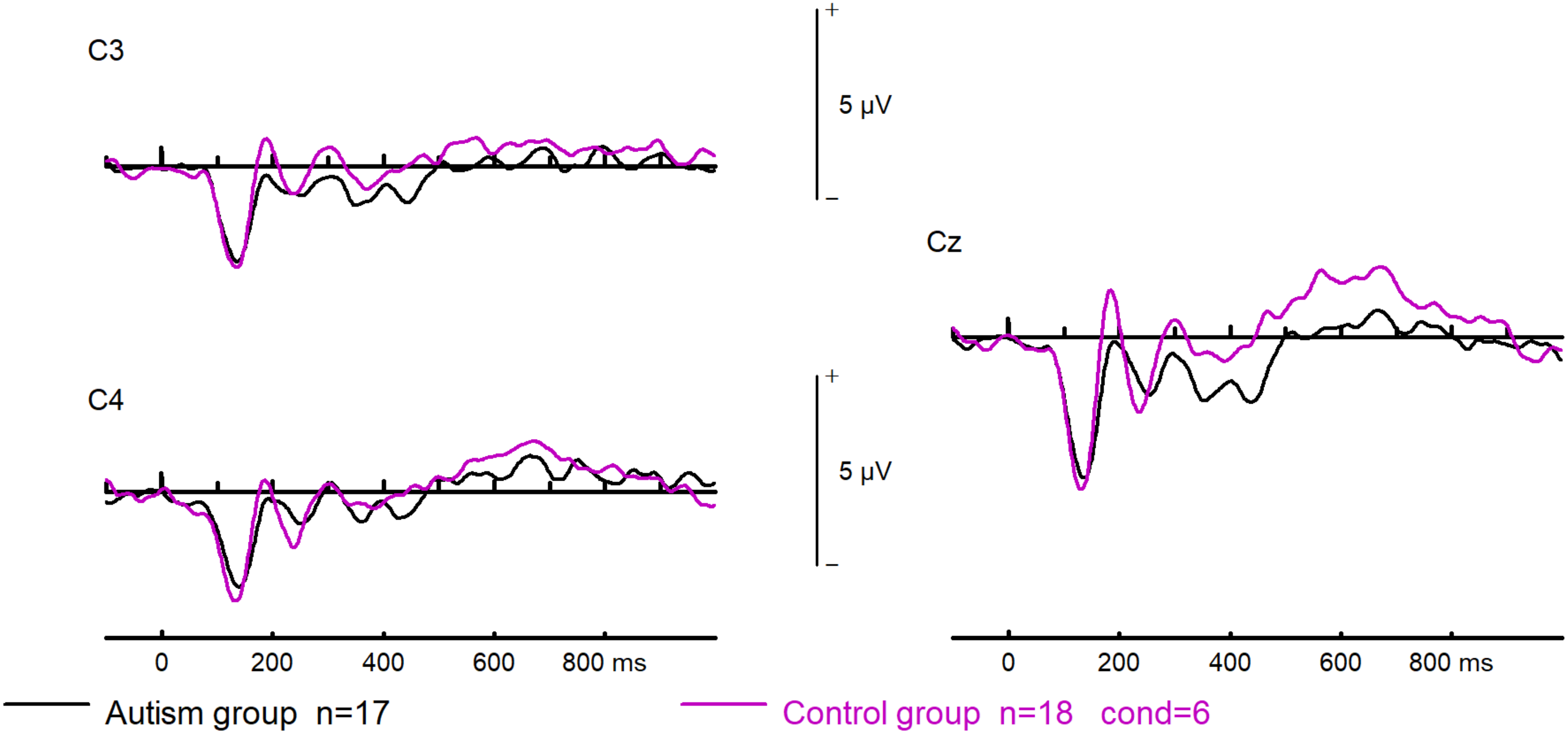

Condition= Faces O, Not X

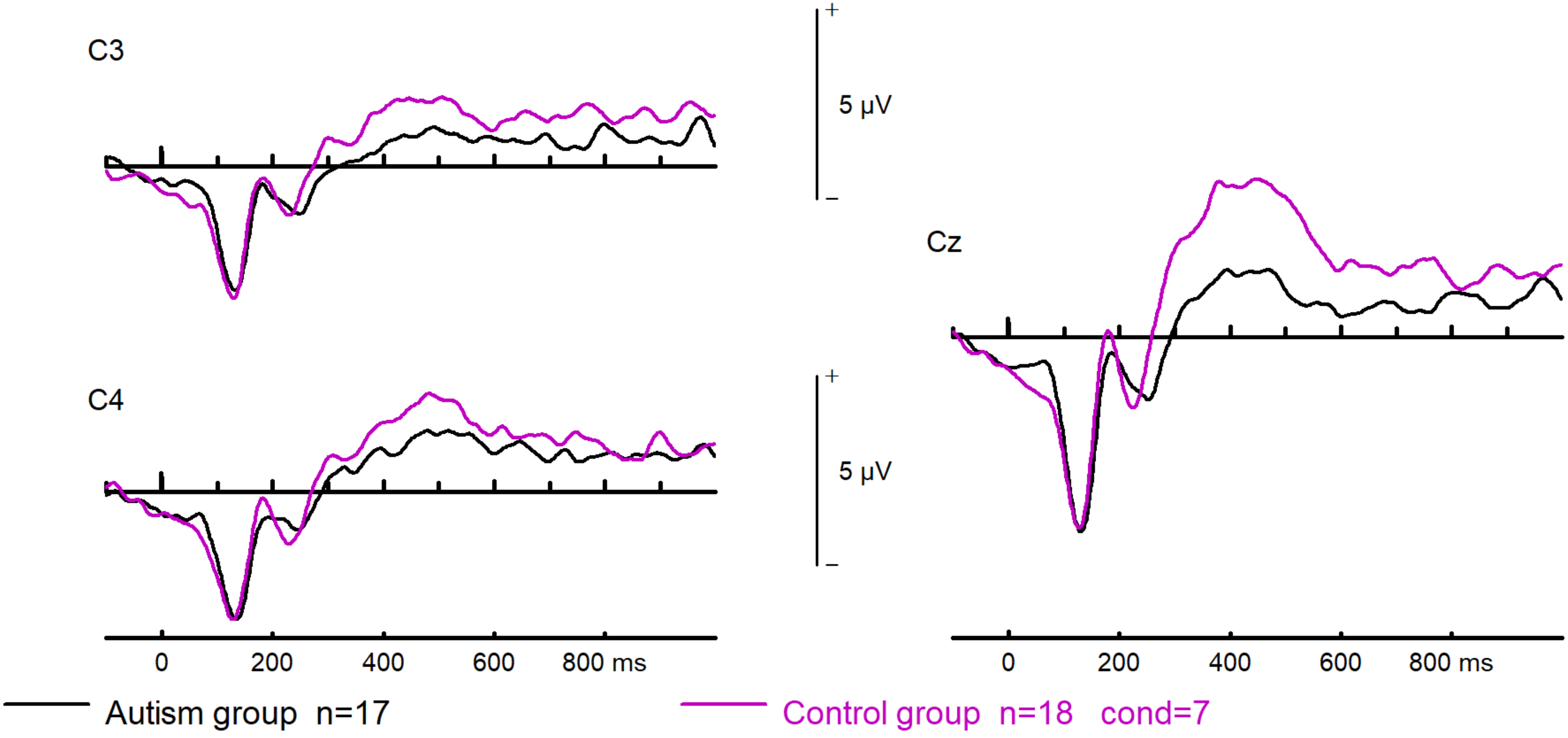

Condition= Faces NotO, NotX

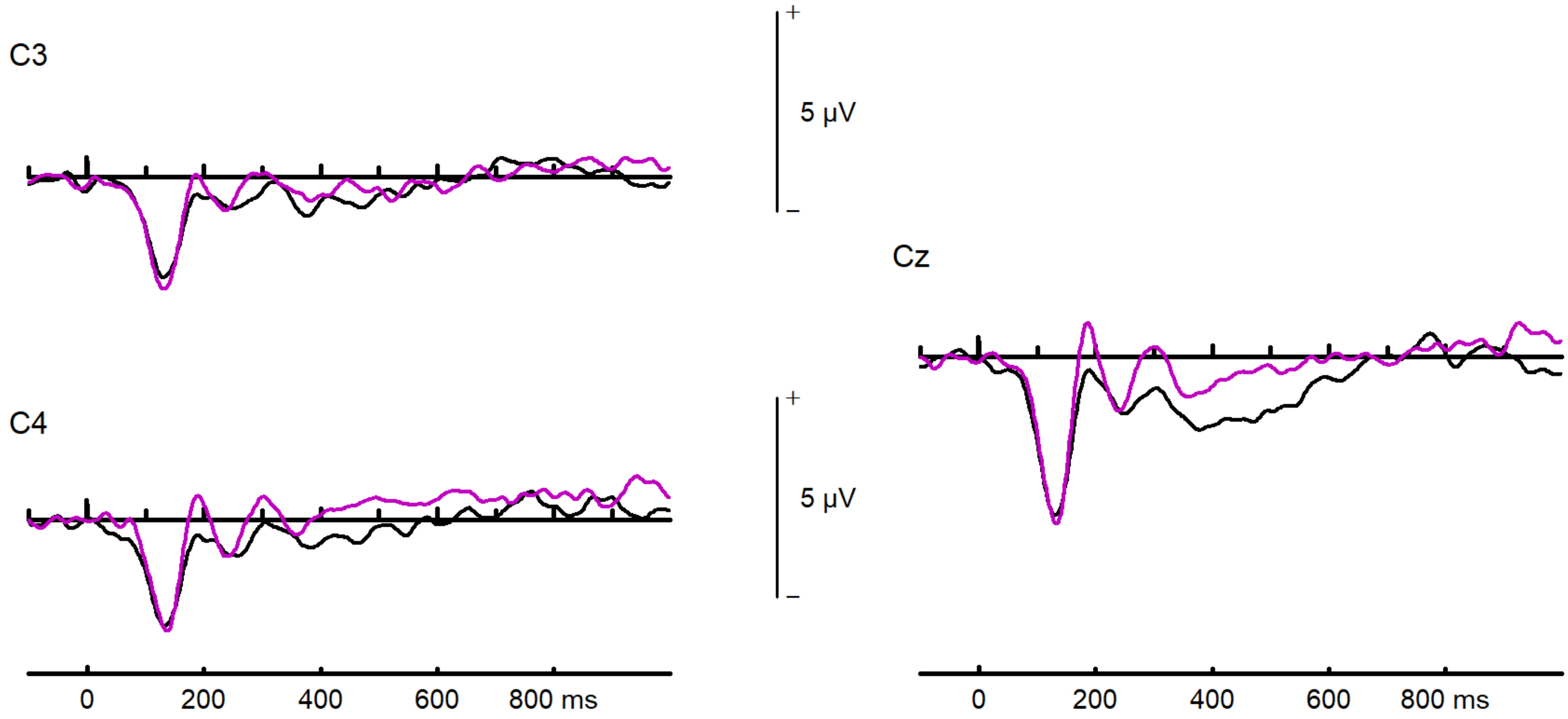

Autism group n=17 — Control group n=18 cond=8

Condition= Letters OX

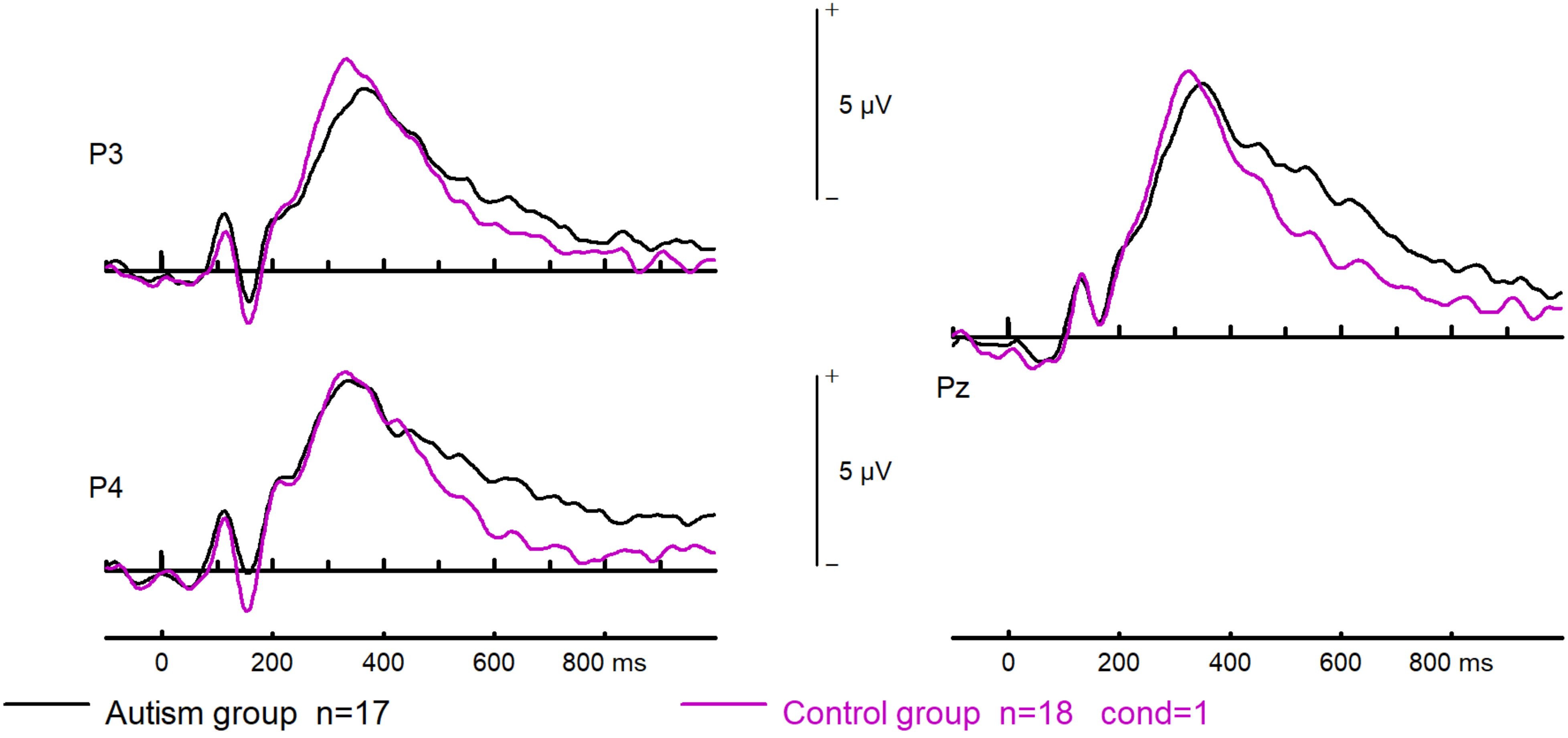

Condition= Letters Not O, X

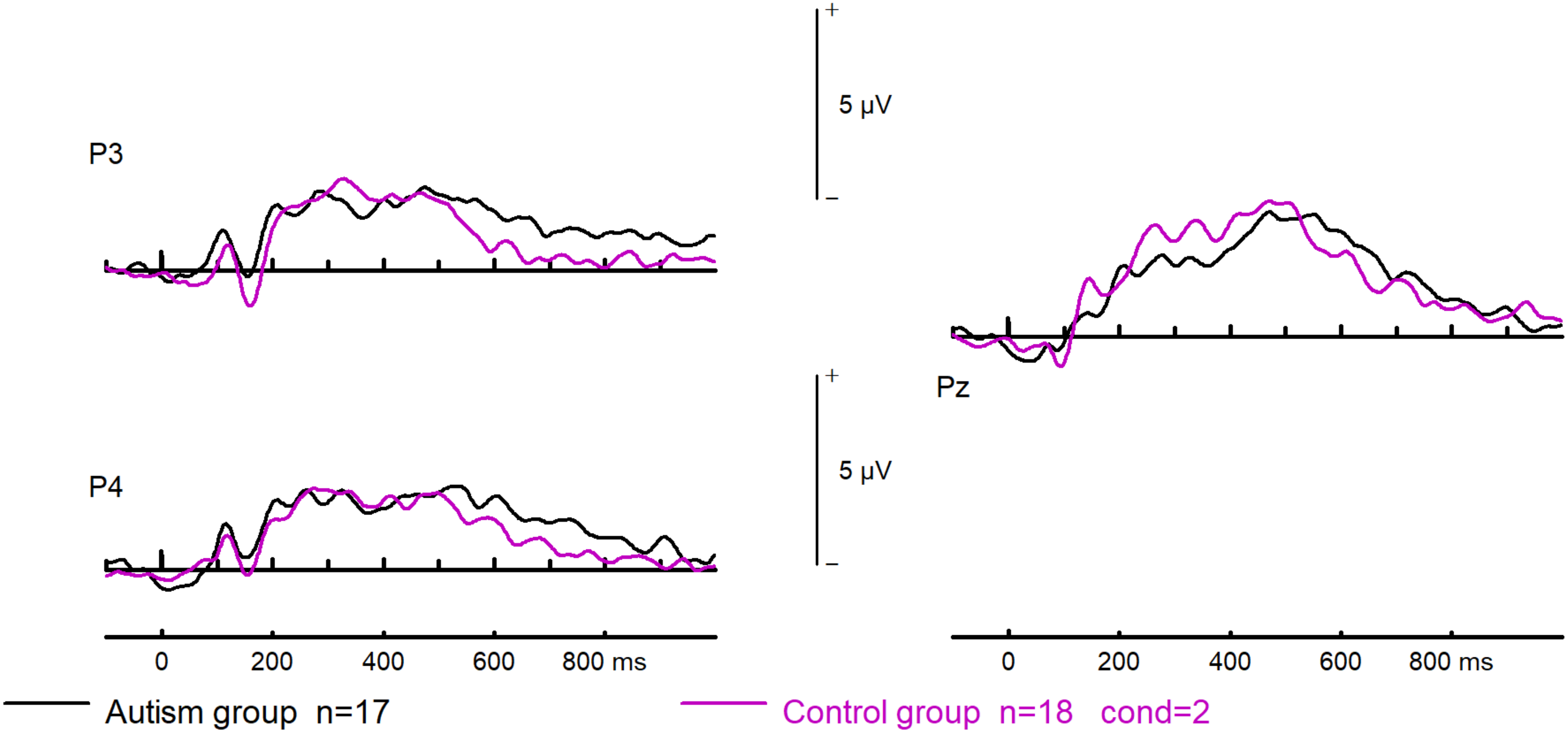

Condition= Letters O, NotX

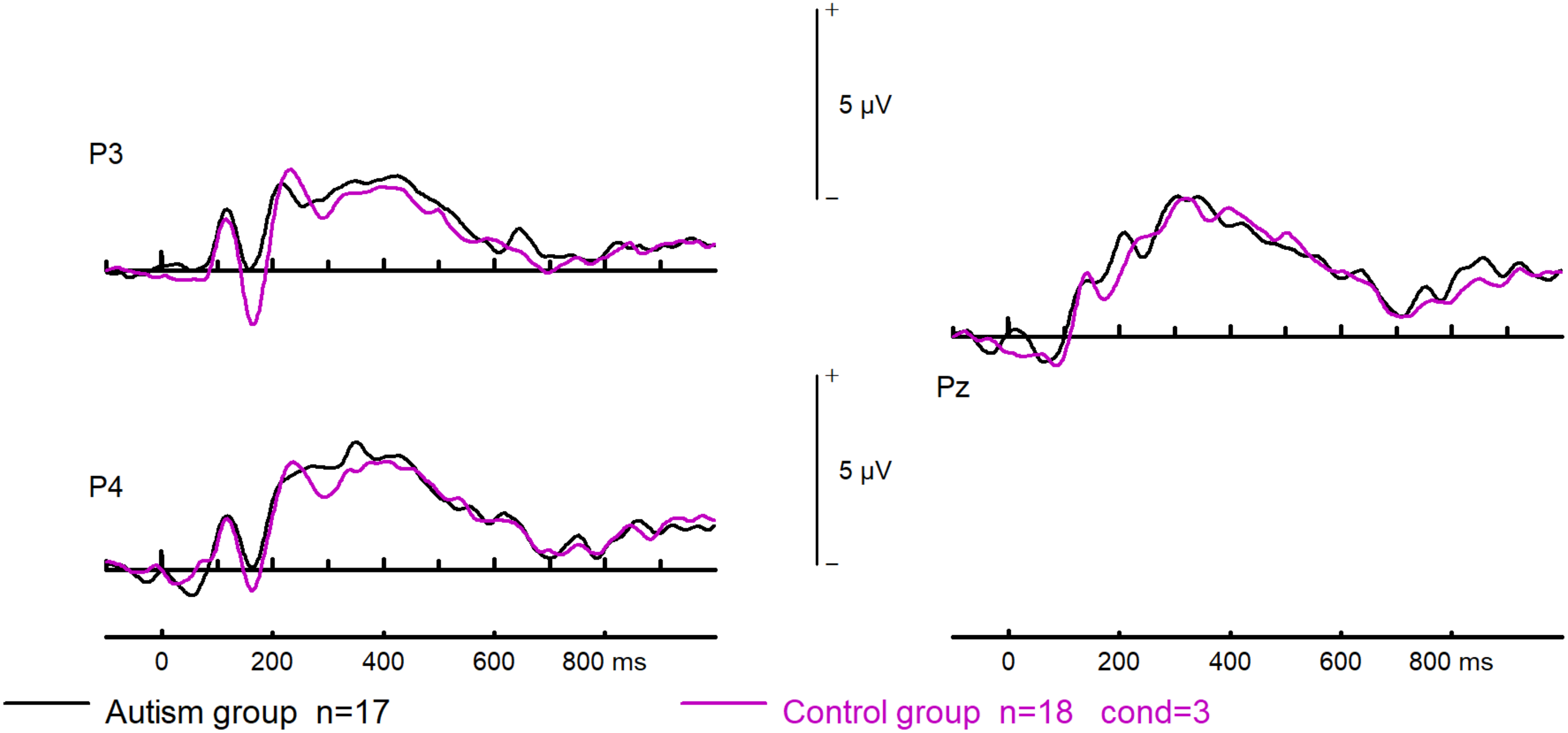

Condition= Letters NotO, NotX

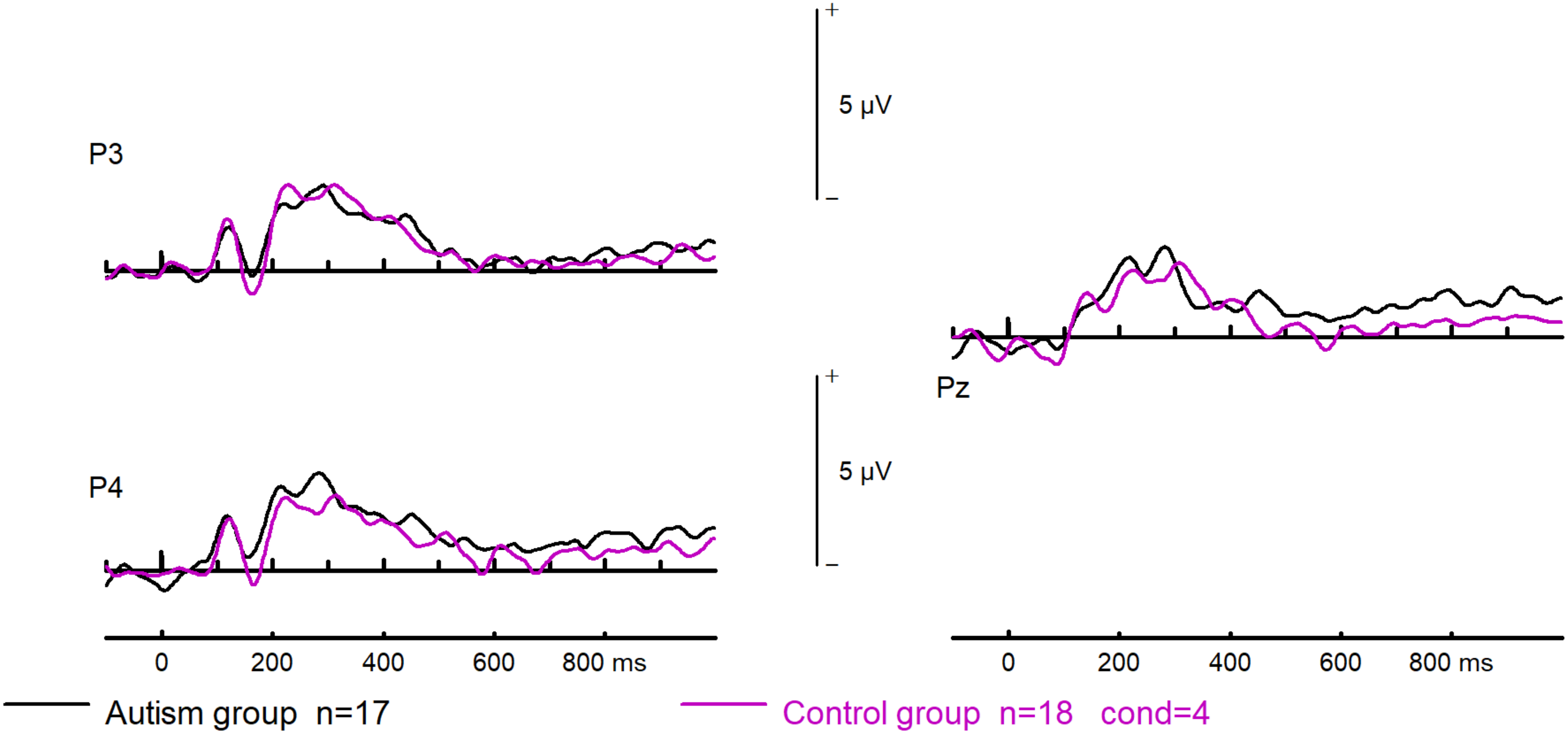

Condition= Faces OX

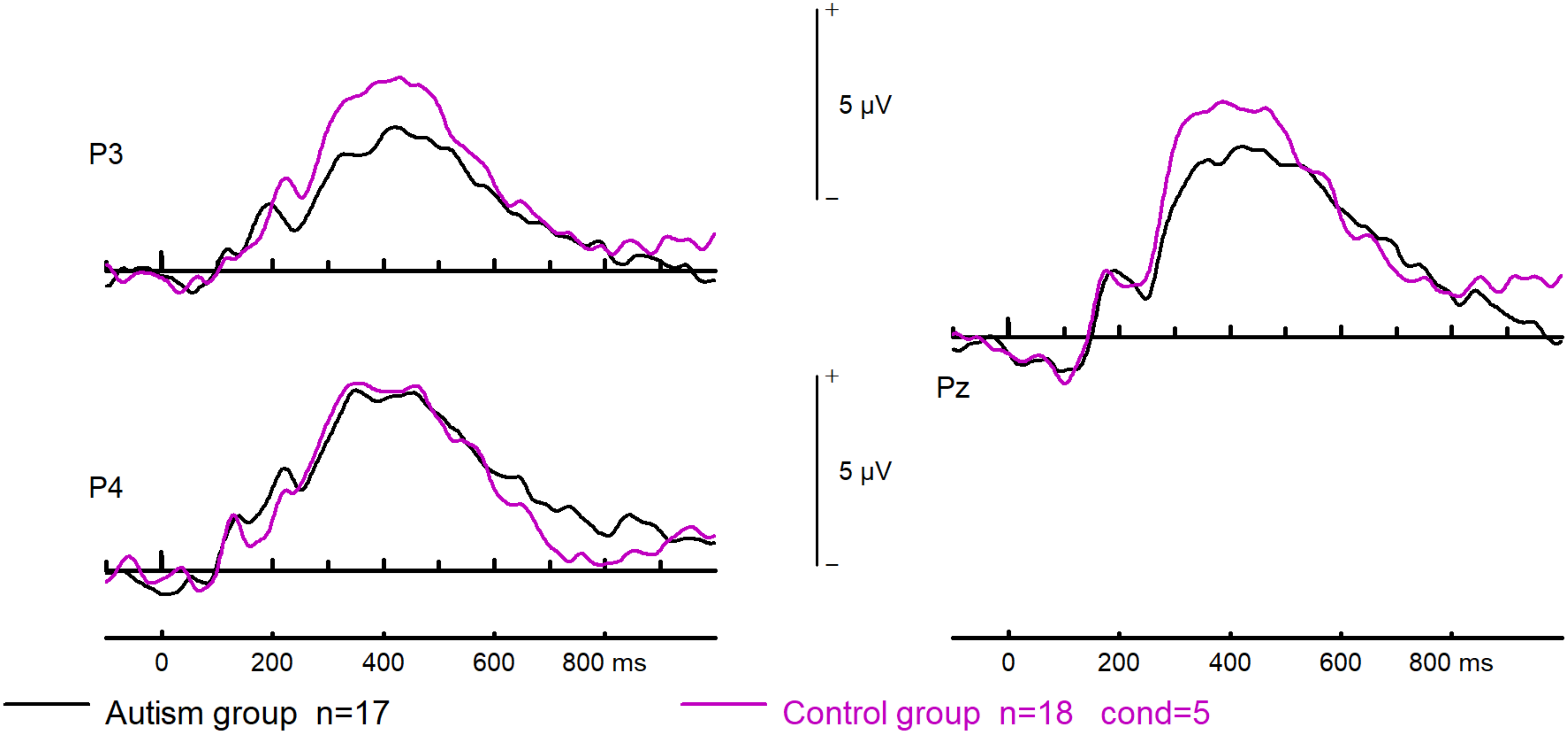

Condition= Faces NotO,X

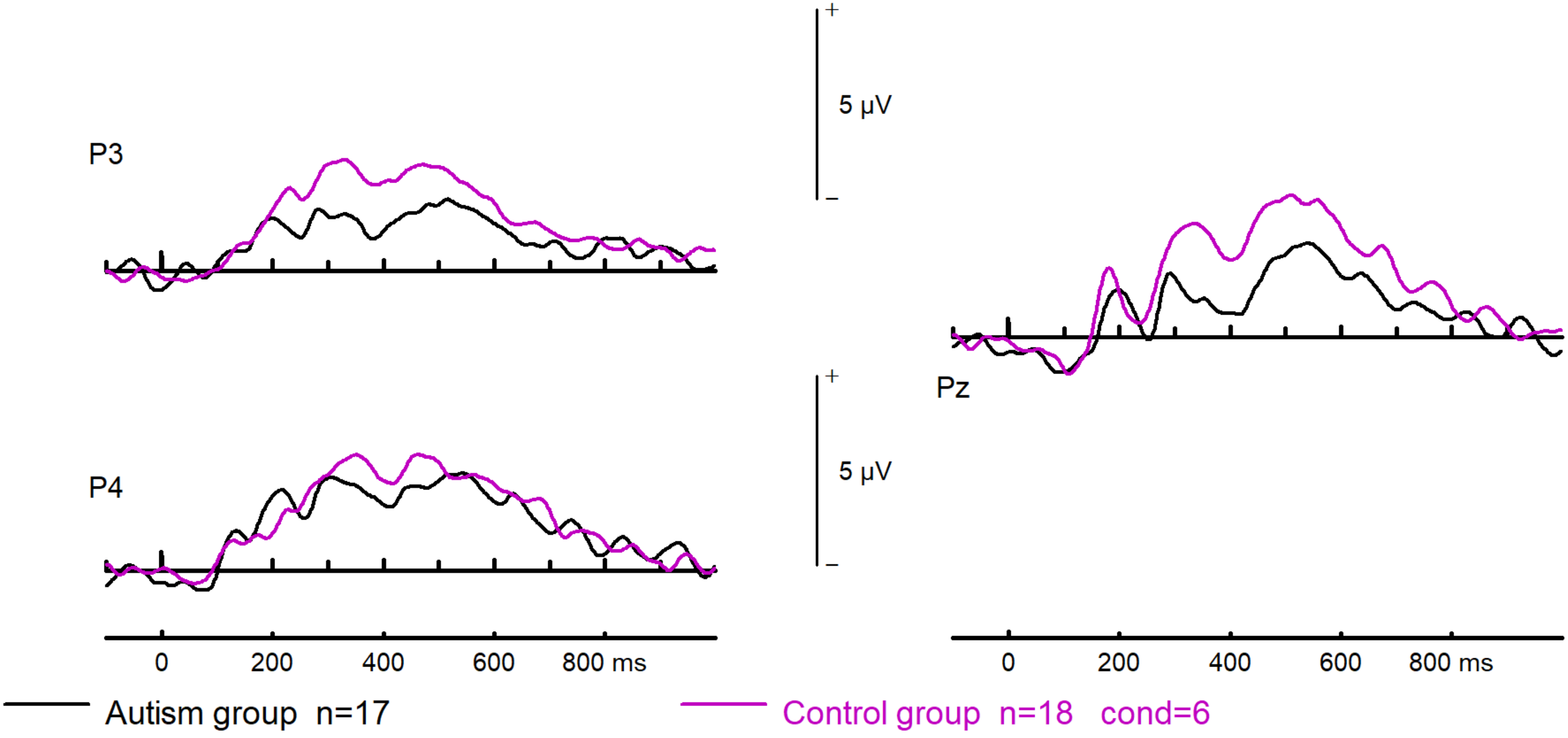

Condition= Faces O, Not X

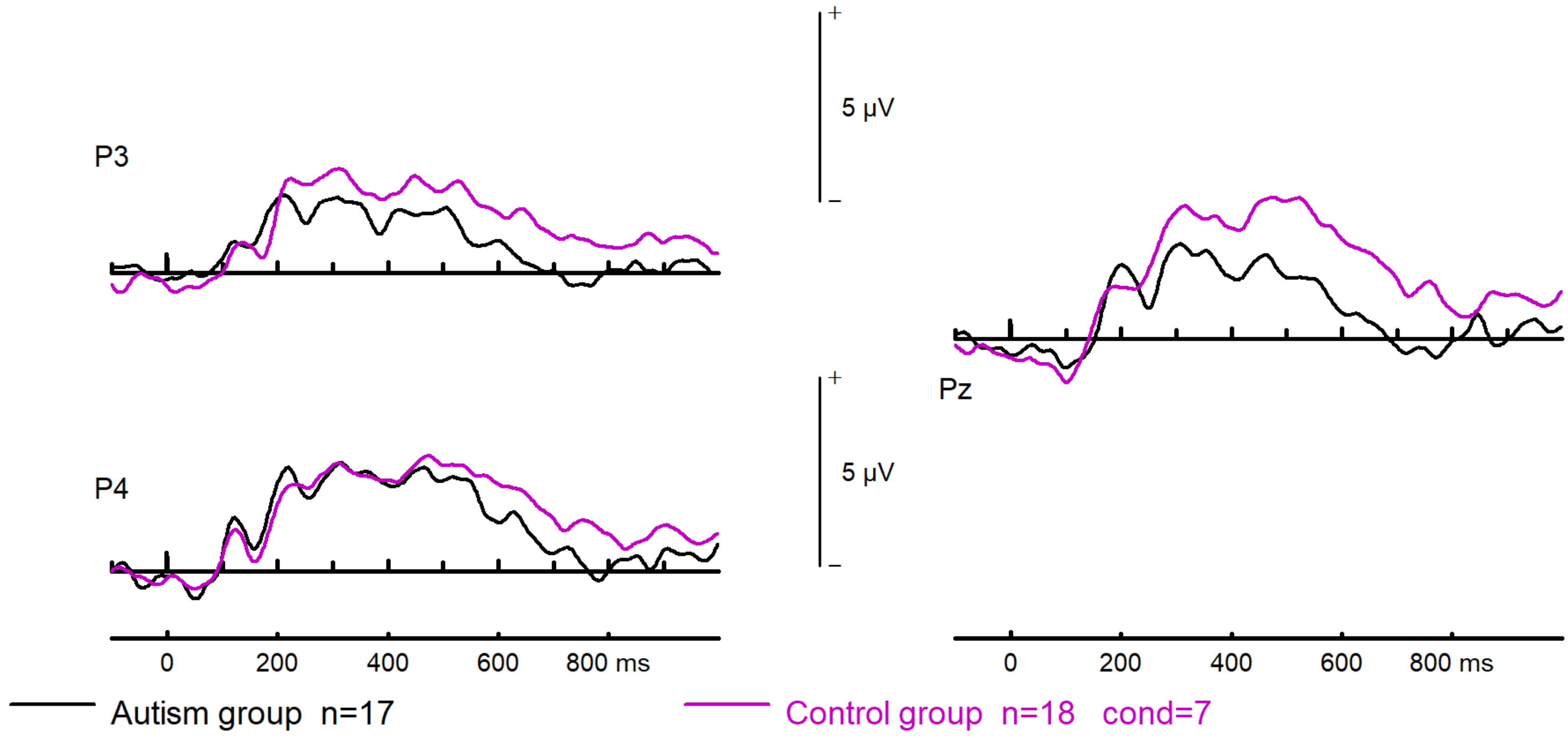

Condition= Faces NotO, NotX

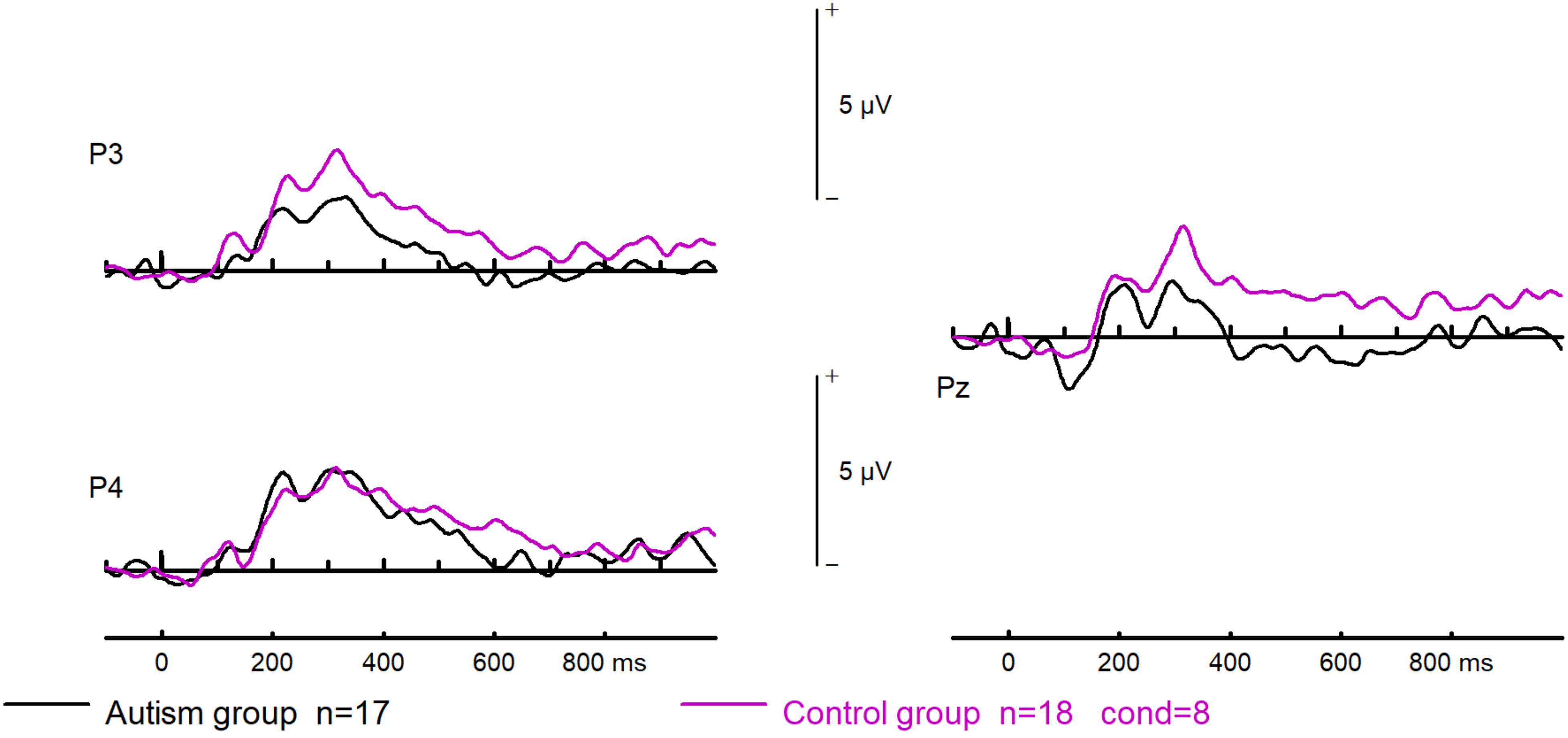

Condition= Letters OX

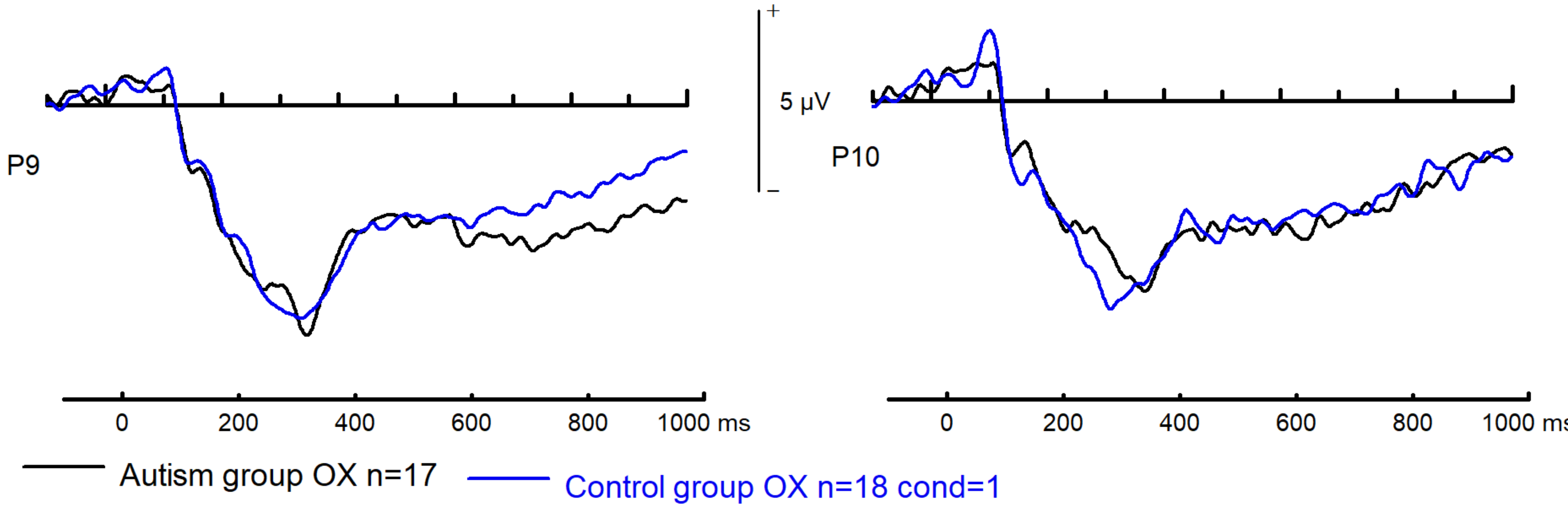

Condition= Letters Not O, X

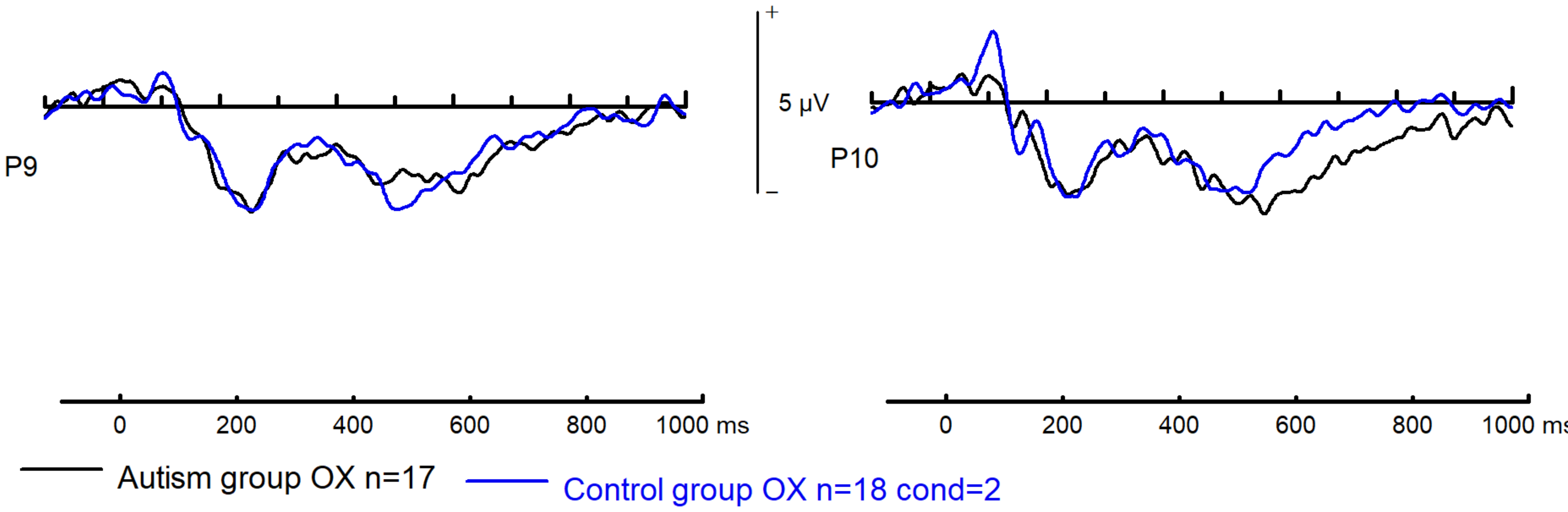

Condition= Letters O, NotX

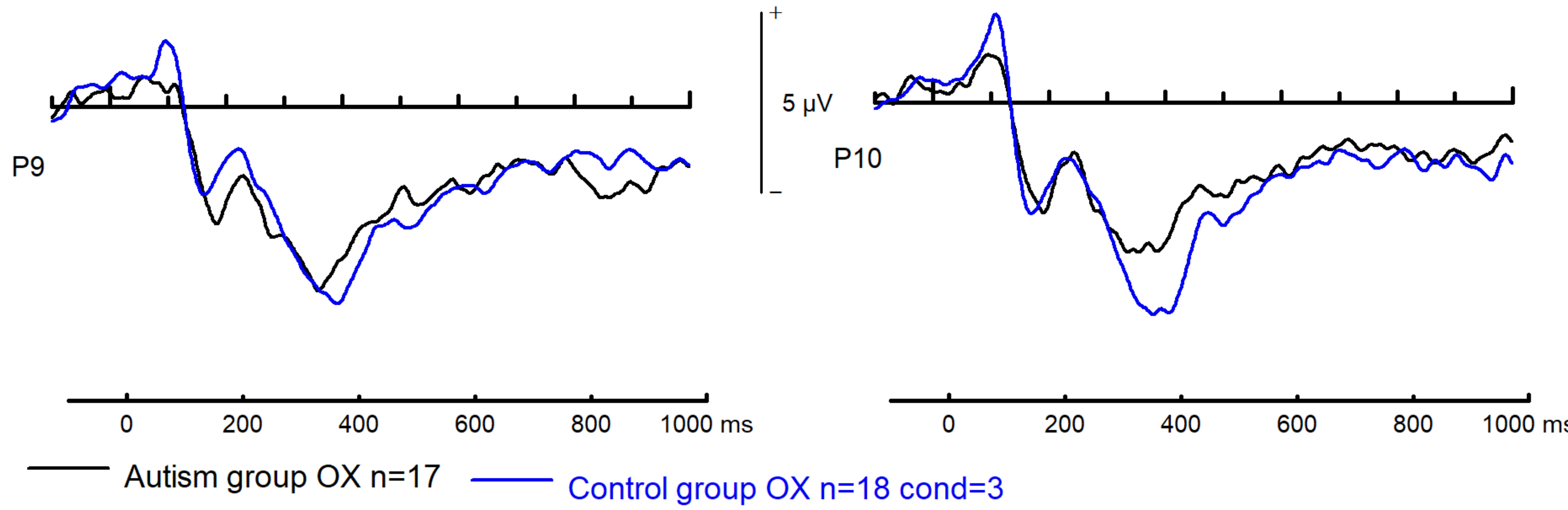

Condition= Letters NotO, NotX

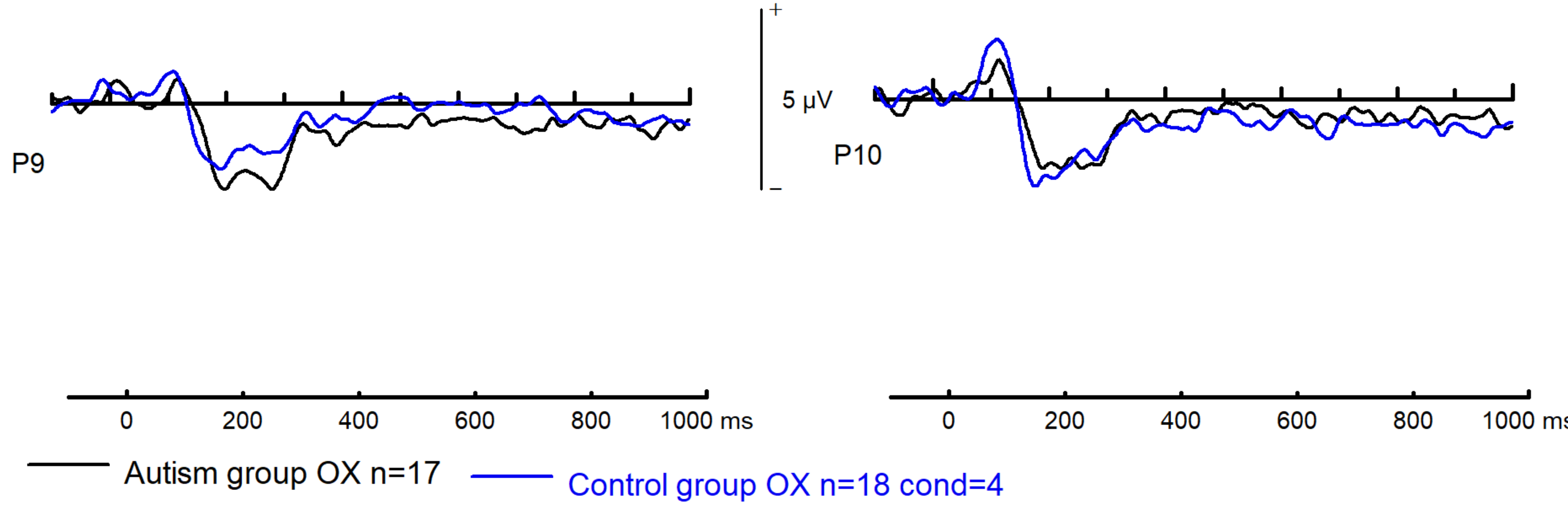

Condition= Faces OX

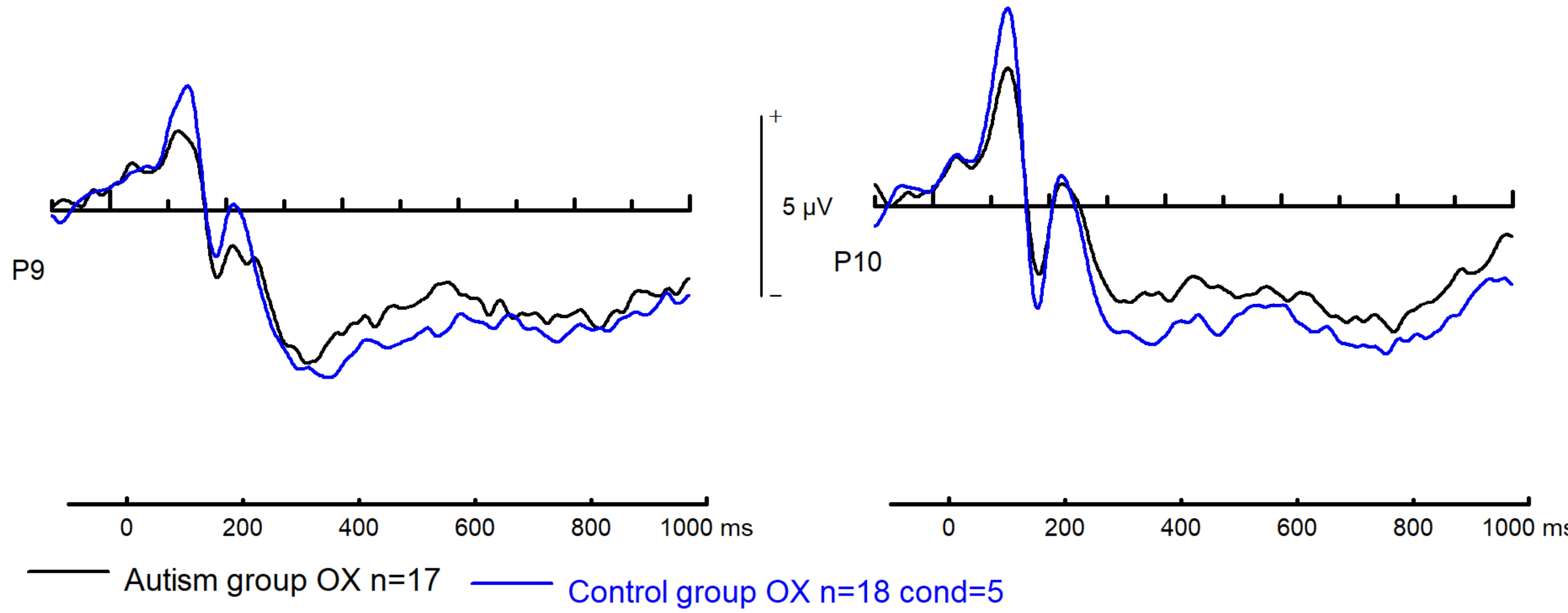

Condition= Faces NotO,X

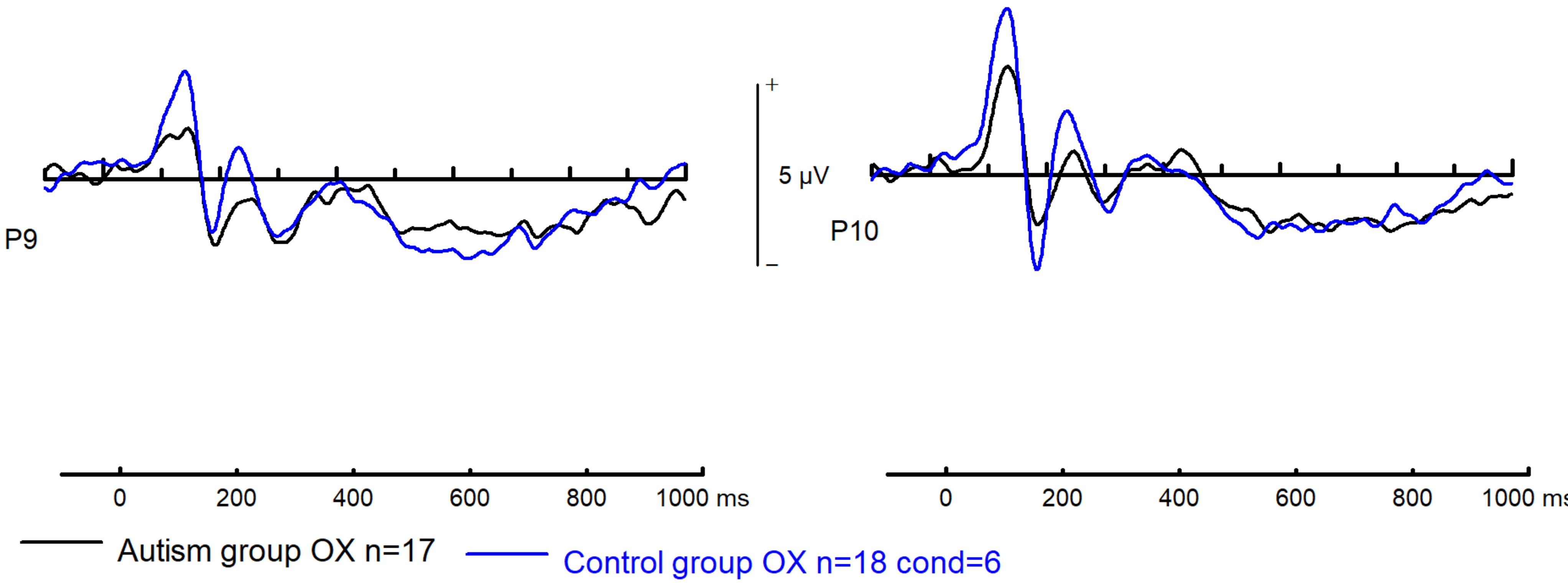

Condition= Faces O, Not X

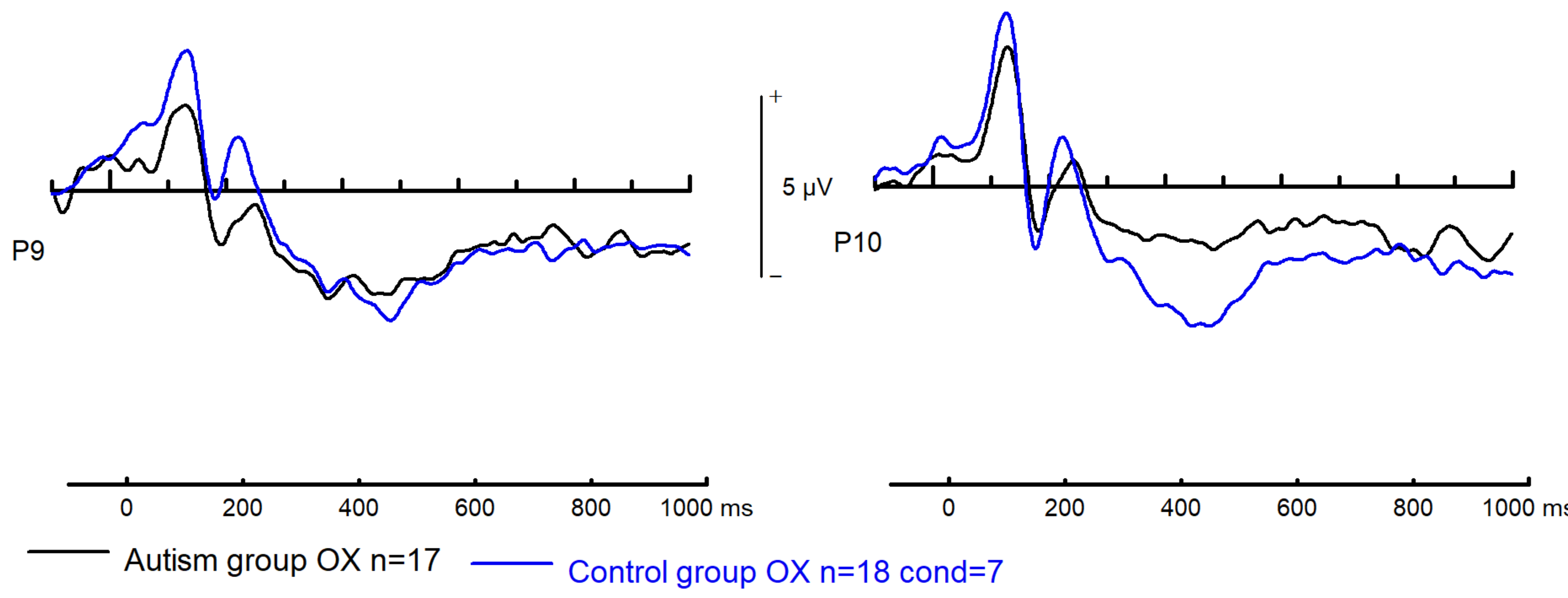

Condition= Faces NotO, NotX

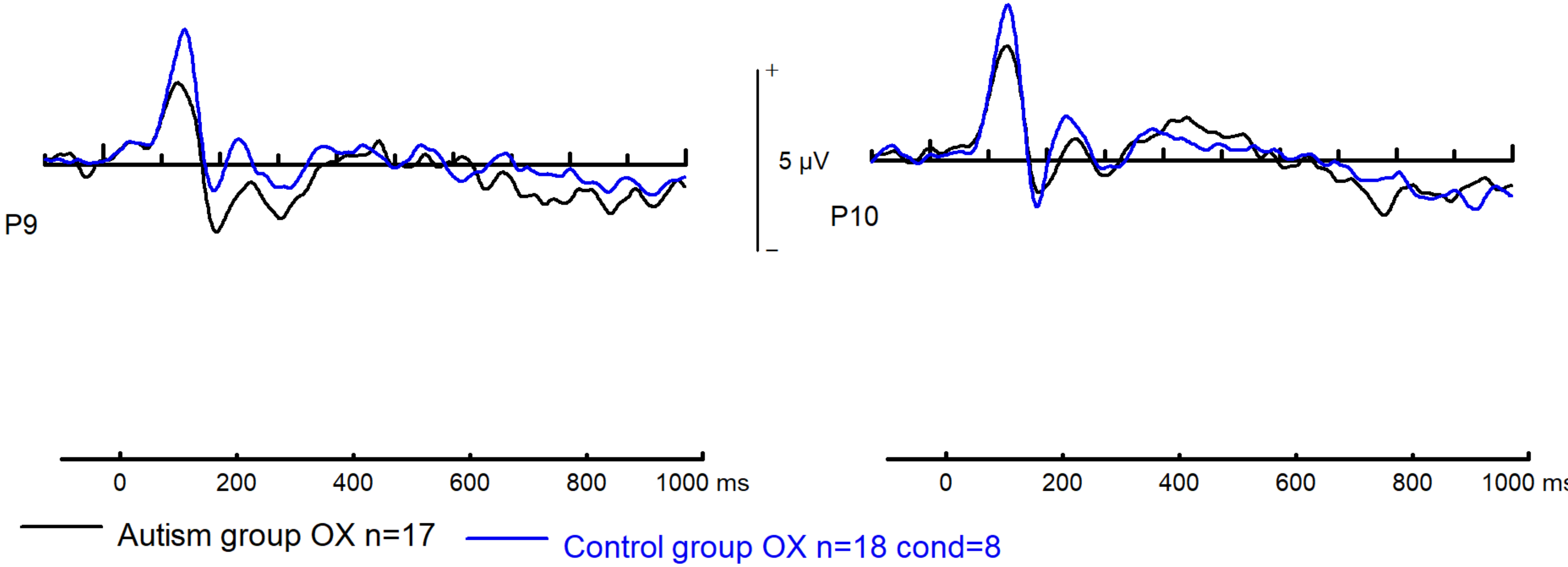

Condition= Letters OX

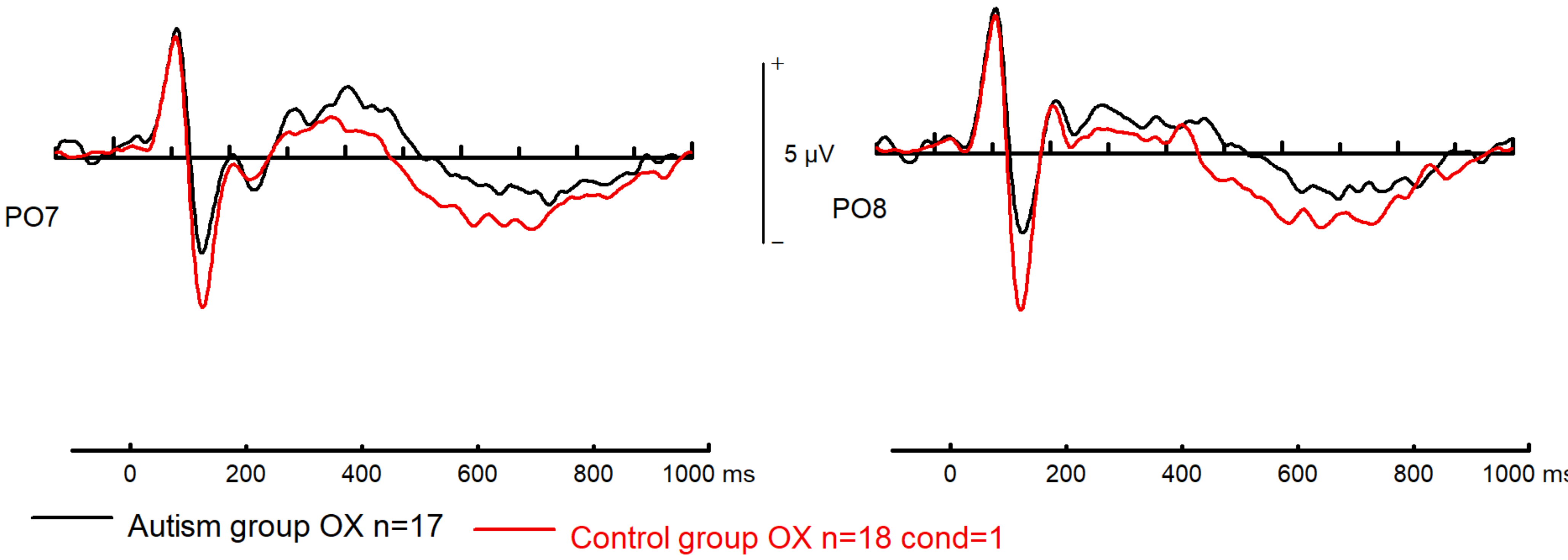

Condition= Letters Not O, X

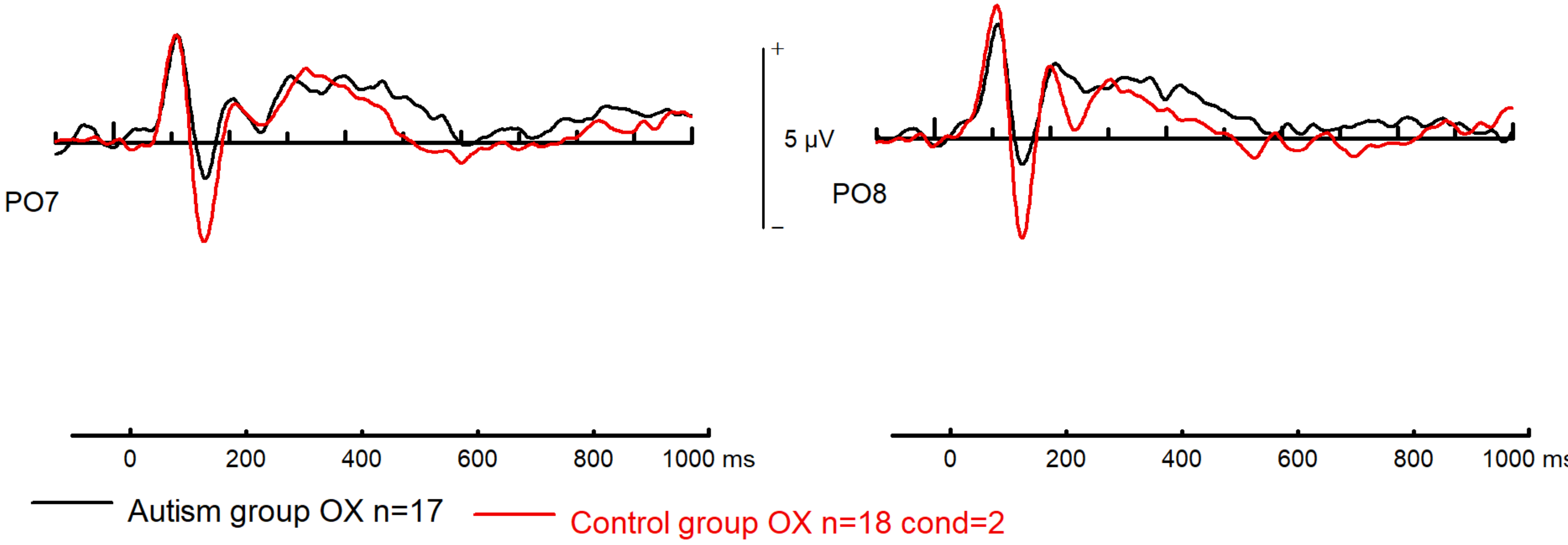

Condition= Letters O, NotX

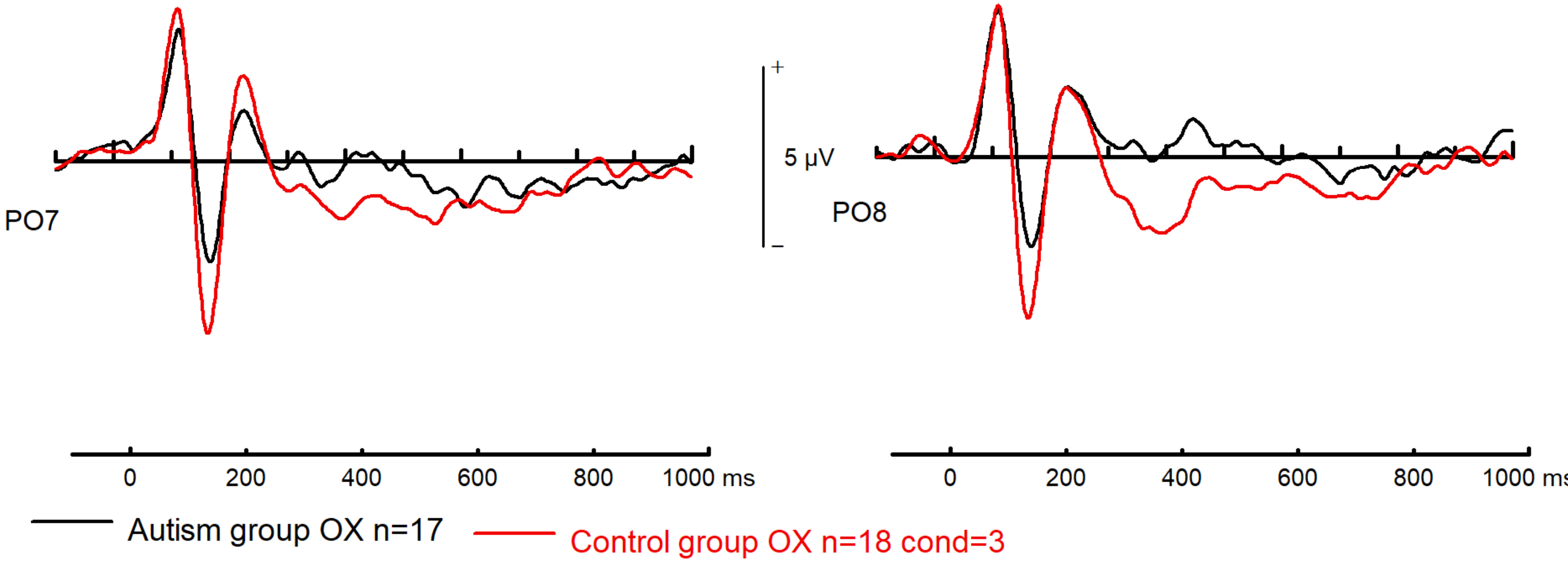

Condition= Letters NotO, NotX

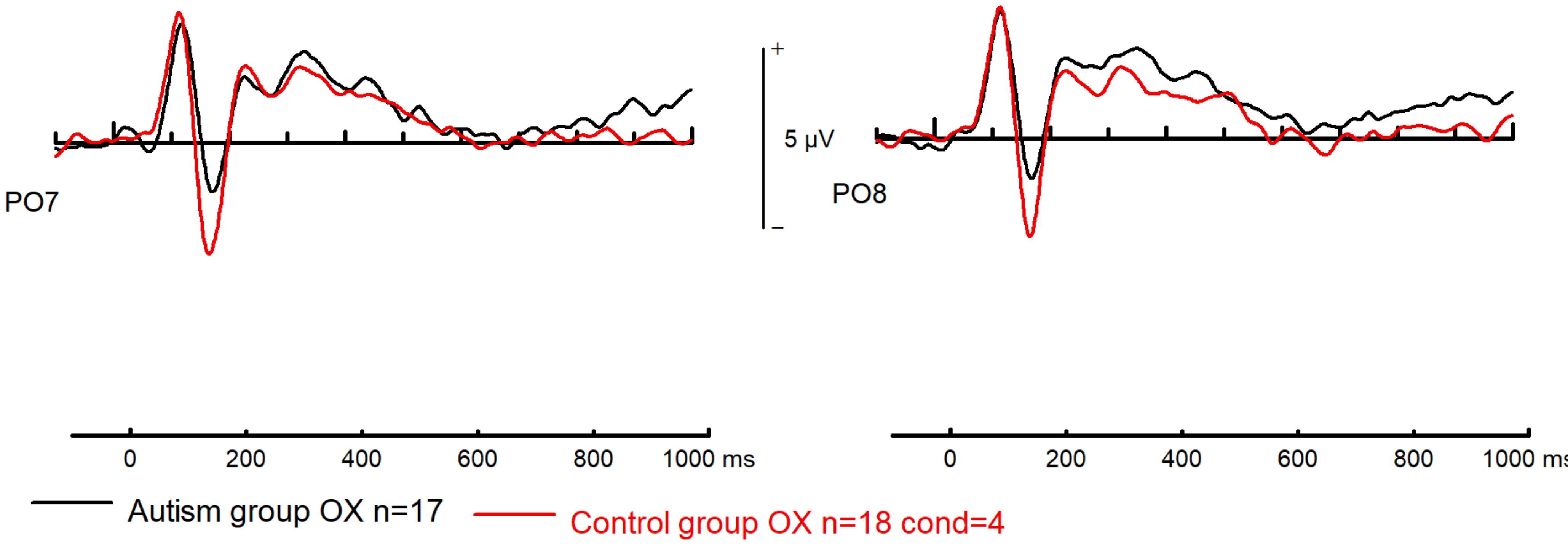

Condition= Faces OX

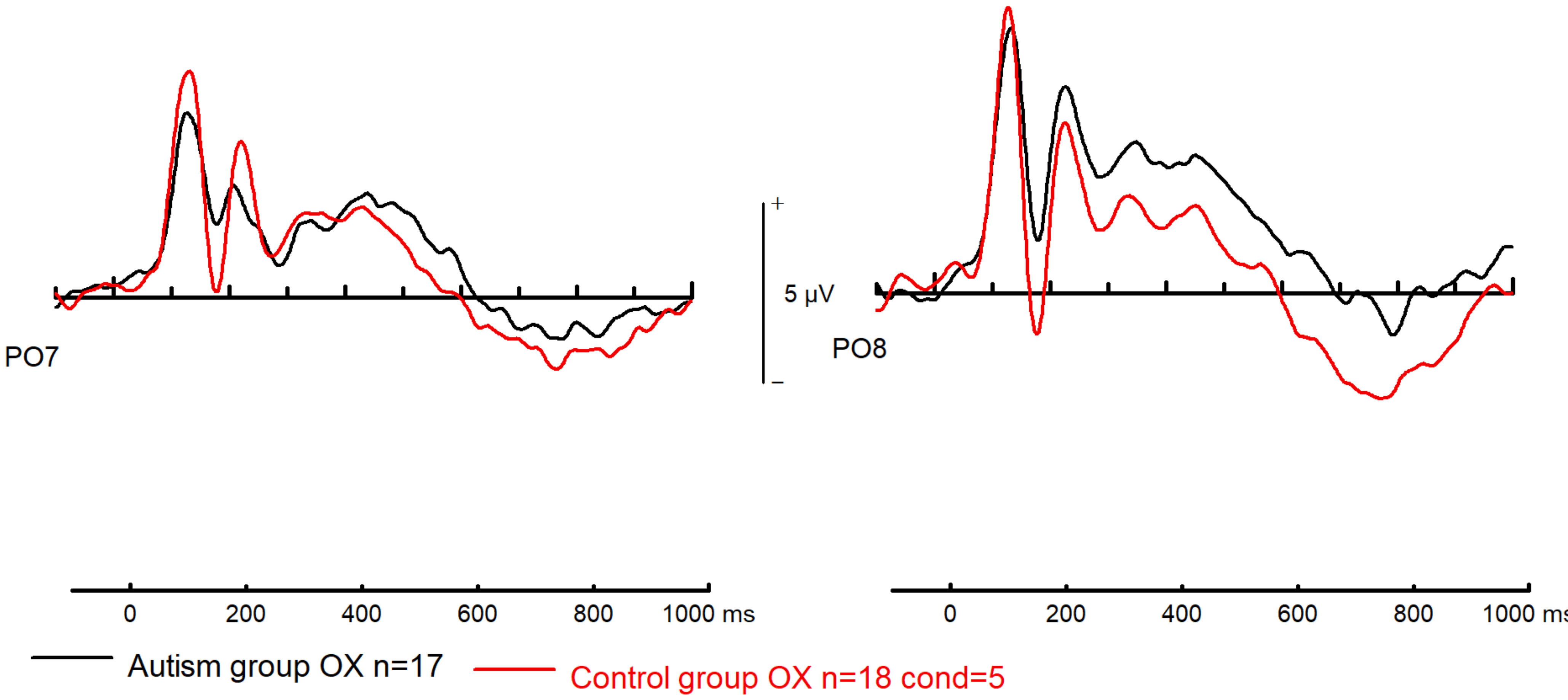

Condition= Faces NotO,X

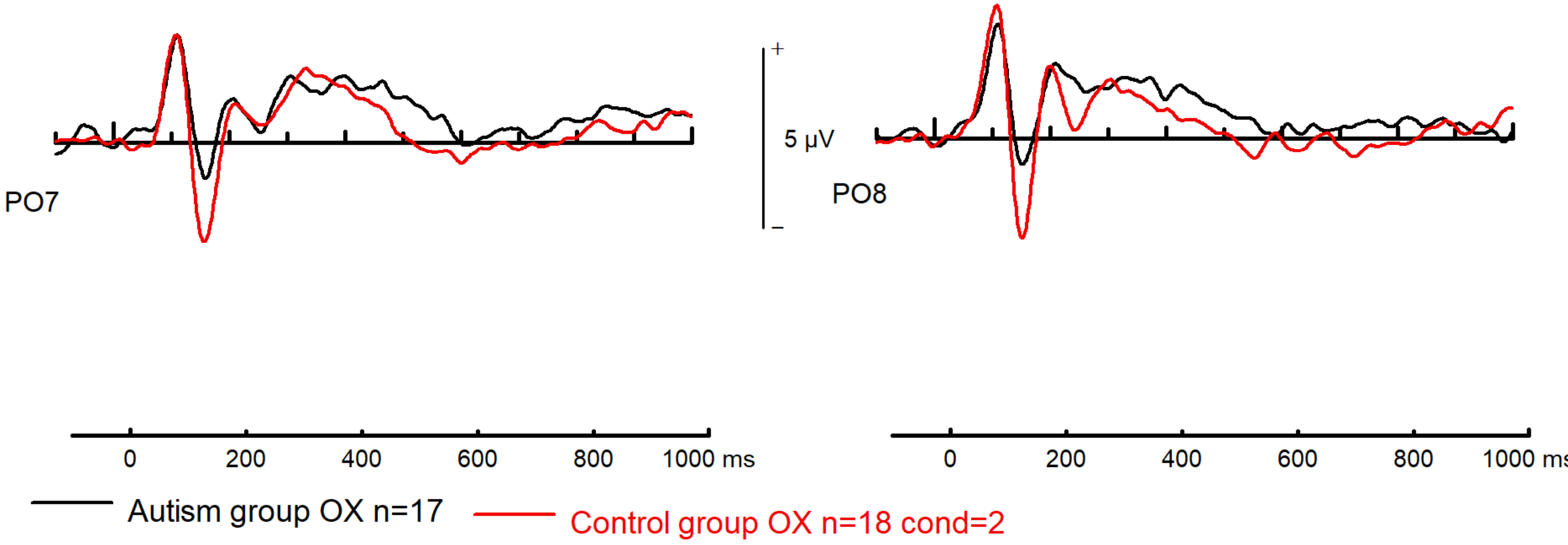

Condition= Faces O, Not X

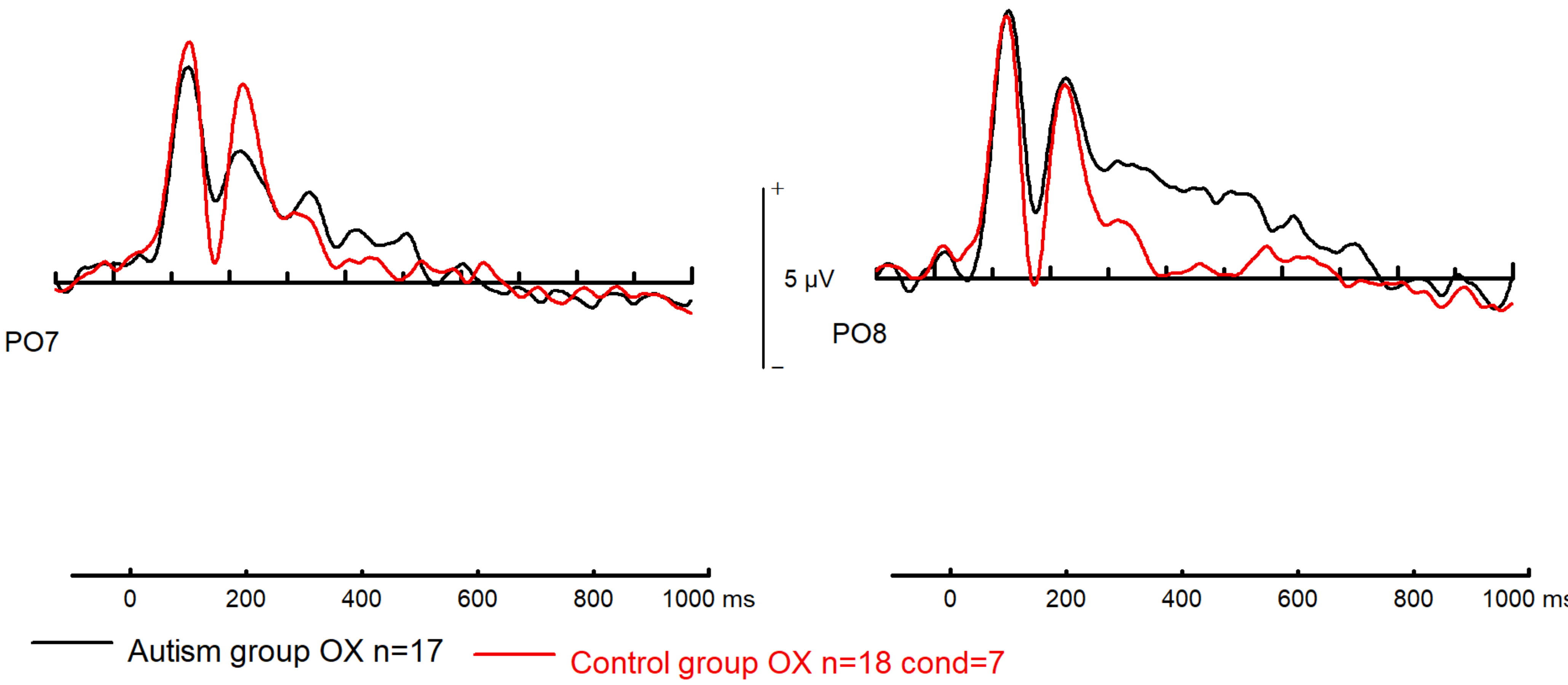

Condition= Faces NotO, NotX

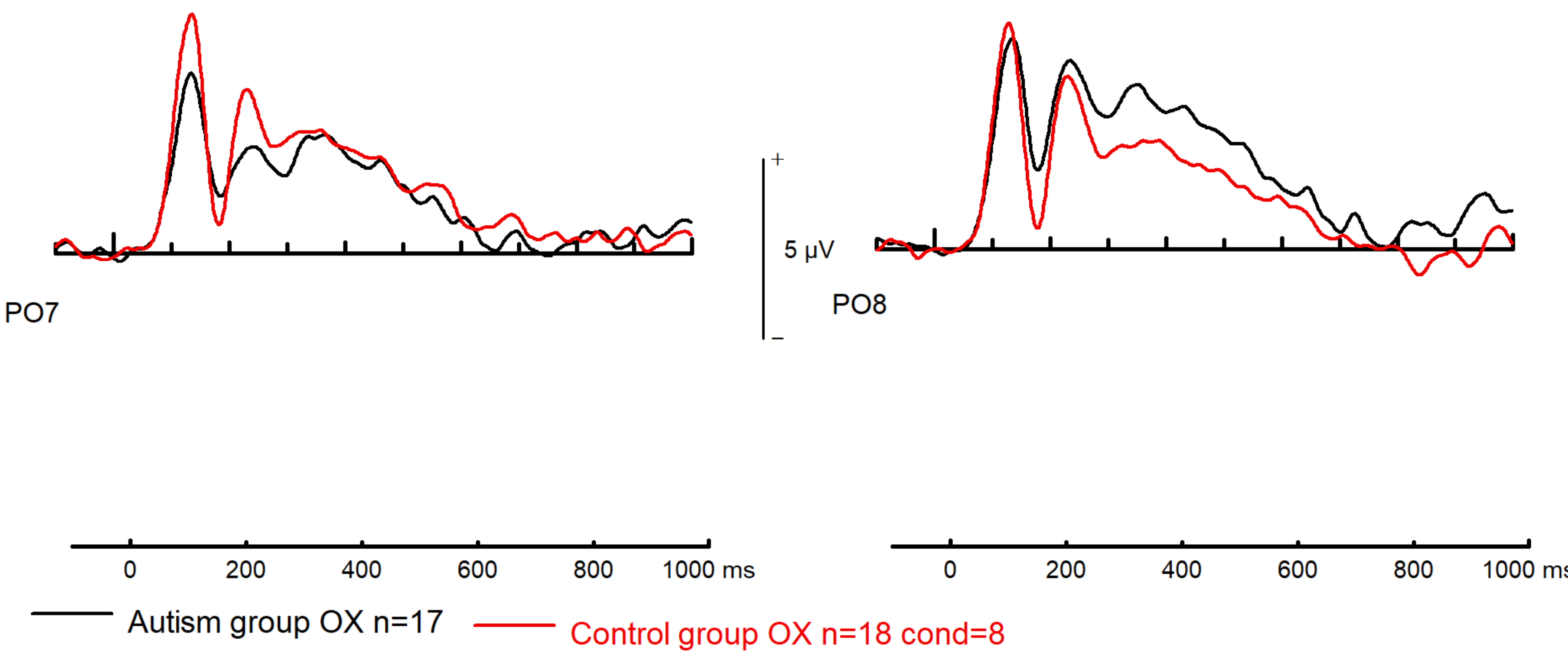

Condition= Letters OX

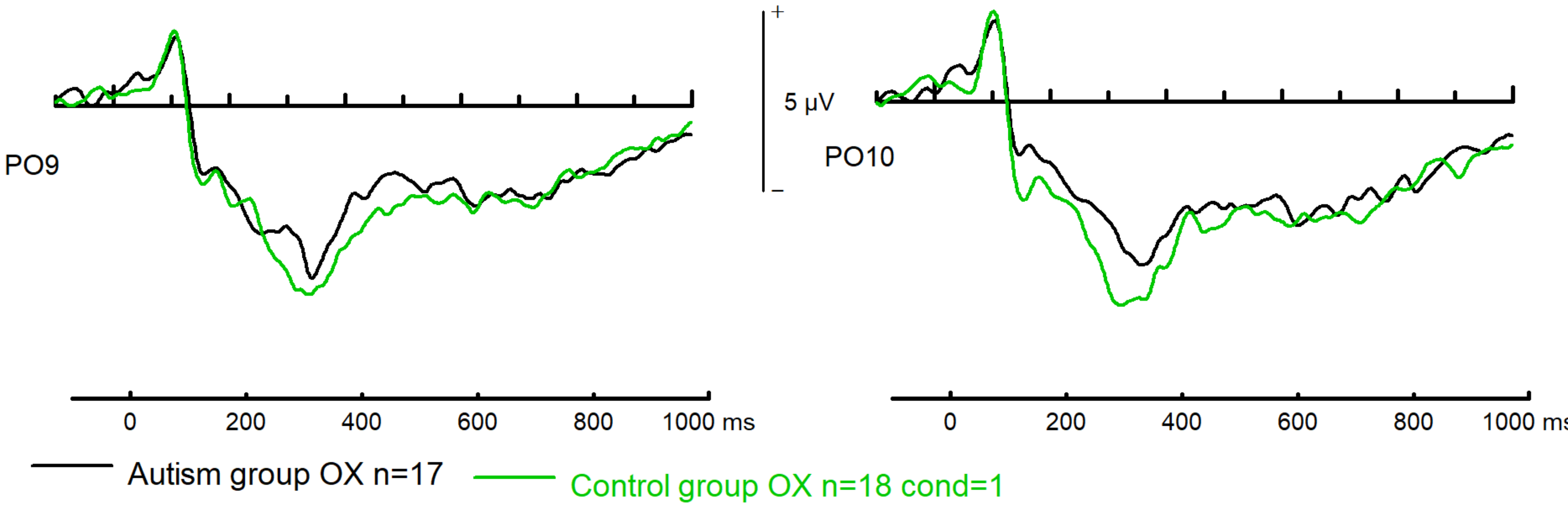

Condition= Letters Not O, X

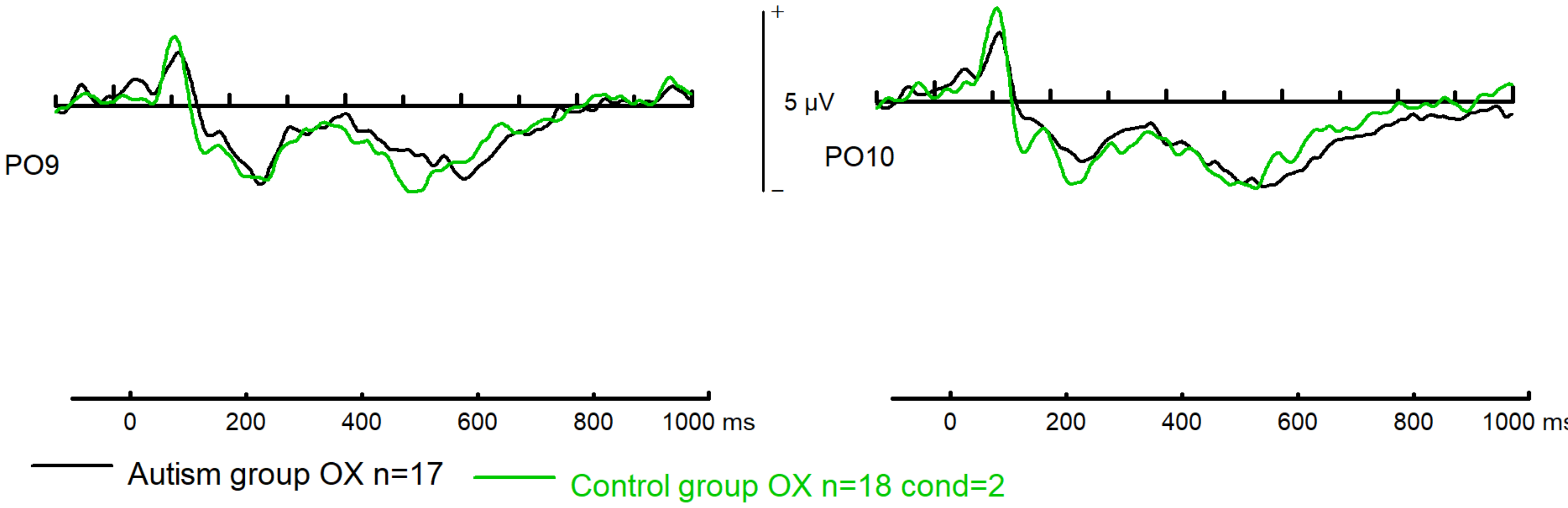

Condition= Letters O, NotX

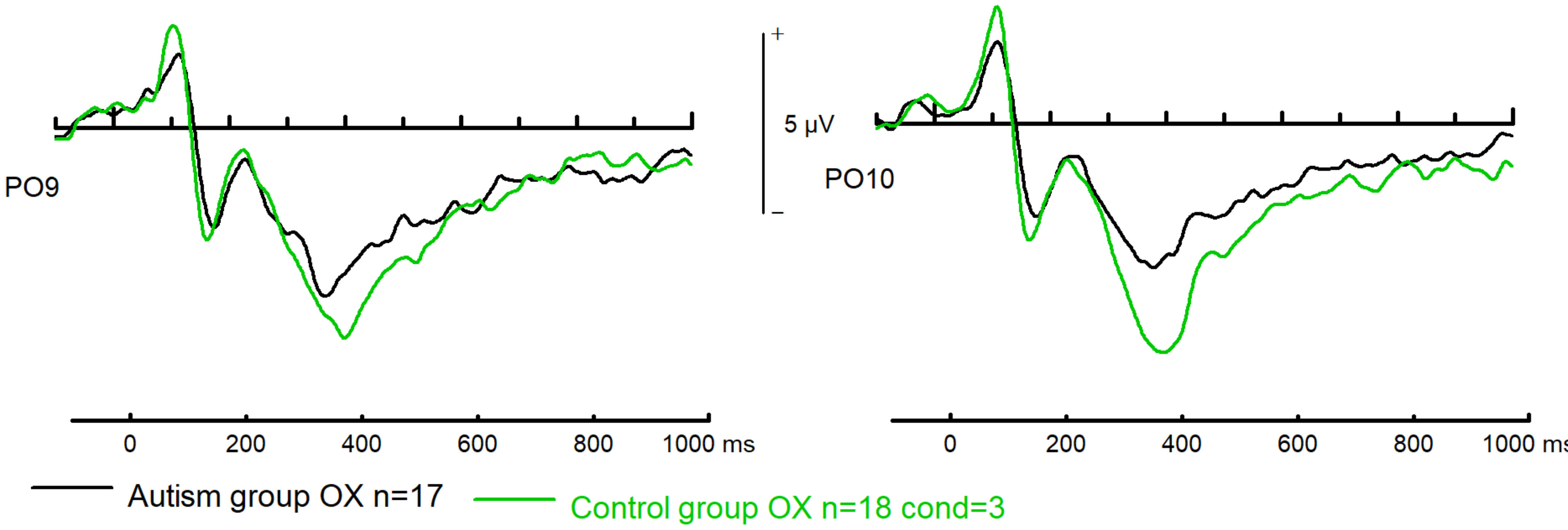

Condition= Letters NotO, NotX

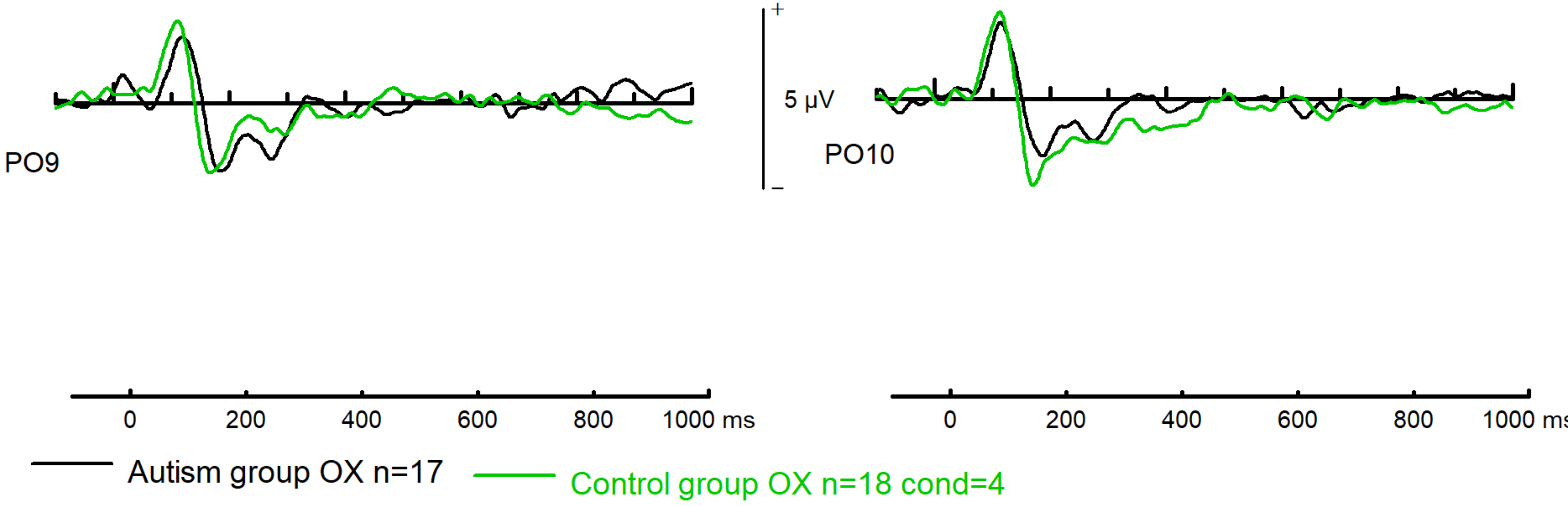

Condition= Faces OX

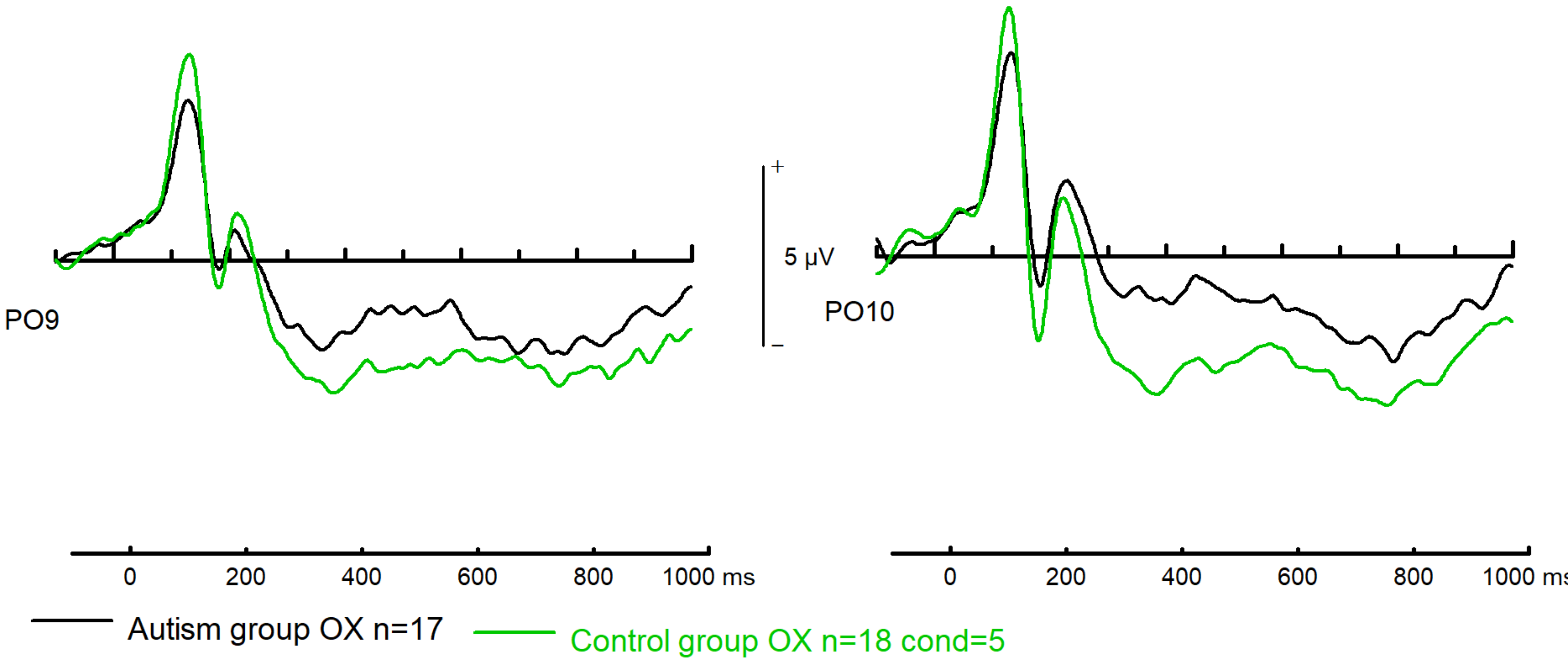

Condition= Faces NotO,X

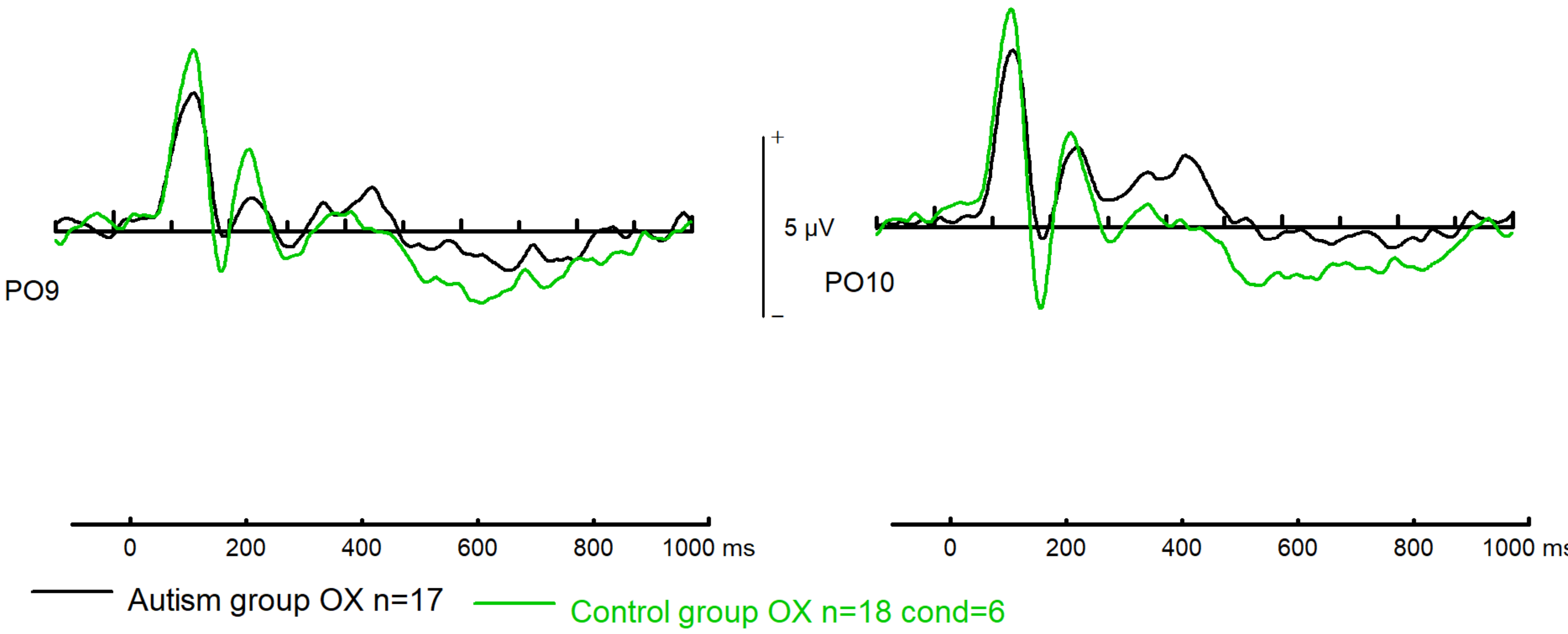

Condition= Faces O, Not X

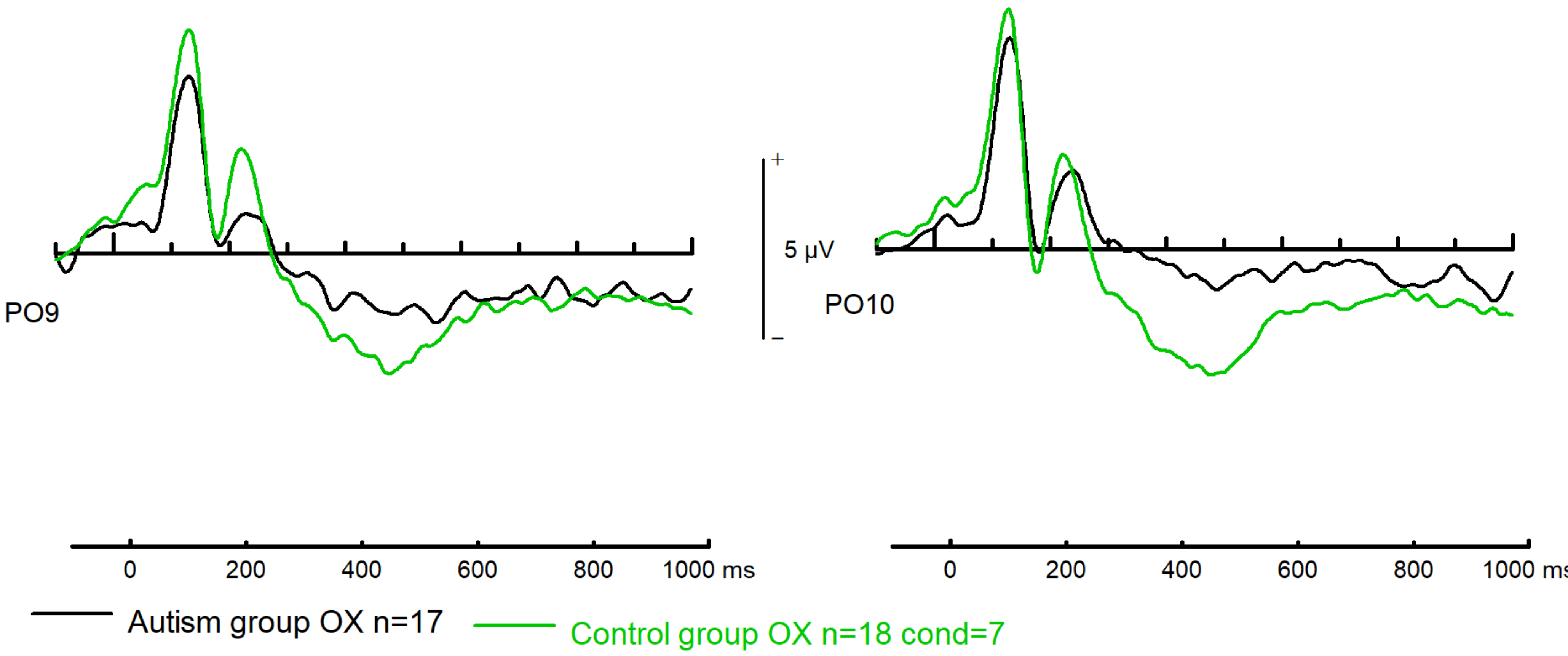

Condition= Faces NotO, NotX

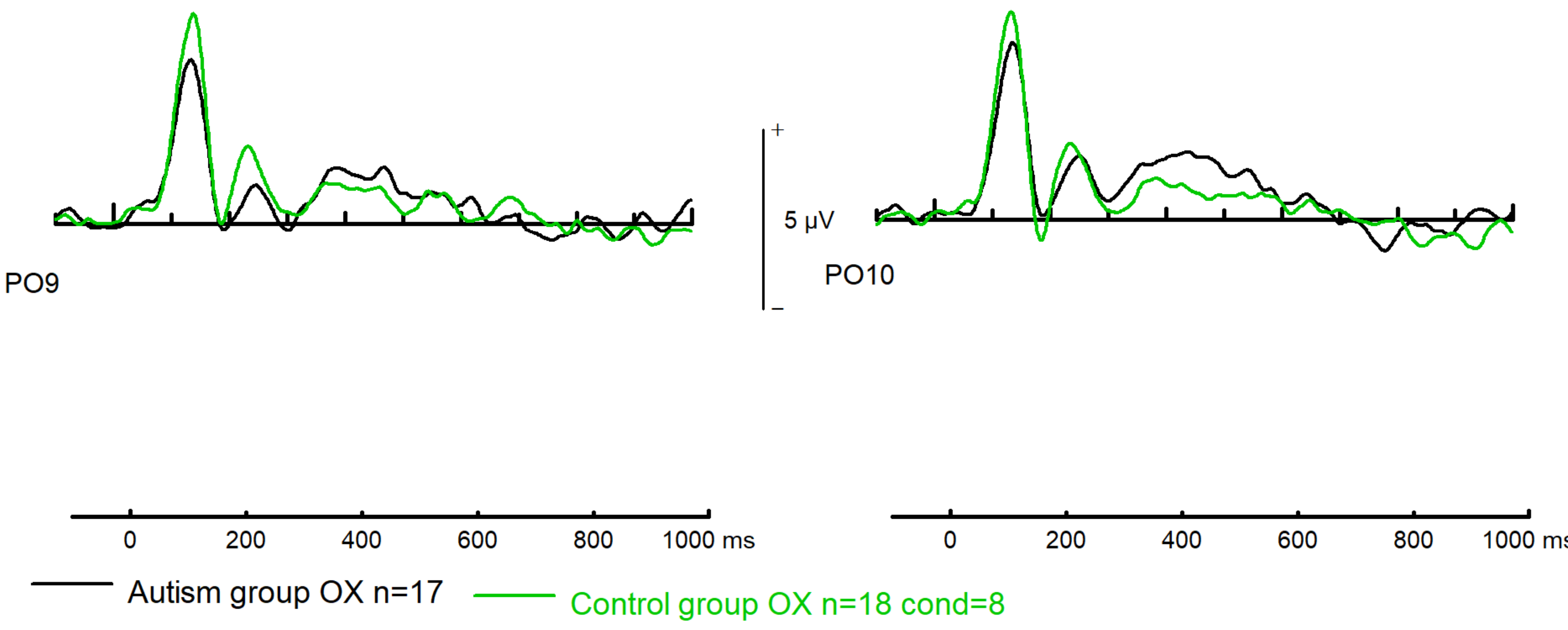

Condition= Letters OX

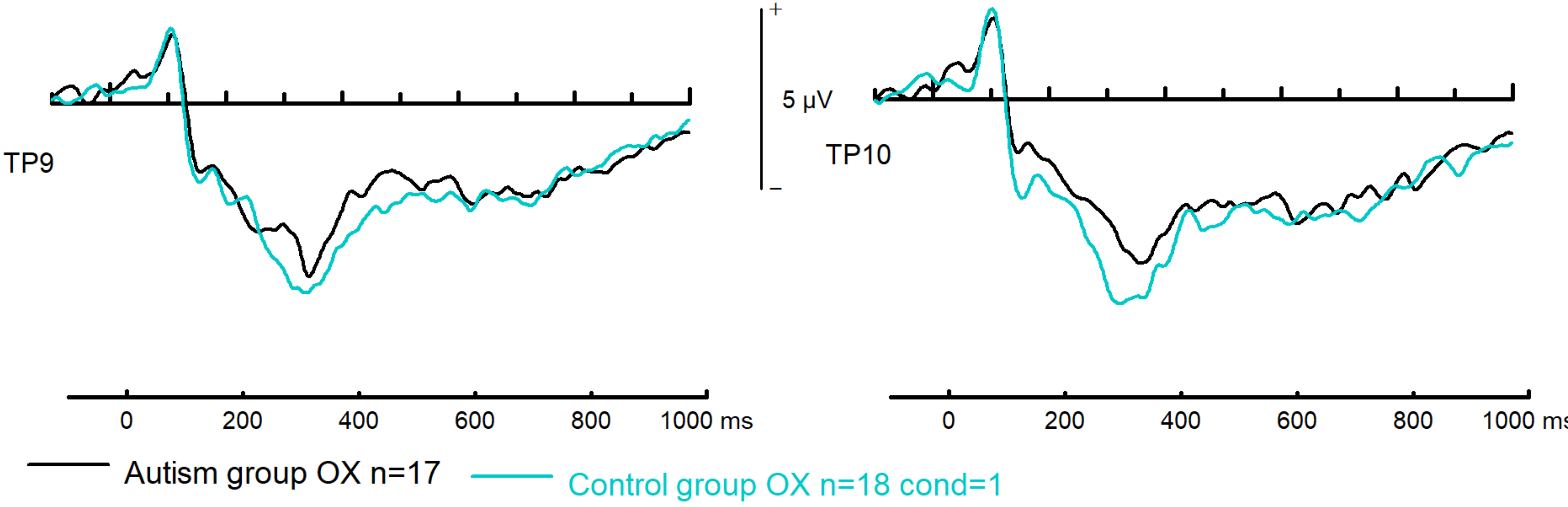

Condition= Letters Not O, X

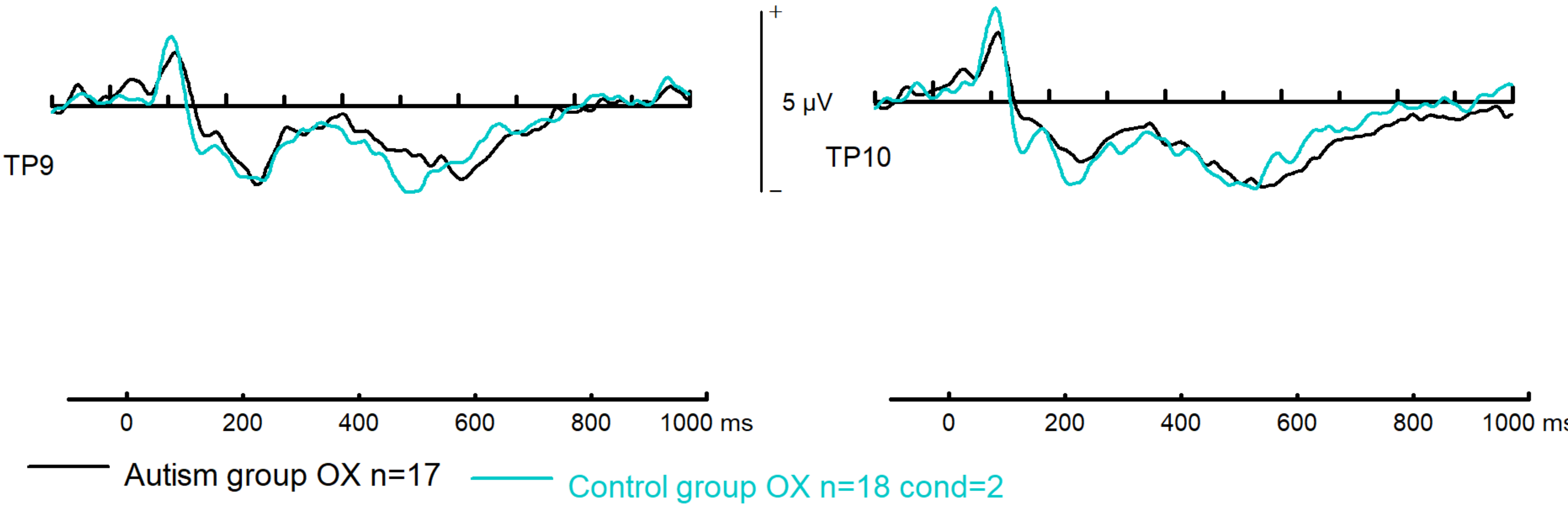

Condition= Letters O, NotX

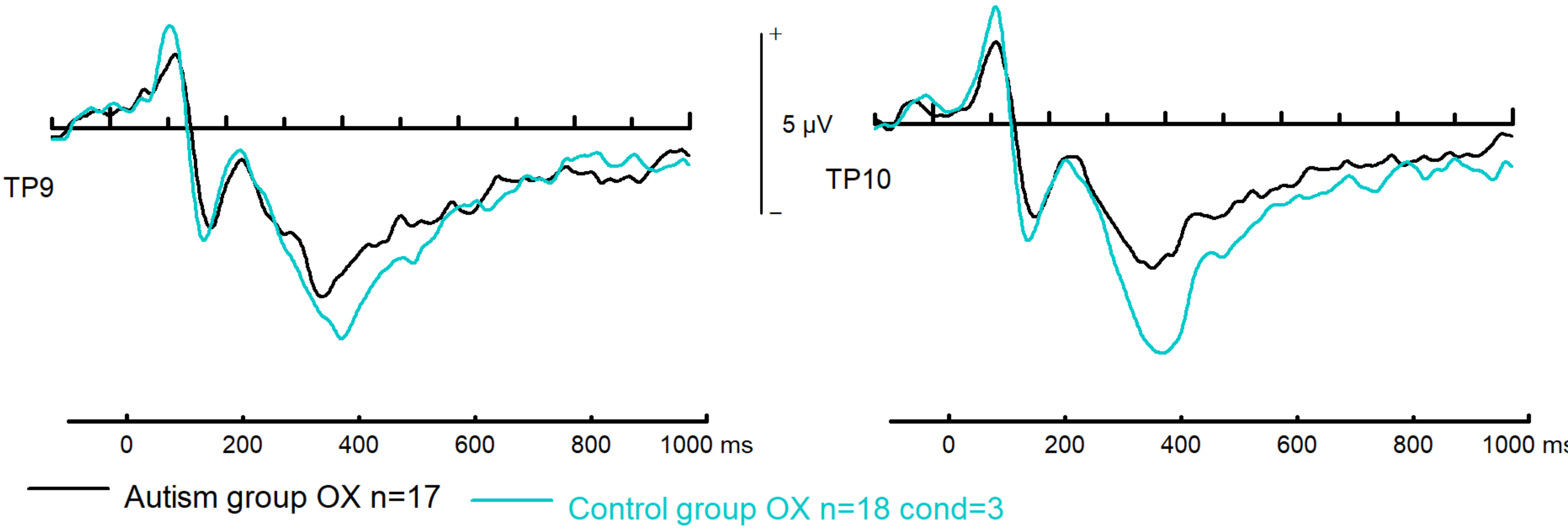

Condition= Letters NotO, NotX

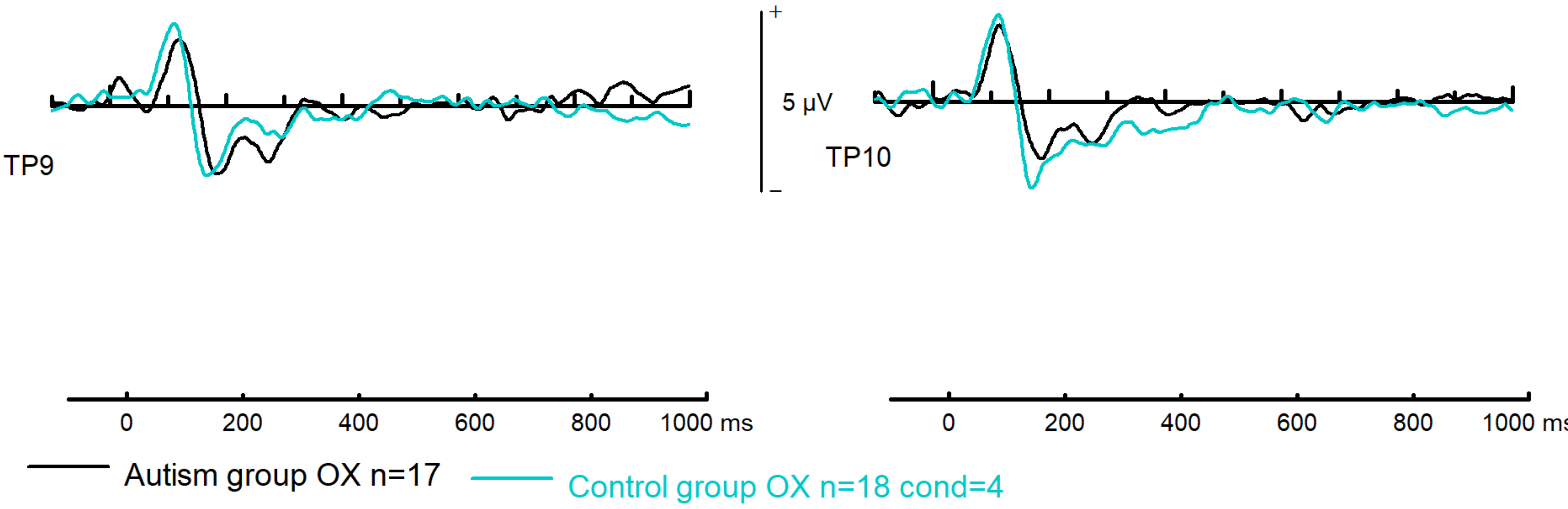

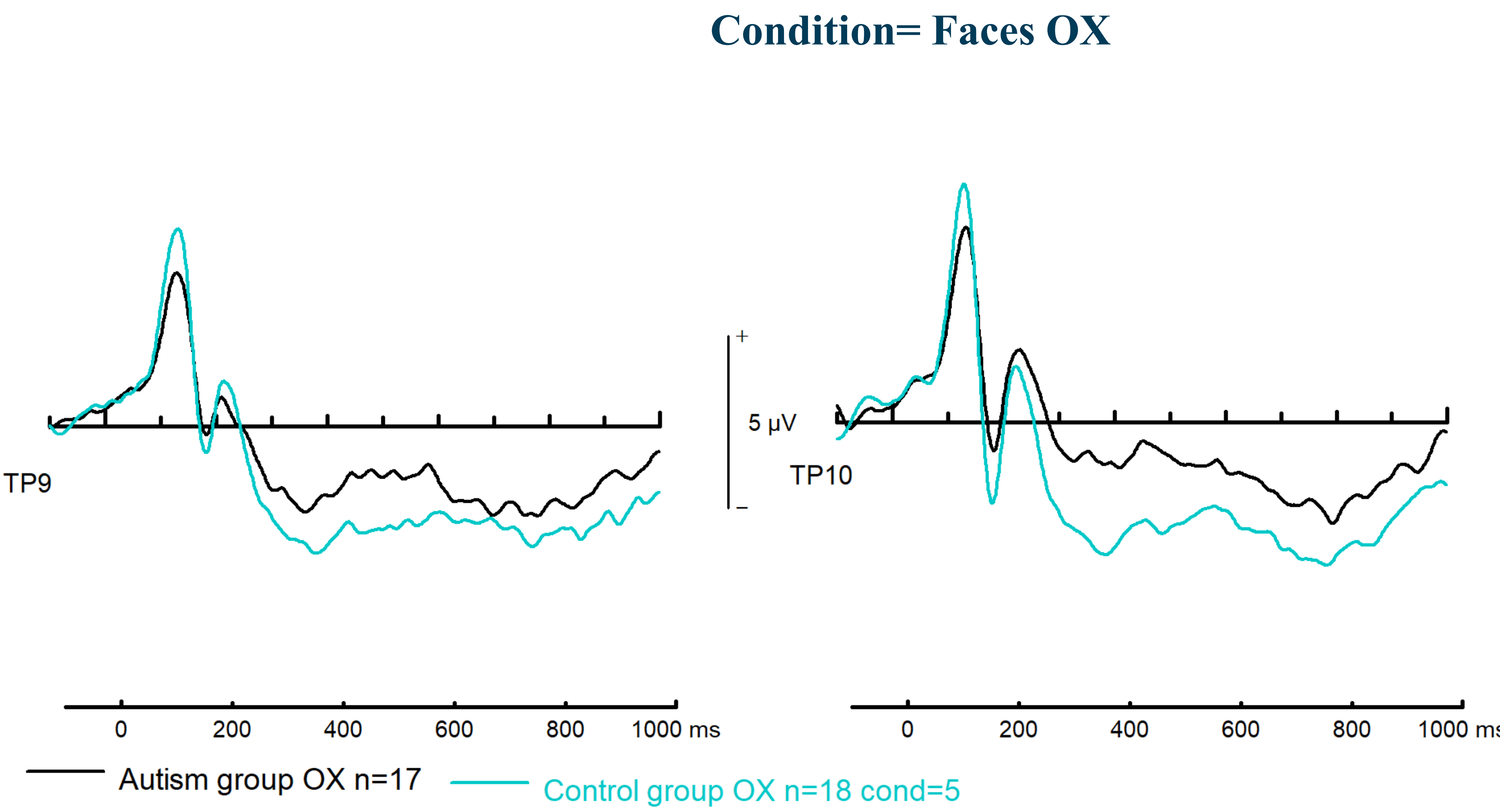

Condition= Faces OX
TP9
5 µV
+
–
TP10
0
200
400
600
800
1000 ms
Autism group OX n=17
Control group OX n=18 cond=5

Condition= Faces NotO,X

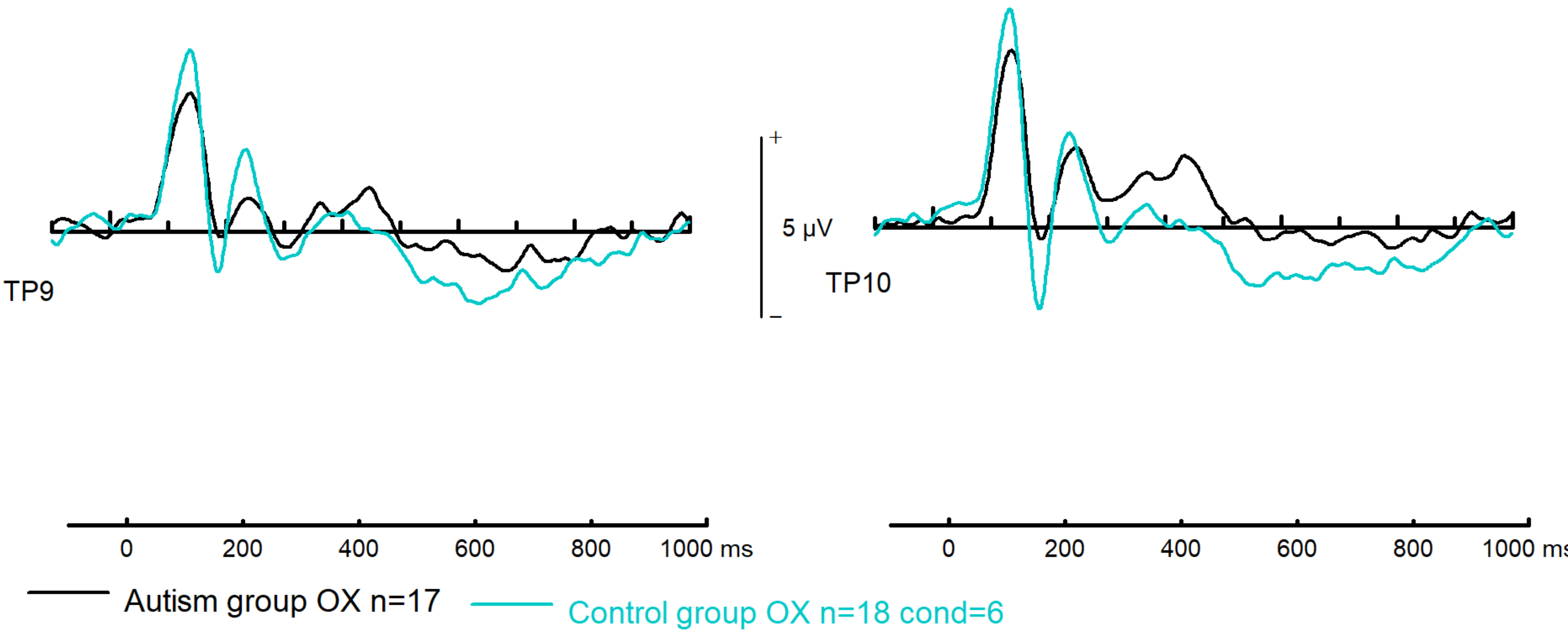

Condition= Faces O, Not X

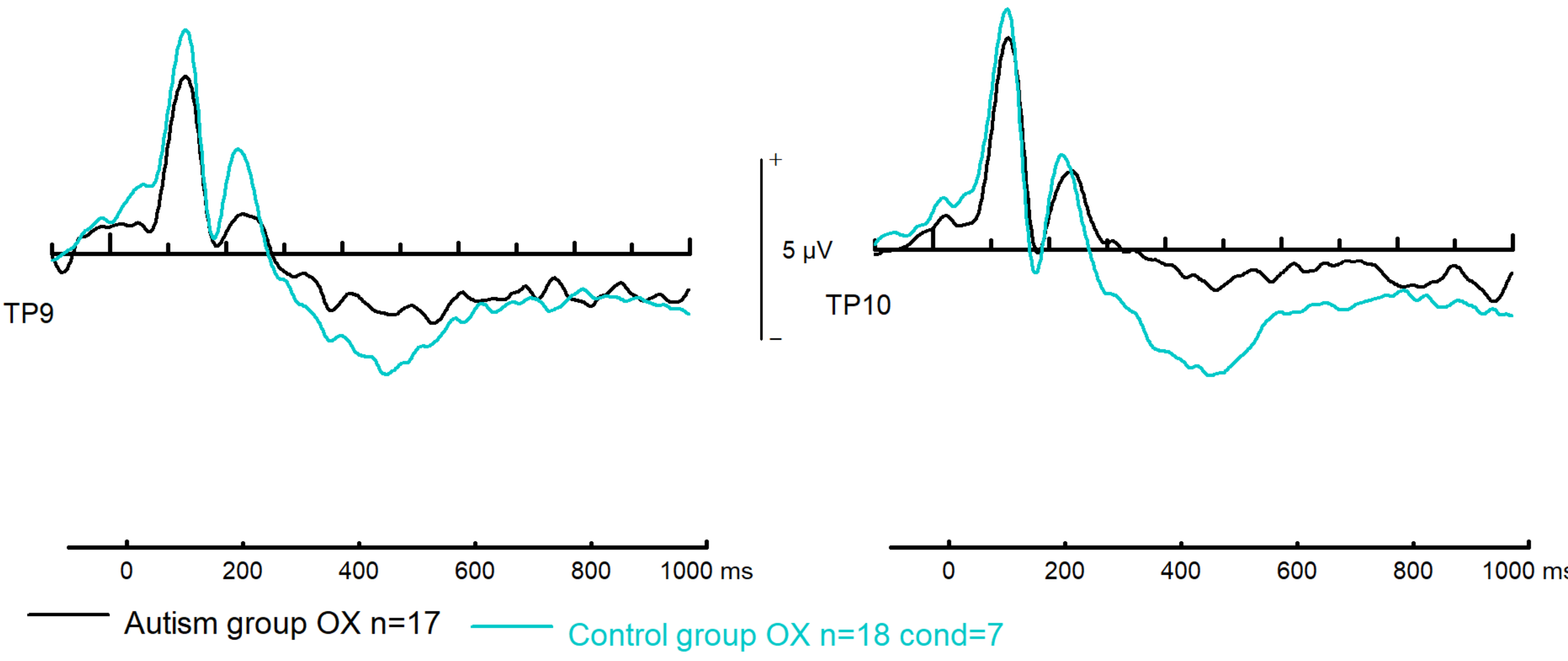

Condition= Faces NotO, NotX

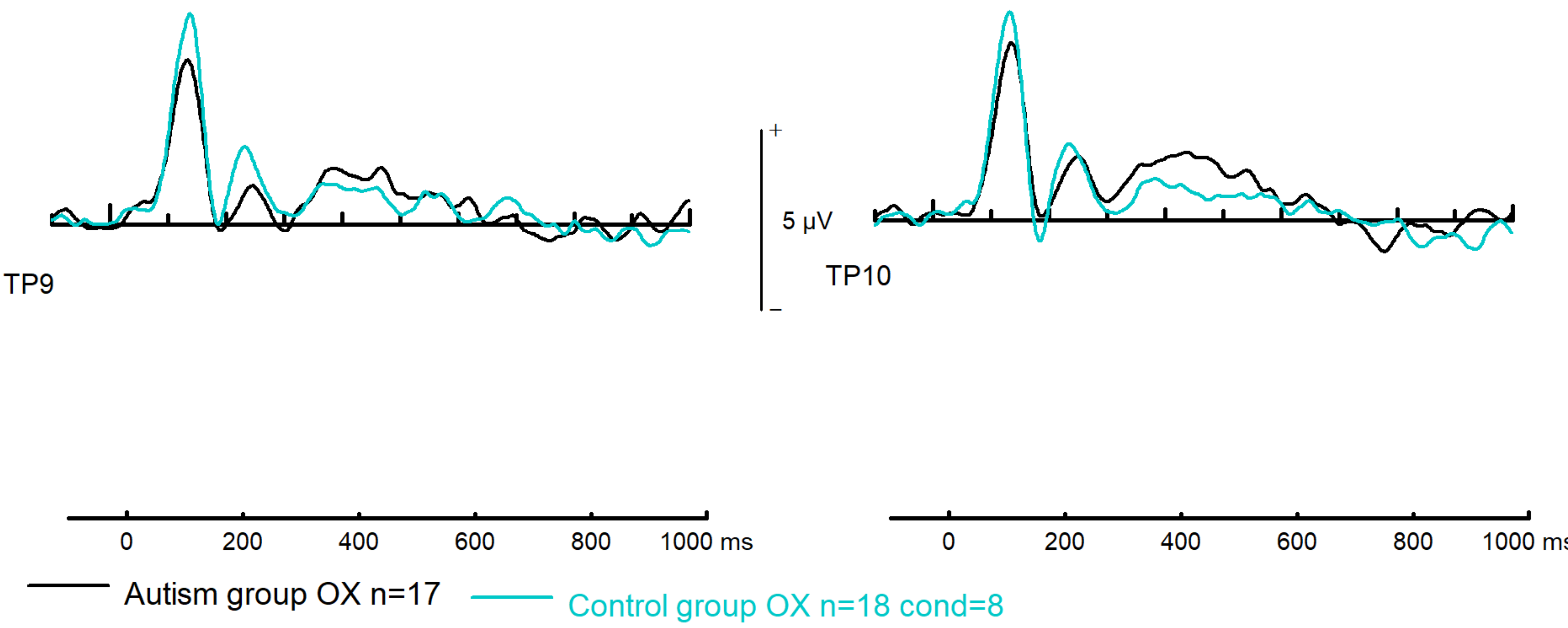

The contingent negative variation

- **ERPs:** Epochs were established offline from from −2200 to 200 ms relative to S2 for the additional analysis of the contingent negative variation (CNV) only. Thresholds for subsequent automatic artifact rejection were 100 µV for amplitude, 75 µV for gradient, and 0.01 µV for low signal. Baseline correction of ERP amplitudes for the CNV analysis was performed for the interval from −200 to 0 ms relative to S1 (−2200 to -2000 ms relative to S2). ERPs were averaged separately for each experimental condition, digitally low-pass filtered (data were filtered low pass 30 Hz 24db/oct and high pass 0.30 Hz 12db/oct, both zero phase shift), and recalculated to average reference, excluding the vertical EOG. Component of interest is CNV at three groups of electrodes: central (C3, Cz, C4), frontal (F3, Fz, F4), and parietal (P3, Pz, P4).

Condition= Letters OX

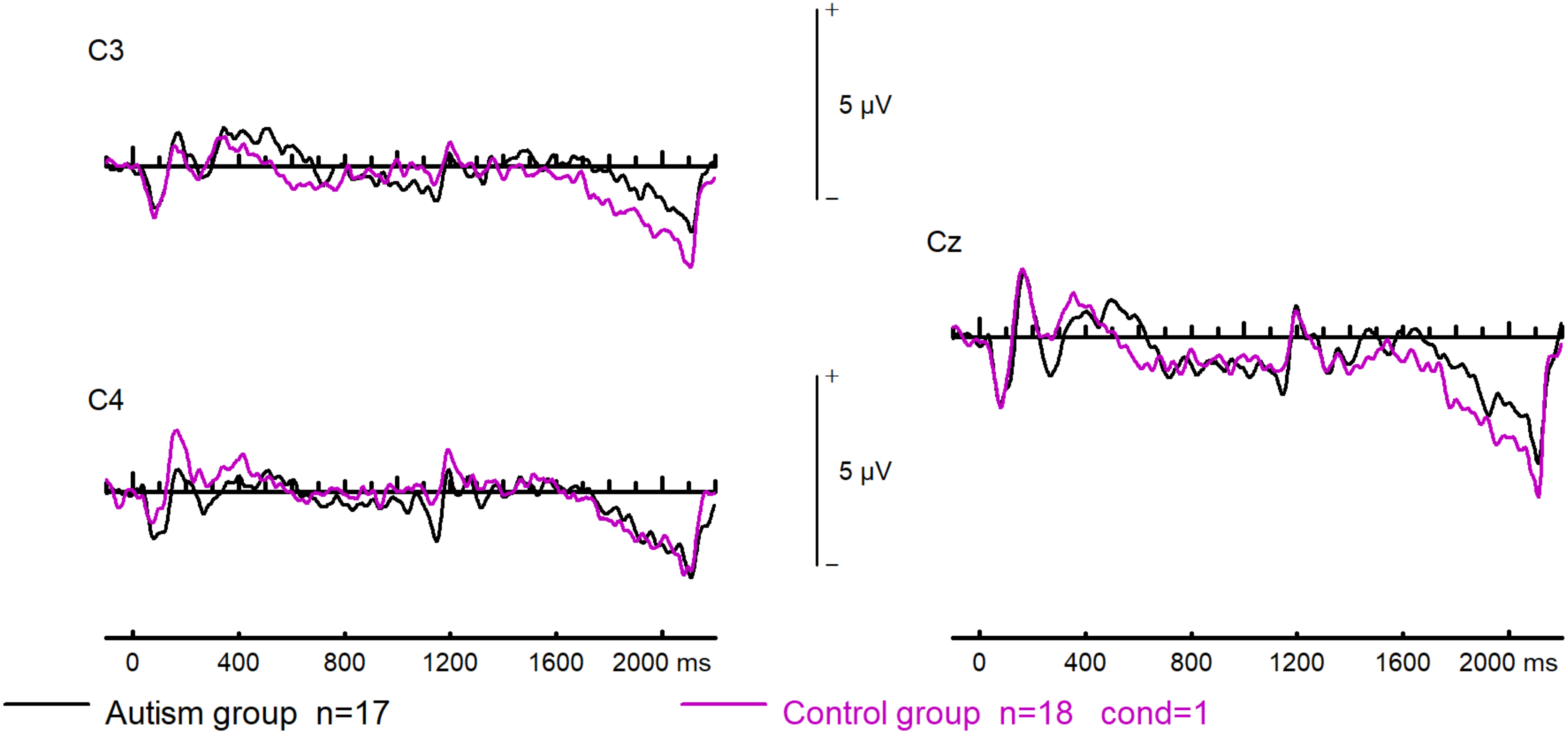

Condition= Letters Not O, X

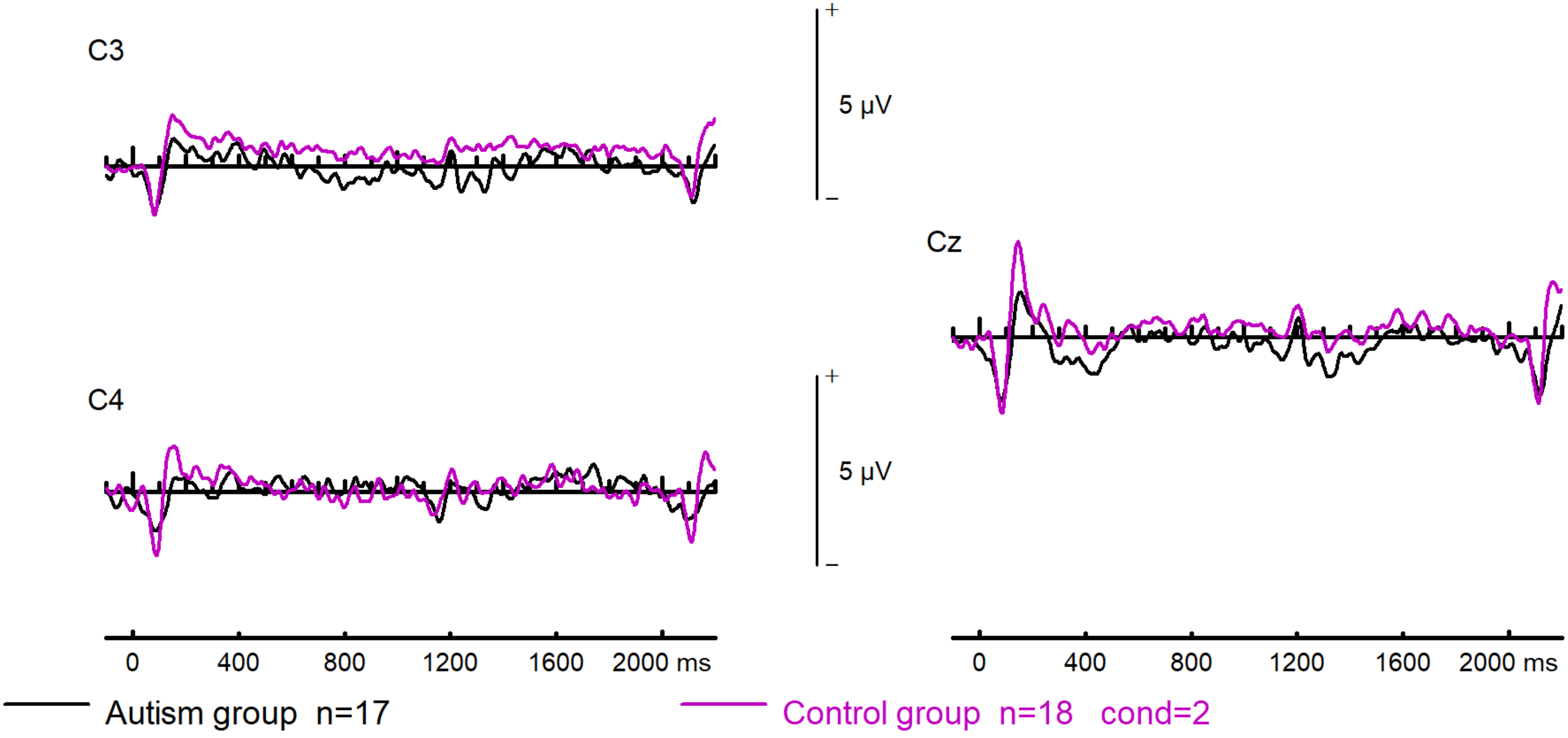

Condition= Letters O, NotX

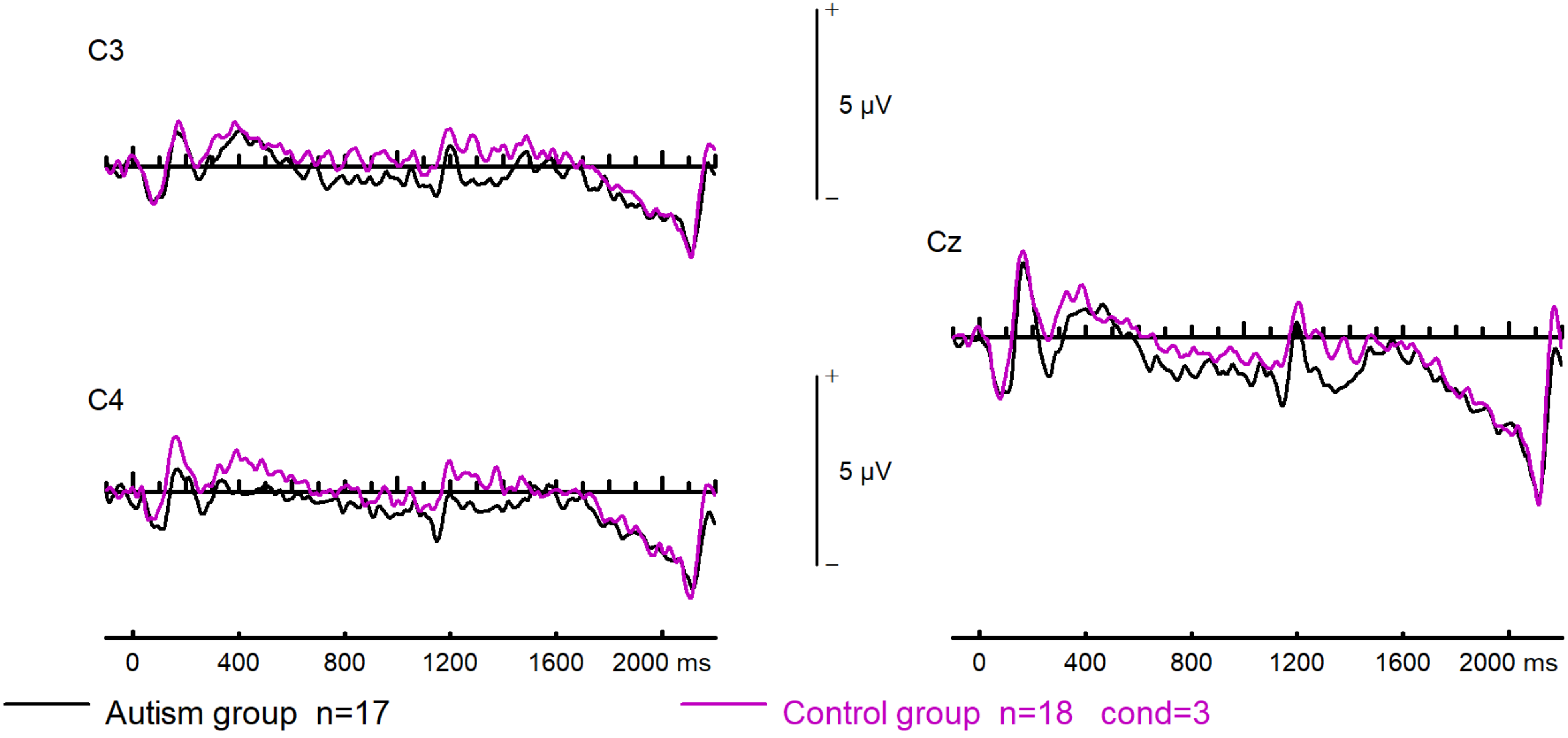

Condition= Letters NotO, NotX

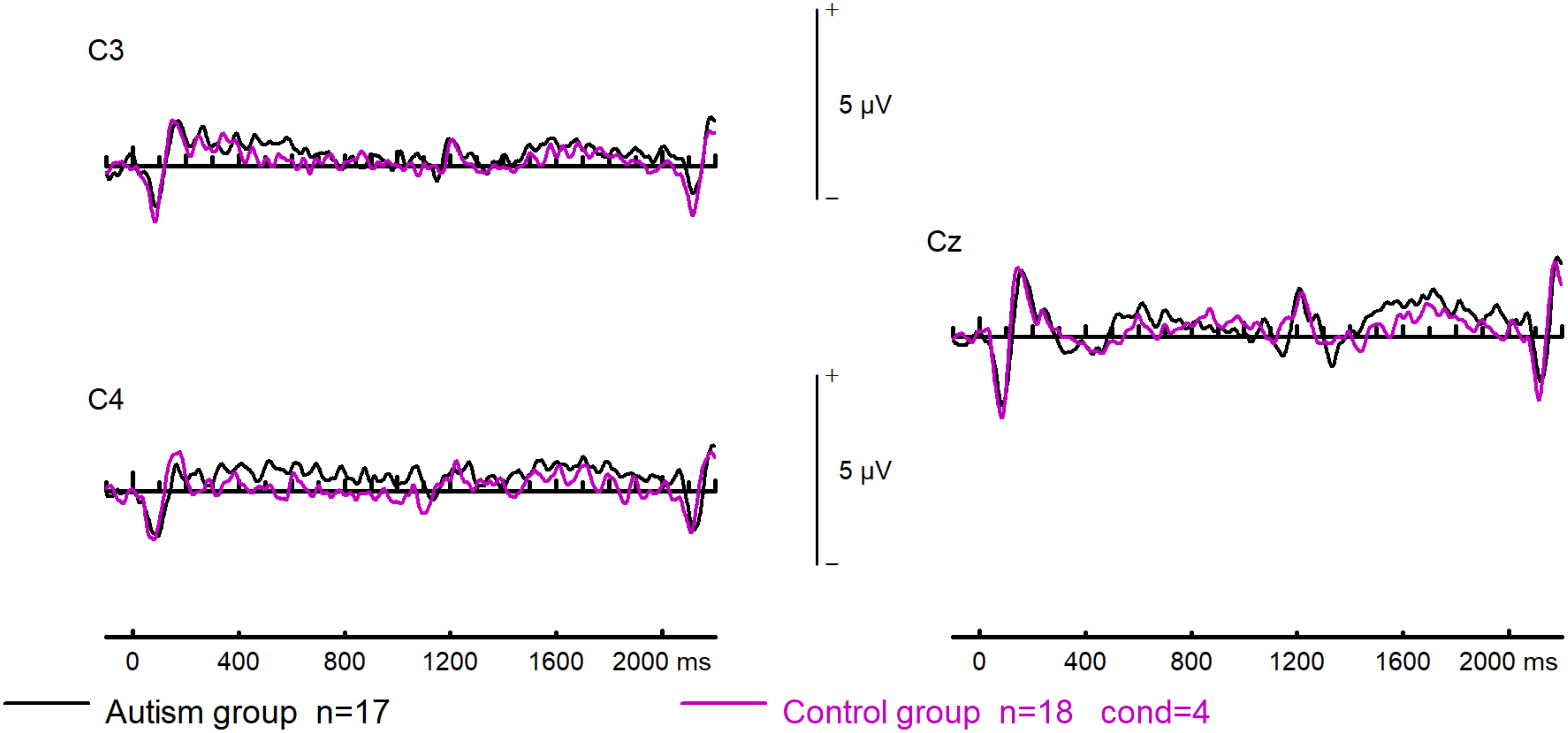

Condition= Faces OX

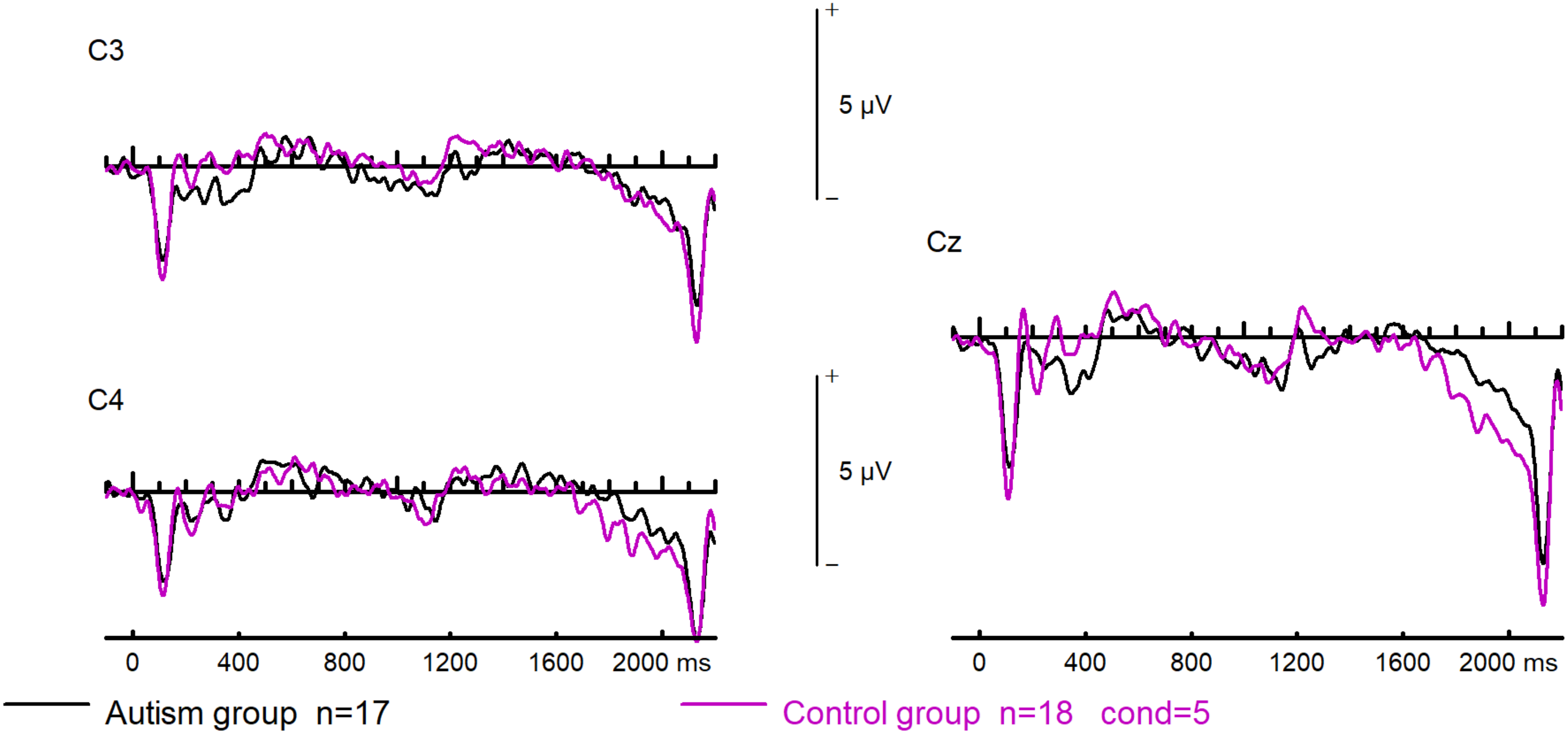

Condition= Faces NotO,X

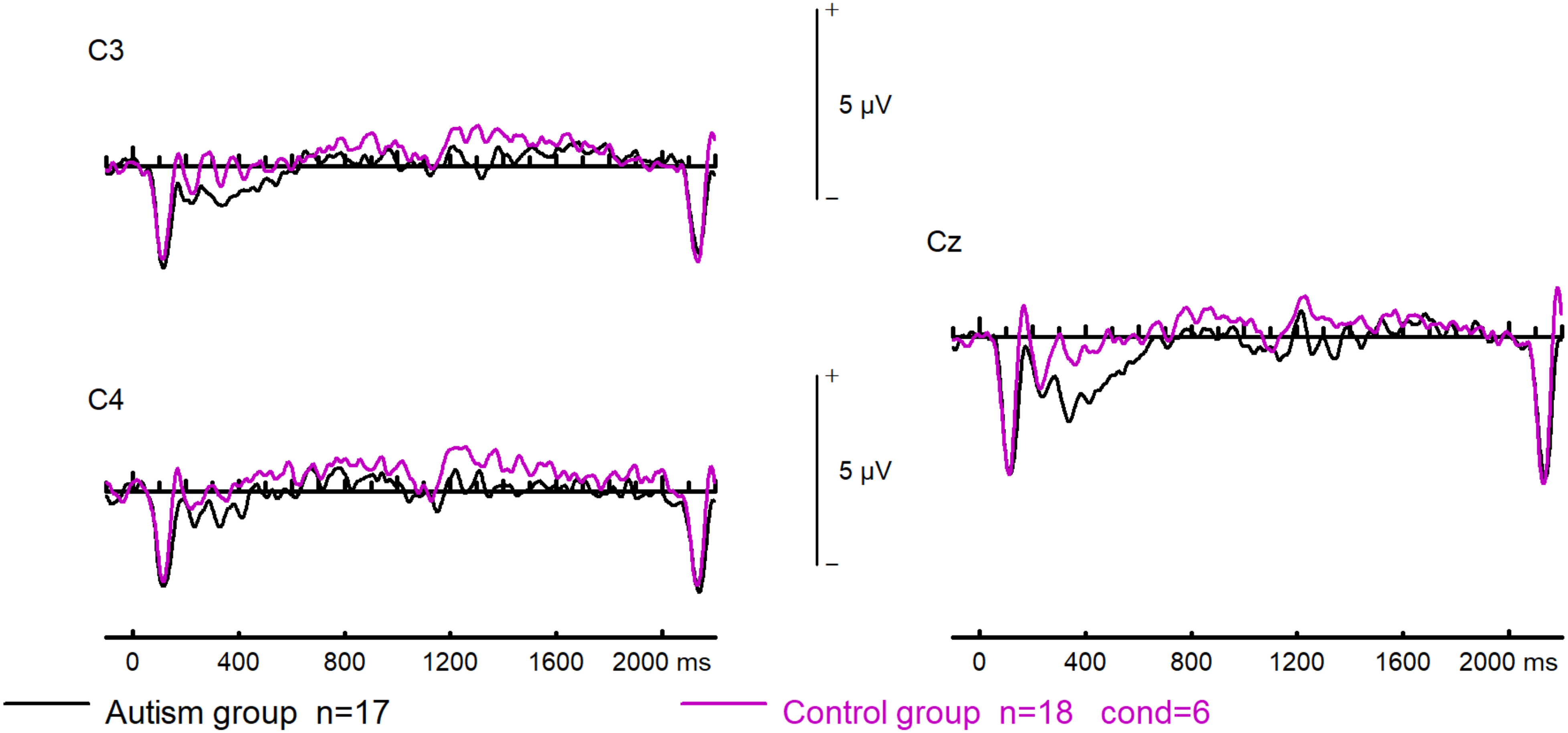

Condition= Faces O, Not X

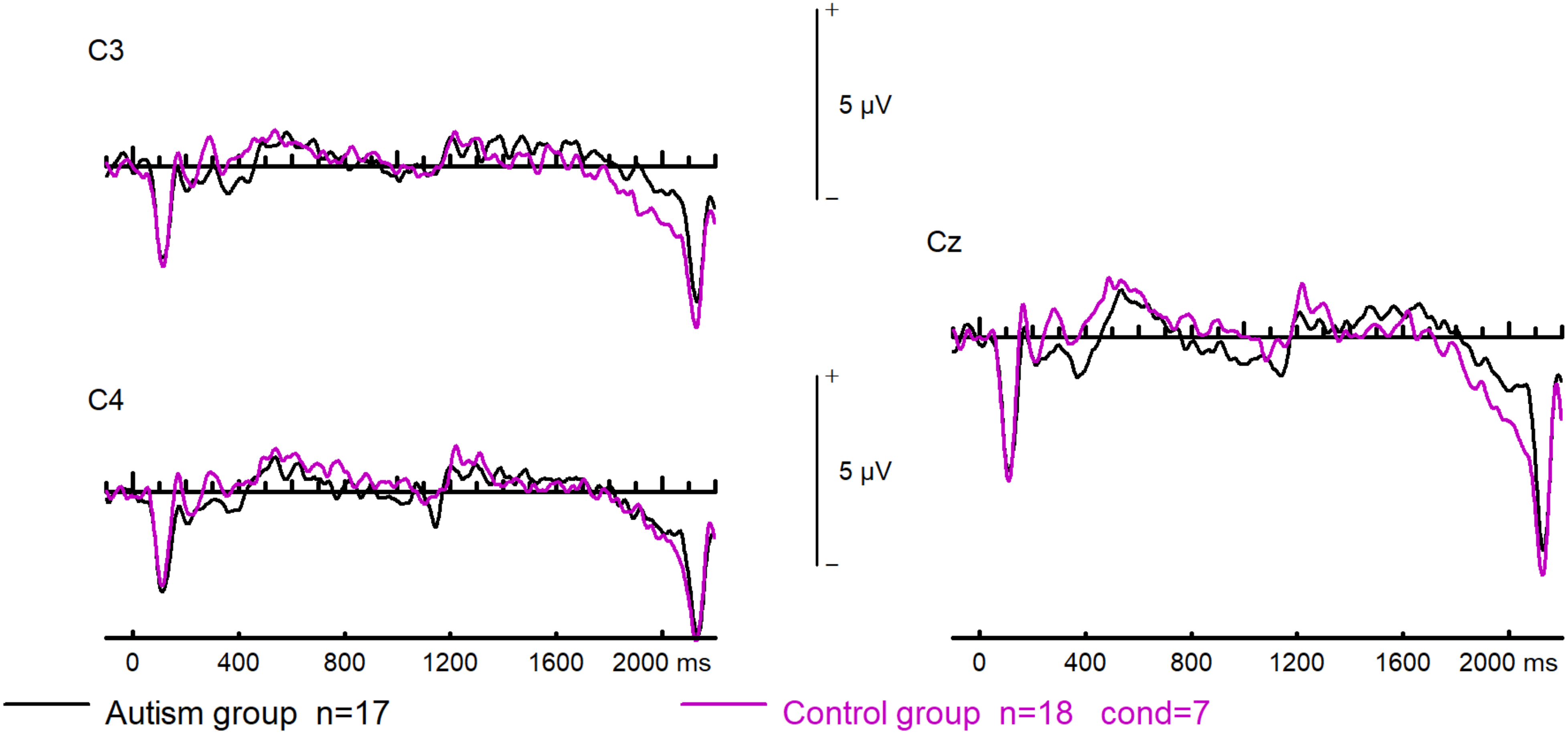

Condition= Faces NotO, NotX

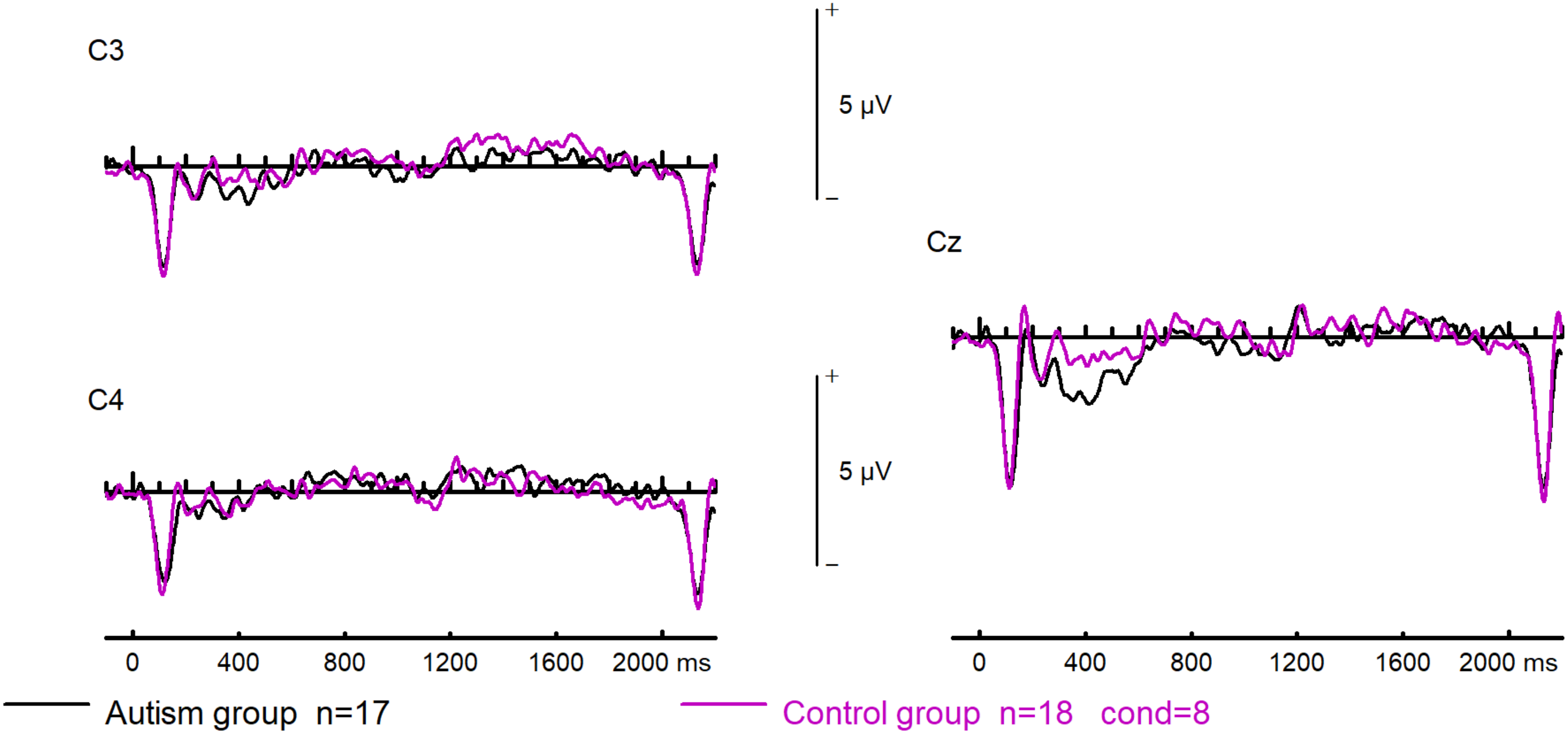

Condition= Letters OX

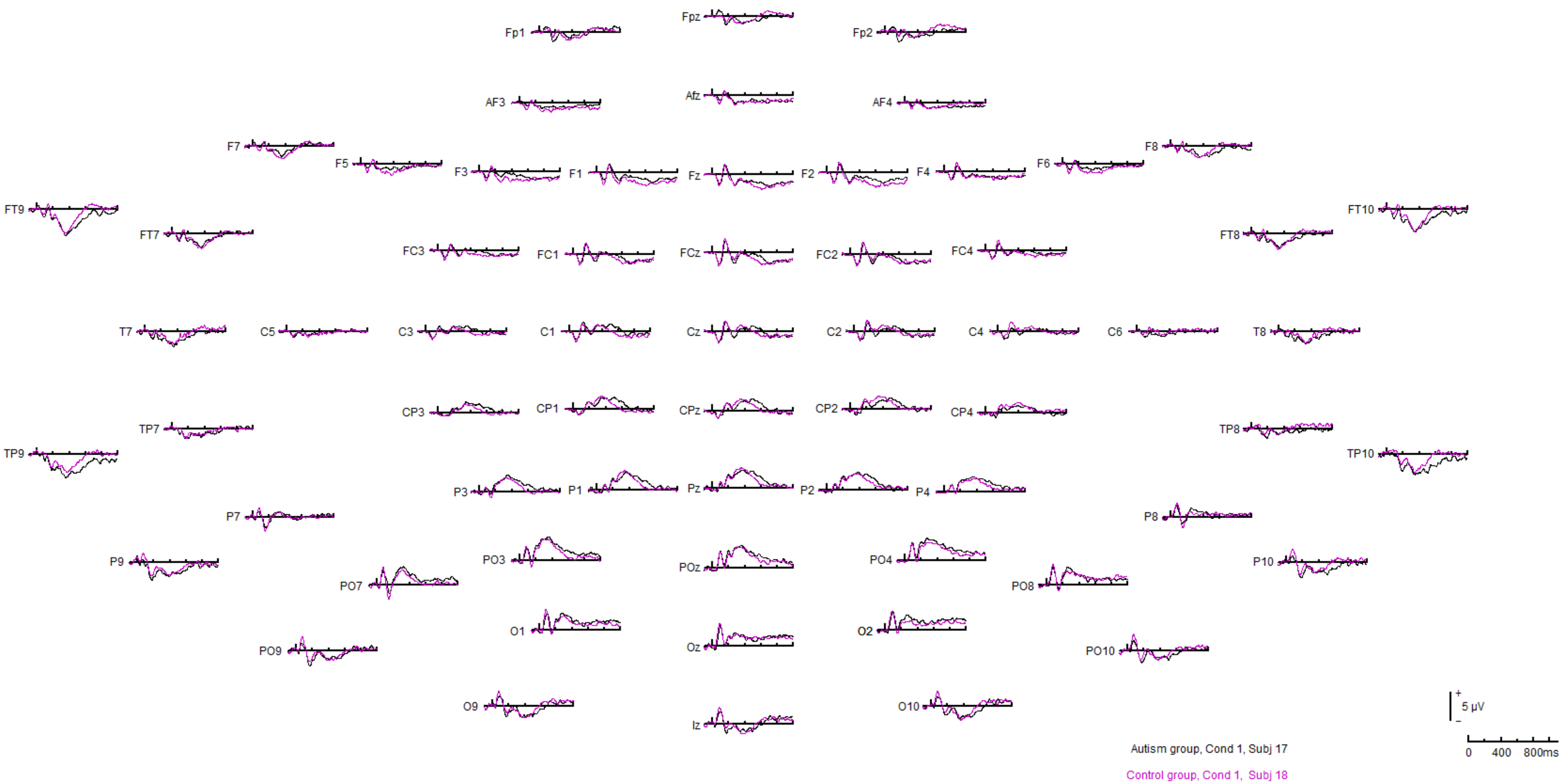

Condition= Letters Not O, X

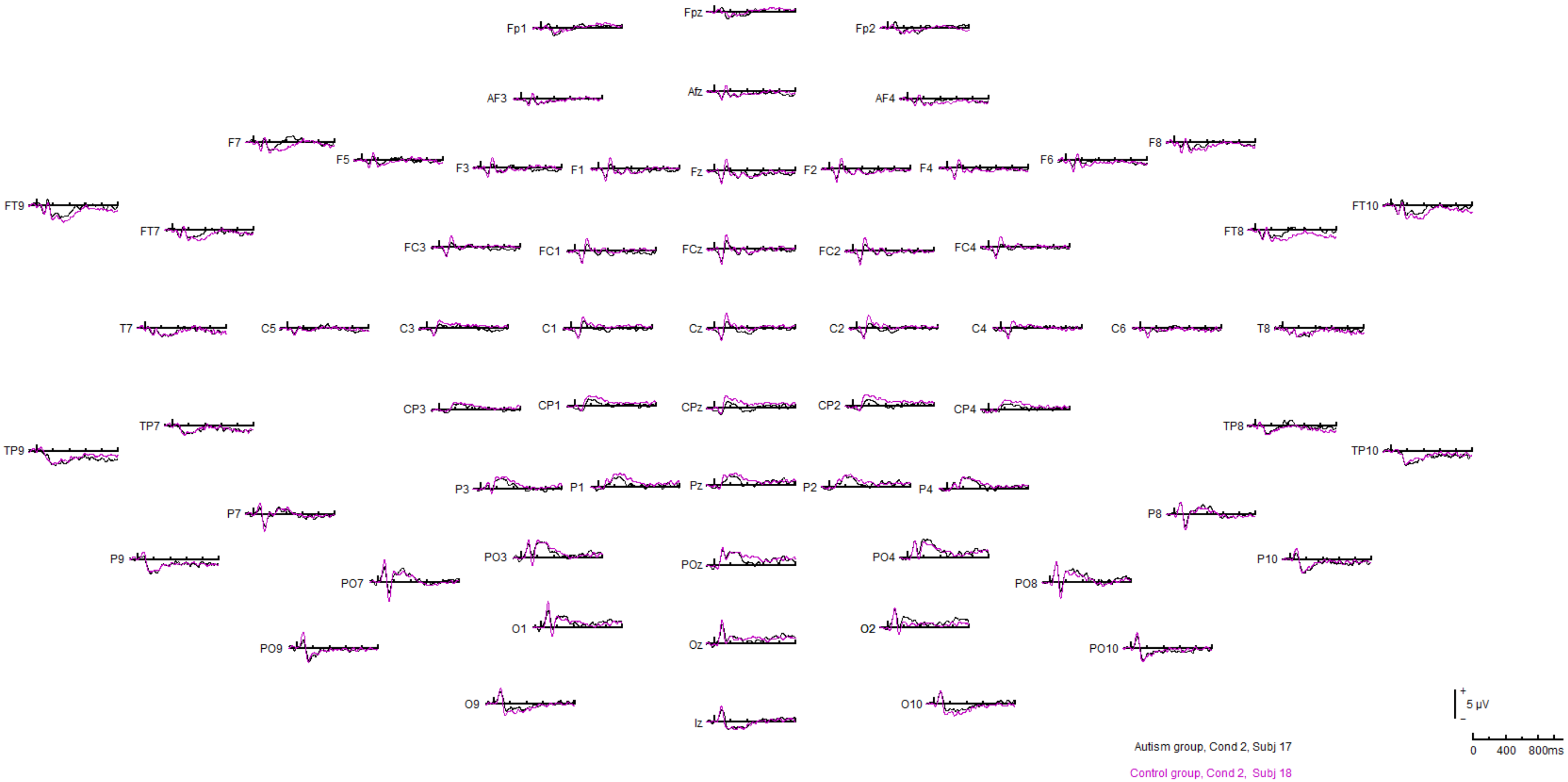

Condition= Letters O, NotX

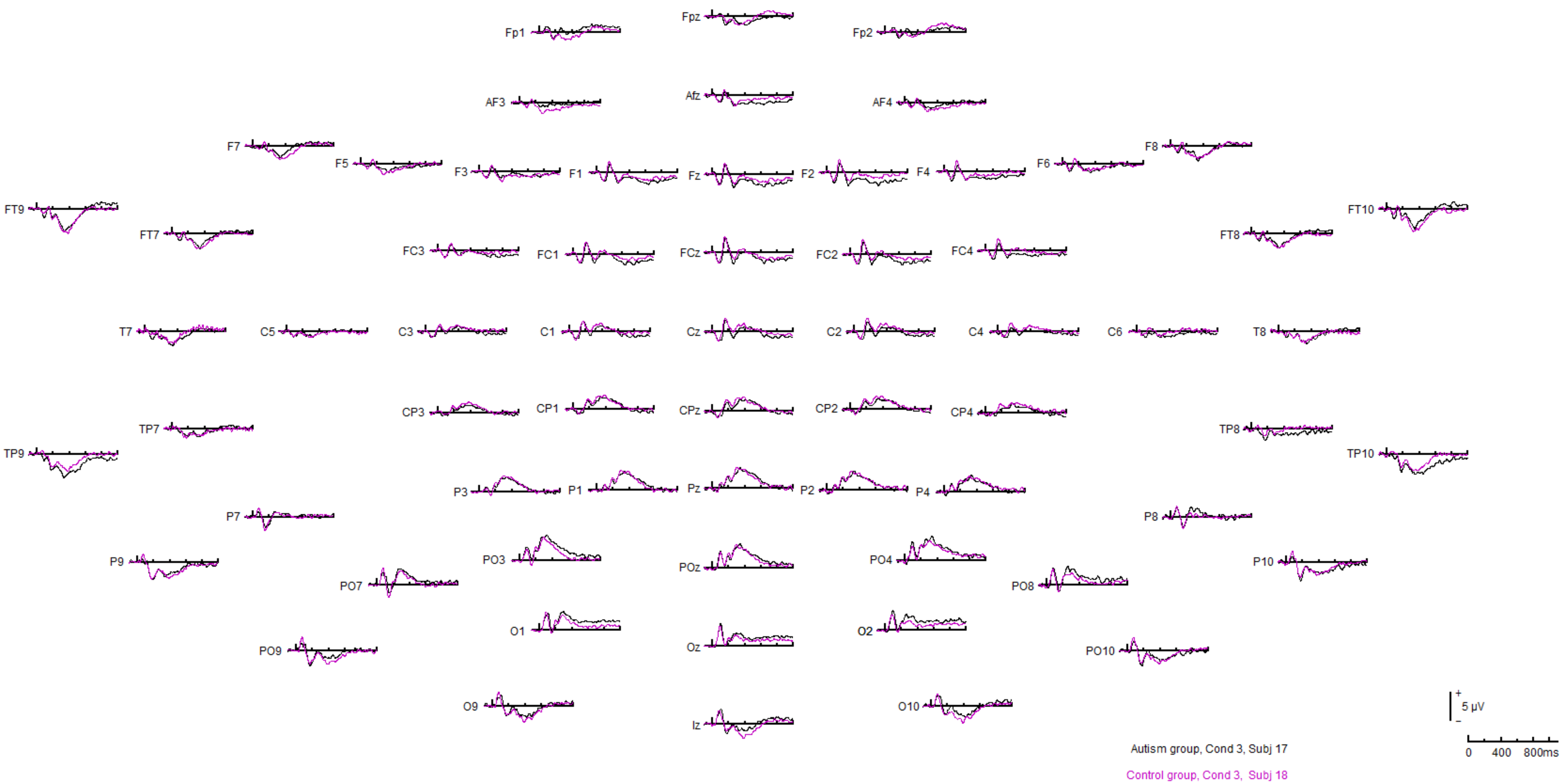

Condition= Letters NotO, NotX

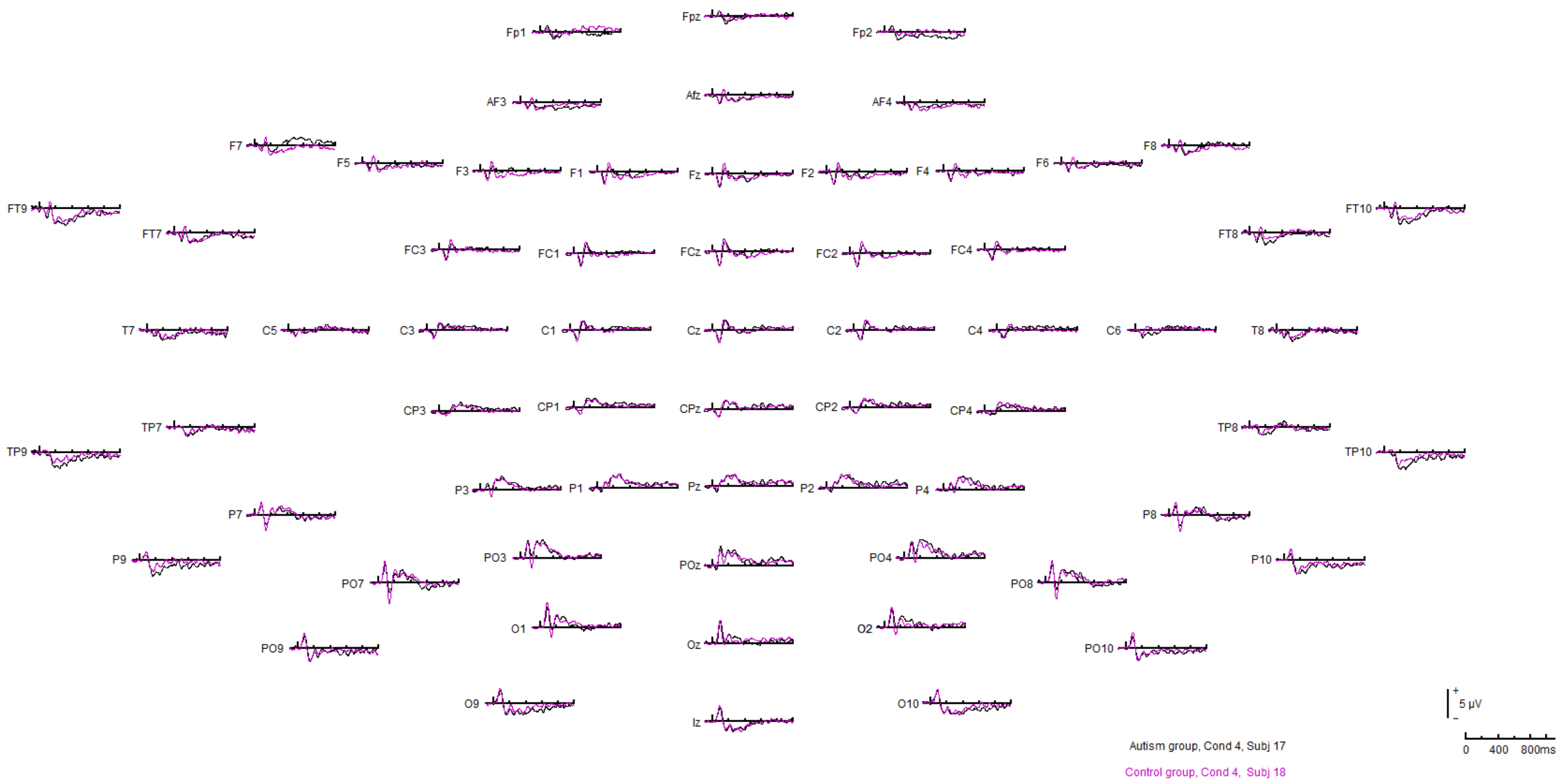

Condition= Faces OX

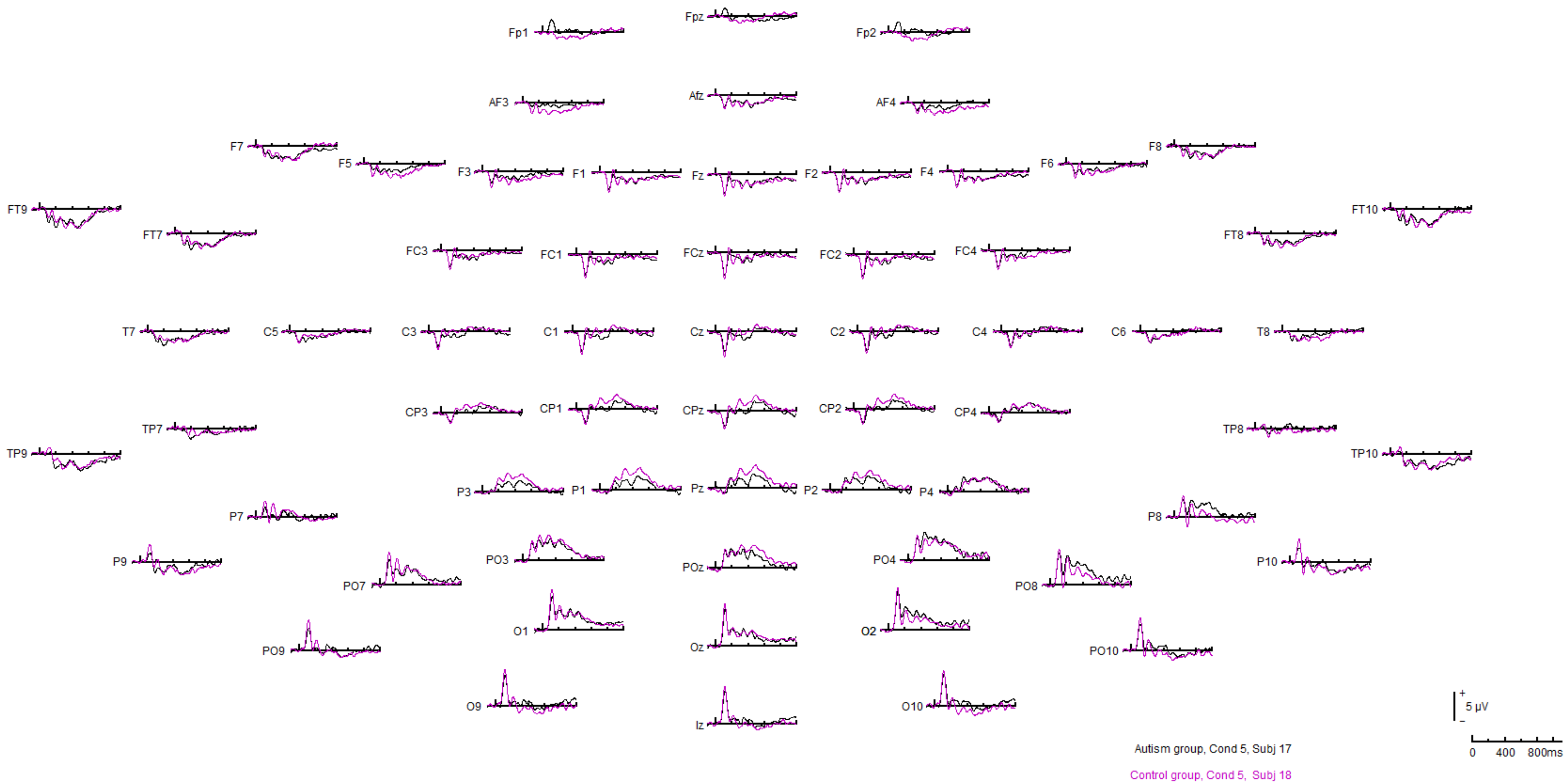

Condition= Faces NotO,X

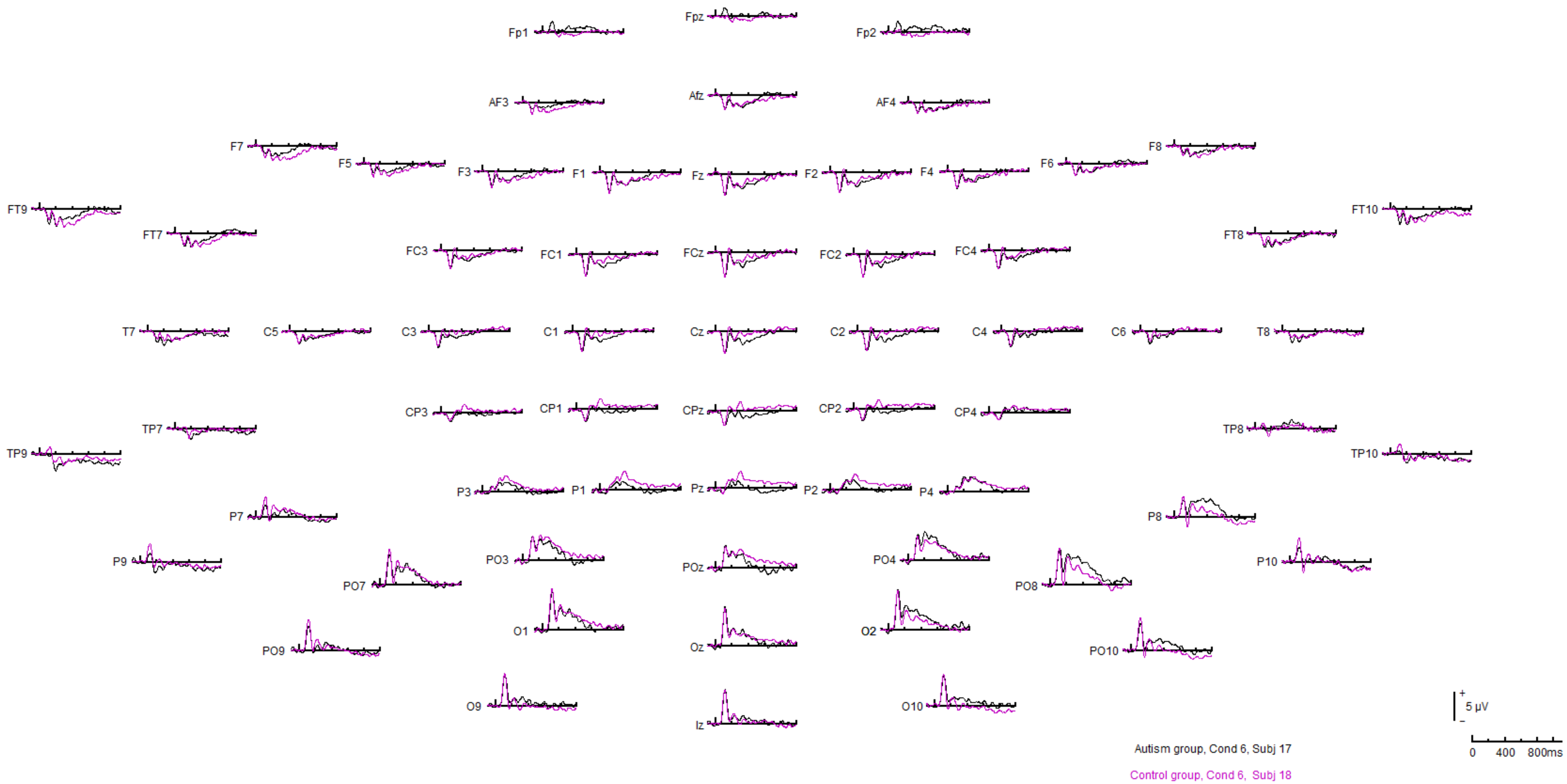

Condition= Faces O, Not X

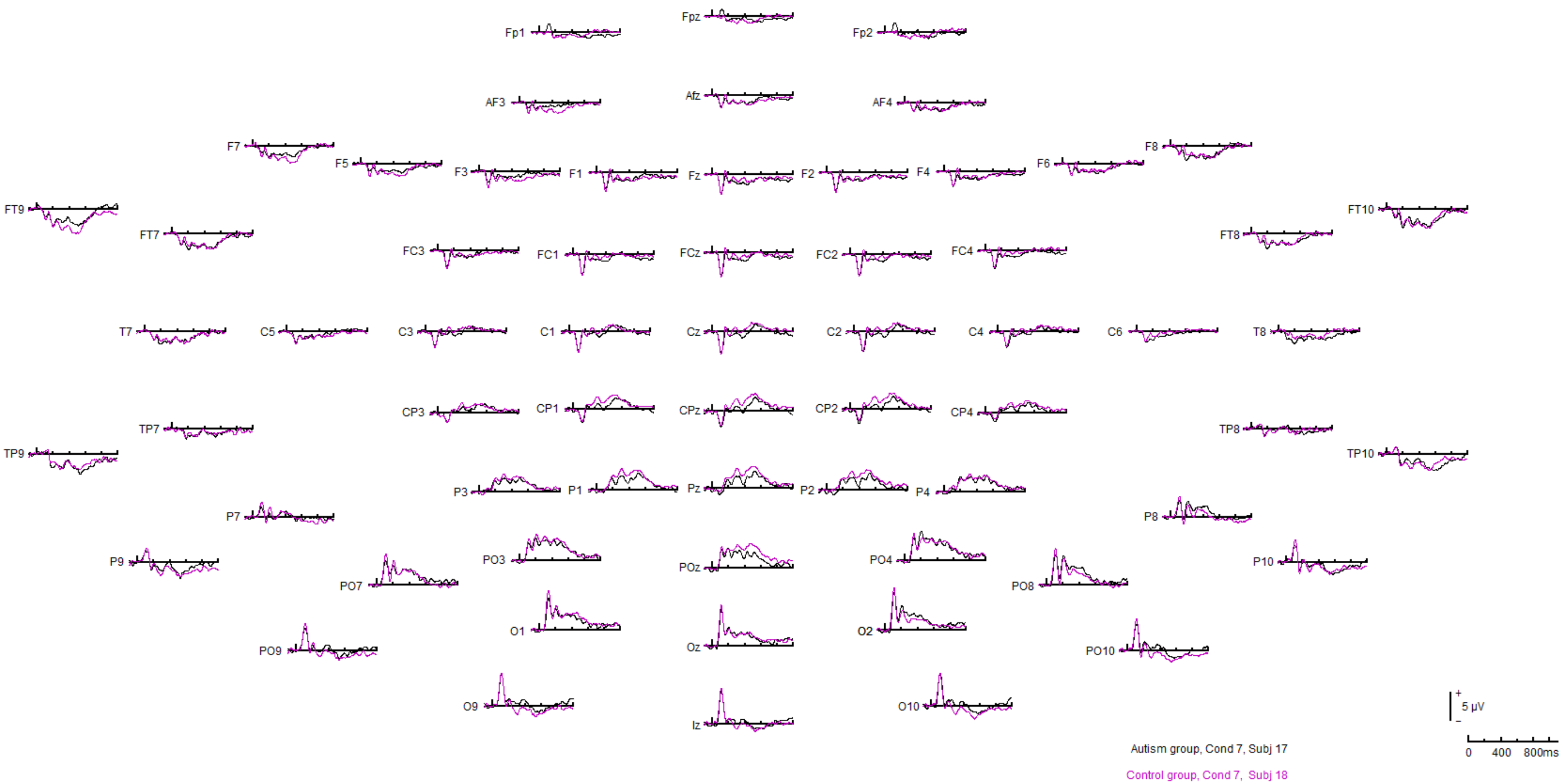

Condition= Faces NotO, NotX

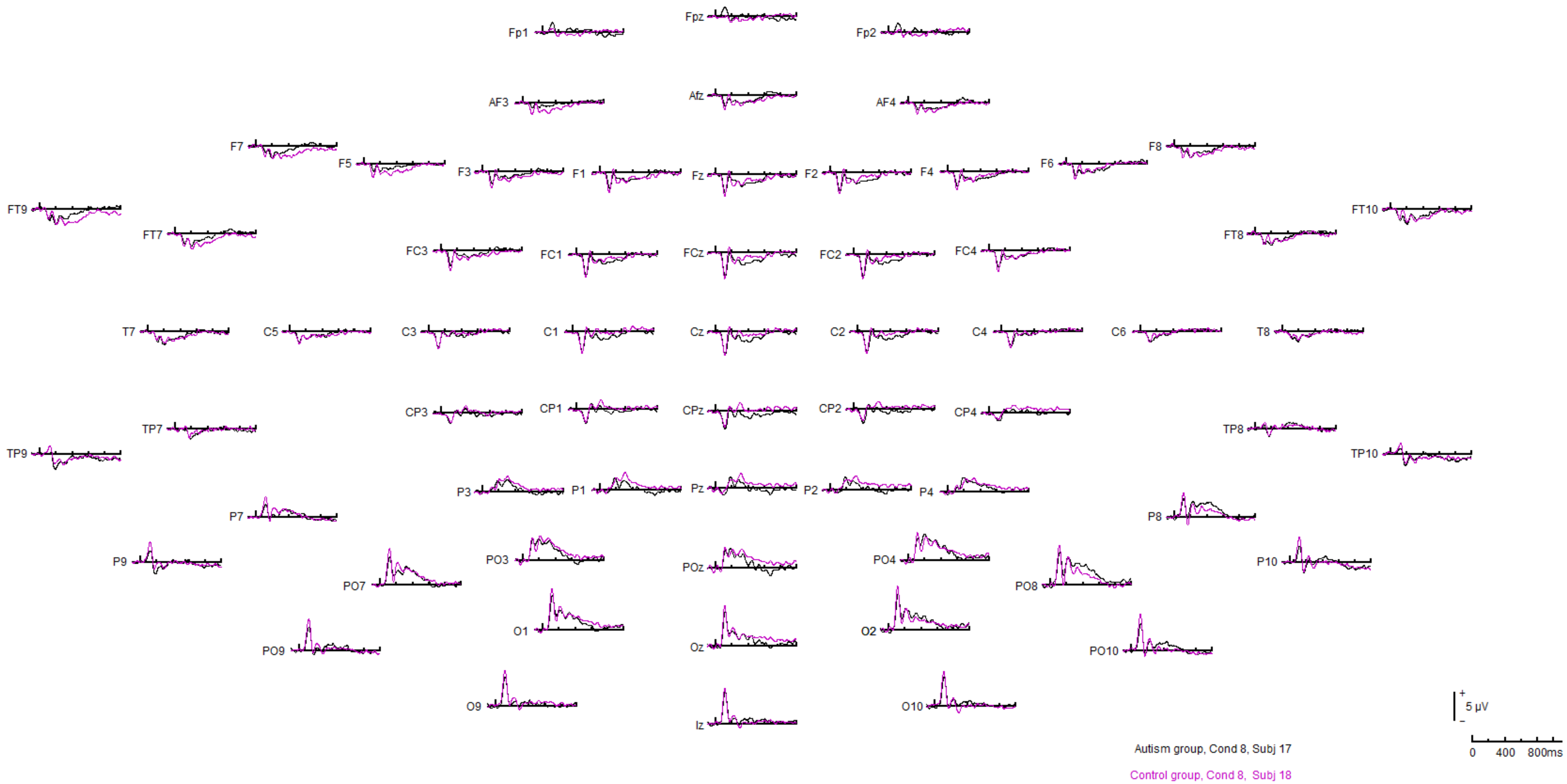

Condition= Letters OX

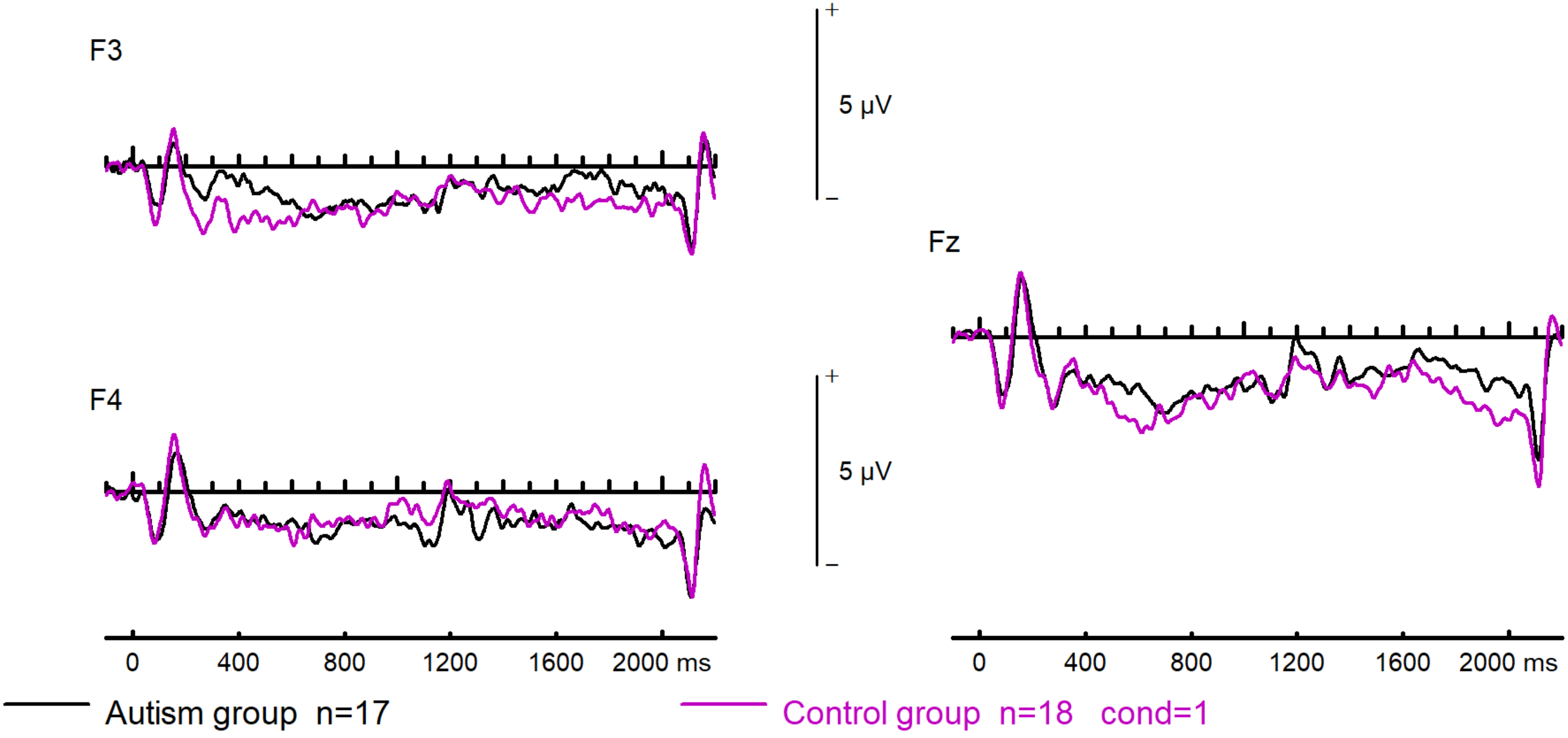

Condition= Letters Not O, X

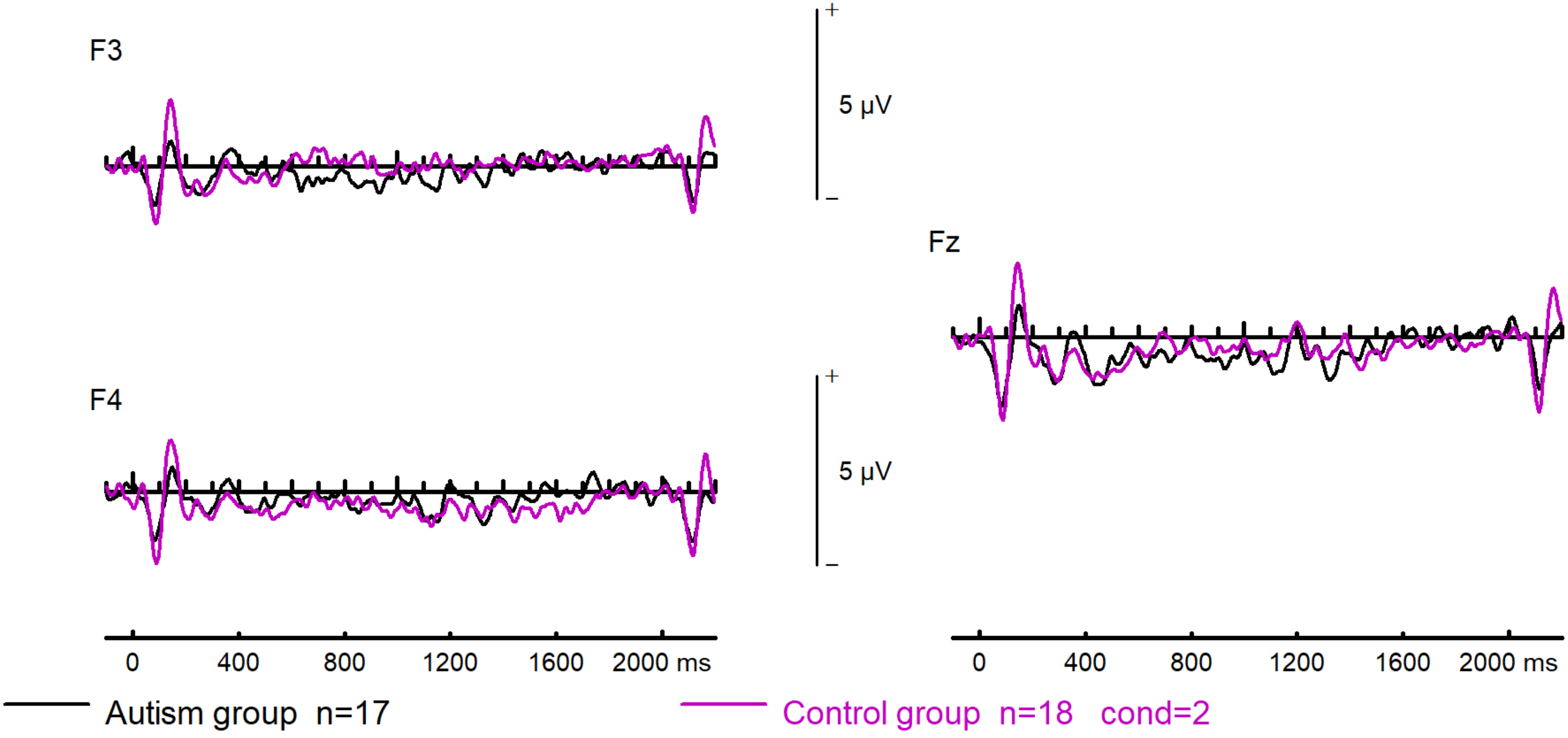

Condition= Letters O, NotX

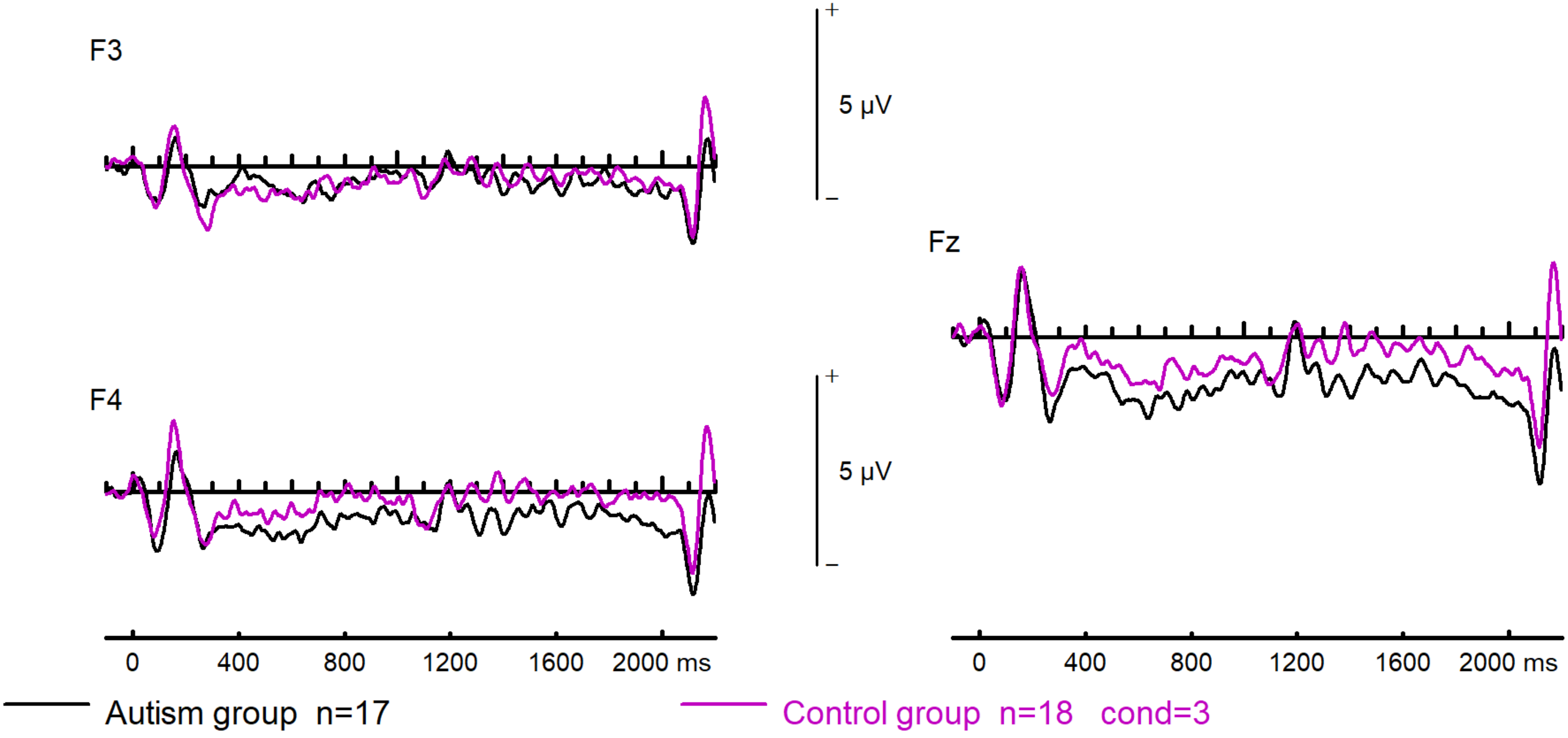

Condition= Letters NotO, NotX

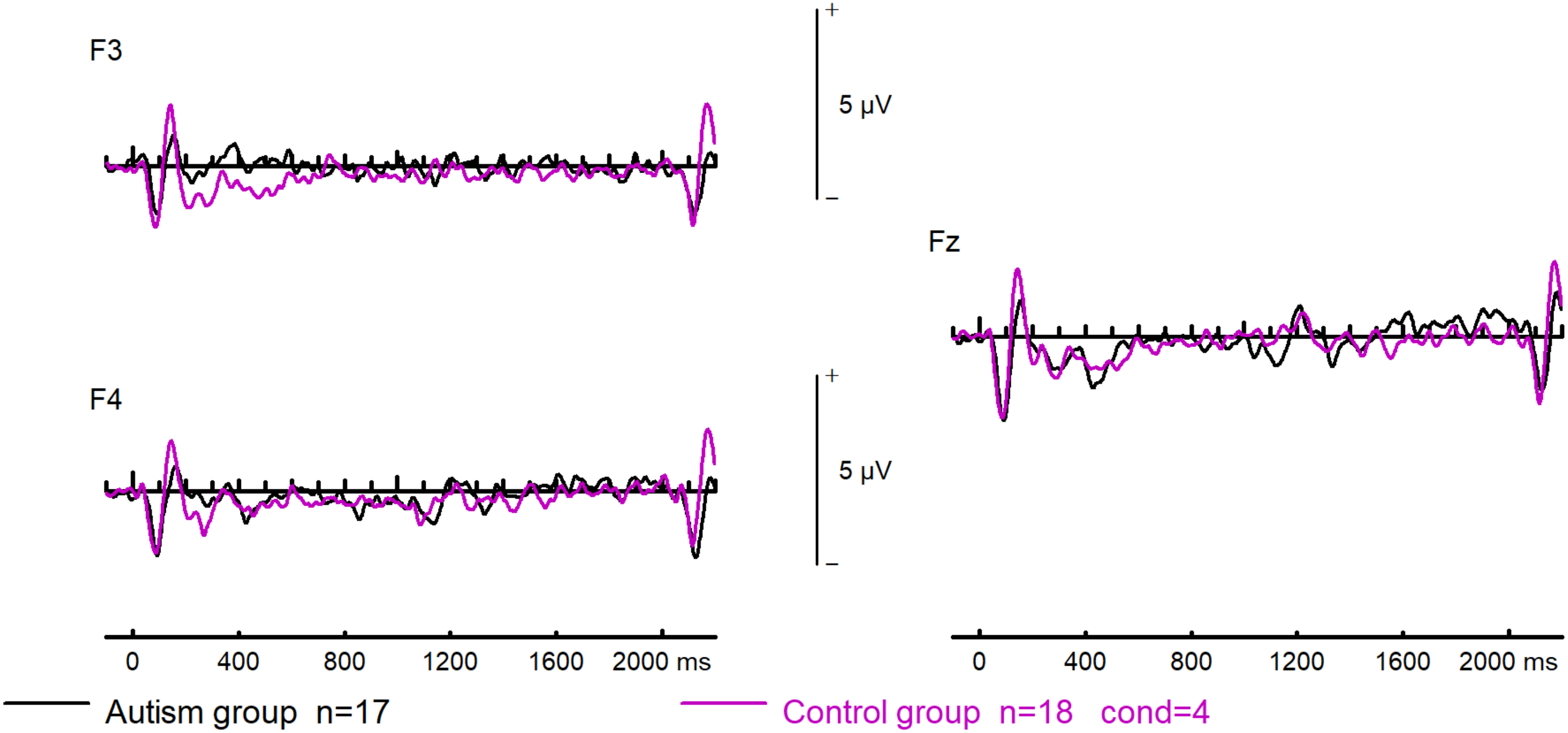

Condition= Faces OX

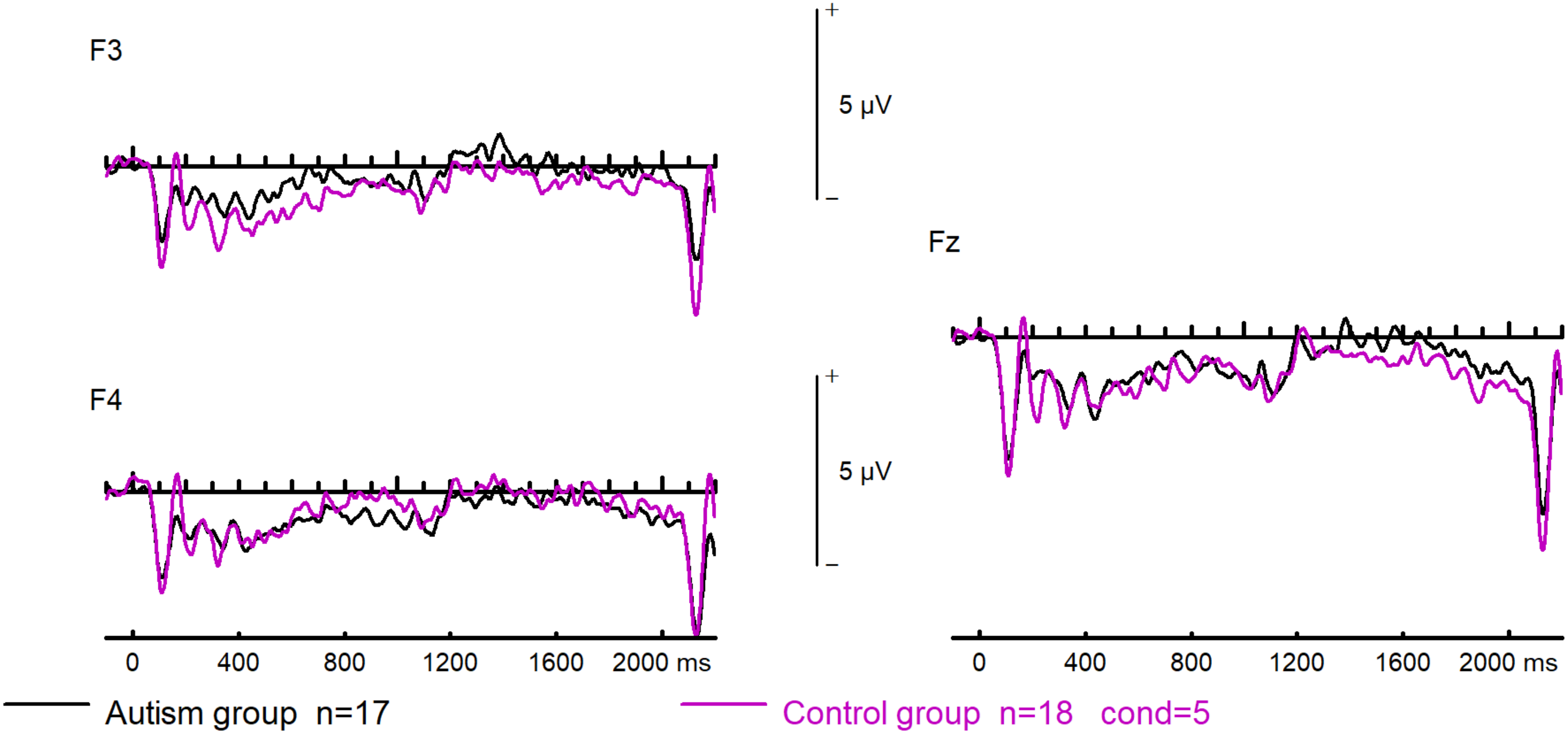

Condition= Faces NotO,X

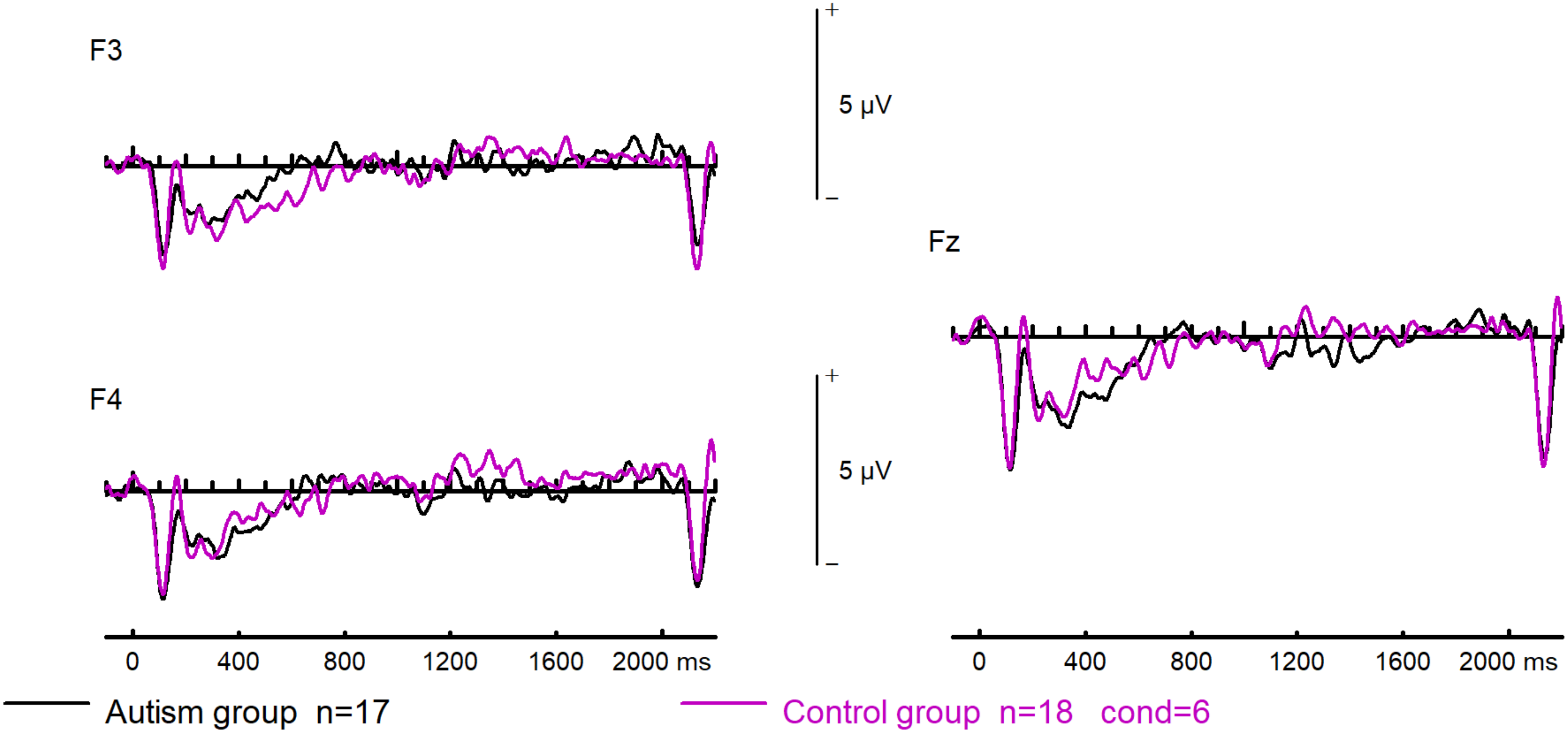

Condition= Faces O, Not X

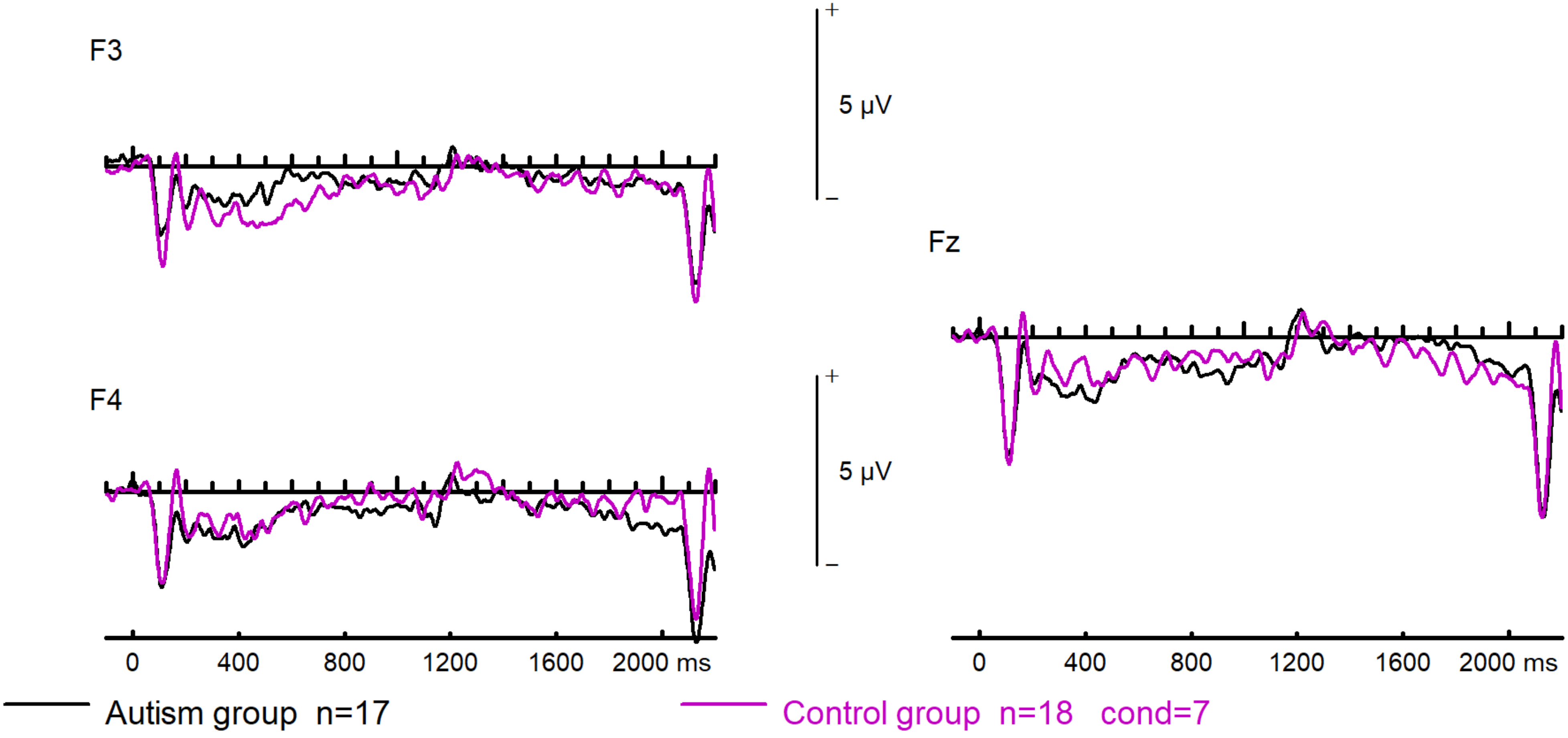

Condition= Faces NotO, NotX

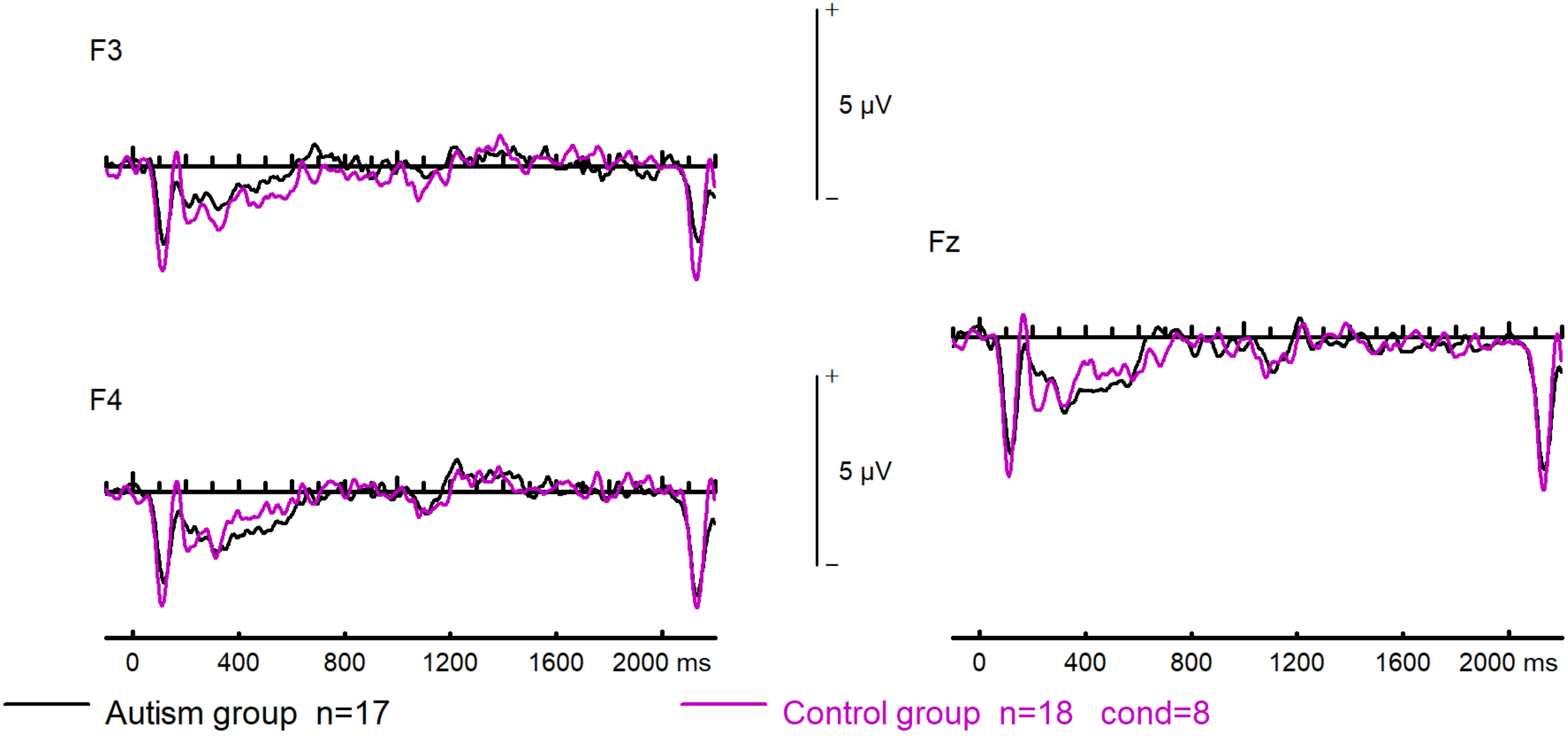

Condition= Letters OX

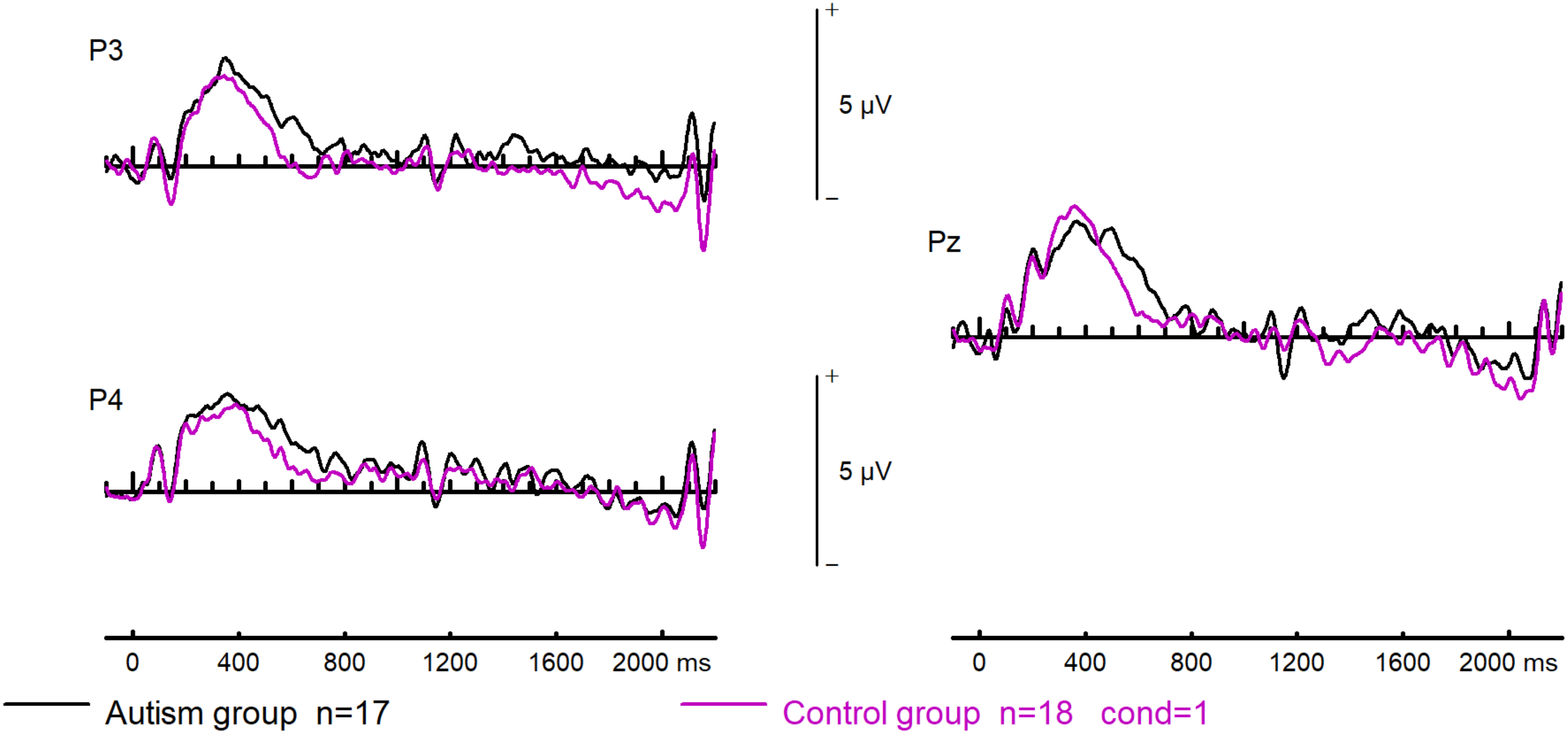

Condition= Letters Not O, X

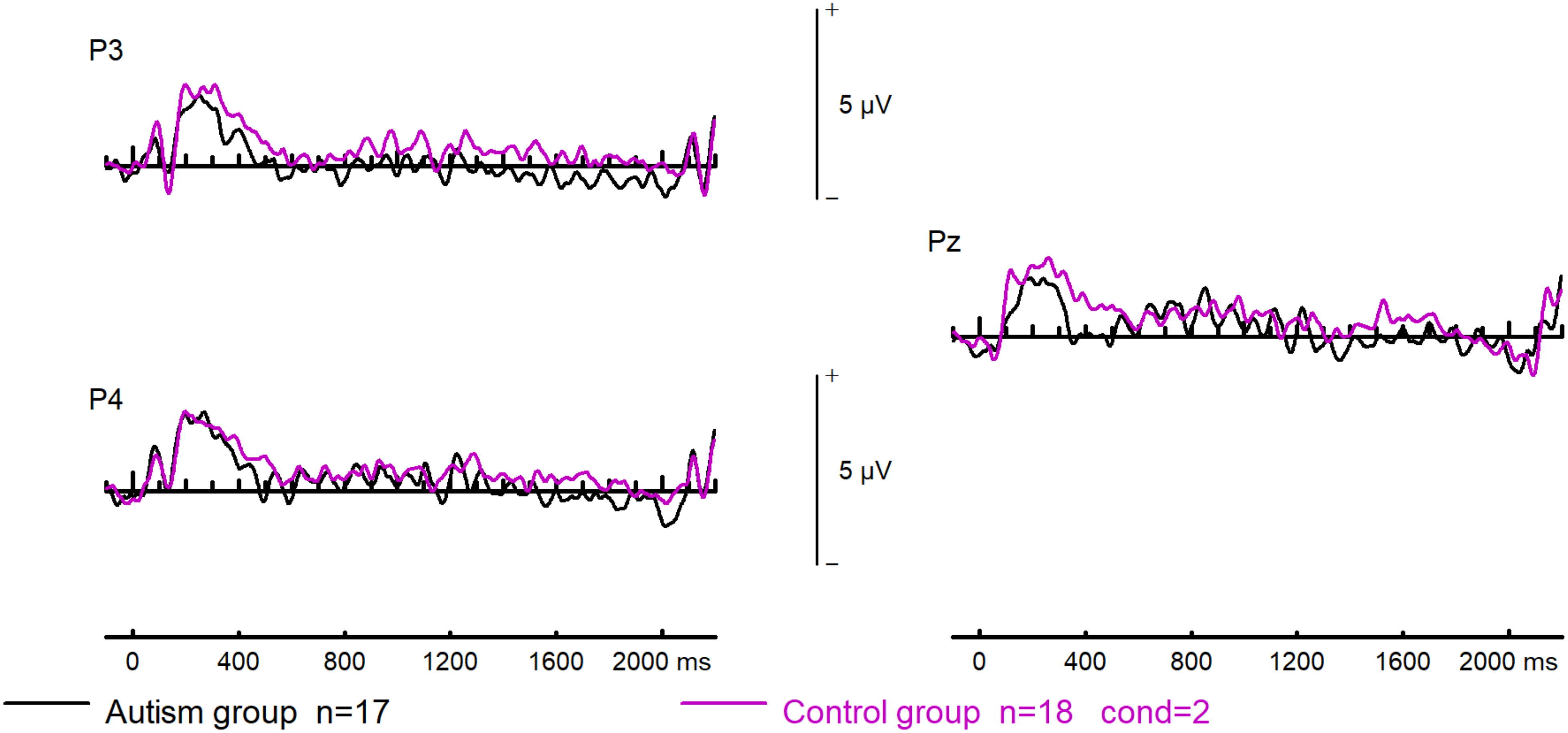

Condition= Letters O, NotX

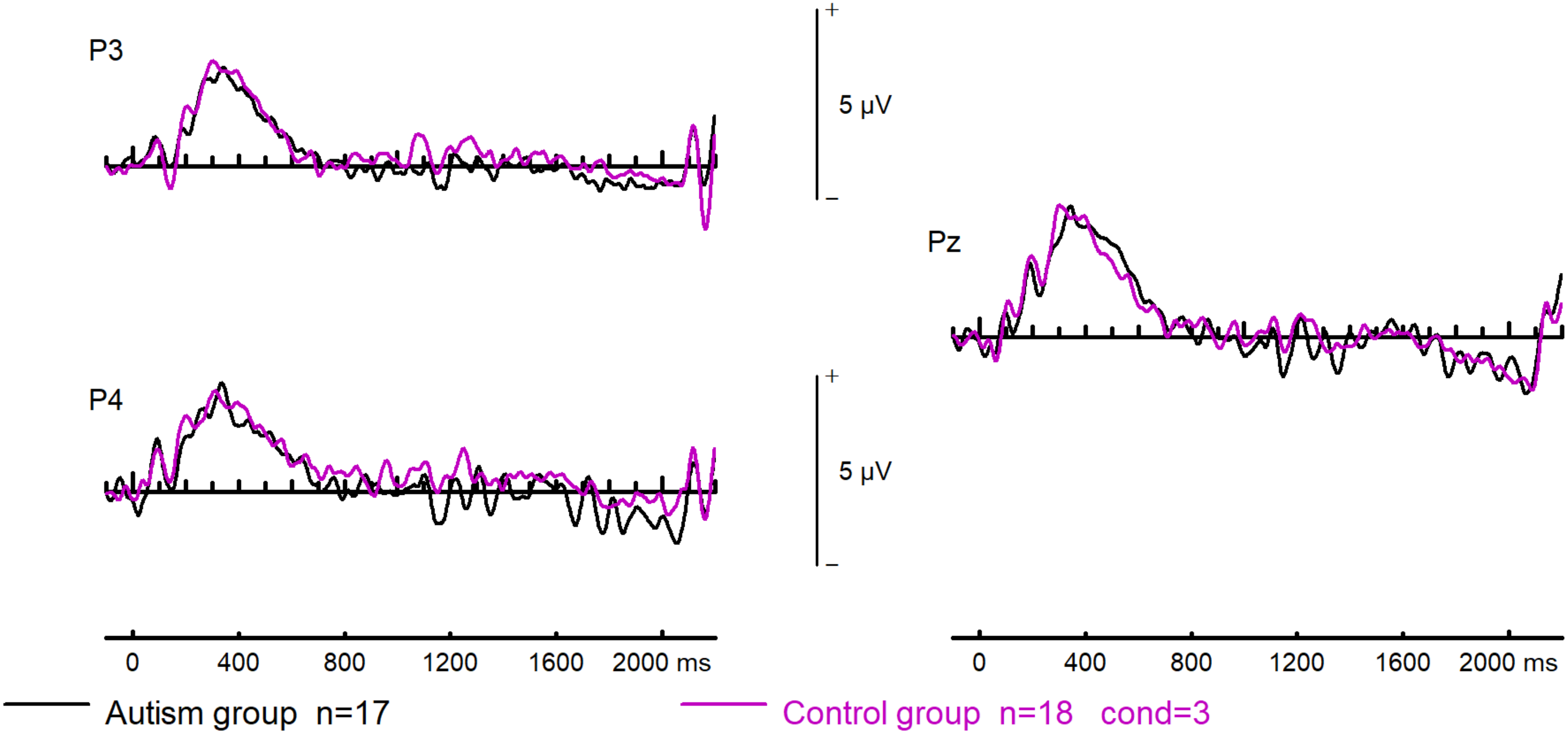

Condition= Letters NotO, NotX

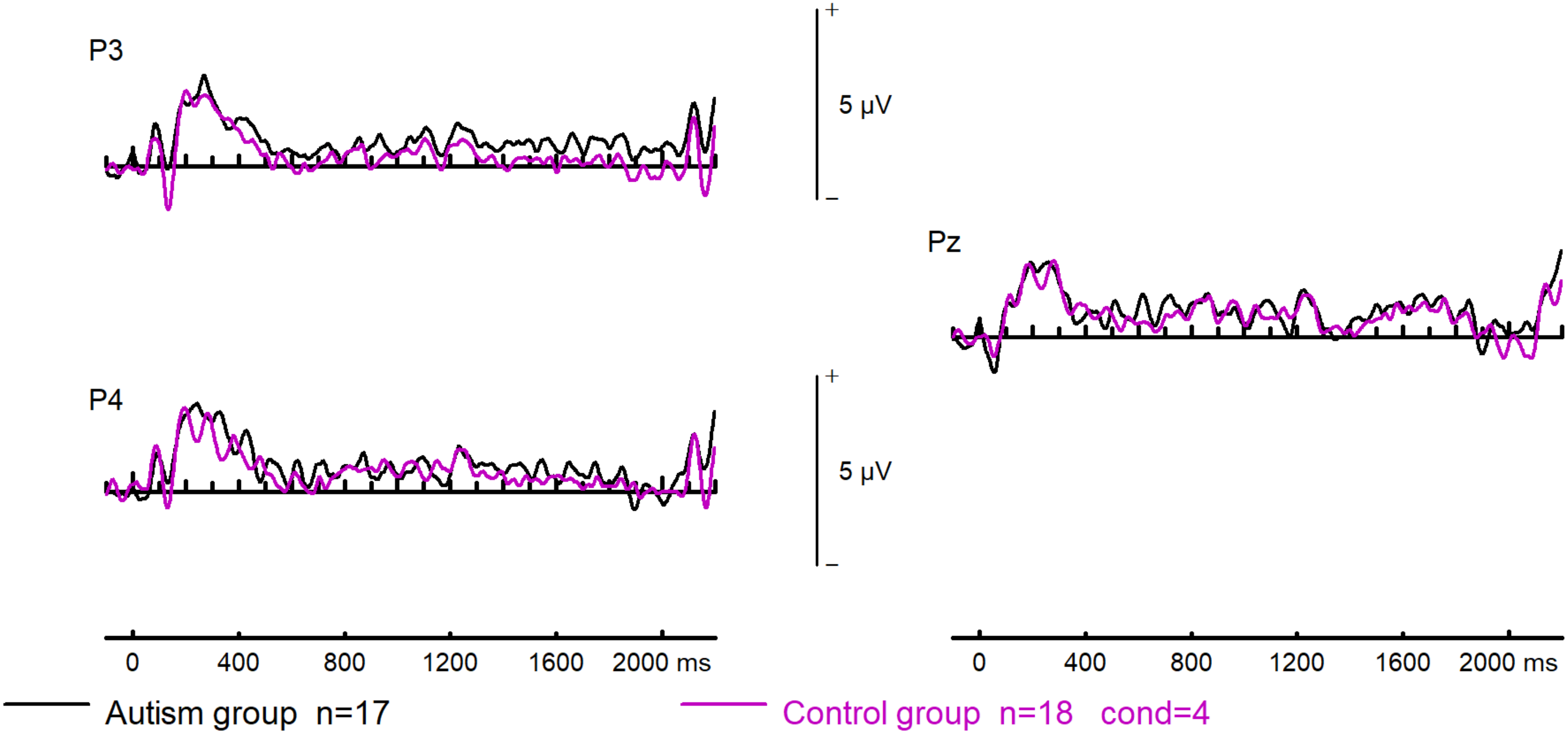

Condition= Faces OX

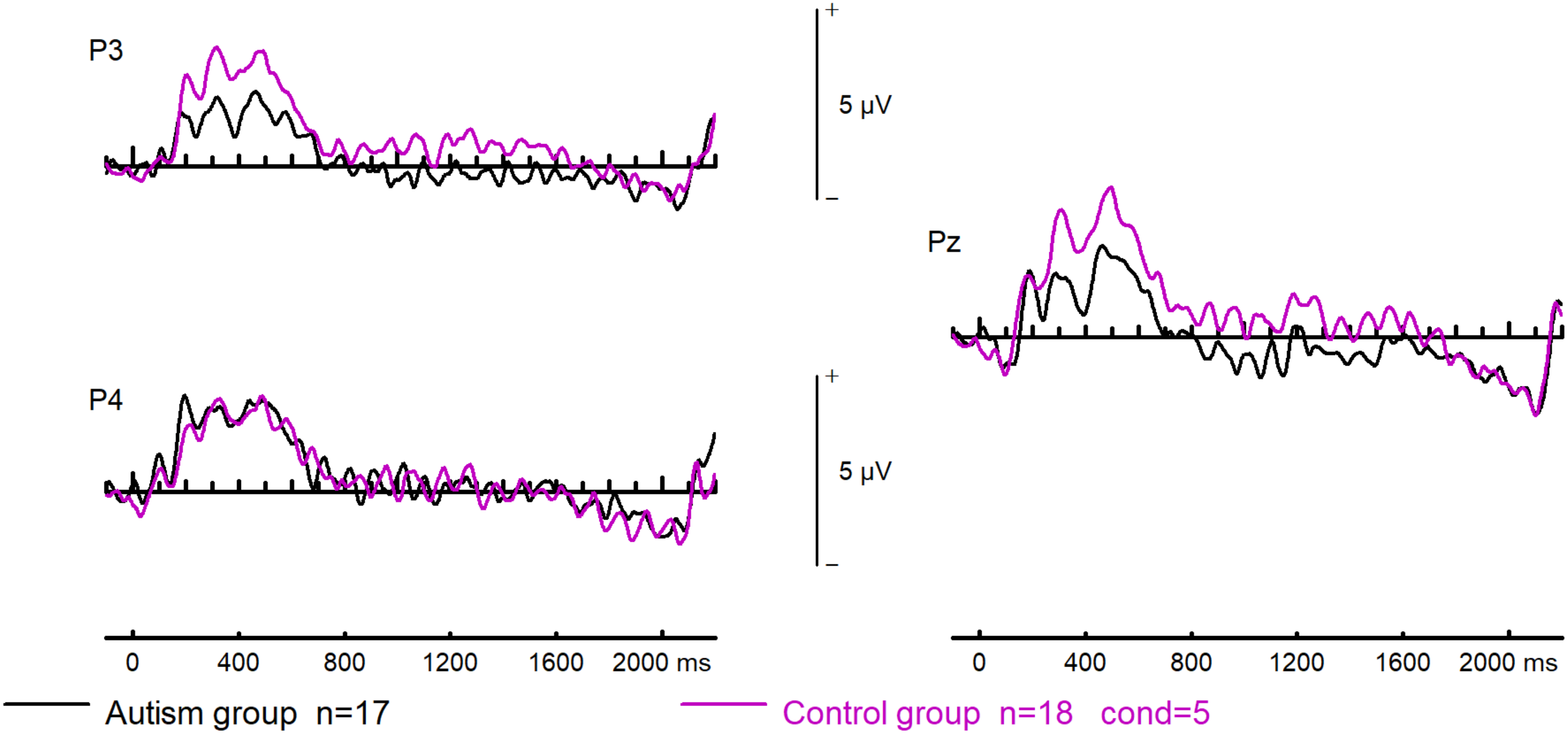

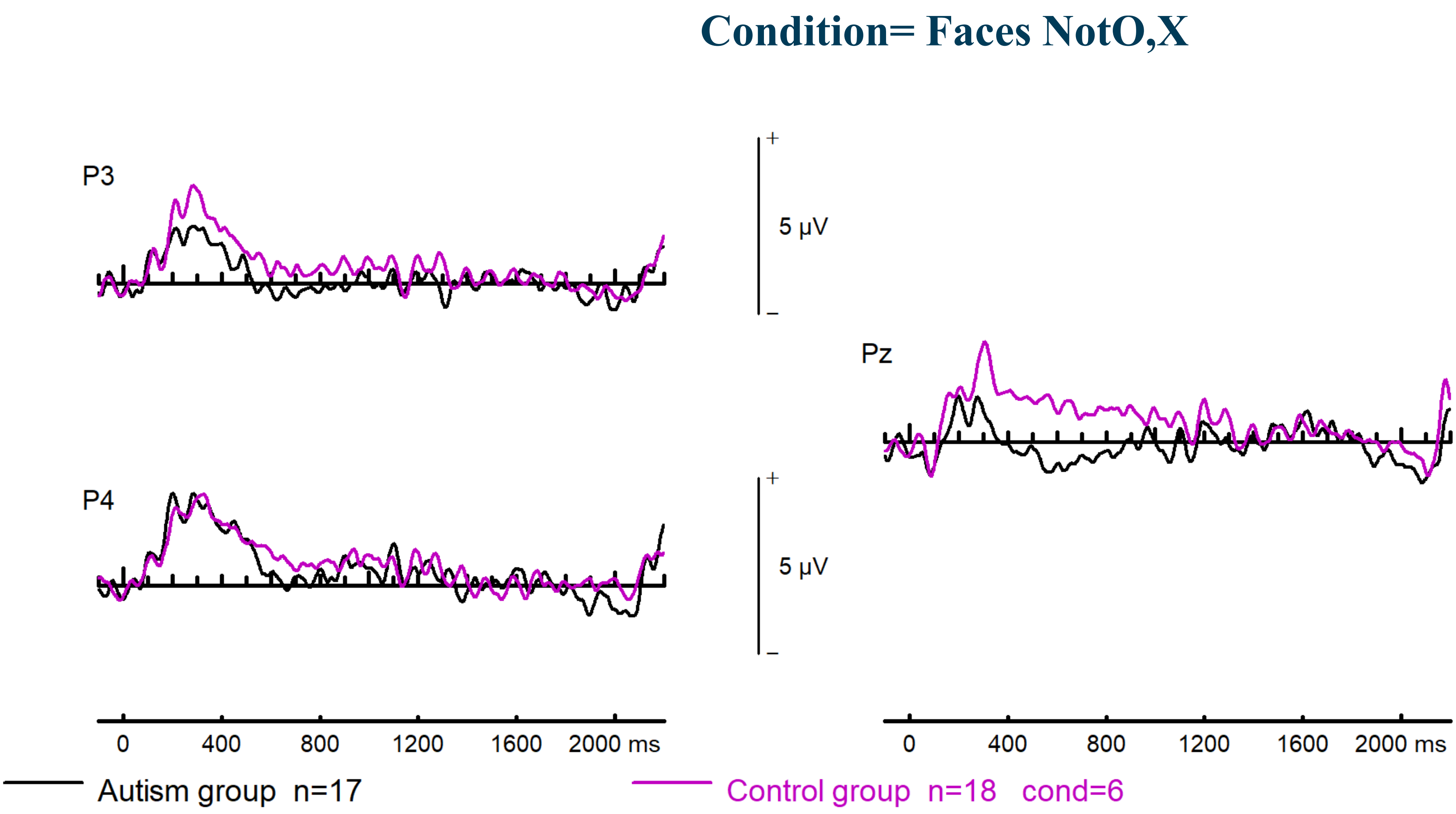
Condition= Faces NotO,X
P3
P4
Pz
+
5 µV
–
+
5 µV
–
0
400
800
1200
1600
2000 ms
0
400
800
1200
1600
2000 ms
Autism group n=17
Control group n=18 cond=6

Condition= Faces O, Not X

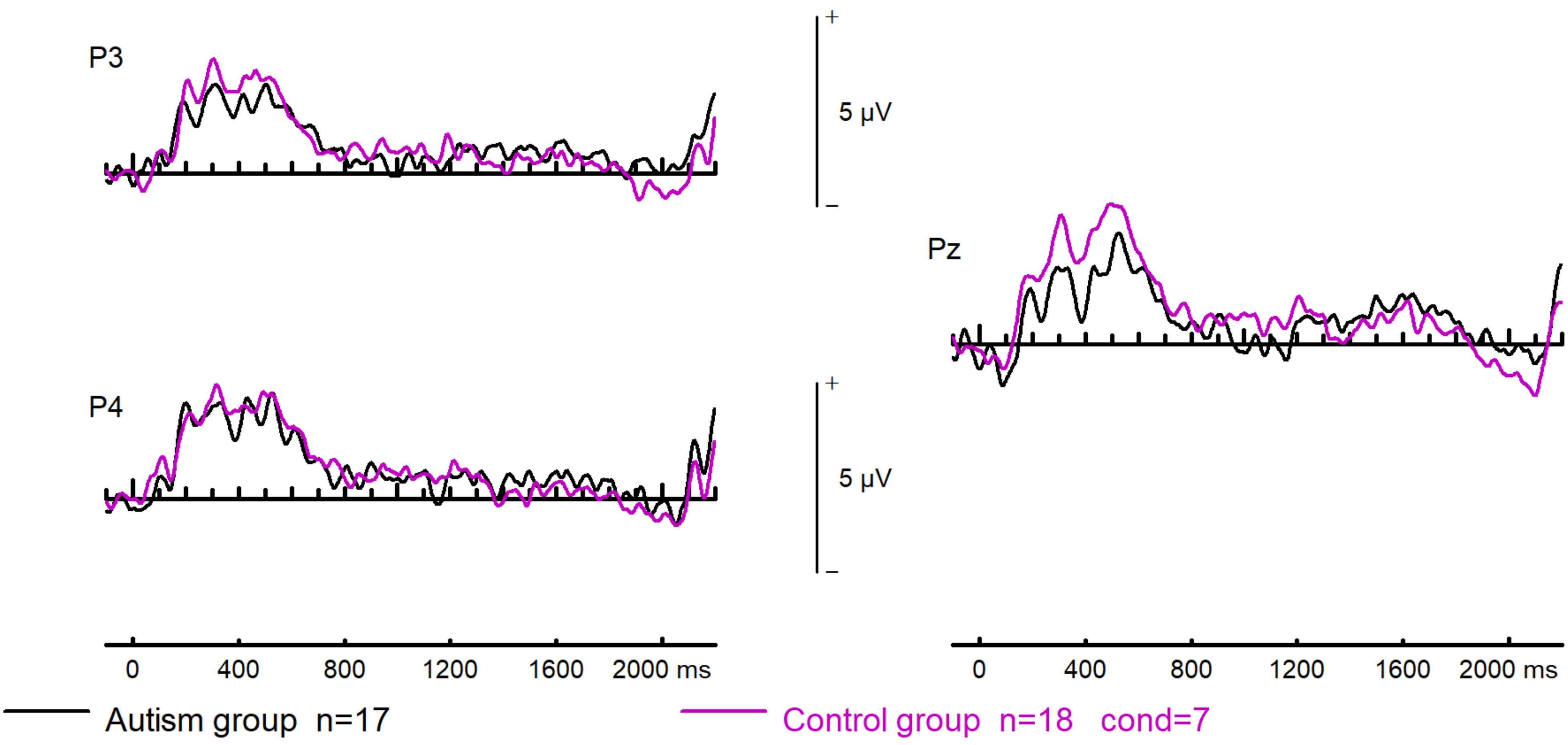

Condition= Faces NotO, NotX

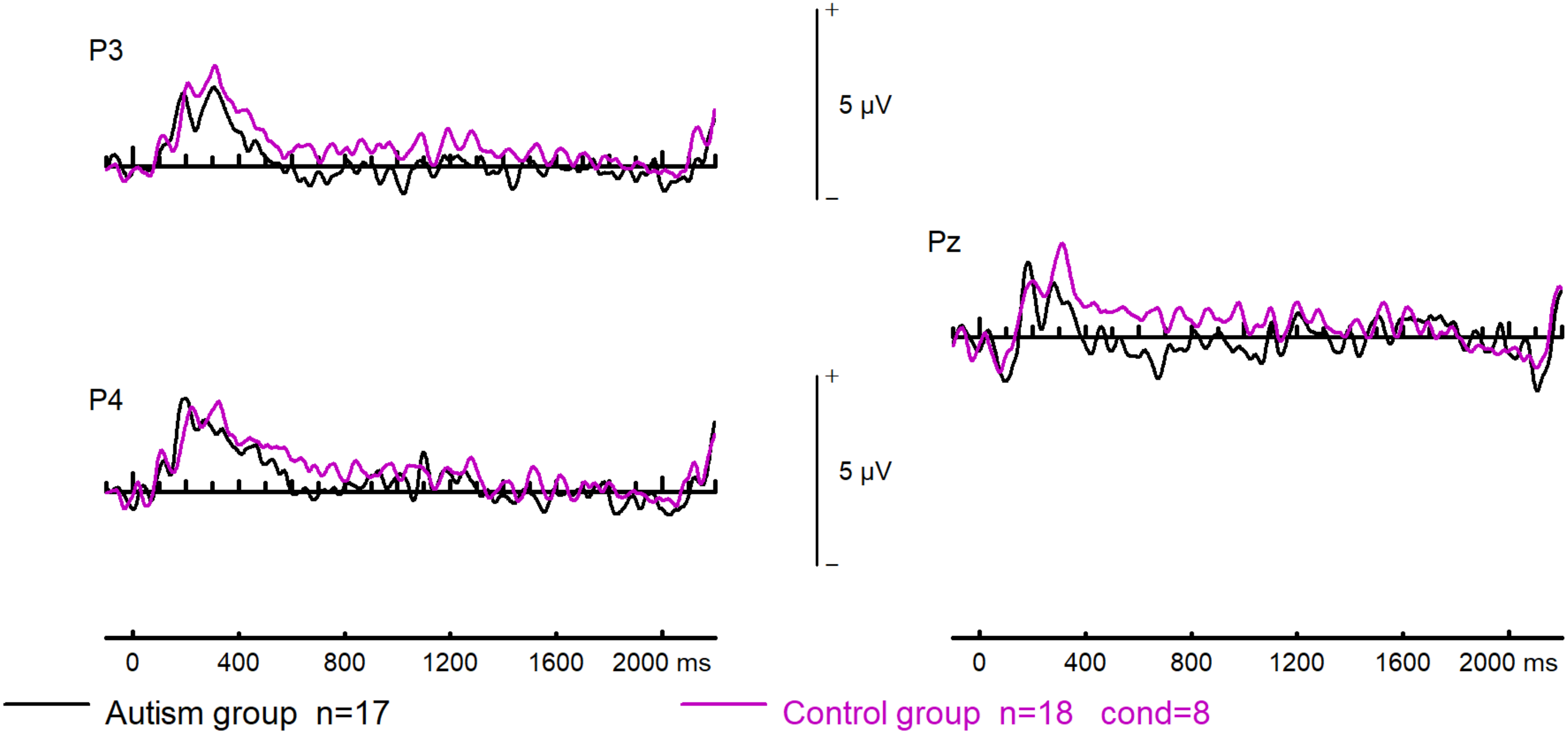

Supplement: Supplementary file 1 — Supplementary file1 (PDF 6366 KB) [file 10803_2025_6825_MOESM1_ESM.pdf]
